# “On a tree”, “terrestrial”, or “on the rocks”? Habit diversity in the megadiverse genus *Peperomia*

**DOI:** 10.1111/plb.70214

**Published:** 2026-05-13

**Authors:** J. Y. L. Tay, G. Zotz, M.‐S. Samain

**Affiliations:** ^1^ Institute for Biology and Environmental Sciences, Functional Ecology Carl von Ossietzky Universität Oldenburg Oldenburg Germany; ^2^ Smithsonian Tropical Research Institute Panama City Panama; ^3^ Red de Diversidad Biológica del Occidente Mexicano, Centro Regional del Bajío Instituto de Ecología, A.C. Pátzcuaro Mexico

**Keywords:** Epiphytes, habit, habitat generalist, habitat specialist, interspecific variation, intraspecific variation, lithophyte, *Peperomia*

## Abstract

- Categorical thinking conceptualizes continuous gradients into distinct entities and is problematic because it obscures the natural variation that exists within and between members of a category. A case in point is the common practice of sorting species into different habits, that is, epiphytic, lithophytic, or terrestrial. This work aims to emphasize that habitat use should be treated as a continuous gradient to avoid missing biological variations and diversity.
- We focused on the genus *Peperomia* and collected relevant information from >400 sources, including online databases, flora checklists, species descriptions, type specimens, and herbarium vouchers. Literal habitat descriptions were translated into numerical proportions that allowed for a quantitative analysis of the tendency to grow on either substrate, and to depict the distribution of *Peperomia* species in the epiphyte–lithophyte–terrestrial (ELT) space.
- Triangular ordinations allowed the visualization of the habits of 1375 species at the genus and subgenus level and portray geographical differences. Species were distributed across the ELT space and not limited to the extremes as specialists, which we defined as species that occur (almost) exclusively in one habitat. Online databases with oversimplified habitat descriptions do not adequately present the existing inter‐ and intraspecific variations in habitat use.
- We present the arguably most comprehensive and up‐to‐date database on *Peperomia*, which will be an important source for future ecological and phylogenetic studies. The main takeaway message that is also applicable to other taxonomic groups is that our numerical approach showed succinctly that habitat use should not be categorized but instead viewed as continuous to properly capture biological variabilities such as inter‐ and intraspecific variation.

## Graphical abstract

The plasticity of a large proportion of *Peperomia* species to occupy multiple types of microhabitats is not well documented due to the typically rigid categorization of habitat use. Therefore, the numerical approach presented is methodologically innovative to advance ecological data integration to provide a more realistic visualization of the habitat use in the pantropical genus.

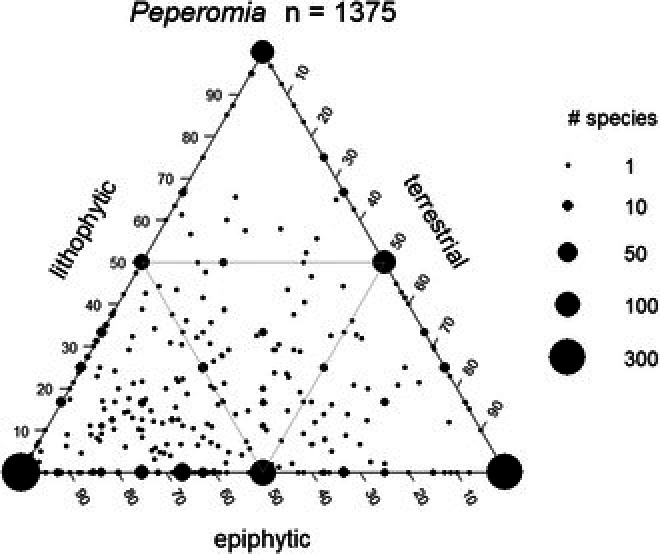

## INTRODUCTION

Labels and categories are social constructs that help to simplify complex realities for easier communication. For example, in botany, broad categories such as evergreen *versus* deciduous, annual *versus* perennial, or C3 *versus* C4 plants are created to make the complexity and variation that exist in plant diversity more manageable. However, categorical thinking, which conceptualizes frequently continuous gradients into distinct entities, can be problematic. It could (i) overstate similarities within categories, (ii) amplify differences between members of other categories, and (iii) treat labels to be static (de Langhe & Fernbach 2019). Therefore, it becomes easy to overlook the natural variation within and between categories.

In a relevant ecological context, an example is the common practice of sorting species based on their habitat use into the categories: ‘epiphytic’, ‘lithophytic’ or ‘terrestrial’. Epiphytes are typically described as ‘non‐parasitic plants that grow on another plant throughout their lives, without contact to the ground’ (Zotz 2016). Lithophytes (synonymous terms are saxicole, rupicolous or epipetric) are defined as plants ‘that grow on rock and derive their nourishment chiefly from the atmosphere’ (*The American Heritage Science Dictionary* 2005). Terrestrials are plants that root in soil on the ground. However, can species really be placed unambiguously into one of these three groups? Typically, lithophytes and epiphytes grow on largely impenetrable substrate (rock or tree bark, respectively) with resulting challenges of anchorage and procurement of water and nutrients. Nevertheless, their growing conditions can resemble those of terrestrial plants, for example, in montane tropical forests when organic material accumulates on tree branches, or on moss covered rocks. Therefore, while an individual plant can be categorized, it is impossible to show that all individuals of a species, past, present and future, use only one type of substrate.

Yet, another factor influencing the degree of ‘epiphytism’ of a given species is regional variation (Barberis *et al*. 2021; Taylor *et al*. 2022). The global distribution of epiphytes shows strong latitudinal patterns, with highest richness in tropical regions (Zotz 2016). Within the Neotropics, the diversity of epiphytic flora also changes across an elevation gradient, with a higher representation in cloud forests at intermediate elevations (Gentry & Dodson 1987). Furthermore, intraspecific variation in habitat use may also vary geographically. For example, ferns of the *Polypodium vulgare* complex are typically terrestrial but can be predominantly epiphytic in some regions (Johnson 1921; Klinghardt & Zotz 2021), and the tank bromeliad *Nidularium procerum* is usually epiphytic but may grow terrestrially in swampy areas in the lowlands in the Atlantic Forest (de Freitas *et al*. 2003). These examples illustrate the ambiguity of assigning strict habitat categories to pigeon‐hole species' habit. Moreover, the terms ‘epiphytic’, ‘lithophytic’ or ‘terrestrial’ are often used inconsistently in the literature and databases. For instance, EpiList 1.0 (Zotz *et al*. 2021) is a global compilation of epiphytes which includes any species mentioned as an epiphyte in at least one reference. However, such a list inevitably pools species with very different degrees of fidelity to the epiphytic habitat, that is, facultative and obligate epiphytes.

**Fig. 1:**
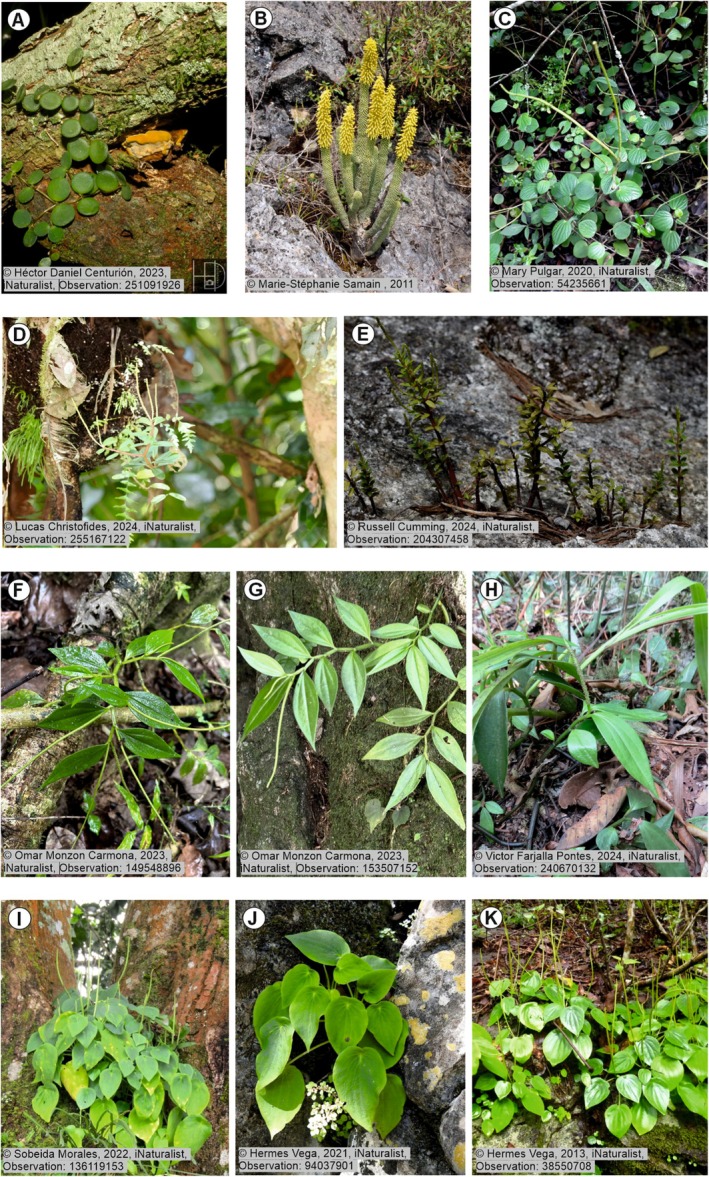
Photographs of *Peperomia* species in their diverse habitats. (A) *Peperomia delicatula* (100% EV) on a branch. (B) *Peperomia cereoides* (100% LV) on a boulder. (C) *Peperomia miqueliana* (100% TV) on the ground. (D, E) *Peperomia enervis* (50% EV, 50% LV), (D) on a branch, and (E) on a boulder. (F–H) *Peperomia alata* (54% EV, 31% LV, 15% TV) (F) on a branch, (G) a boulder, (H) on the ground. (I–K) *Peperomia lanceolatopeltata* (38% EV, 47% LV, 15% TV), (I) in the crotch of a tree, (J) between boulders, (K) on the ground with rocks. EV, LV, TV refer to the Epiphytic Value, Lithophytic Value, and Terrestrial Value, respectively. The EV, LV, and TV values were obtained from our database, indicating the tendency towards occupying a certain habitat (Table S2).

Recently, Zotz & Einzmann (2023) and Zotz *et al*. (2023) used a literature‐based approach to quantify the degree of fidelity of several genera of ferns and lycophytes to epiphytic, lithophytic, and terrestrial habitats. The current study applies the same approach to the megadiverse genus, *Peperomia*, that has more than 40% of its species (ca. 620 out of 1464) categorized as epiphytic in EpiList 1.0 (Zotz *et al*. 2021). *Peperomia* species occur pantropically with their main diversity (>1000 species) in the Neotropics, span a wide elevational range and their habitat use is exceptionally diverse, with many species occupying multiple habitats (Fig. 1), for example, species that have traditionally been categorized as epiphytes may also grow as terrestrials and/or lithophytes, and vice versa. This makes the genus an ideal model for the present study, to illustrate that strict category obscure biological variation. This work provides a comprehensive database on *Peperomia* with a more realistic view of the use of epiphytic, lithophytic, and terrestrial habitats. More specifically, we aim to evaluate information presented in large publicly available epiphyte/plant databases (*i.e*., POWO, EpiList 1.0), whether those databases properly capture the gradient of habitat use within this genus, and if there are differences in *Peperomia* habitat use associated to geographical region. It is expected that habitat use across *Peperomia* species form a continuous gradient within the epiphyte–lithophyte–terrestrial space and that existing global databases fail to capture this variability. The data of this manuscript will be highly relevant for researchers who are interested in exploring the flexibility in habitat use shown in *Peperomia* from a taxonomic and/or evolutionary point of view.

## MATERIALS AND METHODS

This study follows the approach used in two recent publications (Zotz *et al*. 2023; Zotz & Einzmann 2023). Information on habitat use (*i.e*., on trees, rocks or soil) of almost all accepted and known species of *Peperomia* was collected from the taxonomic literature (species descriptions, checklists, floras), herbarium vouchers of type specimens, ecological literature (community studies, vegetation descriptions) and numerous online databases and websites, for example, Brazilian Flora (Carvalho‐Silva *et al*., n.d.) or Flora of China (eFloras 2008). An important criterion to accept a source for this purpose was that it must acknowledge that a species may occur on different substrates. This was mostly not stated explicitly, but assumed when species lists included at least one species as found on multiple substrates, for example, ‘species × is found growing on trees (*i.e*., epiphyte) and on rocks (*i.e*., lithophyte)’. Excluded sources were for example, studies that exclusively focused on vascular epiphytes (*e.g*., Hietz & Hietz‐Seifert 1995; Einzmann *et al*. 2015), unless the authors specifically included accidental or facultative epiphytes (*e.g*., Werner *et al*. 2005), and studies in which all mentioned species were assigned to a single substrate category (*e.g*., Freitas *et al*. 2016; Jiménez López *et al*. 2023). Although this conservative approach inevitably excludes some valid information (*i.e*., genuine habitat specialization), the inclusion of such studies would bias the database towards extreme values of habitat use. For example, if we include studies on vascular epiphytes, it will inevitably inflate our estimate of ‘epiphytes’. Therefore, the exclusion of those sources will make sure that any specialization patterns that emerged from our analyses were the direct result of careful literature and expert‐mediated synthesis. For species with no or very little information on habitat use from the obtained sources, information from the protologues or the type vouchers from herbarium collections was used, and in a few cases collections in which the last author of this study (Samain) was involved and non‐type vouchers.

An actual quantification of epiphytic, lithophytic, or terrestrial occurrences of the individual plants of a given species in a study is exceedingly rare (*e.g*., Acuña‐Tarazona *et al*. 2015). Thus, in almost all cases the literal descriptions of occupancy of an epiphytic, lithophytic, or terrestrial habitat by a given species had to be translated into numerical proportions (Table 1), following Zotz & Einzmann (2023) and Zotz *et al*. (2023). This approach yielded three values per entry – Epiphyte Value (EV), Lithophyte Value (LV), and Terrestrial Value (TV) – which sum up to 100% (Table S1). A pivot table was then produced, with all sources weighted equally to calculate the average EV, LV, and TV for each species (Table S2). Similar to Grime's triangle that defines three primary plant strategies (competitive, stress‐tolerant, ruderal) based on their responses to environmental pressure (Grime 1977), habitat use by *Peperomia* species can be treated as a continuous variable and visualized in an epiphyte–lithophyte–terrestrial (ELT) space (Workflow and code are provided as Fig. S3). There are some caveats to acknowledge here: it is impossible to convert descriptions such as ‘epiphytic and lithophytic’, ‘mostly terrestrial’ and ‘rarely epiphytic’ into precise proportions and these terms are also used inconsistently among authors. However, converting the terms into a numeric system allows for a coarse ranking of the tendency or likelihood of a species to be found in an epiphytic, lithophytic, or terrestrial habitat and represents a clear improvement from simple categorical assignments (Zotz *et al*. 2021). The rules in Table 1 were applied consistently in our article. However, they can be modified if there were objective arguments against the used scheme, making this approach transparent, flexible and adaptable for future analyses.

**Table 1:** Numerical translation system of written descriptions of species occurrences in the original sources as epiphyte value (EV), lithophyte value (LV), or terrestrial value (TV) used in the large majority of all sources.

written information in source	translated numeric system for epiphyte occurrence (=EV)
Epiphyte and lithophyte (50% to LV)/epiphyte or terrestrial/facultative epiphyte (50% to TV)	50
Epiphyte, lithophyte or terrestrial (33%/34% to LV and TV)	33
Holoepiphyte/hemiepiphyte/epiphyte	100
Obligatory epiphyte	100
Rarely (95% to alternative)	5
Very rarely/accidental epiphyte/exceptionally	1
Also as/sometimes/occasionally/uncommonly	15
Commonly/generally/mainly/mostly/preferentially/primarily/usually	85
Often/frequently/most frequently	67

As discussed in the previous publications (Zotz *et al*. 2023; Zotz & Einzmann 2023), the final average EV, LV and TV of any given species should be seen as a rough estimate of all sources that were weighted equally, and resulting values should be interpreted as the result of a structured synthesis of literature‐based information and the ecological interpretation is likely to be species dependent and not straightforward for all taxa. For example, an entry of 50% epiphyte/50% lithophyte for a species in the final results (Table S2) can be due to a number of reasons: (i) a single source may be available that describes the species as, for example, ‘growing on tree trunks and rocks’, (ii) there may be two or more studies, half of which describe the species as ‘growing on tree trunks’, the others as ‘growing on rocks’, or other possible combinations. Such differences could reflect true biological diversity describing regional differences in occurrence patterns as reported, for example, for the bromeliad *Nidularium procerum* (de Freitas *et al*. 2003), but may also reflect erroneous observations in some sources. We explored how much a deviation of true habitat use from a 50%/50% translation would affect our overall results with a sensitivity analysis using a custom‐made programme (Noreen 1989). Briefly, for the cases of 50%/50% in two habitats in the original dataset, the numbers were replaced by randomly picking combination of two numbers from a uniform distribution ranging from 25 to 75, and a modified dataset. A pivot table was produced to calculate the modified mean EV, LV and TV of those species. We then estimated the number of species with a mean EV, LV and TV value of >50% from the modified dataset. This procedure was repeated 1000 times. Finally, by discarding the 25 lowest and 25 highest values for either of the three cases of >50%, we produced confidence intervals, which were compared with the estimates derived from the original dataset.

Zotz & Einzmann (2023) and Zotz *et al*. (2023) interpret values of EV, LV or TV >50% as an indicator of habitat preference. However, the term ‘preference’ is typically used to describe active host selection behaviour (*e.g*., Desjardins *et al*. 2010; Takken & Verhulst 2013), and is less applicable to sessile organisms (Zhou & Hyde 2001). Demonstrating true habitat ‘preference’ would involve a series of germination experiments and long‐term monitoring of survival and establishment. Without this information, a seeming ‘preference’ for a certain habitat might simply be due to the lack or unavailability of other habitat types. Therefore, we avoid the term preference and instead refer to higher tendency or likelihood of occurrence in a given habitat.

Initially, the list of *Peperomia* species was compiled using the World Flora Online database (WFO 2025). All species names were then standardized by the last author (Samain), an expert on *Peperomia*, following the International Plant Names Index (IPNI 2025), Tropicos (2025) and peperomia.net (Mathieu 2001–2023). Geographic distribution was obtained from Plants of the World Online (POWO 2025), which uses the phytogeographical international working group on taxonomic databases for plant sciences (TDWG) standard for recording plant distributions for its geographical search (Brummitt 2001). The geographical distribution data were binary (presence/absence). First, the data were compiled at the country level, then further condensed into continental level, following the continental scheme following TDWG world geographical scheme for recording plant distribution (Brummitt 2001, 19). In short, if a country within the continental level recorded the presence of species A, then the whole continental level was recorded with the presence of species A. The geographical distribution and *Peperomia* database were curated by the last author (Samain). This curation involved the following aspects: (i) assigning synonyms to their currently accepted species, (ii) correcting orthography of species names (otherwise some species with different spellings would have been included twice), (iii) checking and confirming whether a species actually occurs in one or more geographical areas, based on knowledge of the genus and herbarium information. If data were added, this implied additional lines of entry; no previously collected information was replaced. The curation process can be found in Table S4.

To visualize the habit diversity of *Peperomia* species, the approach by Franco & Silvertown (1996) to ordinate demographic components in a triangular space was used. Similar to the elasticity values obtained from matrix analyses in population biology, the epiphyte value (EV), lithophyte value (LV) and terrestrial value (TV) of each species sum up to unity, hence making it possible to explore the distribution of *Peperomia* species in the epiphyte–lithophyte–terrestrial (ELT) space. To produce those triangular ordinations, the plotrix library version 3.8‐1 (Lemon 2006) in R version 4.2.0 (R Core Team 2025) was used.

## RESULTS

The compiled database included more than 4300 entries of 1464 species from 419 sources. No habitat information was available for 89 very rare species, which were generally only known from the historical type collection which lacked information on habitat use. Hence, the database contains information on habitat use for 1375, that is, ca. 94% of the known *Peperomia* species (Table S1). Names used in the original publications can be found Table S1.

### Interspecific and intraspecific variation in habitat use

**Fig. 2:**
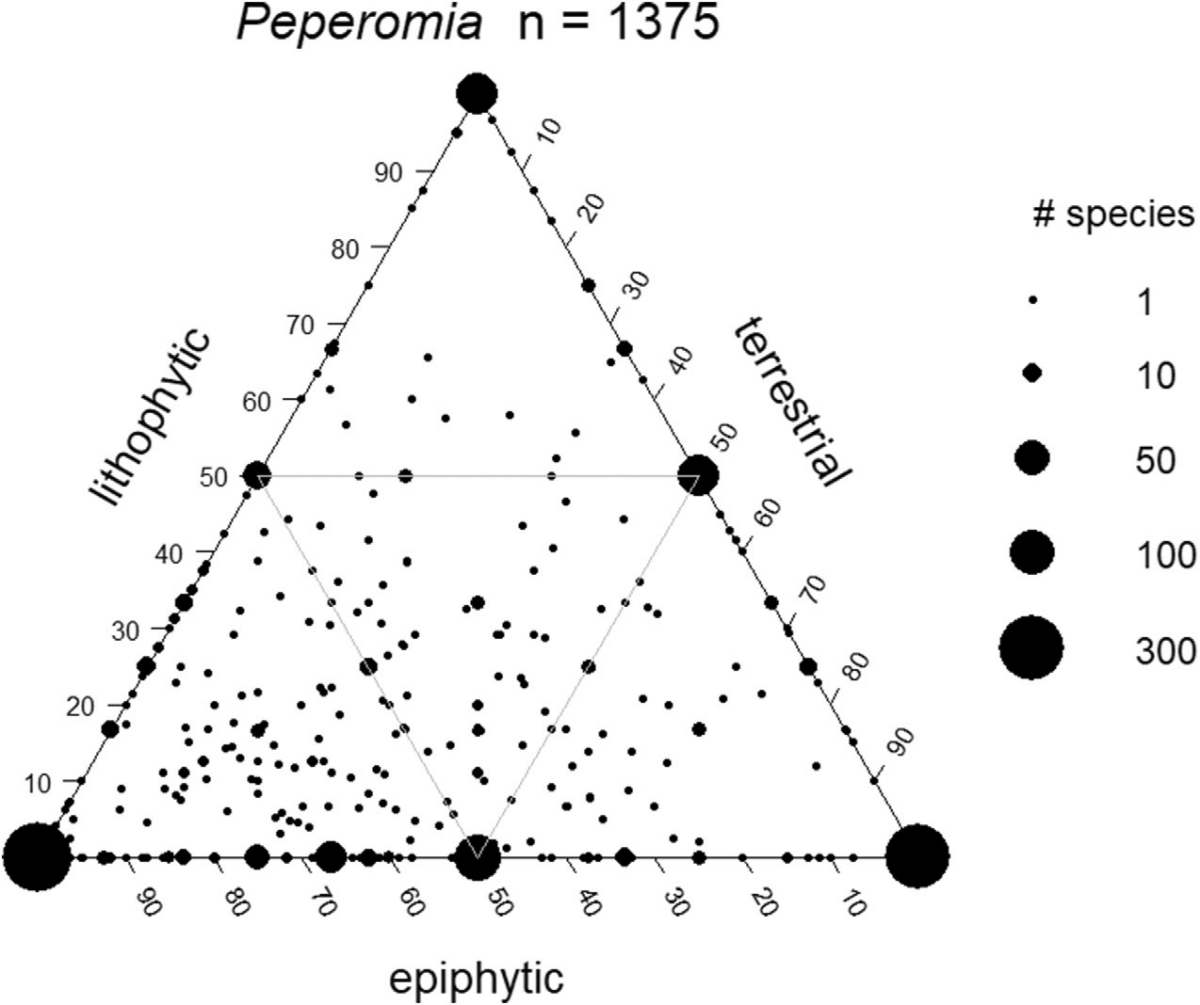
Distribution of 1375 species of *Peperomia* in the epiphyte–lithophyte–terrestrial space. Symbol size varies with the number of species that share the same values. Individual species values are based on 1–64 sources. The grey lines separate the triangle into four smaller ones. The central triangle is occupied by generalists, and the three other triangles indicate the tendency of a species to be found in an epiphytic, lithophytic or terrestrial habitat. The full database is given in Tables S1 and S2.

Triangular ordinations (Fig. 2) allow for visualization of the habitat use of each species in a single analysis. A larger number of sources should improve the quality of the final estimate of habitat(s) use of each species and most of the species (ca. 58%) had information from two or more sources. The species were distributed across the ELT space and were not just limited to the extremes. More than half of the species were specialists, which we define as species that occur (almost) exclusively in one habitat, that is, species in which either EV, LV or TV exceeds 95%. A position in the central small triangle of the ordination identifies generalists, while a position in the other three small triangles indicates the tendency of a species to be found in one of the three habitat types, which we define as cases in which either EV, LV or TV >50% (Fig. 2). Almost 80% of all species had a tendency to occupy primarily one of the three habitat types, that is, 42%, 9% and 28% of all species showed a tendency to grow in an epiphytic, lithophytic and terrestrial habitat, respectively.

The 1000 modified datasets, in which each case of 50%/50% in two habitats in the original dataset was replaced with a combination of two random numbers between 25 and 75, allowed us to obtain a 95% confidence interval for the tendency to occupy either of the three habitats (Table 2). The modified datasets showed that our 50%/50% assignment slightly but consistently underestimated the percentage of species with a tendency to occupy a certain habitat.

**Table 2:** Percentages of species that showed a tendency towards occupying a certain habitat (EV, LV and TV > 50%) in the original dataset and the 95% confidence interval produced from the 1000 iterations of modified datasets.

habitat use	species with tendency to occupy certain habitat (%) – original data set	species with tendency to occupy certain habitat (%) – modified data sets (95% CI)
EV >50%	42	[43.89, 43.94]
LV >50%	9	[11.86, 11.90]
TV >50%	28	[31.26, 32.30]

**Fig. 3:**
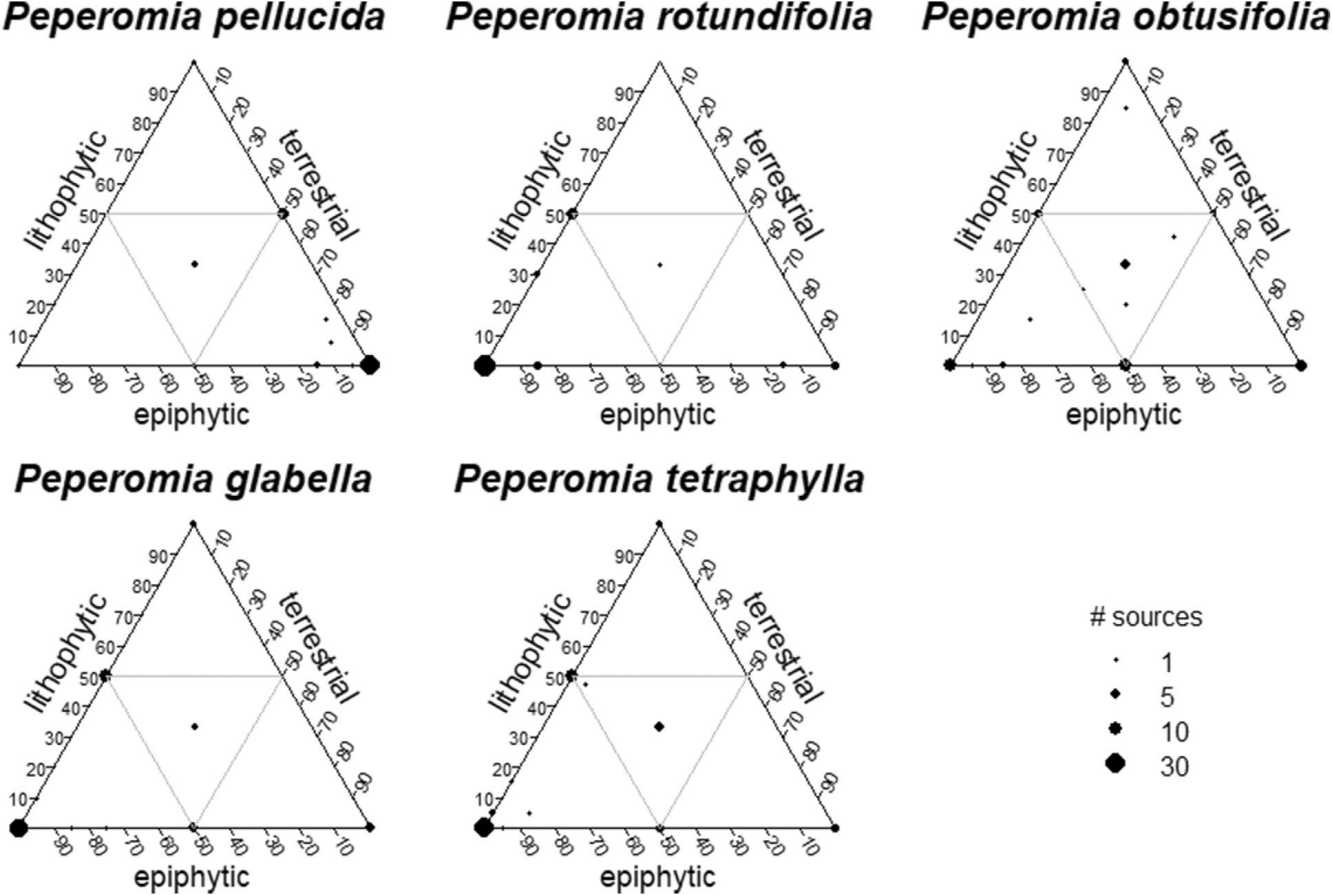
Distribution of five species of *Peperomia* in their respective epiphyte–lithophyte–terrestrial space. Symbol size varies with the number of species that share the same values. Individual species values are based on 48–64 sources. The grey lines separate the triangle into four smaller ones. The central triangle is occupied by generalists, and the three other triangles indicate the tendency of a species to be found in an epiphytic, lithophytic or terrestrial habitat. The full data set is given in Tables S1 and S2.

In the database, of those species that had information on habitat use, 38% of them had information from only one source – either from the type specimen or from just one location. Therefore, information on intraspecific variation in habitat use is very limited in such cases. Nonetheless, out of 62% of the species that had two or more sources with habitat information, five species had ≥48 sources with habitat information (Fig. 3). None of them was strictly found in one habitat. For example, more than 70% of the 52 sources with information on *Peperomia pellucida* indicated that the species was almost exclusively found in terrestrial habitats. However, eight sources indicated that the species was growing both terrestrially and lithophytically (*e.g*., Fosberg & Sachet 1975; Diniz 1996; Saralegui 2004), one source even reported exclusive lithophytic growth (Millspaugh 1902) and three sources reported occurrences in all three habitat types (Backer & van den Brink 1963; Huber 1987; Espejo‐Serna *et al*. 2021). The other four species also showed intraspecific variation (Fig. 3), suggesting that all were opportunistic regarding their growing site.

### Verifying species habitat use information in EpiList 1.0 and POWO

**Fig. 4:**
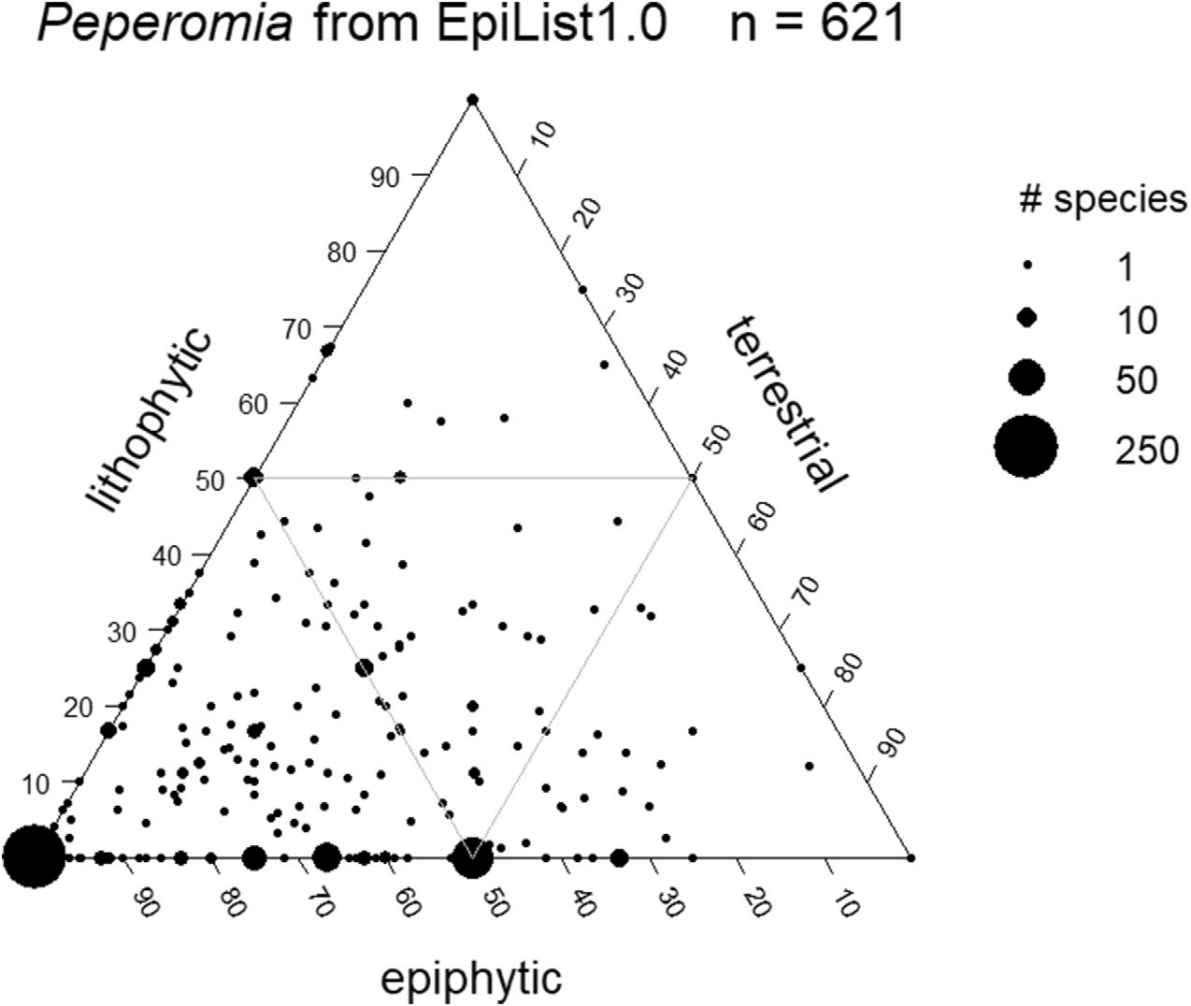
Distribution of 621 species of *Peperomia* (listed as epiphytes in EpiList 1.0) in the epiphyte–lithophyte–terrestrial space. Symbol size varies with the number of species that share the same values. The grey lines separate the triangle into four smaller ones. The central triangle is occupied by generalists, and the three other triangles indicate the tendency of a species to be found in an epiphytic, lithophytic or terrestrial habitat. The full database is given in Tables S1 and S2.

Our database also allows us to critically review previous categorizations of 621 *Peperomia* species as epiphytes in EpiList 1.0 (Zotz *et al*. 2021). As expected, the majority of them indeed have the tendency to occupy epiphytic habitats (Fig. 4). However, more than 10% of the species were found outside the lower left triangle, that is, were not predominantly growing epiphytically. Most noteworthy, there were six species for which none of the used sources in our database recorded an epiphytic habitat (number of references for habitat information and main habitat use are listed in the brackets): *Peperomia condoris* (1, terrestrial), *P. decurrens* (2, lithophytic), *P. guamana* (1, lithophytic), *P. merrillii* (1, lithophytic), *P. rockii* (2, terrestrial) and *P. santa‐elisae* (2, terrestrial and lithophytic).

**Fig. 5:**
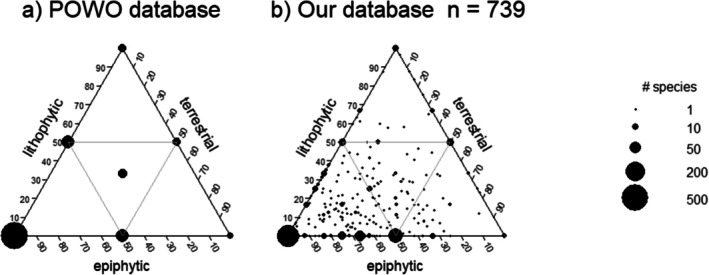
Distribution of 739 species of *Peperomia* (with habitat information from POWO) in the epiphyte–lithophyte–terrestrial space. Symbol size varies with the number of species that share the same values. The grey lines separate the triangle into four smaller ones. The central triangle is occupied by generalists, and the three other triangles indicate the tendency of a species to be found in an epiphytic, lithophytic or terrestrial habitat. The full data set is given in Tables S1 and S2.

We also compared the habitat information for 739 species given in POWO, a large publicly available database, with our data. Habitat information in POWO is based on seven broad descriptions, that is, epiphyte, lithophyte, terrestrial, a combination of two different habitats, and a combination of all three habitats. Consequently, the resulting triangular ordination only showed seven points across the ELT space (Fig. 5a) and indicated very little interspecific variation in habitat occupancy with a majority of the species restricted to epiphytic habitats (70%). A direct comparison of the same 739 species with our database (Fig. 5b) showed the same strong tendency towards occupying the epiphytic habitat, although variation in habitat use is much more heterogeneous and gradual.

### Regional differences in habitat use

The following analysis explored the distribution of the species according to geographical distribution (Table 3 and Fig. S5). Most *Peperomia* species are found in South America. In this region, about 50% of the species are habitat specialists, with about 70% showing a tendency towards either epiphytic or terrestrial growth. More than 1200 accepted species can be found in North, Central, and South America whereas other geographical regions have fewer than 100 species (Table 3). In most of the other geographical regions except Australasia, species primarily tend to be found in epiphytic habitats. A more detailed map on species habitat use based on regional distribution is shown in the Fig. S6 and Table S7.

**Table 3:** Percentages of species that are either specialists (EV, LV, and TV > 95%) or show a tendency towards a certain habitat (EV, LV and TV > 50%) for eight geographical regions.

geographical regions	specialist (value > 95) (%)			tendency to occupy certain habitat (value >50) (%)		
	epiphyte	lithophyte	terrestrial	epiphyte	lithophyte	terrestrial
South America (*n* = 879)	20	3	29	36	4	37
North America (*n* = 129)	16	9	5	52	15	12
Central America (*n* = 282)	23	7	6	54	9	14
Pacific (*n* = 91)	27	15	12	45	20	18
Asia Tropical (*n* = 79)	53	8	0	67	14	4
Asia Temperate (*n* = 12)	17	8	0	42	33	8
Africa (*n* = 58)	46	7	9	67	9	14
Australasia (*n* = 6)	0	17	0	17	50	17

The values in parenthesis indicate the number of species from our database that are found in each region. Mexico is included in North America and the Caribbean is included in Central America (Fig. S3).

## DISCUSSION

This study analyses the diverse habitat use by *Peperomia* species based on both qualitative‐to‐quantitative translation of habitat description from literature‐based information and expert‐mediated synthesis that involved taxonomic curation and synonym resolution, and visualizing habitat use with triangular ordinations. This approach offers a much more realistic representation of the biological diversity in terms of habitat use than broad categorical labels, and advances ecological data interpretation. Our numerical approach largely confirms previously proposed habitat use at a global level with respect to geographic location (Table 3). Habitat diversity is especially high in the Neotropics, with South America having the highest proportion of specialists for terrestrial habitats (Table 3), which is consistent with the hypothesis that *Peperomia* originated from the tropical Andes (Symmank *et al*. 2011) with the terrestrial habit as the most likely ancestral state (Frenzke *et al*. 2016). The widespread distribution of the genus is truly remarkable, ranging from sea level to nearly 5000 m a.s.l. in Peru, where the highest species diversity of the genus can be found (Pino Infante *et al*. 2023). Nonetheless, geographic regions themselves do not influence habitat use tendencies, but rather reflect species‐specific dispersal abilities. The visual and quantitative representations (Table 3 and Fig. S5) are thus valuable tools to improve our understanding of the geographical patterns of habitat use within the genus throughout its distribution area.

Nonetheless, there are some caveats of our numerical approach, with cases when we cannot know the exact proportions, especially regarding the 50%/50% assignment to two habitats. In the analysis with modified datasets (Table 2), where all cases of 50%/50% assignments to two habitats were replaced by two random numbers between 25 and 75 across 1000 iterations, none of the random draws returned exactly 50. Thus, as ‘tendency’ is defined by an EV, LV or TV value >50%, a higher proportion of species with a tendency towards a certain habitat was actually inevitable. This highlights the need for continued refinement of our database. Currently, for lack of a better alternative, we slightly, but systematically, underestimate the proportion of species showing a tendency towards a certain habitat by translating a description of, for example, ‘epiphytic and lithophytic’ into EV = LV = 50. It is emphasized here that the ELT values should be taken as indicative tendencies and not as precise proportions. Nonetheless, Table S1 can be readily updated with newly verified data, enabling an increasingly realistic representation of the gradient of habitat use in *Peperomia*.

### Comparison between current analysis and large floristic databases – EpiList 1.0, POWO and checklists

In a triangular ordination of *Peperomia* species listed as epiphytes in EpiList 1.0 (Zotz *et al*. 2021) (Fig. 4), all data points should *ideally* be found in the lower left triangle as an indication of a tendency to occupy primarily epiphytic habitats (EV >50%). However, this was only true for 90% of the 621 listed species. Such a discrepancy largely reflects difference in the approach used to compile the database. EpiList 1.0 was compiled as a *positive* list based on any credible source that called species × an epiphyte, whereas our database weighs information from all available sources. However, this explanation does not hold for a few species in EpiList 1.0 for which we found no report on epiphytic occurrences at all (Fig. 4), indicating human error. This underscores that production of any species lists is always ‘work in progress’. We are convinced that our numerical system (Table 1) and the compilation of the details in the Data S1 provide the possibility of including all types of growth habitats from multiple literature sources that are essential for capturing natural intraspecific and interspecific variations in habitat use (discussed in detail in the next section).

The Plants Of the World Online (POWO) database is a valuable, freely accessible resource for the scientific community with information on currently accepted plant names and users can easily build checklists (POWO 2025). However, the limitation of such a huge database is that the data resolution is undoubtedly coarse or oversimplified. This is clearly true for life form/habit information: for >700 *Peperomia* species with habitat information, descriptions were simple and limited to seven broad categories (indicated by the points in Fig. 5a). These categories overemphasize epiphytism in *Peperomia* and obscure inter‐ and intraspecific variation. When more detailed habitat descriptions from our database are incorporated, a more realistic picture emerges (Fig. 5b). Therefore, similar to the caveat of EpiList 1.0, simplifying information into broad categories results in loss of valuable information on biological diversity and habitat choice as an expression of plasticity.

Several floristic checklists or catalogues were used to collate information (*e.g*., Britton 1918; Vareschi 1970; Croat 1978; Brako & Zarucchi 1993), but habitat descriptions were sometimes inconsistent. For example, the monograph ‘Piperaceae of northern South America’ (Trelease & Yuncker 1950) documents habitat information of 306 *Peperomia* species. Some descriptions were specific (e.g, small, terrestrial or rupicolous herb) and interpreted as 50% TV and 50% LV. However, in other cases, the habitat description was just ‘succulent herb’. Although ‘terrestrial’ was not explicitly stated, we assumed that this particular species is growing as a terrestrial plant unless otherwise stated, hence yielding the value as 100% TV. Similarly, the ‘Catalogue of the flowering plants and gymnosperms of Peru’ (Brako & Zarucchi 1993) provides brief entries for 373 *Peperomia* species, with the most detailed descriptions indicating ‘endemic epiphytic or terrestrial herb’, but most simply listed ‘endemic herb’, which could only be interpreted as terrestrial. Fortunately, such ambiguity is uncommon because most of the checklists had detailed information. For example (Wight 1853) in ‘Icones plantarum Indiae Orientalis’, described the habitat use for all *Peperomia* species clearly, for example, ‘growing in thick tufts on moist rocks or branches of trees’, which we interpreted as 50% LV and 50% EV. Similarly, catalogues with specific volumes dedicated to Piperaceae provided detailed habitat use. For example, Flora Mesoamericana: Piperaceae (Callejas‐Posada 2020) provided meticulous habitat descriptions for 224 species, such as ‘herbs, terrestrial and rarely epiphytic’, which we interpreted as 95% TV and 5% EV. Thus, caution is needed when extracting habitat information from general floristic lists, whose primary aim is to document species presence and taxonomy, and often just have overgeneralized or missing habitat details. Some checklists cited in this work were compiled many years ago, the oldest being ‘Nova genera et species plantarum’ by von Humboldt & Bonpland (1815), and many species names are now synonyms of currently accepted names. For example, *Peperomia basellifolia* recorded in Fawcett & Rendle (1914) is now a synonym of *Peperomia acuminata*; *Peperomia balsapuertana* recorded in Macbride (1936) is now a synonym of *Peperomia pernambucensis*. Our database included 392 synonyms corresponding to 154 accepted species, all meticulously curated by the last author (Samain), producing one of the most comprehensive and up‐to‐date databases on *Peperomia* (Table S1).

### Intraspecific and interspecific variation in habitat use

The fundamental goal of this study is to capture natural intra‐ and interspecific variation in habitat use within the megadiverse genus *Peperomia*, using numerous sources from the literature and relevant herbarium material. Five species have at least 48 sources with habitat data (Fig. 3), and none of them is restricted to a single habitat. For example, *Peperomia obtusifolia* appears to be occurring more frequently in both epiphytic and terrestrial habitats (*i.e*., 51.4% EV; 42.5% TV), but there are three sources that recorded a tendency for lithophytic growth (*i.e*., >50% LV) (Ichaso & Guimarães 1984; Guimarães & Giordano 2004; Fondom *et al*. 2009). Such versatility suggests these species are opportunistic in exploiting available growth sites, which may help explain their wide distributions. Notable, *Peperomia pellucida* and *Peperomia tetraphylla*, are found on four continents (excluding Europe and Antarctica), with different tendencies to occupy all three habitats. Their pantropical distributions could be attributed to the dispersal success of *Peperomia* (Frenzke *et al*. 2016). Unlike most vascular epiphytes that disperse via tiny wind‐dispersed propagules, or seeds with wings or coma (Madison 1977), *Peperomia* is one of the few vascular plant genera with its epiphytic plant lineages featuring sticky fruits for epizoochorous dispersal (Frenzke *et al*. 2016), facilitating long‐distance dispersal, although potential dispersing animals are rarely discussed (*e.g*., Valdebenito 1989). *Peperomia tetraphylla* in the subgenus *Pseudocupula* bears sticky fruits with a pseudopedicel, which likely facilitates epizoochorous dispersal by adhesion, contributing to its broad geographical extent, not only across the Neotropics but also in Africa, Asia and Oceania (Frenzke *et al*. 2015, 2016). *Peperomia pellucida* in the subgenus *Peperomia*, despite having non‐sticky fruits that lack protuberances on their surface and having no pseudopedicel, is the only pantropical species in an otherwise subgenus that is mostly restricted to the Neotropics. However, the current distribution of both species may be linked to human activities (Kress & Krupnick 2022), as both species are widely used in folk and traditional medicine (Nishanthi *et al*. 2012; Gomes *et al*. 2022). Whether or not humans have aided the widespread distribution of these plants needs further study.

The triangular ordination provides a clear visual representation that about half of all *Peperomia* species are specialists, that is, occur almost exclusively in one habitat (Fig. 2). Similar proportions of epiphytic and terrestrial specialists (*i.e*., ca. 20%), indicate the genus' versatility in terms of the habitats that the species can colonize. This challenges the over‐emphasis of *Peperomia*, especially in older literature, as predominantly epiphytic. For example, the genus *Peperomia* has been described as follows: ‘*Peperomia* Ruiz & Pavon, Herbaceous plants … *most* species are epiphytic … a few are terrestrial’ (Standley 1937); ‘herbs … terrestrial or *more often* epiphytic’ (Burger 1971); ‘herbaceous, perennial or annual, not climbing but *often* rooting from the nodes and epiphytic’ (Huber 1987). Nonetheless, more recent publications typically acknowledge that many species grow as terrestrials.

### Importance of taxonomic curation and field knowledge

The present study is valuable because it underscores the importance of careful and extensive data curation by a taxonomist familiar with the focal plant group (Samain *et al*. 2023). Beyond the potential issues with habitat data and their interpretation mentioned above, floristic lists may have identifications that are, with certainty, erroneous. For example, authors of floristic studies may be unfamiliar with particular species: for instance, Brako & Zarucchi (1993) described *Peperomia ferreyrae* as ‘epiphytic herb on rocky slopes’, although this species has never been observed as an epiphyte (Samain, pers. obs.), nor any other species of this subgenus (*Fenestratae*), all of which are lithophytic or terrestrial. While occasional accidental epiphytism cannot be ruled out (Hoeber & Zotz 2022), the more likely explanation is terminological confusion. Brako & Zarucchi (1993) did not use the term ‘lithophyte’ in their list, but descriptions like ‘on rocks’, ‘on rocky slopes’ and ‘epiphytic herb on rocky slopes’. The first two clearly indicate a lithophytic habitat, while the last is ambiguous, and we had to assign 50%/50% EV/LV, whereas an expert would assign 100% LV. Therefore, this reiterates that field knowledge based on extensive observations is of great relevance to be able to record the full variation in habitat use. For example, one of us (Samain, pers. obs.) found *Peperomia cavispicata* growing lithophytically near a little stream in one field season (type locality, state of Guerrero, municipality Taxco, Tenango del Paraiso, Chacoalco Spring, Mexico), consistent with many other species in the subgenus *Tildenia*. However, in the following year, the same species was found growing exclusively on trees, presumably because the original habitat had been inundated by flood events. This observation aligns with Mathieu *et al*. (2011), who state that the subgenus *Tildenia* is a poorly known group of geophytic species that can occur above‐ground for a few months each year, usually during the local rainy season. Consequently, locating these plants can be difficult to the untrained eyes, without specific knowledge of where and when to search for them. Therefore, we reiterate that our dataset is valuable because the ELT values are not only the outcome of extensive literature search, but also include the meticulous curation by an expert to ensure the credibility of our semi‐quantitative dataset.

## CONCLUSION AND OUTLOOK

The information compiled here is highly relevant for researchers who are interested in the evolutionary and taxonomic aspects of habitat flexibility shown in *Peperomia*. Our database largely corroborates the statements on habitat use in the infrageneric classification by Frenzke *et al*. (2015). For example, the triangular ordination confirms that members of subgenus *Fenestratae* tend to be found in terrestrial and lithophytic habitats, and those of *Pseudocupula* mainly in epiphytic ones, and to a much lesser extent in terrestrial and lithophytic habitats (Fig. S8 and Table S9). Yet, there are also discrepancies in the subgenus *Micropiper*, which Frenzke *et al*. (2015) characterizes as epiphytic or terrestrial, our data indicate numerous generalists and lithophytes. Similarly, species of the subgenus *Erasmia*, characterized as terrestrial by Frenzke *et al*. (2015), actually have an equal tendency to grow in terrestrial and epiphytic habitats. These deviations highlight the strength of our numerical approach, that is methodologically innovative to advance ecological data integration. By translating qualitative habitat descriptions into a continuous numerical system rather than using fixed categories, our method acknowledges ecological variation and provides a more realistic account on habitat use. This framework avoids the loss of ecological variation that results from oversimplification and retains valuable information on inter‐ and intraspecific variation. Although our study focuses on a single plant genus, the general message is clear: categorical thinking still persists in ecological and floristic research partly due to the limited awareness of its pitfalls. As a starting point for improving data quality, we encourage specimen collectors and authors of floristic treatments to document habitat conditions with greater precision and consistency.

## Author Contributions

GZ conceived the study. JT, MS and GZ collected the data. JT analysed and interpreted the data. JT wrote the manuscript draft with inputs from MS and GZ. All authors contributed critically to the drafts and gave final approval for publication.

## Supporting information


**Data S1.** Supporting Information.

**Table S1.** Original data of habitat preference of *Peperomi*a species of 419 sources. Given are 1) valid species names follow the International Plant Names Index, Tropicos and peperomia.net, 2) names used in the original publications, 3) the reference, and 4) the % preference for epiphytic (EV), lithophytic (LV) and terrestrial (TV) habitat, respectively. Full references can be found in Table S10.

**Table S2.** Mean substrate preference value of 1375 species of *Peperomia* for epiphytic (EV), lithophytic (LV) and terrestrial (TV) growth, including the number of sources for each species and their subgenus (if applicable).
**Fig. S3.** Workflow for data compilation and analysis. An annotated R script for creating the pivot table from the database, and to plot the triangle ordination is included as an upload into the supplementary materials (ELT analysis).

**Table S4.** Database showing data curation process detailed in the method section. Full references can be found in Table S10.

**Fig. S5.** Map of geographical regions as listed in Table 3 (Adapted from the world geographical scheme for recording plant distribution (Brummitt 2001)). Brummitt, R. K. 2001. World geographical scheme for recording plant distributions. International working group on taxonomic databases for plants sciences. Website: https://web.archive.org/web/20160125135239/http:/www.nhm.ac.uk/hosted_sites/tdwg/TDWG_geo2.pdf [accessed 27 March 2025].
**Fig. S6.** Percentage of species showing the tendency to be found in the respective habitats (EV/TV/LV >50%). Species geographical distribution was obtained from Plants of the World Online, based on the regional scheme. Species occurring in each regional area have an average ETL value extracted from Table S2, and the percentage of species (within each region) with EV/TV/LV >50% was further calculated (see Table S3 below). This map must be interpreted cautiously because the ETL value from Table S2 is an average value calculated for each species across the entire geographical range, and not collated for individual regions. Hence, it do not reflect intraspecific variation in habitat use due to differences in geographical locations – which can probably be visualized at a much finer scale. Furthermore, the percentage of species that are primarily found on each habitat must be interpreted together with the total number of species found in that region. For example, it seems that a majority of species in the Arabian Peninsula are lithophytic. However, there are only three species in that region, two of which have the tendency to be found in lithophytic habitats. Nonetheless, the map is useful in showing that a large percentage of species in the Andes are indeed found terrestrially.
**Table S7.** Percentages of species that show a tendency towards a certain habitat (EV, LV and TV >50%) for each regional area.
**Fig. S8.** Distribution of *Peperomia* species in five subgenera, in their epiphyte–lithophyte–terrestrial space. Symbol size varies with the number of species that share the same values. The grey lines separate the triangle into four smaller ones. The central triangle is occupied by generalists, and the three other triangles indicate the tendency of a species to be found in an epiphytic, lithophytic or terrestrial habitat. The full data set is given in Tables S1 and S2.
**Table S9.** Percentages of species that are either specialists (EV, LV, and TV >95%) or show a tendency towards a certain habitat (EV, LV and TV >50%) for the 14 subgenera of *Peperomia*.

**Table S10.** References list.

## Data Availability

All data are uploaded in the Supporting Information – S1 and S5.
