# Supplementary material for: “On a tree”, “terrestrial”, or “on the rocks”? Habit diversity in the megadiverse genus Peperomia
Source: Plant Biol (Stuttg). 2026 May 13;28(5):1373–84. doi: 10.1111/plb.70214 (PMC13358651; doi:10.1111/plb.70214)
Supplement: Supplementary file 1 — Data S1. Supporting Information. [file PLB-28-1373-s001.docx]

ELT analysis

Jessica Y. L. Tay

2025-12-11

## Code for ETL analysis

## Load necessary libraries for the analysis

library(tidyr), library(dplyr), library(plotrix)

## Choose your own directory here and set it as the working directory

setwd() ## check if you set it correctly getwd()

## Read your data

## Example data frame

## Test.csv should include the columns “Species”, “EV”, “LV”, “TV

df <- read.csv(“Test.csv”, header = TRUE, sep = “;”, stringsAsFactors=FALSE, dec = “,”)

## Check the data if they are in the correct format

summary(df)

df$EV<-as.numeric(df$EV)

df$LV<-as.numeric(df$LV)

df$TV<-as.numeric(df$TV)

## Pivot table: group by species

pivot_table <- df %>%

group_by(Species) %>% # Group by the 'Species' column

summarise(

Avg_EV = round(mean(EV), 2), # Calculate average of Column1

Avg_LV = round(mean(LV), 2), # Calculate average of Column2

Avg_TV = round(mean(TV), 2) # Calculate average of Column3

) %>%

arrange(Species)

print(pivot_table)

# Sort the dataframe by Column1 (EV), then Column2 (LV), then Column3 (TV) in ascending order

sorted_pivot_table <- pivot_table %>%

arrange(Avg_EV, Avg_LV, Avg_TV)

## Remove species names

sorted_pivot_table <- data.frame(sorted_pivot_table$Avg_EV,sorted_pivot_table$Avg_LV, sorted_pivot_table$Avg_TV)

sorted_pivot_table <- sorted_pivot_table %>%

rename(

Avg_EV = sorted_pivot_table.Avg_EV,

Avg_LV = sorted_pivot_table.Avg_LV,

Avg_TV = sorted_pivot_table.Avg_TV

)

# Create a unique identifier for each row by concatenating Column1, Column2, and Column3

sorted_pivot_table$UniqueRow <- apply(sorted_pivot_table, 1, function(x) paste(x, collapse = “_“))

# Get the frequency of each unique combination

row_frequencies <- table(sorted_pivot_table$UniqueRow)

# Convert the result to a data frame for easier visualization

frequency_sorted <- as.data.frame(row_frequencies) colnames(frequency_sorted) <- c(“UniqueRow”, “Frequency”)

# Split the ‘UniqueRow’ into 3 separate columns (E, L, T)

frequency_sorted_split <- separate(frequency_sorted, UniqueRow, into = c(“E”, “L”, “T”), sep = “_“)

Sort frequency from lowest to highest

sorted_final <- frequency_sorted_split %>% arrange(Frequency)

Export the sorted_final file into two files. 1) EV/LV/TV column as ELT.csv, and Frequency column as Freq.csv file to use in the triangle ordination

Exported files will go directly into your working directory that you set at the beginning

write.table(sorted_final[,1:3], “ELT.csv”, row.names = F, col.names=c(“E”,“L”,“T”)) write.table(sorted_final[,4], “Freq.csv”, row.names = F,col.names=c(“Freq”))

Plot triangular ordinations

Prepare the margins of the plot

par(mfcol=c(1,1), mar=c(1,1,1,1))

elastEpi <- read.csv(“ELT.csv”, header = TRUE, sep = ” “, stringsAsFactors=FALSE, dec =”.”) ## take note, if there is an error message, check the separator and the decimal

elastEpi <- data.frame(elastEpi$E,elastEpi$T, elastEpi$L)

attach (elastEpi) summary(elastEpi)

ElastEpi <- elastEpi/100

Sizes <- read.csv(“Freq.csv”)

as.numeric (SizeS[,1]) -> SizeS; SizeS

## triax.plot(x=ElastEpi,at = seq(0.1, 0.9, by = 0.1), cex=(SizeS/2.5)^(1/2.7), cex.ticks=1,

## pch=16, col.symbols="black", axis.labels=c("epiphytic",

## "terrestrial",

## "lithophytic"),

## mar=c(4,1,0,3), cex.axis=1.75,tick.labels=list(b=seq(10, 90, by=10),

## r=seq(10, 90, by=10),

## l=seq(10, 90, by=10)))

## triax.abline(b=0.5,r=0.5,l=0.5,col="grey",lty=1,cc.axes=FALSE)

Adding legend

Legend <- data.frame(Freq = c(1, 10, 50)) ## change the Frequency class according to your data

as.numeric (Legend[,1]) -> Legend; Legend

legend(1.1,0.8, legend= c("1","10","50"),

pch = 16,

pt.cex = (Legend/2.5)^(1/2.7),

cex = 1.5,

y.intersp = c(1,1,1.3,1.3,1.3),

x.intersp = 4,

title = "# species",

bty = "n")

mtext(expression(paste(italic('Genus or species name here'), " ", "n = =??")), 1,line = 1, font=4, padj=-18,cex=2)
