# Supplementary material for: “On a tree”, “terrestrial”, or “on the rocks”? Habit diversity in the megadiverse genus Peperomia
Source: Plant Biol (Stuttg). 2026 May 13;28(5):1373–84. doi: 10.1111/plb.70214 (PMC13358651; doi:10.1111/plb.70214)
Supplement: Supplementary file 2 — Table S1. Original data of habitat preference of Peperomia species of 419 sources. Given are 1) valid species names follow the International Plant Names Index, Tropicos and peperomia.net, 2) names used in the original publications, 3) the reference, and 4) the % preference for epiphytic (EV), lithophytic (LV) and terrestrial (TV) habitat, respectively. Full references can be found in Table S10. [file PLB-28-1373-s005.pdf]

Table S1: Original data of habitat preferences of Peperomia species from 419 sources. Given are 1) valid species name follow the International Plant Names Index, Tropicos and peperomia.net, 2) names used in the original publications, 3) the reference, and 4) the % preference for epiphytic (EV), lithophytic (LV) and terrestrial (TV) habitat, respectively. Full references can be found in Table S10.

Valid species name	Name in publication	Reference	EV(%)	LV(%)	TV(%)
Peperomia abbreviatipes	*Peperomia abbreviatipes*	255	100	0	0
Peperomia abdita	*Peperomia abdita*	198	0	100	0
Peperomia abnormis	*Peperomia abnormis*	158	100	0	0
Peperomia abnormis	*Peperomia abnormis*	374	62.5	0	37.5
Peperomia abnormis	*Peperomia abnormis*	373	100	0	0
Peperomia abnormis	*Peperomia abnormis*	255	100	0	0
Peperomia abnormis	*Peperomia abnormis*	191	100	0	0
Peperomia abscondita	*Peperomia abscondita*	198	0	100	0
Peperomia abscondita	*Peperomia abscondita*	255	0	50	50
Peperomia abyssinica	*Peperomia abyssinica*	2	100	0	0
Peperomia abyssinica	*Peperomia abyssinica*	114	50	0	50
Peperomia abyssinica	*Peperomia abyssinica*	414	0	100	0
Peperomia abyssinica	*Peperomia abyssinica*	138	50	50	0
Peperomia abyssinica	*Peperomia abyssinica*	144	50	50	0
Peperomia abyssinica	*Peperomia abyssinica*	123	50	50	0
Peperomia abyssinica	*Peperomia abyssinica*	1	30	0	70
Peperomia abyssinica	*Peperomia abyssinica*	384	70	15	15
Peperomia abyssinica	*Peperomia abyssinica*	91	90	10	0
Peperomia abyssinica	*Peperomia abyssinica*	16	50	0	50
Peperomia abyssinica	*Peperomia abyssinica*	61	50	50	0
Peperomia abyssinica	*Peperomia abyssinica*	255	50	0	50
Peperomia abyssinica	*Peperomia abyssinica*	30	0	100	0
Peperomia abyssinica	*Peperomia rungwensis*	122	0	0	100
Peperomia abyssinica	*Peperomia stuhlmannii*	122	50	0	50
Peperomia abyssinica	*Peperomia goetzeana*	91	50	50	0
Peperomia abyssinica	*Peperomia goetzeana*	30	100	0	0
Peperomia abyssinica	*Peperomia goetzeana*	88	15	85	0
Peperomia abyssinica	*Peperomia abyssinica*	88	50	50	0
Peperomia acaulis	*Peperomia acaulis*	198	0	0	100
Peperomia acaulis	*Peperomia acaulis*	294	0	0	100
Peperomia aceramarcana	*Peperomia aceramarcana*	255	100	0	0
Peperomia aceramarcana	*Peperomia aceramarcana*	290	0	100	0
Peperomia aceroana	*Peperomia aceroana*	375	91.6	0	8.4
Peperomia aceroana	*Peperomia aceroana*	412	100	0	0
Peperomia aceroana	*Peperomia aceroana*	255	100	0	0
Peperomia acreana	*Peperomia acreana*	51	0	0	100
Peperomia acreana	*Peperomia acreana*	82	100	0	0
Peperomia acreana	*Peperomia acreana*	42	100	0	0
Peperomia acreana	*Peperomia acreana*	255	100	0	0
Peperomia acuminata	*Peperomia acuminata*	51	50	0	50
Peperomia acuminata	*Peperomia acuminata*	43	0	0	100
Peperomia acuminata	*Peperomia acuminata*	57	0	0	100
Peperomia acuminata	*Peperomia acuminata*	110	50	50	0
Peperomia acuminata	*Peperomia acuminata*	150	0	0	100
Peperomia acuminata	*Peperomia acuminata*	213	50	0	50
Peperomia acuminata	*Peperomia acuminata*	219	50	0	50
Peperomia acuminata	*Peperomia acuminata*	374	26.7	0	73.3
Peperomia acuminata	*Peperomia acuminata*	29	0	0	100
Peperomia acuminata	*Peperomia huacachiana*	29	0	0	100
Peperomia acuminata	*Peperomia acuminata*	294	33.3	33.4	33.3
Peperomia acuminata	*Peperomia acuminata*	214	0	0	100
Peperomia acuminata	*Peperomia acuminata*	37	50	0	50
Peperomia acuminata	*Peperomia acuminata*	216	0	100	0
Peperomia acuminata	*Peperomia acuminata*	178	0	0	100
Peperomia acuminata	*Peperomia basellifolia*	112	0	0	100
Peperomia acuminata	*Peperomia pyrifolia*	386	50	50	0
Peperomia acuminata	*Peperomia acuminata*	90	15	0	85
Peperomia acuminata	*Peperomia cacuminicola*				
Peperomia acuminata	*Peperomia ruiziana*				
Peperomia adamsonia	*Peperomia adamsonia*	388	50	50	0
Peperomia adamsonia	*Peperomia adamsonia*	115	50	50	0
Peperomia adamsonia	*Peperomia adamsonia*	255	50	50	0
Peperomia adenocarpa	*Peperomia adenocarpa*	310	0	0	100
Peperomia adscendens	*Peperomia adscendens*	19	100	0	0
Peperomia adscendens	*Peperomia adscendens*	43	50	0	50
Peperomia adscendens	*Peperomia adscendens*	150	90	0	10

Peperomia adscendens	*Peperomia adscendens*	219	50	0	50
Peperomia adscendens	*Peperomia adscendens*	368	95	0	5
Peperomia adscendens	*Peperomia adscendens*	29	100	0	0
Peperomia adscendens	*Peperomia adscendens*	373	100	0	0
Peperomia adscendens	*Peperomia queserana*	366	100	0	0
Peperomia adscendens	*Peperomia sarcodes*	5	100	0	0
Peperomia adscendens	*Peperomia casitana*	393	100	0	0
Peperomia adscendens	*Peperomia adscendens*	90	85	0	15
Peperomia adscendens	*Peperomia glabra*				
Peperomia adsurgens	*Peperomia adsurgens*	51	50	0	50
Peperomia adsurgens	*Peperomia adsurgens*	82	50	0	50
Peperomia aerea	*Peperomia aerea*	255	100	0	0
Peperomia aerea	*Peperomia aerea*	178	0	0	100
Peperomia aggregata	*Peperomia aggregata*	51	0	100	0
Peperomia aggregata	*Peperomia aggregata*	50	0	95	5
Peperomia aggregata	*Peperomia aggregata*	82	0	100	0
Peperomia aguabonitensis	*Peperomia aguabonitensis*	373	0	0	100
Peperomia aguaditana	*Peperomia aguaditana*	373	0	0	100
Peperomia agusanensis	*Peperomia agusanensis*				
Peperomia ainana	*Peperomia ainana*	178	0	0	100
Peperomia alata	*Peperomia alata*	18	100	0	0
Peperomia alata	*Peperomia alata*	51	33	34	33
Peperomia alata	*Peperomia alata*	31	100	0	0
Peperomia alata	*Peperomia alata*	80	100	0	0
Peperomia alata	*Peperomia alata*	114	100	0	0
Peperomia alata	*Peperomia alata*	150	34	33	33
Peperomia alata	*Peperomia alata*	219	100	0	0
Peperomia alata	*Peperomia alata*	374	60	0	40
Peperomia alata	*Peperomia alata*	83	34	33	33
Peperomia alata	*Peperomia alata*	29	100	0	0
Peperomia alata	*Peperomia alata*	328	0	100	0
Peperomia alata	*Peperomia alata*	373	30	30	40
Peperomia alata	*Peperomia alata*	273	50	50	0
Peperomia alata	*Peperomia alata*	220	50	50	0
Peperomia alata	*Peperomia alata*	294	33.3	33.3	33.4
Peperomia alata	*Peperomia alata*	221	0	50	50
Peperomia alata	*Peperomia alata*	214	100	0	0
Peperomia alata	*Peperomia alata*	85	50	50	0
Peperomia alata	*Peperomia alata*	255	50	50	0
Peperomia alata	*Peperomia alata*	156	0	100	0
Peperomia alata	*Peperomia alata*	178	0	100	0
Peperomia alata	*Peperomia alata*	37	100	0	0
Peperomia alata	*Peperomia alata*	32	100	0	0
Peperomia alata	*Peperomia alata*	112	0	100	0
Peperomia alata	*Peperomia alata*	191	50	0	50
Peperomia alata	*Peperomia microreticulata*	337	100	0	0
Peperomia alata	*Peperomia nilssonii*	336	0	0	100
Peperomia alata	*Peperomia alata*	128	50	50	0
Peperomia alata	*Peperomia alata*	90	33.3	33.3	33.4
Peperomia alata	*Peperomia acuminatissima*				
Peperomia alata	*Peperomia laevis*				
Peperomia alatiscapa	*Peperomia alatiscapa*	29	100	0	0
Peperomia alatiscapa	*Peperomia alatiscapa*	255	100	0	0
Peperomia alatiscapa	*Peperomia alatiscapa*	178	100	0	0
Peperomia albertiae	*Peperomia albertiae*	363	50	50	0
Peperomia albertiana	*Peperomia albertiana*	198	100	0	0
Peperomia albertiana	*Peperomia albertiana*	307	100	0	0
Peperomia albertiana	*Peperomia albertiana*	255	100	0	0
Peperomia albert-smithii	*Peperomia albert-smithii*	150	0	0	100
Peperomia albert-smithii	*Peperomia albert-smithii*	374	0	0	100
Peperomia albert-smithii	*Peperomia albert-smithii*	373	0	0	100
Peperomia albidiflora	*Peperomia albidiflora*	373	100	0	0
Peperomia albidiflora	*Peperomia albidiflora*	255	100	0	0
Peperomia albolineata	*Peperomia albolineata*				
Peperomia albonervosa	*Peperomia albonervosa*	198	0	0	100
Peperomia albonervosa	*Peperomia albonervosa*	204	0	0	100
Peperomia albopilosa	*Peperomia albopilosa*	51	0	100	0
Peperomia albopilosa	*Peperomia albopilosa*	82	0	100	0
Peperomia albopilosa	*Peperomia albopilosa*	223	0	100	0
Peperomia albovittata	*Peperomia albovittata*	373	0	0	100
Peperomia aldrinii	*Peperomia aldrinii*	255	100	0	0
Peperomia alegrensis	*Peperomia alegrensis*				
Peperomia alibacophylla	*Peperomia alibacophylla*	368	85	0	15
Peperomia alibacophylla	*Peperomia alibacophylla*	373	0	15	85
Peperomia alismifolia	*Peperomia alismifolia*	29	0	0	100
Peperomia alismifolia	*Peperomia alismifolia*	178	0	0	100
Peperomia alpina	*Peperomia alpina*	43	50	0	50
Peperomia alpina	*Peperomia alpina*	57	0	0	100
Peperomia alpina	*Peperomia alpina*	110	50	0	50
Peperomia alpina	*Peperomia alpina*	150	50	0	50

Peperomia alpina	*Peperomia alpina*	219	50	0	50
Peperomia alpina	*Peperomia alpina*	214	0	0	100
Peperomia alpina	*Peperomia alpina*	37	50	0	50
Peperomia alpina	*Peperomia alpina*	112	50	0	50
Peperomia alpina	*Peperomia longirostrata*	373	0	0	100
Peperomia alpina	*Peperomia machaerodonta*	163	100	0	0
Peperomia alpina	*Peperomia longemucronata*				
Peperomia alpina	*Peperomia pachyphlebia*				
Peperomia alpina	*Peperomia alpina*	90	0	0	100
Peperomia alternifolia	*Peperomia alternifolia*	387	50	50	0
Peperomia alternifolia	*Peperomia alternifolia*	255	100	0	0
Peperomia alwynii	*Peperomia alwynii*	374	0	0	100
Peperomia alwynii	*Peperomia alwynii*	41	15	0	85
Peperomia ambiguifolia	*Peperomia ambiguifolia*	373	0	0	100
Peperomia amnicola	*Peperomia amnicola*	198	100	0	0
Peperomia amphitricha	*Peperomia amphitricha*	43	50	0	50
Peperomia amphitricha	*Peperomia amphitricha*	57	0	0	100
Peperomia amphitricha	*Peperomia amphitricha*	219	50	0	50
Peperomia amphitricha	*Peperomia amphitricha*	37	50	0	50
Peperomia ampla	*Peperomia ampla*	204	0	0	100
Peperomia amplexicaulis	*Peperomia amplexicaulis*				
Peperomia amplexifolia	*Peperomia amplexifolia*	112	0	100	0
Peperomia andicola	*Peperomia andicola*	29	100	0	0
Peperomia andicola	*Peperomia andicola*	255	100	0	0
Peperomia andicola	*Peperomia andicola*	191	100	0	0
Peperomia andina	*Peperomia andina*	198	0	50	50
Peperomia andrei	*Peperomia andrei*	373	0	50	50
Peperomia angularis	*Peperomia angularis*	368	100	0	0
Peperomia angularis	*Peperomia angularis*	29	100	0	0
Peperomia angularis	*Peperomia angulariopsis*	373	0	0	100
Peperomia angularis	*Peperomia angularis*	373	0	0	100
Peperomia angularis	*Peperomia angularis*	255	33.3	33.3	33.4
Peperomia angularis	*Peperomia angularis*	37	85	0	15
Peperomia angularis	*Peperomia bethaniana*	373	0	0	100
Peperomia angularis	*Peperomia stenophyllopsis*	322	100	0	0
Peperomia angularis	*Peperomia aristeodora*				
Peperomia angustata	*Peperomia angustata*	51	100	0	0
Peperomia angustata	*Peperomia angustata*	31	100	0	0
Peperomia angustata	*Peperomia angustata*	43	100	0	0
Peperomia angustata	*Peperomia angustata*	57	0	0	100
Peperomia angustata	*Peperomia angustata*	110	90	10	0
Peperomia angustata	*Peperomia angustata*	150	50	50	0
Peperomia angustata	*Peperomia angustata*	154	100	0	0
Peperomia angustata	*Peperomia angustata*	83	33.3	33.3	33.4
Peperomia angustata	*Peperomia angustata*	29	100	0	0
Peperomia angustata	*Peperomia angustata*	373	50	0	50
Peperomia angustata	*Peperomia angustata*	381	100	0	0
Peperomia angustata	*Peperomia angustata*	86	50	50	0
Peperomia angustata	*Peperomia angustata*	335	100	0	0
Peperomia angustata	*Peperomia angustata*	130	100	0	0
Peperomia angustata	*Peperomia angustata*	293	85	15	0
Peperomia angustata	*Peperomia angustata*	255	50	50	0
Peperomia angustata	*Peperomia angustata*	37	50	50	0
Peperomia angustata	*Peperomia angustata*	276	100	0	0
Peperomia angustata	*Peperomia lundellii*	176	100	0	0
Peperomia angustata	*Peperomia rhodophlebia*	405	100	0	0
Peperomia angustata	*Peperomia sarcocarpa*	392	0	100	0
Peperomia angustata	*Peperomia viridispica*	373	33.3	33.3	33.4
Peperomia angustata	*Peperomia wagneri*	408	100	0	0
Peperomia angustata	*Peperomia crassiuscula*	328	50	50	0
Peperomia angustata	*Peperomia victoriana*	373	100	0	0
Peperomia angustata	*Peperomia victoriana*	276	100	0	0
Peperomia angustata	*Peperomia longispicata*				
Peperomia angustata	*Peperomia friabilis*				
Peperomia anisophylla	*Peperomia anisophylla*	29	0	0	100
Peperomia ankaranensis	*Peperomia ankaranensis*	201	0	0	100
Peperomia antioquiensis	*Peperomia antioquiensis*	198	0	100	0
Peperomia antoniana	*Peperomia antoniana*	29	0	0	100
Peperomia antoniana	*Peperomia antoniana*	178	0	0	100
Peperomia apiahyensis	*Peperomia apiahyensis*	51	0	0	100
Peperomia apiahyensis	*Peperomia apiahyensis*	82	0	0	100
Peperomia apodophylla	*Peperomia apodophylla*	373	0	0	100
Peperomia apodophylla	*Peperomia campii*	414	0	0	100
Peperomia apurimacana	*Peperomia apurimacana*	29	100	0	0
Peperomia apurimacana	*Peperomia apurimacana*	255	100	0	0
Peperomia apurimacana	*Peperomia apurimacana*	178	100	0	0
Peperomia arboricola	*Peperomia arboricola*	43	100	0	0
Peperomia arboricola	*Peperomia arboricola*	110	90	10	0
Peperomia arboricola	*Peperomia arboricola*	255	100	0	0
Peperomia arborigaudens	*Peperomia arborigaudens*	29	100	0	0

Peperomia arborigaudens	*Peperomia arborigaudens*	255	100	0	0
Peperomia arborigaudens	*Peperomia arborigaudens*	178	100	0	0
Peperomia arboriseda	*Peperomia arboriseda*	29	100	0	0
Peperomia arboriseda	*Peperomia arboriseda*	255	100	0	0
Peperomia arboriseda	*Peperomia arboriseda*	178	100	0	0
Peperomia arboriseda	*Peperomia arboriseda*	29	100	0	0
Peperomia arctebaccata	*Peperomia arctebaccata*	29	0	0	100
Peperomia arctebaccata	*Peperomia arctebaccata*	255	100	0	0
Peperomia arcuatisicpa	*Peperomia arcuatisicpa*	29	0	0	100
Peperomia arenillasensis	*Peperomia arenillasensis*	373	100	0	0
Peperomia arenillasensis	*Peperomia arenillasensis*	255	100	0	0
Peperomia areolata	*Peperomia areolata*	29	0	0	100
Peperomia areolata	*Peperomia areolata*	288	0	100	0
Peperomia argenteobracteata	*Peperomia argenteobracteata*	373	0	0	100
Peperomia argenteobracteata	*Peperomia argenteobracteata*	255	100	0	0
Peperomia argyraea	*Peperomia argyraea*	255	50	0	50
Peperomia argyreia	*Peperomia argyreia*	51	50	0	50
Peperomia argyreia	*Peperomia argyreia*	43	50	0	50
Peperomia argyreia	*Peperomia argyreia*	150	100	0	0
Peperomia argyreia	*Peperomia argyreia*	412	50	0	50
Peperomia argyreia	*Peperomia argyreia*	373	50	0	50
Peperomia argyreia	*Peperomia sandersii*				
Peperomia argyroneura	*Peperomia palauensis*	118	0	100	0
Peperomia arifolia	*Peperomia arifolia*	51	33	34	33
Peperomia arifolia	*Peperomia arifolia*	43	0	0	100
Peperomia arifolia	*Peperomia arifolia*	275	0	0	100
Peperomia arifolia	*Peperomia arifolia*	220	0	50	50
Peperomia arifolia	*Peperomia arifolia*	113	0	100	0
Peperomia arifolia	*Peperomia arifolia*	48	0	100	0
Peperomia arifolia	*Peperomia arifolia*	135	0	100	0
Peperomia arifolia	*Peperomia arifolia*	84	0	50	50
Peperomia arifolia	*Peperomia arifolia*	42	100	0	0
Peperomia arifolia	*Peperomia arifolia*	85	0	50	50
Peperomia arifolia	*Peperomia arifolia*	418	42.5	15	42.5
Peperomia arifolia	*Peperomia arifolia*	156	0	100	0
Peperomia arifolia	*Peperomia arifolia*	252	33.3	33.3	33.4
Peperomia arifolia	*Peperomia misionense*	235	0	100	0
Peperomia aristeguietae	*Peperomia aristeguietae*	150	0	0	100
Peperomia aristeguietae	*Peperomia aristeguietae*	255	100	0	0
Peperomia armadana	*Peperomia armadana*		100	0	0
Peperomia armondii	*Peperomia armondii*	51	100	0	0
Peperomia armondii	*Peperomia armondii*	80	0	100	0
Peperomia armondii	*Peperomia armondii*	160	50	50	0
Peperomia armondii	*Peperomia armondii*	85	100	0	0
Peperomia armondii	*Peperomia armondii*	417	50	50	0
Peperomia armondii	*Peperomia armondii*	418	100	0	0
Peperomia armondii	*Peperomia armondii*	255	50	50	0
Peperomia armondii	*Peperomia armondii*	252	100	0	0
Peperomia armstrongii	*Peperomia armstrongii*	418	0	0	100
Peperomia armstrongii	*Peperomia armstrongii*	255	0	0	100
Peperomia aroensis	*Peperomia aroensis*	150	100	0	0
Peperomia aroensis	*Peperomia aroensis*	255	100	0	0
Peperomia artatiflora	*Peperomia artatiflora*	29	0	0	100
Peperomia arthurii	*Peperomia arthurii*	368	100	0	0
Peperomia arthurii	*Peperomia arthurii*	255	100	0	0
Peperomia asarifolia	*Peperomia asarifolia*	43	0	50	50
Peperomia asarifolia	*Peperomia pseudodependens*	57	0	0	100
Peperomia asarifolia	*Peperomia asarifolia*	110	10	45	45
Peperomia asarifolia	*Peperomia asarifolia*	150	0	50	50
Peperomia asarifolia	*Peperomia asarifolia*	328	0	70	30
Peperomia asarifolia	*Peperomia asarifolia*	293	5	47.5	47.5
Peperomia asarifolia	*Peperomia asarifolia*	255	100	0	0
Peperomia asarifolia	*Peperomia brevipeduncula*	57	0	0	100
Peperomia asarifolia	*Peperomia heydei*				
Peperomia asarifolioides	*Peperomia asarifolioides*	43	0	50	50
Peperomia asarifolioides	*Peperomia asarifolioides*	127	0	50	50
Peperomia asarifolioides	*Peperomia asarifolioides*	255	0	100	0
Peperomia asperula	*Peperomia asperula*	265	0	0	100
Peperomia asperula	*Peperomia asperula*	361	0	100	0
Peperomia asplundii	*Peperomia asplundii*	29	0	0	100
Peperomia asplundii	*Peperomia asplundii*	255	100	0	0
Peperomia asterophylla	*Peperomia asterophylla*	29	100	0	0
Peperomia asterophylla	*Peperomia asterophylla*	255	100	0	0
Peperomia attenuata	*Peperomia attenuata*	198	50	0	50
Peperomia attenuata	*Peperomia attenuata*	307	50	0	50
Peperomia attenuata	*Peperomia attenuata*	255	50	0	50
Peperomia augescens	*Peperomia augescens*	51	33	34	33
Peperomia augescens	*Peperomia augescens*	82	34	33	33
Peperomia augescens	*Peperomia augescens*	255	50	0	50
Peperomia augescens	*Peperomia augescens*	220	0	50	50

Peperomia augescens	*Peperomia augescens*	221	50	0	50
Peperomia aurorana	*Peperomia aurorana*	43	0	0	100
Peperomia aurorana	*Peperomia aurorana*	255	0	0	100
Peperomia austin-smithii	*Peperomia austin-smithii*	43	50	0	50
Peperomia austin-smithii	*Peperomia austin-smithii*	255	50	0	50
Peperomia australana	*Peperomia australana*	198	50	0	50
Peperomia australana	*Peperomia australana*	115	50	50	0
Peperomia australana	*Peperomia raivavaeana*	314	100	0	0
Peperomia australana	*Peperomia rurutana*	316	0	100	0
Peperomia ayacuchoana	*Peperomia ayacuchoana*	292	0	0	100
Peperomia bajana	*Peperomia bajana*	373	0	0	100
Peperomia balansana	*Peperomia balansana*	51	0	50	50
Peperomia balansana	*Peperomia balansana*	375	14.2	0	85.8
Peperomia balansana	*Peperomia balansana*	418	0	0	100
Peperomia balansana	*Peperomia balansana*	252	33.3	33.3	33.4
Peperomia balfourii	*Peperomia balfourii*	255	100	0	0
Peperomia bamleri	*Peperomia bamleri*	44	100	0	0
Peperomia bamleri	*Peperomia bamleri*	255	100	0	0
Peperomia bangii	*Peperomia bangii*	29	0	0	100
Peperomia bangroana	*Peperomia bangroana*	1	100	0	0
Peperomia bangroana	*Peperomia bangroana*	255	50	0	50
Peperomia barahonana	*Peperomia barahonana*	16	50	50	0
Peperomia barbarana	*Peperomia barbarana*	51	0	0	100
Peperomia barbarana	*Peperomia barbarana*	53	100	0	0
Peperomia barbarana	*Peperomia barbarana*	275	0	0	100
Peperomia barbarana	*Peperomia barbarana*	375	20	10	70
Peperomia barbarana	*Peperomia barbarana*	418	0	0	100
Peperomia barbaranoides	*Peperomia barbaranoides*	255	100	0	0
Peperomia barbata	*Peperomia barbata*	112	0	100	0
Peperomia barbulata	*Peperomia barbulata*	43	50	0	50
Peperomia barbulata	*Peperomia barbulata*	255	50	0	50
Peperomia baronii	*Peperomia baronii*	255	100	0	0
Peperomia barryana	*Peperomia barryana*	43	0	0	100
Peperomia basiradicans	*Peperomia basiradicans*	204	0	100	0
Peperomia basiradicans	*Peperomia basiradicans*	293	0	0	100
Peperomia bavina	*Peperomia bavina*	340	0	50	50
Peperomia bavina	*Peperomia bavina*	255	0	50	50
Peperomia beccarii	*Peperomia beccarii*				
Peperomia beckeri	*Peperomia beckeri*				
Peperomia bella	*Peperomia bella*	373	100	0	0
Peperomia bellatula	*Peperomia bellatula*	373	0	50	50
Peperomia bellendenkerensis	*Peperomia bellendenkerensis*	26	15	85	0
Peperomia berlandieri	*Peperomia berlandieri*	110	50	50	0
Peperomia berlandieri	*Peperomia berlandieri*	255	100	0	0
Peperomia berlandieri	*Peperomia berlandieri*	293	50	50	0
Peperomia berlandieri	*Peperomia berlandieri*	278	0	0	100
Peperomia berlandieri	*Peperomia papantlacensis*	110	100	0	0
Peperomia bermudezana	*Peperomia bermudezana*	29	100	0	0
Peperomia bermudezana	*Peperomia bermudezana*	255	100	0	0
Peperomia bermudezana	*Peperomia bermudezana*	178	100	0	0
Peperomia bernhardiana	*Peperomia bernhardiana*	51	0	50	50
Peperomia bernhardiana	*Peperomia bernhardiana*	82	0	0	100
Peperomia bernhardiana	*Peperomia bernhardiana*	255	0	100	0
Peperomia bernieriana	*Peperomia bernieriana*	22	100	0	0
Peperomia bernoullii	*Peperomia bernoullii*	43	0	25	75
Peperomia bernoullii	*Peperomia bernoullii*	328	0	0	100
Peperomia bernoullii	*Peperomia uphofii*	327	0	100	0
Peperomia bernoullii	*Peperomia izalcoana*				
Peperomia bernoullii	*Peperomia violifolia*				
Peperomia berryi	*Peperomia berryi*	19	100	0	0
Peperomia berryi	*Peperomia berryi*	150	0	50	50
Peperomia berteroana	*Peperomia berteroana*	412	0	0	100
Peperomia berteroana	*Peperomia berteroana*	306	0	60	40
Peperomia berteroana	*Peperomia tristanensis*				
Peperomia biamenta	*Peperomia biamenta*	255	100	0	0
Peperomia bicolor	*Peperomia bicolor*	29	100	0	0
Peperomia bicolor	*Peperomia bicolor*	373	0	0	100
Peperomia bicolor	*Peperomia bicolor*	121	0	100	0
Peperomia biformis	*Peperomia biformis*	266	0	0	100
Peperomia bilobulata	*Peperomia bilobulata*	373	0	0	100
Peperomia bismarckiana	*Peperomia bismarckiana*	255	100	0	0
Peperomia biuncialis	*Peperomia biuncialis*				
Peperomia blackii	*Peperomia blackii*	51	100	0	0
Peperomia blackii	*Peperomia blackii*	82	100	0	0
Peperomia blackii	*Peperomia blackii*	255	100	0	0
Peperomia blanda	*Peperomia blanda*	19	100	0	0
Peperomia blanda	*Peperomia blanda*	51	33	34	33
Peperomia blanda	*Peperomia blanda*	43	0	0	100
Peperomia blanda	*Peperomia blanda*	53	100	0	0
Peperomia blanda	*Peperomia blanda*	80	0	100	0

Peperomia blanda	*Peperomia blanda*	110	50	0	50
Peperomia blanda	*Peperomia blanda*	114	5	0	95
Peperomia blanda	*Peperomia blanda*	150	33.3	33.3	33.4
Peperomia blanda	*Peperomia increscens*	184	5	0	95
Peperomia blanda	*Peperomia blanda*	374	33.4	0	66.6
Peperomia blanda	*Peperomia blanda*	27	0	50	50
Peperomia blanda	*Peperomia dindygulensis*	264	0	50	50
Peperomia blanda	*Peperomia blanda*	247	33.3	33.3	33.4
Peperomia blanda	*Peperomia blanda*	375	12.5	75	12.5
Peperomia blanda	*Peperomia blanda*	236	33.3	33.3	33.4
Peperomia blanda	*Peperomia blanda*	29	0	0	100
Peperomia blanda	*Peperomia blanda*	144	15	30	55
Peperomia blanda	*Peperomia blanda*	328	50	50	0
Peperomia blanda	*Peperomia blanda*	376	0	50	50
Peperomia blanda	*Peperomia blanda*	373	0	50	50
Peperomia blanda	*Peperomia blanda*	384	5	55	40
Peperomia blanda	*Peperomia blanda*	220	50	50	0
Peperomia blanda	*Peperomia blanda*	335	0	0	100
Peperomia blanda	*Peperomia blanda*	155	50	0	50
Peperomia blanda	*Peperomia blanda*	113	33.3	33.3	33.4
Peperomia blanda	*Peperomia blanda*	368	100	0	0
Peperomia blanda	*Peperomia blanda*	179	0	0	100
Peperomia blanda	*Peperomia blanda*	221	0	50	50
Peperomia blanda	*Peperomia blanda*	48	0	100	0
Peperomia blanda	*Peperomia blanda*	119	5	47.5	47.5
Peperomia blanda	*Peperomia blanda*	293	0	0	100
Peperomia blanda	*Peperomia blanda*	224	33.3	33.3	33.4
Peperomia blanda	*Peperomia blanda*	115	50	50	0
Peperomia blanda	*Peperomia blanda*	255	100	0	0
Peperomia blanda	*Peperomia blanda*	252	33.3	33.3	33.4
Peperomia blanda	*Peperomia porriginifera*	150	0	0	100
Peperomia blanda	*Peperomia porriginifera*	373	100	0	0
Peperomia blanda	*Peperomia blanda*	88	0	50	50
Peperomia blanda	*Peperomia tortugana*				
Peperomia blanda	*Peperomia ciliata*				
Peperomia blanda	*Peperomia constanzana*				
Peperomia blanda	*Peperomia ellipticifolia*				
Peperomia blanda	*Peperomia salvaje*				
Peperomia blephariphylla	*Peperomia blephariphylla*	57	100	0	0
Peperomia blephariphylla	*Peperomia blephariphylla*	373	15	0	85
Peperomia blephariphylla	*Peperomia blephariphylla*	255	100	0	0
Peperomia blepharipus	*Peperomia blepharipus*	29	100	0	0
Peperomia blepharipus	*Peperomia blepharipus*	255	100	0	0
Peperomia blepharipus	*Peperomia blepharipus*	178	100	0	0
Peperomia boekei	*Peperomia boekei*	29	0	0	100
Peperomia boivinii	*Peperomia boivinii*	255	100	0	0
Peperomia boliviensis	*Peperomia boliviensis*	186	100	0	0
Peperomia boninsimensis	*Peperomia boninsimensis*	255	50	50	0
Peperomia bopiana	*Peperomia bopiana*	255	100	0	0
Peperomia borbonensis	*Peperomia borbonensis*	255	100	0	0
Peperomia borbonensis	*Peperomia hircina*				
Peperomia borburatensis	*Peperomia borburatensis*	150	0	0	100
Peperomia botterii	*Peperomia botterii*	43	100	0	0
Peperomia botterii	*Peperomia botterii*	110	50	0	50
Peperomia botterii	*Peperomia botterii*	255	100	0	0
Peperomia bourneae	*Peperomia bourneae*	255	100	0	0
Peperomia brachypoda	*Peperomia brachypoda*	198	100	0	0
Peperomia brachypoda	*Peperomia brachypoda*	255	100	0	0
Peperomia brachytricha	*Peperomia brachytricha*				
Peperomia bracteata	*Peperomia bracteata*	43	0	50	50
Peperomia bracteata	*Peperomia bracteata*	255	50	0	50
Peperomia bracteata	*Peperomia bracteata*	293	5	47.5	47.5
Peperomia bracteata	*Peperomia campylotropa*	328	0	30	70
Peperomia bracteispica	*Peperomia bracteispica*	29	100	0	0
Peperomia bracteispica	*Peperomia bracteispica*	255	100	0	0
Peperomia bracteispica	*Peperomia bracteispica*	178	100	0	0
Peperomia bradei	*Peperomia bradei*	51	100	0	0
Peperomia bradei	*Peperomia bradei*	82	100	0	0
Peperomia bradei	*Peperomia bradei*	255	100	0	0
Peperomia brasiliensis	*Peperomia brasiliensis*	51	50	0	50
Peperomia brasiliensis	*Peperomia brasiliensis*	82	50	0	50
Peperomia brasiliensis	*Peperomia brasiliensis*	255	0	0	100
Peperomia brasiliensis	*Peperomia wedelii*	57	100	0	0
Peperomia brasiliensis	*Peperomia verhuellia*				
Peperomia breedlovei	*Peperomia breedlovei*	43	0	50	50
Peperomia breedlovei	*Peperomia breedlovei*	255	0	50	50
Peperomia brevihirtella	*Peperomia brevihirtella*	255	100	0	0
Peperomia breviramula	*Peperomia breviramula*	118	100	0	0
Peperomia breviramula	*Peperomia breviramula*	255	100	0	0
Peperomia brevispica	*Peperomia brevispica*				

Peperomia brittonii	*Peperomia brittonii*	29	0	0	100
Peperomia brouetiana	*Peperomia brouetiana*	255	100	0	0
Peperomia bryophila	*Peperomia bryophila*	255	100	0	0
Peperomia buchtienii	*Peperomia buchtienii*	255	100	0	0
Peperomia buchtienii	*Peperomia buchtienii*	347	0	0	100
Peperomia buxifolia	*Peperomia buxifolia*	373	0	0	100
Peperomia cacaophila	*Peperomia cacaophila*	89	100	0	0
Peperomia cacaophila	*Peperomia cacaophila*	374	100	0	0
Peperomia cacaophila	*Peperomia cacaophila*	251	50	50	0
Peperomia cacaophila	*Peperomia cacaophila*	373	50	0	50
Peperomia cacaophila	*Peperomia cacaophila*	255	50	50	0
Peperomia cachabiana	*Peperomia cachabiana*	43	100	0	0
Peperomia cachabiana	*Peperomia cachabiana*	374	75	0	25
Peperomia cachabiana	*Peperomia cachabiana*	373	0	0	100
Peperomia cachabiana	*Peperomia cachabiana*	255	100	0	0
Peperomia caducifolia	*Peperomia caducifolia*	29	0	0	100
Peperomia caducipilosa	*Peperomia caducipilosa*	395	100	0	0
Peperomia caespitosa	*Peperomia caespitosa*	374	92.8	0	7.2
Peperomia caespitosa	*Peperomia caespitosa*	373	0	0	100
Peperomia caespitosa	*Peperomia caespitosa*	255	100	0	0
Peperomia cainarachiana	*Peperomia cainarachiana*	374	100	0	0
Peperomia cainarachiana	*Peperomia cainarachiana*	29	0	0	100
Peperomia cainarachiana	*Peperomia tetrica*	255	100	0	0
Peperomia cainarachiana	*Peperomia cainarachiana*	191	50	0	50
Peperomia calcicola	*Peperomia calcicola*	51	100	0	0
Peperomia calcicola	*Peperomia calcicola*	82	0	100	0
Peperomia calcicola	*Peperomia calcicola*	187	0	100	0
Peperomia caledonica	*Peperomia caledonica*	255	100	0	0
Peperomia caliginigaudens	*Peperomia caliginigaudens*	150	0	50	50
Peperomia caliginigaudens	*Peperomia caliginigaudens*	373	0	0	100
Peperomia caliginigaudens	*Peperomia caliginigaudens*	255	0	50	50
Peperomia callana	*Peperomia callana*	247	0	50	50
Peperomia callejasii	*Peperomia callejasii*	43	0	0	100
Peperomia callitrichoides	*Peperomia callitrichoides*	137	100	0	0
Peperomia calophylla	*Peperomia calophylla*	82	0	0	100
Peperomia calophylla	*Peperomia calophylla*	51	0	0	100
Peperomia calvescens	*Peperomia calvescens*	29	0	0	100
Peperomia calvicaulis	*Peperomia calvicaulis*	43	50	50	0
Peperomia calvicaulis	*Peperomia calvicaulis*	219	100	0	0
Peperomia calvicaulis	*Peperomia calvicaulis*	255	50	50	0
Peperomia calvifolia	*Peperomia sessilifolia*	43	33.3	33.3	33.4
Peperomia calvifolia	*Peperomia sessilifolia*	255	33.3	33.3	33.4
Peperomia campana	*Peperomia campana*	43	100	0	0
Peperomia campana	*Peperomia campana*	255	100	0	0
Peperomia campinasana	*Peperomia campinasana*	51	50	50	0
Peperomia campinasana	*Peperomia campinasana*	82	50	50	0
Peperomia campinasana	*Peperomia campinasana*	255	100	0	0
Peperomia campinasana	*Peperomia campinasana*	113	100	0	0
Peperomia campinasana	*Peperomia campinasana*	48	50	50	0
Peperomia camposii	*Peperomia camposii*	198	0	0	100
Peperomia camptotricha	*Peperomia camptotricha*	110	50	50	0
Peperomia canalensis	*Peperomia canalensis*	295	0	100	0
Peperomia canaminana	*Peperomia canaminana*	255	100	0	0
Peperomia candelaber	*Peperomia candelaber*	43	100	0	0
Peperomia candelaber	*Peperomia candelaber*	255	100	0	0
Peperomia candelaber	*Peperomia candelaber*	37	50	0	50
Peperomia candida	*Peperomia candida*	217	50	50	0
Peperomia caniana	*Peperomia caniana*	29	0	0	100
Peperomia canlaonensis	*Peperomia canlaonensis*	238	100	0	0
Peperomia canlaonensis	*Peperomia canlaonensis*	255	100	0	0
Peperomia caperata	*Peperomia caperata*	255	0	0	100
Peperomia capitis-bovis	*Peperomia capitis-bovis*	255	100	0	0
Peperomia caraboboensis	*Peperomia caraboboensis*	150	0	0	100
Peperomia caraboboensis	*Peperomia caraboboensis*	90	0	0	100
Peperomia cardenasii	*Peperomia cardenasii*	51	100	0	0
Peperomia cardenasii	*Peperomia cardenasii*	42	100	0	0
Peperomia carnevalii	*Peperomia carnevalii*	150	0	0	100
Peperomia carnicaulis	*Peperomia carnicaulis*				
Peperomia carnifolia	*Peperomia carnifolia*	412	100	0	0
Peperomia carnifolia	*Peperomia carnifolia*	255	100	0	0
Peperomia carpapatana	*Peperomia carpapatana*	29	0	0	100
Peperomia carpinterana	*Peperomia carpinterana*	43	50	0	50
Peperomia carpinterana	*Peperomia carpinterana*	255	50	0	50
Peperomia carpinterana	*Peperomia carpinterana*	37	100	0	0
Peperomia casapiana	*Peperomia casapiana*	29	0	0	100
Peperomia casapiana	*Peperomia casapiana*	255	100	0	0
Peperomia casarettoi	*Peperomia casarettoi*	255	100	0	0
Peperomia casimirii	*Peperomia casimirii*				
Peperomia castelosensis	*Peperomia castelosensis*	51	100	0	0
Peperomia castelosensis	*Peperomia castelosensis*	82	100	0	0

Peperomia castelosensis	*Peperomia castelosensis*	255	100	0	0
Peperomia castilloi	*Peperomia castilloi*	157	100	0	0
Peperomia catesbaeifolia	*Peperomia arbuscula*	255	100	0	0
Peperomia catharinae	*Peperomia catharinae*	51	100	0	0
Peperomia catharinae	*Peperomia catharinae*	184	85	15	0
Peperomia catharinae	*Peperomia catharinae*	184	100	0	0
Peperomia catharinae	*Peperomia catharinae*	273	50	50	0
Peperomia catharinae	*Peperomia catharinae*	220	100	0	0
Peperomia catharinae	*Peperomia catharinae*	179	100	0	0
Peperomia catharinae	*Peperomia catharinae*	418	100	0	0
Peperomia catharinae	*Peperomia catharinae*	255	100	0	0
Peperomia catharinae	*Peperomia catharinae*	252	100	0	0
Peperomia catharinensis	*Peperomia catharinensis*	62	50	0	50
Peperomia caucana	*Peperomia deodorata*	29	100	0	0
Peperomia caucana	*Peperomia caucana*	373	0	0	100
Peperomia cavaleriei	*Peperomia cavaleriei*	340	100	0	0
Peperomia cavaleriei	*Peperomia cavaleriei*	27	0	100	0
Peperomia cavaleriei	*Peperomia cavaleriei*	342	100	0	0
Peperomia cavaleriei	*Peperomia cavaleriei*	255	100	0	0
Peperomia cavispicata	*Peperomia cavispicata*	204	0	100	0
Peperomia cavispicata	*Peperomia cavispicata*	289	100	0	0
Peperomia celiae	*Peperomia celiae*	150	0	0	100
Peperomia celiae	*Peperomia celiae*	414	0	0	100
Peperomia celiae	*Peperomia celiae*	255	100	0	0
Peperomia cerea	*Peperomia cerea*	29	0	50	50
Peperomia cereoides	*Peperomia cereoides*	242	0	100	0
Peperomia cereoides	*Peperomia cereoides*	243	0	100	0
Peperomia ceroderma	*Peperomia ceroderma*	43	100	0	0
Peperomia ceroderma	*Peperomia ceroderma*	255	50	50	0
Peperomia cerrateae	*Peperomia cerrateae*	292	0	0	100
Peperomia cerrateae	*Peperomia cerrateae*	198	0	0	100
Peperomia chalhuapuquiana	*Peperomia chalhuapuquiana*	29	100	0	0
Peperomia chanchamayana	*Peperomia chanchamayana*	29	100	0	0
Peperomia chanchamayana	*Peperomia chanchamayana*	255	100	0	0
Peperomia chanchamayana	*Peperomia chanchamayana*	178	100	0	0
Peperomia chapensis	*Peperomia chapensis*	150	100	0	0
Peperomia chapensis	*Peperomia chapensis*	255	100	0	0
Peperomia chazaroi	*Peperomia chazaroi*	205	0	100	0
Peperomia chicamochana	*Peperomia chicamochana*	373	0	50	50
Peperomia chigorodoana	*Peperomia chigorodoana*	373	100	0	0
Peperomia chigorodoana	*Peperomia chigorodoana*	255	100	0	0
Peperomia chimboana	*Peperomia chimboana*	368	0	0	100
Peperomia chimboana	*Peperomia albispica*	335	100	0	0
Peperomia chlorodisca	*Peperomia chlorodisca*	373	100	0	0
Peperomia chlorodisca	*Peperomia chlorodisca*	255	100	0	0
Peperomia choritana	*Peperomia choritana*	373	15	0	85
Peperomia choritana	*Peperomia choritana*	255	100	0	0
Peperomia choroniana	*Peperomia choroniana*	51	50	0	50
Peperomia choroniana	*Peperomia choroniana*	43	5	0	95
Peperomia choroniana	*Peperomia choroniana*	150	50	0	50
Peperomia choroniana	*Peperomia choroniana*	219	100	0	0
Peperomia choroniana	*Peperomia choroniana*	338	0	0	100
Peperomia choroniana	*Peperomia choroniana*	374	38.4	0	61.6
Peperomia choroniana	*Peperomia choroniana*	21	100	0	0
Peperomia choroniana	*Peperomia choroniana*	373	50	0	50
Peperomia choroniana	*Peperomia choroniana*	255	50	0	50
Peperomia choroniana	*Peperomia choroniana*	285	0	100	0
Peperomia christophersenii	*Peperomia christophersenii*	55	0	0	100
Peperomia chrysotricha	*Peperomia chrysotricha*	29	0	0	100
Peperomia chutanka	*Peperomia chutanka*	245	0	100	0
Peperomia ciezae	*Peperomia ciezae*	247	0	100	0
Peperomia ciliaris	*Peperomia ciliaris*	43	100	0	0
Peperomia ciliaris	*Peperomia ciliaris*	57	0	0	100
Peperomia ciliaris	*Peperomia ciliaris*	374	100	0	0
Peperomia ciliaris	*Peperomia ciliaris*	373	100	0	0
Peperomia ciliaris	*Peperomia ciliaris*	255	100	0	0
Peperomia ciliatifolia	*Peperomia ciliatifolia*	29	100	0	0
Peperomia ciliatifolia	*Peperomia ciliatifolia*	255	100	0	0
Peperomia ciliatifolia	*Peperomia ciliatifolia*	178	100	0	0
Peperomia ciliatifolia	*Peperomia ciliatifolia*	29	0	0	100
Peperomia ciliatocaespitosa	*Peperomia ciliatocaespitosa*	82	0	50	50
Peperomia ciliatocaespitosa	*Peperomia ciliatocaespitosa*	198	0	100	0
Peperomia ciliatocaespitosa	*Peperomia ciliatocaespitosa*	51	0	50	50
Peperomia ciliatocaespitosa	*Peperomia ciliatocaespitosa*	46	0	50	50
Peperomia cilifolia	*Peperomia cilifolia*				
Peperomia ciliifolia	*Peperomia ciliifolia*	307	0	0	100
Peperomia ciliolata	*Peperomia ciliolata*				
Peperomia ciliolibractea	*Peperomia ciliolibractea*	43	100	0	0
Peperomia ciliolibractea	*Peperomia ciliolibractea*	57	0	0	100
Peperomia ciliolibractea	*Peperomia ciliolibractea*	60	95	0	5

Peperomia ciliolibractea	*Peperomia ciliolibractea*	255	50	50	0
Peperomia ciliosa	*Peperomia ciliosa*	255	100	0	0
Peperomia circinnata	*Peperomia circinnata*	51	100	0	0
Peperomia circinnata	*Peperomia circinnata*	80	100	0	0
Peperomia circinnata	*Peperomia circinnata*	87	100	0	0
Peperomia circinnata	*Peperomia circinnata*	150	100	0	0
Peperomia circinnata	*Peperomia circinnata*	419	100	0	0
Peperomia circinnata	*Peperomia circinnata*	29	100	0	0
Peperomia circinnata	*Peperomia circinnata*	86	100	0	0
Peperomia circinnata	*Peperomia circinnata*	113	0	100	0
Peperomia circinnata	*Peperomia circinnata*	223	100	0	0
Peperomia circinnata	*Peperomia circinnata*	47	100	0	0
Peperomia circinnata	*Peperomia circinnata*	42	0	0	100
Peperomia circinnata	*Peperomia circinnata*	418	100	0	0
Peperomia circinnata	*Peperomia circinnata*	224	100	0	0
Peperomia circinnata	*Peperomia circinnata*	175	100	0	0
Peperomia circinnata	*Peperomia circinnata*	255	100	0	0
Peperomia circinnata	*Peperomia circinnata*	375	100	0	0
Peperomia circinnata	*Peperomia desfontainesii*				
Peperomia circulifolia	*Peperomia circulifolia*	396	100	0	0
Peperomia cirillii-nelsonii	*Peperomia cirillii-nelsonii*	43	0	0	100
Peperomia cirillii-nelsonii	*Peperomia cirillii-nelsonii*	382	0	0	100
Peperomia cladara	*Peperomia cladara*	150	0	100	0
Peperomia cladara	*Peperomia cladara*	414	0	100	0
Peperomia cladara	*Peperomia cladara*	255	0	100	0
Peperomia cladara	*Peperomia cladara*	181	0	100	0
Peperomia claudii	*Peperomia claudii*	29	100	0	0
Peperomia claudii	*Peperomia claudii*	255	100	0	0
Peperomia claudii	*Peperomia claudii*	178	100	0	0
Peperomia claussenii	*Peperomia claussenii*	51	0	0	100
Peperomia claussenii	*Peperomia claussenii*	82	0	0	100
Peperomia clavatispica	*Peperomia clavatispica*	29	0	0	100
Peperomia clavatispica	*Peperomia clavatispica*	373	0	0	100
Peperomia clavigera	*Peperomia clavigera*	43	0	50	50
Peperomia clavigera	*Peperomia clavigera*	328	0	100	0
Peperomia clavigera	*Peperomia clavigera*	207	0	0	100
Peperomia clavigera	*Peperomia clavigera*	255	0	50	50
Peperomia claytonioides	*Peperomia claytonioides*	43	0	50	50
Peperomia claytonioides	*Peperomia claytonioides*	328	33.3	33.3	33.4
Peperomia claytonioides	*Peperomia claytonioides*	335	0	50	50
Peperomia claytonioides	*Peperomia claytonioides*	255	50	50	0
Peperomia claytonioides	*Peperomia claytonioides*	37	0	50	50
Peperomia claytonioides	*Peperomia schizostachya*				
Peperomia claytonioides	*Peperomia sciaphila*				
Peperomia clivicola	*Peperomia clivicola*	51	50	50	0
Peperomia clivicola	*Peperomia clivicola*	82	50	50	0
Peperomia clivicola	*Peperomia clivicola*	255	100	0	0
Peperomia clivicola	*Peperomia clivicola*	220	50	50	0
Peperomia clivigaudens	*Peperomia clivigaudens*	414	0	0	100
Peperomia clusiifolia	*Peperomia clusiifolia*	255	100	0	0
Peperomia clusiifolia	*Peperomia clusiifolia*	112	33.3	33.3	33.4
Peperomia coatzacoalcosensis	*Peperomia coatzacoalcosensis*	43	100	0	0
Peperomia coatzacoalcosensis	*Peperomia coatzacoalcosensis*	110	100	0	0
Peperomia coatzacoalcosensis	*Peperomia coatzacoalcosensis*	255	100	0	0
Peperomia cobana	*Peperomia cobana*	43	85	0	15
Peperomia cobana	*Peperomia cobana*	110	45	45	10
Peperomia cobana	*Peperomia cobana*	328	100	0	0
Peperomia cobana	*Peperomia cobana*	255	100	0	0
Peperomia cobana	*Peperomia cobana*	176	100	0	0
Peperomia cochinensis	*Peperomia cochinensis*	340	100	0	0
Peperomia cochinensis	*Peperomia cochinensis*	342	100	0	0
Peperomia cochinensis	*Peperomia cochinensis*	255	100	0	0
Peperomia cocleana	*Peperomia cocleana*	43	100	0	0
Peperomia cocleana	*Peperomia cocleana*	57	0	0	100
Peperomia cocleana	*Peperomia cocleana*	255	100	0	0
Peperomia coenosa	*Peperomia coenosa*	373	0	0	100
Peperomia cogniauxii	*Peperomia cogniauxii*	172	100	0	0
Peperomia cogniauxii	*Peperomia cogniauxii*	171	50	50	0
Peperomia cogniauxii	*Peperomia cogniauxii*	255	100	0	0
Peperomia cogniauxii	*Peperomia cogniauxii*	260	50	0	50
Peperomia cogniauxii	*Peperomia crispipila*				
Peperomia collinsii	*Peperomia collinsii*	418	85	0	15
Peperomia collinsii	*Peperomia collinsii*	255	100	0	0
Peperomia collocata	*Peperomia collocata*	328	85	0	15
Peperomia coloniae	*Peperomia coloniae*	29	0	0	100
Peperomia colorata	*Peperomia colorata*	368	0	0	100
Peperomia colorata	*Peperomia colorata*	373	0	0	100
Peperomia colossina	*Peperomia colossina*				
Peperomia columella	*Peperomia columella*	29	0	0	100
Peperomia columella	*Peperomia columella*	196	0	50	50

Peperomia columnaris	*Peperomia columnaris*	244	0	50	50
Peperomia comaltitlanensis	*Peperomia comaltitlanensis*	43	0	50	50
Peperomia comaltitlanensis	*Peperomia comaltitlanensis*	255	0	50	50
Peperomia comarapana	*Peperomia comarapana*	183	1	0	99
Peperomia comarapana	*Peperomia comarapana*	184	5	0	95
Peperomia comarapana	*Peperomia comarapana*	412	0	0	100
Peperomia comarapana	*Peperomia comarapana*	418	0	0	100
Peperomia comarapana	*Peperomia comarapana*	375	50	0	50
Peperomia commersonii	*Peperomia commersonii*	255	100	0	0
Peperomia concava	*Peperomia concava*	29	0	0	100
Peperomia condoris	*Peperomia condoris*	373	0	0	100
Peperomia condormiens	*Peperomia condormiens*	328	85	0	15
Peperomia condotoana	*Peperomia condotoana*				
Peperomia confertispica	*Peperomia confertispica*	29	0	0	100
Peperomia confertispica	*Peperomia confertispica*	178	0	0	100
Peperomia confertispica	*Peperomia confertispica*	29	0	0	100
Peperomia congerro	*Peperomia congerro*	373	0	0	100
Peperomia congesta	*Peperomia congesta*	29	0	0	100
Peperomia congesta	*Peperomia congesta*	193	0	100	0
Peperomia congestispica	*Peperomia congestispica*	29	0	0	100
Peperomia congona	*Peperomia congona*				
Peperomia conjugata	*Peperomia conjugata*	197	0	50	50
Peperomia connixa	*Peperomia connixa*	29	0	0	100
Peperomia connixa	*Peperomia connixa*	373	0	30	70
Peperomia conocarpa	*Peperomia conocarpa*	255	100	0	0
Peperomia conocarpa	*Peperomia chucanebana*	176	100	0	0
Peperomia conocarpa	*Peperomia chucanebana*	325	15	0	85
Peperomia consoquitlana	*Peperomia consoquitlana*	110	100	0	0
Peperomia consoquitlana	*Peperomia consoquitlana*	255	100	0	0
Peperomia consoquitlana	*Peperomia consoquitlana*	281	0	50	50
Peperomia conturbans	*Peperomia conturbans*				
Peperomia convexa	*Peperomia convexa*	217	100	0	0
Peperomia convexa	*Peperomia recurvata*	238	50	50	0
Peperomia convexa	*Peperomia reinwardtiana*	217	100	0	0
Peperomia cookiana	*Peperomia kohalana*	73	0	0	100
Peperomia cookiana	*Peperomia cookiana*	255	100	0	0
Peperomia cookiana	*Peperomia cookiana*	387	33.3	33.3	33.4
Peperomia cookiana	*Peperomia asperulata*	29	0	0	100
Peperomia cookiana	*Peperomia pleistostachya*	148	0	0	100
Peperomia cookiana	*Peperomia opacilimba*				
Peperomia cookiana	*Peperomia flavinerva*				
Peperomia cookiana	*Peperomia hiloana*				
Peperomia cookiana	*Peperomia javanica*				
Peperomia cookiana	*Peperomia knudsenii*				
Peperomia cookiana	*Peperomia pukooana*				
Peperomia cookiana	*Peperomia refractifolia*				
Peperomia cookiana	*Peperomia minutilimba*				
Peperomia cookiana	*Peperomia kamoloana*				
Peperomia copelandii	*Peperomia copelandii*				
Peperomia coquimbensis	*Peperomia coquimbensis*	255	100	0	0
Peperomia corcovadensis	*Peperomia corcovadensis*	51	50	50	0
Peperomia corcovadensis	*Peperomia corcovadensis*	120	50	50	0
Peperomia corcovadensis	*Peperomia corcovadensis*	273	50	50	0
Peperomia corcovadensis	*Peperomia corcovadensis*	220	50	50	0
Peperomia corcovadensis	*Peperomia corcovadensis*	179	33.3	33.3	33.4
Peperomia corcovadensis	*Peperomia corcovadensis*	221	100	0	0
Peperomia corcovadensis	*Peperomia corcovadensis*	48	100	0	0
Peperomia corcovadensis	*Peperomia corcovadensis*	84	50	50	0
Peperomia corcovadensis	*Peperomia corcovadensis*	85	50	50	0
Peperomia corcovadensis	*Peperomia corcovadensis*	418	50	50	0
Peperomia corcovadensis	*Peperomia corcovadensis*	161	50	50	0
Peperomia corcovadensis	*Peperomia corcovadensis*	156	100	0	0
Peperomia corcovadensis	*Peperomia corcovadensis*	255	100	0	0
Peperomia corcovadensis	*Peperomia corcovadensis*	128	100	0	0
Peperomia corcovadensis	*Peperomia subsessilifolia*	70	100	0	0
Peperomia corcovadensis	*Peperomia guarujana*	51	100	0	0
Peperomia corcovadensis	*Peperomia lundii*				
Peperomia corcovadensis	*Peperomia saldasiana*				
Peperomia cordata	*Peperomia cordata*	373	0	0	100
Peperomia cordifolia	*Peperomia cordifolia*				
Peperomia cordigera	*Peperomia cordigera*	51	0	100	0
Peperomia cordigera	*Peperomia cordigera*	82	100	0	0
Peperomia cordigera	*Peperomia cordigera*	255	100	0	0
Peperomia cordovana	*Peperomia cordovana*	110	100	0	0
Peperomia cordovana	*Peperomia cordovana*	255	100	0	0
Peperomia cordulata	*Peperomia cordulata*	43	100	0	0
Peperomia cordulata	*Peperomia cordulata*	57	100	0	0
Peperomia cordulata	*Peperomia cordulata*	60	100	0	0
Peperomia cordulata	*Peperomia cordulata*	21	100	0	0
Peperomia cordulata	*Peperomia cordulata*	255	100	0	0

Peperomia cordulatiformis	*Peperomia cordulatiformis*	43	0	0	100
Peperomia cordulatiformis	*Peperomia cordulatiformis*	57	0	0	100
Peperomia cordulatiformis	*Peperomia cordulatiformis*	219	0	0	100
Peperomia cordulatiformis	*Peperomia cordulatiformis*	21	100	0	0
Peperomia cordulatiformis	*Peperomia cordulatiformis*	255	100	0	0
Peperomia cordulatiformis	*Peperomia digitinervia*	57	100	0	0
Peperomia cordulilimba	*Peperomia cordulilimba*	29	100	0	0
Peperomia cordulilimba	*Peperomia cordulilimba*	255	100	0	0
Peperomia cordulilimba	*Peperomia cordulilimba*	178	100	0	0
Peperomia coroicoensis	*Peperomia coroicoensis*	412	50	0	50
Peperomia costaricensis	*Peperomia costaricensis*	37	85	0	15
Peperomia costata	*Peperomia costata*	199	50	0	50
Peperomia costata	*Peperomia costata*	255	100	0	0
Peperomia cotoneasterifolia	*Peperomia cotoneasterifolia*	29	0	0	100
Peperomia cotoneasterifolia	*Peperomia cotoneasterifolia*	178	100	0	0
Peperomia cotyledon	*Peperomia cotyledon*	374	0	0	100
Peperomia coulteri	*Peperomia coulteri*	255	100	0	0
Peperomia cowanii	*Peperomia cowanii*	51	100	0	0
Peperomia crassicaulis	*Peperomia crassicaulis*	255	100	0	0
Peperomia crassicaulis	*Peperomia crassicaulis*	112	50	0	50
Peperomia crassicaulis	*Peperomia unguiculata*				
Peperomia crassicaulis	*Peperomia leoclemerocana*				
Peperomia crassispica	*Peperomia crassispica*	255	100	0	0
Peperomia crassulicaulis	*Peperomia crassulicaulis*	29	0	0	100
Peperomia crinicaulis	*Peperomia crinicaulis*	51	100	0	0
Peperomia crinicaulis	*Peperomia crinicaulis*	82	100	0	0
Peperomia crinicaulis	*Peperomia crinicaulis*	220	100	0	0
Peperomia crinicaulis	*Peperomia crinicaulis*	179	0	100	0
Peperomia crinicaulis	*Peperomia crinicaulis*	48	50	50	0
Peperomia crinicaulis	*Peperomia crinicaulis*	255	100	0	0
Peperomia crinigera	*Peperomia crinigera*	29	0	0	100
Peperomia crispa	*Peperomia crispa*	374	0	0	100
Peperomia crispa	*Peperomia crispa*	373	0	0	100
Peperomia crispa	*Peperomia suizana*				
Peperomia crispipetiola	*Peperomia crispipetiola*	43	100	0	0
Peperomia crispipetiola	*Peperomia crispipetiola*	219	100	0	0
Peperomia crispipetiola	*Peperomia crispipetiola*	255	100	0	0
Peperomia crispipetiola	*Peperomia tatamana*	21	100	0	0
Peperomia croizatiana	*Peperomia croizatiana*	150	100	0	0
Peperomia croizatiana	*Peperomia croizatiana*	255	100	0	0
Peperomia crotalophora	*Peperomia semielongata*	29	0	0	100
Peperomia crotalophora	*Peperomia crotalophora*	40	100	0	0
Peperomia crotalophora	*Peperomia crotalophora*	29	0	0	100
Peperomia crotalophora	*Peperomia crotalophora*	373	0	0	100
Peperomia crotalophora	*Peperomia crotalophora*	178	0	0	100
Peperomia cruentata	*Peperomia cruentata*	43	100	0	0
Peperomia cruentata	*Peperomia cruentata*	255	100	0	0
Peperomia crusculibacca	*Peperomia crusculibacca*	29	0	0	100
Peperomia cruzeirensis	*Peperomia cruzeirensis*	51	100	0	0
Peperomia cruzeirensis	*Peperomia cruzeirensis*	49	100	0	0
Peperomia cruzeirensis	*Peperomia cruzeirensis*	82	100	0	0
Peperomia crypticola	*Peperomia crypticola*	51	0	100	0
Peperomia crypticola	*Peperomia crypticola*	82	0	100	0
Peperomia crypticola	*Peperomia crypticola*	188	0	100	0
Peperomia cryptostachya	*Peperomia cryptostachya*	373	100	0	0
Peperomia cryptostachya	*Peperomia cryptostachya*	255	100	0	0
Peperomia crystallina	*Peperomia crystallina*	51	50	0	50
Peperomia crystallina	*Peperomia crystallina*	29	0	0	100
Peperomia crystallina	*Peperomia mandonii*	29	0	50	50
Peperomia crystallina	*Peperomia nonhispidula*	29	0	0	100
Peperomia crystallina	*Peperomia umbelliformis*	29	0	0	100
Peperomia crystallina	*Peperomia crystallina*	418	0	0	100
Peperomia crystallina	*Peperomia crystallina*	354	0	100	0
Peperomia cuatrecasasana	*Peperomia cuatrecasasana*	373	100	0	0
Peperomia cubensis	*Peperomia cubensis*	294	0	50	50
Peperomia cubensis	*Peperomia cubensis*	255	100	0	0
Peperomia cubugonana	*Peperomia cubugonana*	373	100	0	0
Peperomia cubugonana	*Peperomia cubugonana*	255	100	0	0
Peperomia cuchumatanica	*Peperomia cuchumatanica*	43	0	0	100
Peperomia cuchumatanica	*Peperomia cuchumatanica*	255	0	0	100
Peperomia cumbreana	*Peperomia cumbreana*	373	100	0	0
Peperomia cumbreana	*Peperomia cumbreana*	255	100	0	0
Peperomia cundinamarcana	*Peperomia cundinamarcana*	373	0	0	100
Peperomia cuprea	*Peperomia cuprea*	29	0	0	100
Peperomia cuprea	*Peperomia cuprea*	255	100	0	0
Peperomia cuprea	*Peperomia cuprea*	29	0	0	100
Peperomia cupularis	*Peperomia cupularis*				
Peperomia curruciformis	*Peperomia curruciformis*	29	0	0	100
Peperomia curruciformis	*Peperomia curruciformis*	178	0	0	100
Peperomia curticaulis	*Peperomia curticaulis*	29	0	0	100

Peperomia curtipes	*Peperomia curtipes*	29	0	0	100
Peperomia curtipes	*Peperomia brevicaulis*	29	0	0	100
Peperomia curtipes	*Peperomia curtipes*	29	0	0	100
Peperomia curtispica	*Peperomia curtispica*	409	100	0	0
Peperomia curtispica	*Peperomia curtispica*	307	100	0	0
Peperomia curtispica	*Peperomia curtispica*	255	100	0	0
Peperomia cushiana	*Peperomia cushiana*	29	0	0	100
Peperomia cushiana	*Peperomia cushiana*	255	100	0	0
Peperomia cusilluyocana	*Peperomia cusilluyocana*	29	0	0	100
Peperomia cuspidata	*Peperomia cuspidata*	294	0	0	100
Peperomia cuspidata	*Peperomia cuspidata*	255	100	0	0
Peperomia cuspidilimba	*Peperomia cuspidilimba*	373	0	0	100
Peperomia cuspidilimba	*Peperomia cuspidilimba*	255	100	0	0
Peperomia cyclaminoides	*Peperomia cyclaminoides*	147	0	0	100
Peperomia cyclophylla	*Peperomia cyclophylla*	51	100	0	0
Peperomia cyclophylla	*Peperomia cyclophylla*	43	100	0	0
Peperomia cyclophylla	*Peperomia cyclophylla*	57	0	0	100
Peperomia cyclophylla	*Peperomia cyclophylla*	110	100	0	0
Peperomia cyclophylla	*Peperomia cyclophylla*	150	0	0	100
Peperomia cyclophylla	*Peperomia cyclophylla*	21	100	0	0
Peperomia cyclophylla	*Peperomia cyclophylla*	29	0	0	100
Peperomia cyclophylla	*Peperomia cyclophylla*	335	100	0	0
Peperomia cyclophylla	*Peperomia cyclophylla*	42	100	0	0
Peperomia cyclophylla	*Peperomia cyclophylla*	255	50	50	0
Peperomia cyclophylla	*Peperomia cyclophylla*	37	100	0	0
Peperomia cylindrica	*Peperomia cylindrica*				
Peperomia cymbifolia	*Peperomia cymbifolia*	251	0	100	0
Peperomia cymbifolia	*Peperomia cymbifolia*	255	0	100	0
Peperomia cymbifolia	*Peperomia cymbifolia*	244	0	50	50
Peperomia cymbifolia	*Peperomia cymbifolia*	251	0	100	0
Peperomia daguana	*Peperomia daguana*	255	100	0	0
Peperomia dahlstedtii	*Peperomia dahlstedtii*	51	100	0	0
Peperomia dahlstedtii	*Peperomia dahlstedtii*	312	100	0	0
Peperomia dahlstedtii	*Peperomia dahlstedtii*	47	0	50	50
Peperomia dahlstedtii	*Peperomia dahlstedtii*	255	100	0	0
Peperomia damazioi	*Peperomia damazioi*	69	100	0	0
Peperomia darienensis	*Peperomia darienensis*	43	50	50	0
Peperomia darienensis	*Peperomia darienensis*	255	50	50	0
Peperomia dasystachya	*Peperomia dasystachya*	29	0	0	100
Peperomia dauleana	*Peperomia dauleana*	373	0	0	100
Peperomia dauleana	*Peperomia dauleana*	255	100	0	0
Peperomia davidsoniae	*Peperomia davidsoniae*	43	0	0	100
Peperomia davidsoniae	*Peperomia davidsoniae*	57	0	0	100
Peperomia debilipes	*Peperomia debilipes*	29	100	0	0
Peperomia debilipes	*Peperomia debilipes*	255	100	0	0
Peperomia debilipes	*Peperomia debilipes*	178	100	0	0
Peperomia debilipes	*Peperomia debilipes*	29	0	0	100
Peperomia deceptrix	*Peperomia deceptrix*	29	100	0	0
Peperomia deceptrix	*Peperomia deceptrix*	255	100	0	0
Peperomia deceptrix	*Peperomia deceptrix*	178	100	0	0
Peperomia decipiens	*Peperomia decipiens*	214	0	50	50
Peperomia decora	*Peperomia decora*	51	0	100	0
Peperomia decora	*Peperomia decora*	82	0	100	0
Peperomia decora	*Peperomia decora*	255	100	0	0
Peperomia decumbens	*Peperomia decumbens*	376	0	0	100
Peperomia decumbens	*Peperomia decumbens*	255	100	0	0
Peperomia decurrens	*Peperomia decurrens*	150	0	50	50
Peperomia decurrens	*Peperomia decurrens*	338	0	100	0
Peperomia deficiens	*Peperomia deficiens*	29	0	0	100
Peperomia defoliata	*Peperomia defoliata*	373	0	0	100
Peperomia defoliata	*Peperomia defoliata*	255	100	0	0
Peperomia degeneri	*Peperomia degeneri*	387	0	100	0
Peperomia degeneri	*Peperomia degeneri*	255	100	0	0
Peperomia delascioi	*Peperomia delascioi*	150	0	0	100
Peperomia delascioi	*Peperomia delascioi*	83	0	0	100
Peperomia delascioi	*Peperomia delascioi*	255	100	0	0
Peperomia delicatula	*Peperomia delicatula*	51	100	0	0
Peperomia delicatula	*Peperomia delicatula*	179	100	0	0
Peperomia delicatula	*Peperomia delicatula*	42	100	0	0
Peperomia delicatula	*Peperomia delicatula*	418	100	0	0
Peperomia delicatula	*Peperomia delicatula*	161	100	0	0
Peperomia delicatula	*Peperomia delicatula*	255	100	0	0
Peperomia delicatula	*Peperomia delicatula*	252	100	0	0
Peperomia dendroides	*Peperomia dendroides*	178	100	0	0
Peperomia dendrophila	*Peperomia dendrophila*	57	0	0	100
Peperomia dendrophila	*Peperomia dendrophila*	110	90	0	10
Peperomia dendrophila	*Peperomia dendrophila*	150	50	0	50
Peperomia dendrophila	*Peperomia dendrophila*	213	50	50	0
Peperomia dendrophila	*Peperomia dendrophila*	219	50	0	50
Peperomia dendrophila	*Peperomia dendrophila*	374	50	0	50

Peperomia dendrophila	*Peperomia dendrophila*	21	100	0	0
Peperomia dendrophila	*Peperomia dendrophila*	335	85	0	15
Peperomia dendrophila	*Peperomia dendrophila*	255	50	0	50
Peperomia dendrophila	*Peperomia dendrophila*	112	100	0	0
Peperomia dendrophila	*Peperomia montis-verticis*	399	100	0	0
Peperomia dendrophila	*Peperomia novae-helvetiae*	3	100	0	0
Peperomia dendrophila	*Peperomia dendrophila*	90	50	0	50
Peperomia densifolia	*Peperomia densifolia*	373	0	0	100
Peperomia dependens	*Peperomia dependens*	29	0	0	100
Peperomia dependens	*Peperomia cordifolia*	134	33.3	33.3	33.4
Peperomia dependens	*Peperomia cordifolia*	112	50	50	0
Peperomia dependens	*Peperomia opiziana*	29	0	0	100
Peperomia deppeana	*Peperomia deppeana*	31	100	0	0
Peperomia deppeana	*Peperomia deppeana*	43	100	0	0
Peperomia deppeana	*Peperomia deppeana*	57	100	0	0
Peperomia deppeana	*Peperomia deppeana*	110	100	0	0
Peperomia deppeana	*Peperomia deppeana*	219	100	0	0
Peperomia deppeana	*Peperomia deppeana*	328	100	0	0
Peperomia deppeana	*Peperomia deppeana*	335	100	0	0
Peperomia deppeana	*Peperomia deppeana*	15	100	0	0
Peperomia deppeana	*Peperomia deppeana*	255	50	50	0
Peperomia deppeana	*Peperomia deppeana*	37	100	0	0
Peperomia deppeana	*Peperomia polochicana*	176	100	0	0
Peperomia deppeana	*Peperomia sepicola*	367	100	0	0
Peperomia deppeana	*Peperomia standleyi*				
Peperomia diamantinensis	*Peperomia diamantinensis*	51	50	0	50
Peperomia diamantinensis	*Peperomia diamantinensis*	49	0	50	50
Peperomia diamantinensis	*Peperomia diamantinensis*	82	0	50	50
Peperomia diaphanoides	*Peperomia diaphanoides*	51	50	0	50
Peperomia diaphanoides	*Peperomia diaphanoides*	220	100	0	0
Peperomia diaphanoides	*Peperomia diaphanoides*	179	100	0	0
Peperomia diaphanoides	*Peperomia diaphanoides*	221	100	0	0
Peperomia diaphanoides	*Peperomia diaphanoides*	418	0	0	100
Peperomia diaphanoides	*Peperomia diaphanoides*	255	100	0	0
Peperomia dichotoma	*Peperomia dichotoma*	51	100	0	0
Peperomia dichotoma	*Peperomia dichotoma*	82	100	0	0
Peperomia dichotoma	*Peperomia dichotoma*	273	100	0	0
Peperomia diffusa	*Peperomia diffusa*	373	0	0	100
Peperomia dimota	*Peperomia dimota*	373	0	0	100
Peperomia dimota	*Peperomia dimota*	255	100	0	0
Peperomia dindygulensis	*Peperomia dindygulensis*	272	0	100	0
Peperomia dindygulensis	*Peperomia dindygulensis*	341	0	100	0
Peperomia dindygulensis	*Peperomia dindygulensis*	342	0	100	0
Peperomia dindygulensis	*Peperomia dindygulensis*	391	50	0	50
Peperomia discifolia	*Peperomia discifolia*	373	0	0	100
Peperomia discifolia	*Peperomia discifolia*	255	100	0	0
Peperomia discilimba	*Peperomia discilimba*	150	50	0	50
Peperomia discilimba	*Peperomia discilimba*	373	0	0	100
Peperomia discolor	*Peperomia discolor*				
Peperomia disjunctiflora	*Peperomia disjunctiflora*	414	0	0	100
Peperomia disjunctiflora	*Peperomia disjunctiflora*	255	100	0	0
Peperomia distachyos	*Peperomia distachyos*	172	50	50	0
Peperomia distachyos	*Peperomia distachyos*	29	100	0	0
Peperomia distachyos	*Peperomia distachyos*	373	0	0	100
Peperomia distachyos	*Peperomia distachyos*	171	50	50	0
Peperomia distachyos	*Peperomia distachyos*	134	0	100	0
Peperomia distachyos	*Peperomia distachyos*	335	100	0	0
Peperomia distachyos	*Peperomia distachyos*	294	40	40	20
Peperomia distachyos	*Peperomia distachyos*	260	50	0	50
Peperomia distachyos	*Peperomia distachyos*	37	100	0	0
Peperomia distachyos	*Peperomia distachyos*	112	50	50	0
Peperomia distachyos	*Peperomia distachyos*	191	50	0	50
Peperomia distachyos	*Peperomia distachyos*	51	50	0	50
Peperomia distachyos	*Peperomia distachyos*	43	33.3	33.3	33.4
Peperomia distachyos	*Peperomia distachyos*	57	0	0	100
Peperomia distachyos	*Peperomia distachyos*	110	100	0	0
Peperomia distachyos	*Peperomia distachyos*	150	0	0	100
Peperomia distachyos	*Peperomia distachyos*	374	87.5	0	12.5
Peperomia distachyos	*Peperomia elegans*	29	0	0	100
Peperomia distachyos	*Peperomia distachyos*	255	50	50	0
Peperomia distachyos	*Peperomia ovatolanceolata*	373	50	0	50
Peperomia distachyos	*Peperomia foveolata*	150	50	50	0
Peperomia disticha	*Peperomia disticha*	415	100	0	0
Peperomia disticha	*Peperomia disticha*	307	100	0	0
Peperomia disticha	*Peperomia disticha*	255	100	0	0
Peperomia divaricata	*Peperomia divaricata*	51	0	100	0
Peperomia diversifolia	*Peperomia diversifolia*	386	0	100	0
Peperomia doellii	*Peperomia doellii*				
Peperomia dolabella	*Peperomia dolabella*	29	0	0	100
Peperomia dolabella	*Peperomia dolabella*	255	0	0	100

Peperomia dolabriformis	*Peperomia dolabriformis*	29	0	50	50
Peperomia dolabriformis	*Peperomia dolabriformis*	374	0	0	100
Peperomia dolabriformis	*Peperomia dolabriformis*	29	0	50	50
Peperomia dolabriformis	*Peperomia dolabriformis*	244	0	100	0
Peperomia dolabriformis	*Peperomia dolabriformis*	245	0	100	0
Peperomia dolabriformis	*Peperomia dolabriformis*	245	0	0	100
Peperomia dolabriformis	*Peperomia dolabriformis*	29	0	0	100
Peperomia dominicana	*Peperomia dominicana*	255	100	0	0
Peperomia donaguiana	*Peperomia donaguiana*	52	100	0	0
Peperomia donaguiana	*Peperomia donaguiana*	110	33.3	33.3	33.4
Peperomia donaguiana	*Peperomia donaguiana*	293	33.3	33.3	33.4
Peperomia dondonensis	*Peperomia dondonensis*				
Peperomia donnell-smithii	*Peperomia donnell-smithii*	219	100	0	0
Peperomia dorstenioides	*Peperomia dorstenioides*	43	0	50	50
Peperomia dorstenioides	*Peperomia dorstenioides*	328	0	50	50
Peperomia dorstenioides	*Peperomia dorstenioides*	255	0	50	50
Peperomia dotana	*Peperomia dotana*	43	0	0	100
Peperomia dotana	*Peperomia dotana*	255	50	0	50
Peperomia dotana	*Peperomia dotana*	37	50	0	50
Peperomia dotana	*Peperomia dotana*	37	50	0	50
Peperomia dotana	*Peperomia isidroana*				
Peperomia drapeta	*Peperomia drapeta*	29	0	0	100
Peperomia drapeta	*Peperomia drapeta*	255	100	0	0
Peperomia drapeta	*Peperomia drapeta*	178	100	0	0
Peperomia drusophila	*Peperomia drusophila*	110	100	0	0
Peperomia drusophila	*Peperomia drusophila*	255	100	0	0
Peperomia dryadica	*Peperomia dryadica*	190	5	95	0
Peperomia dryadum	*Peperomia dryadum*	255	100	0	0
Peperomia duartei	*Peperomia duartei*	51	0	0	100
Peperomia duartei	*Peperomia duartei*	82	0	0	100
Peperomia dubia	*Peperomia dubia*	91	100	0	0
Peperomia dubia	*Peperomia dubia*	16	100	0	0
Peperomia dubia	*Peperomia dubia*	255	100	0	0
Peperomia duendensis	*Peperomia duendensis*	373	0	0	100
Peperomia duendensis	*Peperomia duendensis*	255	100	0	0
Peperomia duidana	*Peperomia duidana*	374	100	0	0
Peperomia duidana	*Peperomia duidana*	21	100	0	0
Peperomia durandii	*Peperomia durandii*	198	0	0	100
Peperomia duricaulis	*Peperomia duricaulis*	43	50	0	50
Peperomia duricaulis	*Peperomia duricaulis*	219	50	0	50
Peperomia duricaulis	*Peperomia duricaulis*	255	50	0	50
Peperomia dusenii	*Peperomia dusenii*	91	100	0	0
Peperomia dusenii	*Peperomia dusenii*	255	100	0	0
Peperomia dyscrita	*Peperomia dyscrita*	43	50	50	0
Peperomia dyscrita	*Peperomia dyscrita*	255	50	50	0
Peperomia ebingeri	*Peperomia ebingeri*	43	100	0	0
Peperomia ebingeri	*Peperomia ebingeri*	57	0	0	100
Peperomia ebingeri	*Peperomia ebingeri*	60	100	0	0
Peperomia ebingeri	*Peperomia ebingeri*	21	100	0	0
Peperomia ebingeri	*Peperomia ebingeri*	416	100	0	0
Peperomia ebingeri	*Peperomia ebingeri*	255	100	0	0
Peperomia ebingeri	*Peperomia ebingeri*	37	100	0	0
Peperomia eburnea	*Peperomia eburnea*	374	60	0	40
Peperomia ecuadorensis	*Peperomia ecuadorensis*	373	0	0	100
Peperomia edulis	*Peperomia edulis*	293	100	0	0
Peperomia eekana	*Peperomia eekana*	255	100	0	0
Peperomia eekana	*Peperomia nahikuensis*	313	100	0	0
Peperomia eekana	*Peperomia sarcostigma*				
Peperomia eekana	*Peperomia treleasei*				
Peperomia effusa	*Peperomia effusa*	255	100	0	0
Peperomia effusa	*Peperomia effusa*	346	0	0	100
Peperomia efimbriata	*Peperomia efimbriata*	255	100	0	0
Peperomia eggersii	*Peperomia eggersii*	387	85	0	15
Peperomia eggersii	*Peperomia eggersii*	255	100	0	0
Peperomia egleri	*Peperomia egleri*	51	100	0	0
Peperomia egleri	*Peperomia egleri*	82	100	0	0
Peperomia ekakesara	*Peperomia ekakesara*	198	0	100	0
Peperomia ekakesara	*Peperomia ekakesara*	262	0	100	0
Peperomia elata	*Peperomia elata*	43	50	50	0
Peperomia elata	*Peperomia elata*	57	0	0	100
Peperomia elata	*Peperomia elata*	219	100	0	0
Peperomia elata	*Peperomia elata*	255	50	50	0
Peperomia elata	*Peperomia elata*	37	0	0	100
Peperomia elatior	*Peperomia elatior*	204	0	100	0
Peperomia elegantifolia	*Peperomia elegantifolia*	29	0	0	100
Peperomia elliptica	*Peperomia mauritiana*				
Peperomia elliptica	*Peperomia decaisnei*				
Peperomia elliptica	*Peperomia serpyllifolia*				
Peperomia elliptica	*Peperomia elliptica*	255	100	0	0
Peperomia ellipticibacca	*Peperomia ellipticibacca*	387	50	0	50

Peperomia ellipticibacca	*Peperomia ellipticibacca*	255	50	0	50
Peperomia ellipticorhombea	*Peperomia ellipticorhombea*	29	0	0	100
Peperomia ellipticorhombea	*Peperomia ellipticorhombea*	255	100	0	0
Peperomia ellsworthii	*Peperomia ellsworthii*	373	0	0	100
Peperomia ellsworthii	*Peperomia ellsworthii*	255	100	0	0
Peperomia elmeri	*Peperomia elmeri*	70	100	0	0
Peperomia elmeri	*Peperomia elmeri*	238	100	0	0
Peperomia elmeri	*Peperomia elmeri*	255	100	0	0
Peperomia elongata	*Peperomia elongata*	18	100	0	0
Peperomia elongata	*Peperomia elongata*	51	50	0	50
Peperomia elongata	*Peperomia elongata*	110	100	0	0
Peperomia elongata	*Peperomia elongata*	150	33.3	33.3	33.4
Peperomia elongata	*Peperomia elongata*	374	50	0	50
Peperomia elongata	*Peperomia elongata*	412	100	0	0
Peperomia elongata	*Peperomia elongata*	29	100	0	0
Peperomia elongata	*Peperomia elongata*	373	100	0	0
Peperomia elongata	*Peperomia elongata*	261	100	0	0
Peperomia elongata	*Peperomia elongata*	86	50	50	0
Peperomia elongata	*Peperomia elongata*	214	100	0	0
Peperomia elongata	*Peperomia elongata*	42	100	0	0
Peperomia elongata	*Peperomia elongata*	85	50	0	50
Peperomia elongata	*Peperomia elongata*	191	100	0	0
Peperomia elongata	*Peperomia macedoana*	255	100	0	0
Peperomia elongata	*Peperomia piresii*	42	100	0	0
Peperomia elongata	*Peperomia piresii*	255	100	0	0
Peperomia elongata	*Peperomia controversa*	42	100	0	0
Peperomia elongata	*Peperomia barbulipetiola*				
Peperomia elsana	*Peperomia elsana*	373	0	0	100
Peperomia elsiae	*Peperomia elsiae*				
Peperomia elsieae	*Peperomia pseudoestrellensis*	51	50	0	50
Peperomia elsieae	*Peperomia pseudoestrellensis*	82	50	0	50
Peperomia elsieae	*Peperomia pseudoestrellensis*	255	100	0	0
Peperomia elsieae	*Peperomia pseudoestrellensis*	220	50	0	50
Peperomia elsieae	*Peperomia pseudoestrellensis*	221	100	0	0
Peperomia elsieae	*Peperomia pseudoestrellensis*	85	33.3	33.4	33.3
Peperomia elsieae	*Peperomia pseudoestrellensis*	128	100	0	0
Peperomia emarginatifolia	*Peperomia emarginatifolia*	192	100	0	0
Peperomia emarginella	*Peperomia emarginella*	24	100	0	0
Peperomia emarginella	*Peperomia emarginella*	51	100	0	0
Peperomia emarginella	*Peperomia emarginella*	39	100	0	0
Peperomia emarginella	*Peperomia emarginella*	43	100	0	0
Peperomia emarginella	*Peperomia emarginella*	57	100	0	0
Peperomia emarginella	*Peperomia emarginella*	110	100	0	0
Peperomia emarginella	*Peperomia emarginella*	150	50	50	0
Peperomia emarginella	*Peperomia emarginella*	227	50	50	0
Peperomia emarginella	*Peperomia emarginella*	21	100	0	0
Peperomia emarginella	*Peperomia emarginella*	83	50	50	0
Peperomia emarginella	*Peperomia emarginella*	232	70	0	30
Peperomia emarginella	*Peperomia emarginella*	172	50	50	0
Peperomia emarginella	*Peperomia emarginella*	29	100	0	0
Peperomia emarginella	*Peperomia emarginella*	373	50	50	0
Peperomia emarginella	*Peperomia emarginella*	171	50	50	0
Peperomia emarginella	*Peperomia emarginella*	335	100	0	0
Peperomia emarginella	*Peperomia emarginella*	294	100	0	0
Peperomia emarginella	*Peperomia emarginella*	153	100	0	0
Peperomia emarginella	*Peperomia emarginella*	255	50	50	0
Peperomia emarginella	*Peperomia emarginella*	260	50	50	0
Peperomia emarginella	*Peperomia emarginella*	37	100	0	0
Peperomia emarginella	*Peperomia emarginella*	112	100	0	0
Peperomia emarginella	*Peperomia emarginella*	128	100	0	0
Peperomia emarginulata	*Peperomia emarginulata*	39	100	0	0
Peperomia emarginulata	*Peperomia emarginulata*	89	100	0	0
Peperomia emarginulata	*Peperomia emarginulata*	374	91	0	9
Peperomia emarginulata	*Peperomia emarginulata*	368	50	0	50
Peperomia emarginulata	*Peperomia emarginulata*	251	50	50	0
Peperomia emarginulata	*Peperomia emarginulata*	29	0	0	100
Peperomia emarginulata	*Peperomia emarginulata*	373	0	30	70
Peperomia emiliana	*Peperomia emiliana*	43	100	0	0
Peperomia emiliana	*Peperomia emiliana*	57	100	0	0
Peperomia emiliana	*Peperomia emiliana*	110	100	0	0
Peperomia emiliana	*Peperomia emiliana*	335	100	0	0
Peperomia emiliana	*Peperomia emiliana*	15	100	0	0
Peperomia emiliana	*Peperomia emiliana*	255	50	50	0
Peperomia emiliana	*Peperomia emiliana*	37	100	0	0
Peperomia emiliana	*Peperomia emiliana*	191	100	0	0
Peperomia emiliana	*Peperomia staminea*	328	100	0	0
Peperomia emiliana	*Peperomia staminea*	325	85	0	15
Peperomia emiliana	*Peperomia bocasensis*				
Peperomia endlichii	*Peperomia endlichii*	43	100	0	0
Peperomia endlichii	*Peperomia endlichii*	255	100	0	0

Peperomia enenyasensis	*Peperomia enenyasensis*	29	0	0	100
Peperomia enenyasensis	*Peperomia enenyasensis*	255	100	0	0
Peperomia enenyasensis	*Peperomia enenyasensis*	178	100	0	0
Peperomia enervis	*Peperomia enervis*	119	50	50	0
Peperomia enervis	*Peperomia johnsonii*				
Peperomia epidendron	*Peperomia epidendron*	43	50	0	50
Peperomia epidendron	*Peperomia epidendron*	110	100	0	0
Peperomia epidendron	*Peperomia epidendron*	255	50	0	50
Peperomia epilobioides	*Peperomia epilobioides*	373	0	0	100
Peperomia epipetrica	*Peperomia epipetrica*	43	0	50	50
Peperomia epipetrica	*Peperomia epipetrica*	255	0	50	50
Peperomia epipremnifolia	*Peperomia epipremnifolia*	51	0	0	100
Peperomia epipremnifolia	*Peperomia epipremnifolia*	82	0	0	100
Peperomia epipremnifolia	*Peperomia epipremnifolia*	222	30	0	70
Peperomia eripipunctulata	*Peperomia eripipunctulata*	397	100	0	0
Peperomia erosa	*Peperomia erosa*	247	0	50	50
Peperomia erythrocaulis	*Peperomia erythrocaulis*	201	0	0	100
Peperomia erythropremna	*Peperomia erythropremna*	294	0	50	50
Peperomia erythropremna	*Peperomia erythropremna*	255	100	0	0
Peperomia erythropremna	*Peperomia semidecurrens*	103	0	100	0
Peperomia erythrospicata	*Peperomia erythrospicata*	43	100	0	0
Peperomia erythrospicata	*Peperomia erythrospicata*	255	100	0	0
Peperomia erythrostachya	*Peperomia erythrostachya*	213	100	0	0
Peperomia erythrostachya	*Peperomia erythrostachya*	29	0	0	100
Peperomia erythrostachya	*Peperomia erythrostachya*	255	100	0	0
Peperomia erythrostachya	*Peperomia erythrostachya*	178	100	0	0
Peperomia erythrostachya	*Peperomia rubrispica*	29	0	0	100
Peperomia esmeraldana	*Peperomia esmeraldana*	373	0	0	100
Peperomia esmeraldana	*Peperomia esmeraldana*	255	100	0	0
Peperomia esperanzana	*Peperomia esperanzana*	43	100	0	0
Peperomia esperanzana	*Peperomia esperanzana*	219	100	0	0
Peperomia esperanzana	*Peperomia esperanzana*	255	50	0	50
Peperomia esperanzana	*Peperomia esperanzana*	37	85	0	15
Peperomia espinosae	*Peperomia espinosae*	248	0	0	100
Peperomia espinosae	*Peperomia espinosae*	194	0	100	0
Peperomia estaminea	*Peperomia estaminea*	255	100	0	0
Peperomia estrellana	*Peperomia estrellana*	29	100	0	0
Peperomia estrellana	*Peperomia estrellana*	255	100	0	0
Peperomia estrellana	*Peperomia estrellana*	178	100	0	0
Peperomia ewanii	*Peperomia ewanii*	373	0	0	100
Peperomia ewanii	*Peperomia rhombeo-ovata*	255	100	0	0
Peperomia exclamationis	*Peperomia exclamationis*	204	0	100	0
Peperomia exigua	*Peperomia exigua*	238	0	90	10
Peperomia exigua	*Peperomia exigua*	70	0	100	0
Peperomia exigua	*Peperomia exigua*	255	0	100	0
Peperomia exigua	*Peperomia hymenophylla*				
Peperomia exiguispica	*Peperomia exiguispica*	29	0	0	100
Peperomia exiguispica	*Peperomia exiguispica*	178	100	0	0
Peperomia exilamenta	*Peperomia exilamenta*	29	0	0	100
Peperomia exilamenta	*Peperomia exilamenta*	255	100	0	0
Peperomia exilamenta	*Peperomia exilamenta*	178	50	0	50
Peperomia exiliramea	*Peperomia exiliramea*	29	0	0	100
Peperomia exiliramea	*Peperomia exiliramea*	255	100	0	0
Peperomia exiliramea	*Peperomia exiliramea*	178	100	0	0
Peperomia expallescens	*Peperomia expallescens*	387	50	0	50
Peperomia expallescens	*Peperomia expallescens*	255	100	0	0
Peperomia fagerlindii	*Peperomia fagerlindii*	255	100	0	0
Peperomia falanana	*Peperomia falanana*	373	0	0	100
Peperomia falcata	*Peperomia falcata*	415	100	0	0
Peperomia falcata	*Peperomia falcata*	307	100	0	0
Peperomia falcata	*Peperomia falcata*	255	100	0	0
Peperomia falconensis	*Peperomia falconensis*	150	0	50	50
Peperomia falconensis	*Peperomia falconensis*	255	0	50	50
Peperomia falsa	*Peperomia falsa*	29	0	0	100
Peperomia falsa	*Peperomia falsa*	178	0	50	50
Peperomia famelica	*Peperomia famelica*	29	0	0	100
Peperomia famelica	*Peperomia famelica*	178	0	0	100
Peperomia farctifolia	*Peperomia farctifolia*	373	100	0	0
Peperomia farctifolia	*Peperomia farctifolia*	255	100	0	0
Peperomia fawcettii	*Peperomia fawcettii*	255	100	0	0
Peperomia fawcettii	*Peperomia fawcettii*	112	100	0	0
Peperomia fendleriana	*Peperomia fendleriana*	150	50	50	0
Peperomia fendleriana	*Peperomia fendleriana*	373	0	0	100
Peperomia fendleriana	*Peperomia fendleriana*	255	50	50	0
Peperomia fenzli	*Peperomia fenzli*				
Peperomia fernandeziana	*Peperomia fernandeziana*	412	50	0	50
Peperomia fernandeziana	*Peperomia fernandeziana*	255	50	0	50
Peperomia fernandeziana	*Peperomia fernandeziana*	306	33.3	33.3	33.4
Peperomia fernandopoiana	*Peperomia fernandopoiana*	54	50	0	50
Peperomia fernandopoiana	*Peperomia fernandopoiana*	144	100	0	0

Peperomia fernandopoiana	*Peperomia fernandopoiana*	384	85	0	15
Peperomia fernandopoiana	*Peperomia fernandopoiana*	91	70	30	0
Peperomia fernandopoiana	*Peperomia fernandopoiana*	255	100	0	0
Peperomia fernandopoiana	*Peperomia butaguensis*	1	0	0	100
Peperomia fernandopoiana	*Peperomia butaguensis*	16	50	50	0
Peperomia fernandopoiana	*Peperomia preussii*	256	100	0	0
Peperomia fernandopoiana	*Peperomia ruwenzoriensis*	270	100	0	0
Peperomia fernandopoiana	*Peperomia staudtii*	109	50	50	0
Peperomia fernandopoiana	*Peperomia crassifolia*				
Peperomia ferreyrae	*Peperomia ferreyrae*	29	50	50	0
Peperomia ferreyrae	*Peperomia ferreyrae*	195	0	50	50
Peperomia ferreyrae	*Peperomia ferreyrae*	247	0	50	50
Peperomia ficta	*Peperomia ficta*	29	0	0	100
Peperomia ficta	*Peperomia ficta*	255	100	0	0
Peperomia ficta	*Peperomia ficta*	178	100	0	0
Peperomia filicaulis	*Peperomia filicaulis*	43	100	0	0
Peperomia filicaulis	*Peperomia filicaulis*	255	100	0	0
Peperomia filiformis	*Peperomia filiformis*	29	100	0	0
Peperomia filiformis	*Peperomia filiformis*	255	100	0	0
Peperomia filiformis	*Peperomia filiformis*	112	50	0	50
Peperomia filiformis	*Peperomia brachystachya*				
Peperomia fissicola	*Peperomia fissicola*	29	0	0	100
Peperomia fissicola	*Peperomia fissicola*	178	0	100	0
Peperomia fissispica	*Peperomia fissispica*	43	100	0	0
Peperomia fissispica	*Peperomia fissispica*	219	100	0	0
Peperomia fissispica	*Peperomia fissispica*	255	100	0	0
Peperomia flabilis	*Peperomia flabilis*	29	0	0	100
Peperomia flabilis	*Peperomia flabilis*	255	100	0	0
Peperomia flavamenta	*Peperomia flavamenta*	29	0	0	100
Peperomia flavamenta	*Peperomia flavamenta*	255	100	0	0
Peperomia flavescens	*Peperomia flavescens*	29	100	0	0
Peperomia flavescens	*Peperomia flavescens*	29	0	0	100
Peperomia flavescens	*Peperomia flavescens*	29	0	0	100
Peperomia flavescentifolia	*Peperomia flavescentifolia*	29	0	0	100
Peperomia flavescentifolia	*Peperomia flavescentifolia*	255	100	0	0
Peperomia flavescentifolia	*Peperomia flavescentifolia*	178	100	0	0
Peperomia flavida	*Peperomia flavida*	307	100	0	0
Peperomia flavida	*Peperomia flavida*	255	100	0	0
Peperomia flexicaulis	*Peperomia flexicaulis*	51	100	0	0
Peperomia flexicaulis	*Peperomia flexicaulis*	82	100	0	0
Peperomia flexicaulis	*Peperomia flexicaulis*	255	100	0	0
Peperomia flexinervia	*Peperomia flexinervia*	43	50	0	50
Peperomia flexinervia	*Peperomia flexinervia*	255	50	0	50
Peperomia fluviatilis	*Peperomia fluviatilis*	51	100	0	0
Peperomia fluviatilis	*Peperomia fluviatilis*	82	100	0	0
Peperomia fluviatilis	*Peperomia fluviatilis*	255	100	0	0
Peperomia foliata	*Peperomia foliata*	29	0	0	100
Peperomia foliiflora	*Peperomia foliiflora*	29	0	0	100
Peperomia foliiflora	*Peperomia foliiflora*	283	0	100	0
Peperomia foliiflora	*Peperomia phyllantha*	203	0	0	100
Peperomia foliosa	*Peperomia foliosa*	373	0	0	100
Peperomia folsomii	*Peperomia folsomii*	43	100	0	0
Peperomia folsomii	*Peperomia folsomii*	255	100	0	0
Peperomia foraminum	*Peperomia foraminum*	72	0	100	0
Peperomia foraminum	*Peperomia perinduta*	231	0	100	0
Peperomia foraminum	*Peperomia corozosana*				
Peperomia fosbergii	*Peperomia fosbergii*	115	95	5	0
Peperomia fosbergii	*Peperomia fosbergii*	255	50	50	0
Peperomia fosbergii	*Peperomia tahitensis*				
Peperomia fournieri	*Peperomia fournieri*	43	0	34	66
Peperomia fournieri	*Peperomia fournieri*	255	100	0	0
Peperomia foveolata	*Peperomia foveolata*	255	50	50	0
Peperomia fragilis	*Peperomia fragilis*	373	0	0	100
Peperomia fragilis	*Peperomia fragilis*	255	100	0	0
Peperomia fragilissima	*Peperomia fragilissima*	29	0	0	100
Peperomia fragrans	*Peperomia fragrans*	150	0	100	0
Peperomia fragrans	*Peperomia fragrans*	373	0	50	50
Peperomia fragrans	*Peperomia binispica*	29	0	0	100
Peperomia franciscoi	*Peperomia franciscoi*	43	0	50	50
Peperomia franciscoi	*Peperomia franciscoi*	255	0	50	50
Peperomia fraseri	*Peperomia fraseri*	43	33	33	34
Peperomia fraseri	*Peperomia fraseri*	89	0	0	100
Peperomia fraseri	*Peperomia fraseri*	248	0	0	100
Peperomia fraseri	*Peperomia fraseri*	374	0	33.4	66.6
Peperomia fraseri	*Peperomia fraseri*	251	0	50	50
Peperomia fraseri	*Peperomia fraseri*	236	100	0	0
Peperomia fraseri	*Peperomia fraseri*	29	0	0	100
Peperomia fraseri	*Peperomia fraseri*	373	0	30	70
Peperomia fraseri	*Peperomia fraseri*	255	33.3	33.3	33.4
Peperomia fraseri	*Peperomia resediflora*				

Peperomia fruticetorum	*Peperomia fruticetorum*	374	30	0	70
Peperomia fruticetorum	*Peperomia fruticetorum*	373	0	0	100
Peperomia fuertesii	*Peperomia fuertesii*				
Peperomia fulvescens	*Peperomia fulvescens*	373	100	0	0
Peperomia fulvescens	*Peperomia fulvescens*	255	100	0	0
Peperomia fundacionensis	*Peperomia fundacionensis*	150	33.3	33.3	33.4
Peperomia fundacionensis	*Peperomia fundacionensis*	255	50	0	50
Peperomia fundacionensis	*Peperomia fundacionensis*	90	50	0	50
Peperomia fundus-oculi	*Peperomia fundus-oculi*	249	0	50	50
Peperomia furcata	*Peperomia furcata*	29	100	0	0
Peperomia fuscipunctata	*Peperomia fuscipunctata*	414	0	0	100
Peperomia fuscipunctata	*Peperomia fuscipunctata*	374	50	0	50
Peperomia fuscispica	*Peperomia fuscispica*	29	100	0	0
Peperomia fuscispica	*Peperomia fuscispica*	255	100	0	0
Peperomia fuscispica	*Peperomia fuscispica*	178	100	0	0
Peperomia gabinetensis	*Peperomia gabinetensis*	373	100	0	0
Peperomia gabinetensis	*Peperomia gabinetensis*	255	100	0	0
Peperomia galapagensis	*Peperomia galapagensis*	373	50	50	0
Peperomia galapagensis	*Peperomia galapagensis*	390	50	0	50
Peperomia galapagensis	*Peperomia galapagensis*	255	100	0	0
Peperomia galioides	*Peperomia galioides*	9	0	100	0
Peperomia galioides	*Peperomia galioides*	19	100	0	0
Peperomia galioides	*Peperomia galioides*	51	33	34	33
Peperomia galioides	*Peperomia galioides*	43	33.3	33.3	33.4
Peperomia galioides	*Peperomia galioides*	52	100	0	0
Peperomia galioides	*Peperomia galioides*	57	0	0	100
Peperomia galioides	*Peperomia galioides*	110	50	50	0
Peperomia galioides	*Peperomia galioides*	150	33.3	33.3	33.4
Peperomia galioides	*Peperomia galioides*	219	100	0	0
Peperomia galioides	*Peperomia galioides*	380	0	0	100
Peperomia galioides	*Peperomia galioides*	374	27.5	9	63.5
Peperomia galioides	*Peperomia galioides*	83	33.3	33.3	33.4
Peperomia galioides	*Peperomia galioides*	251	0	50	50
Peperomia galioides	*Peperomia galioides*	412	50	0	50
Peperomia galioides	*Peperomia galioides*	29	0	0	100
Peperomia galioides	*Peperomia galioides*	328	85	7.5	7.5
Peperomia galioides	*Peperomia galioides*	373	50	0	50
Peperomia galioides	*Peperomia galioides*	220	0	50	50
Peperomia galioides	*Peperomia galioides*	390	50	0	50
Peperomia galioides	*Peperomia galioides*	335	33.3	33.3	33.4
Peperomia galioides	*Peperomia galioides*	294	50	50	0
Peperomia galioides	*Peperomia galioides*	221	0	0	100
Peperomia galioides	*Peperomia galioides*	214	0	50	50
Peperomia galioides	*Peperomia galioides*	175	0	50	50
Peperomia galioides	*Peperomia galioides*	255	100	0	0
Peperomia galioides	*Peperomia galioides*	37	50	0	50
Peperomia galioides	*Peperomia galioides*	7	100	0	0
Peperomia galioides	*Peperomia melanosticta*	373	0	0	100
Peperomia galioides	*Peperomia menkeana*	82	100	0	0
Peperomia galioides	*Peperomia brachyiula*	29	0	0	100
Peperomia galioides	*Peperomia longispica*	29	0	0	100
Peperomia galioides	*Peperomia longispica*	177	0	100	0
Peperomia galioides	*Peperomia chillonensis*	29	0	0	100
Peperomia galioides	*Peperomia chillonensis*	178	0	50	50
Peperomia galioides	*Peperomia dendroides*	29	0	0	100
Peperomia galioides	*Peperomia dendromorphis*	29	0	0	100
Peperomia galioides	*Peperomia distractiflora*	29	0	0	100
Peperomia galioides	*Peperomia ceapanana*	29	0	0	100
Peperomia galioides	*Peperomia galioides*	90	33.3	33.3	33.4
Peperomia galioides	*Peperomia agapatensis*				
Peperomia galioides	*Peperomia amphoterophylla*				
Peperomia galioides	*Peperomia apoda*				
Peperomia galioides	*Peperomia oblongifolia*				
Peperomia galioides	*Peperomia okarana*				
Peperomia galioides	*Peperomia suaveolens*				
Peperomia galioides	*Peperomia galiifolia*				
Peperomia galioides	*Peperomia gallitoensis*				
Peperomia galioides	*Peperomia garrapatilla*				
Peperomia galioides	*Peperomia granata*				
Peperomia galioides	*Peperomia guayabillosana*				
Peperomia galioides	*Peperomia jamesonii*				
Peperomia galioides	*Peperomia redondoana*				
Peperomia galioides	*Peperomia subcorymbosa*				
Peperomia galioides	*Peperomia muscisedens*				
Peperomia galioides	*Peperomia trullifolia*				
Peperomia garcia-barrigana	*Peperomia garcia-barrigana*	373	0	0	100
Peperomia gardneriana	*Peperomia gardneriana*	51	0	100	0
Peperomia gardneriana	*Peperomia gardneriana*	373	0	0	100
Peperomia gardneriana	*Peperomia gardneriana*	113	0	50	50
Peperomia gaultheriifolia	*Peperomia gaultheriifolia*	373	0	0	100

Peperomia gaultheriifolia	*Peperomia gaultheriifolia*	255	100	0	0
Peperomia gayi	*Peperomia gayi*	29	0	0	100
Peperomia gayi	*Peperomia gayi*	255	100	0	0
Peperomia gedehana	*Peperomia gedehana*	78	100	0	0
Peperomia gehrigeri	*Peperomia gehrigeri*	150	0	0	100
Peperomia gehrigeri	*Peperomia gehrigeri*	373	50	0	50
Peperomia gehrigeri	*Peperomia gehrigeri*	255	100	0	0
Peperomia gemella	*Peperomia gemella*	255	100	0	0
Peperomia gemella	*Peperomia malaccensis*	376	0	100	0
Peperomia gemella	*Peperomia malaccensis*	272	95	5	0
Peperomia gemella	*Peperomia wallichii*				
Peperomia geminispica	*Peperomia geminispica*	43	50	50	0
Peperomia geminispica	*Peperomia geminispica*	373	100	0	0
Peperomia geminispica	*Peperomia geminispica*	255	50	50	0
Peperomia gentryi	*Peperomia gentryi*	150	0	0	100
Peperomia gentryi	*Peperomia gentryi*	198	0	0	100
Peperomia gerardoi	*Peperomia gerardoi*	43	0	100	0
Peperomia gerardoi	*Peperomia gerardoi*	255	0	100	0
Peperomia gibba	*Peperomia gibba*	29	0	0	100
Peperomia gigantea	*Peperomia gigantea*	292	0	0	100
Peperomia giralana	*Peperomia giralana*	198	0	0	100
Peperomia glabella	*Peperomia glabella*	18	100	0	0
Peperomia glabella	*Peperomia glabella*	24	100	0	0
Peperomia glabella	*Peperomia glabella*	51	100	0	0
Peperomia glabella	*Peperomia glabella*	43	100	0	0
Peperomia glabella	*Peperomia glabella*	56	100	0	0
Peperomia glabella	*Peperomia glabella*	57	0	0	100
Peperomia glabella	*Peperomia glabella*	60	100	0	0
Peperomia glabella	*Peperomia glabella*	110	90	10	0
Peperomia glabella	*Peperomia glabella*	150	50	50	0
Peperomia glabella	*Peperomia glabella*	154	100	0	0
Peperomia glabella	*Peperomia glabella*	213	50	0	50
Peperomia glabella	*Peperomia glabella*	227	100	0	0
Peperomia glabella	*Peperomia glabella*	374	75	0	25
Peperomia glabella	*Peperomia glabella*	21	100	0	0
Peperomia glabella	*Peperomia glabella*	83	100	0	0
Peperomia glabella	*Peperomia glabella*	368	0	0	100
Peperomia glabella	*Peperomia glabella*	273	50	50	0
Peperomia glabella	*Peperomia glabella*	172	33.3	33.3	33.4
Peperomia glabella	*Peperomia glabella*	29	100	0	0
Peperomia glabella	*Peperomia glabella*	259	50	50	0
Peperomia glabella	*Peperomia glabella*	328	100	0	0
Peperomia glabella	*Peperomia glabella*	373	50	0	50
Peperomia glabella	*Peperomia glabella*	220	100	0	0
Peperomia glabella	*Peperomia glabella*	171	50	50	0
Peperomia glabella	*Peperomia glabella*	381	100	0	0
Peperomia glabella	*Peperomia glabella*	335	100	0	0
Peperomia glabella	*Peperomia glabella*	15	100	0	0
Peperomia glabella	*Peperomia glabella*	294	33.3	33.3	33.4
Peperomia glabella	*Peperomia glabella*	153	100	0	0
Peperomia glabella	*Peperomia glabella*	136	100	0	0
Peperomia glabella	*Peperomia glabella*	214	100	0	0
Peperomia glabella	*Peperomia glabella*	42	100	0	0
Peperomia glabella	*Peperomia glabella*	85	0	0	100
Peperomia glabella	*Peperomia glabella*	293	100	0	0
Peperomia glabella	*Peperomia glabella*	260	50	50	0
Peperomia glabella	*Peperomia glabella*	255	50	50	0
Peperomia glabella	*Peperomia glabella*	37	85	0	15
Peperomia glabella	*Peperomia glabella*	32	50	50	0
Peperomia glabella	*Peperomia glabella*	112	0	100	0
Peperomia glabella	*Peperomia glabella*	191	50	0	50
Peperomia glabella	*Peperomia glabella*	33	0	100	0
Peperomia glabella	*Peperomia glabella*	128	100	0	0
Peperomia glabella	*Peperomia nigropunctata*	328	50	50	0
Peperomia glabella	*Peperomia nigropunctata*	153	50	0	50
Peperomia glabella	*Peperomia sintenisii*	260	50	0	50
Peperomia glabella	*Peperomia maleuvreana*	230	100	0	0
Peperomia glabella	*Peperomia longeacuminata*	325	50	50	0
Peperomia glabella	*Peperomia quadrivii*	370	100	0	0
Peperomia glabella	*Peperomia rubefacta*	408	0	0	100
Peperomia glabella	*Peperomia quiriguana*	176	100	0	0
Peperomia glabella	*Peperomia glabella*	90	95	5	0
Peperomia glabella	*Peperomia atropunctata*				
Peperomia glabella	*Peperomia linatifolia*				
Peperomia glabella	*Peperomia macorisiana*				
Peperomia glabella	*Peperomia pololensis*				
Peperomia glabella	*Peperomia yojoana*				
Peperomia glabella	*Peperomia cattii*				
Peperomia glabella	*Peperomia caulibarbis*				
Peperomia glabella	*Peperomia taylorii*				

Peperomia glabrilimba	*Peperomia glabrilimba*	71	100	0	0
Peperomia glabrior	*Peperomia glabrior*	211	50	0	50
Peperomia glabrirhachis	*Peperomia glabrirhachis*	29	0	0	100
Peperomia glabrirhachis	*Peperomia glabrirhachis*	255	100	0	0
Peperomia glandulosa	*Peperomia glandulosa*	373	0	0	100
Peperomia glandulosa	*Peperomia glandulosa*	255	100	0	0
Peperomia glareosa	*Peperomia glareosa*	29	0	0	100
Peperomia glareosa	*Peperomia glareosa*	255	100	0	0
Peperomia glareosa	*Peperomia glareosa*	191	100	0	0
Peperomia glassmanii	*Peperomia glassmanii*	198	100	0	0
Peperomia glassmanii	*Peperomia glassmanii*	149	75	25	0
Peperomia glassmanii	*Peperomia glassmanii*	118	100	0	0
Peperomia glassmanii	*Peperomia glassmanii*	255	100	0	0
Peperomia glauca	*Peperomia glauca*	246	0	50	50
Peperomia glauca	*Peperomia glauca*	255	0	50	50
Peperomia glazioui	*Peperomia glazioui*	51	50	50	0
Peperomia glazioui	*Peperomia glazioui*	273	50	50	0
Peperomia glazioui	*Peperomia glazioui*	82	50	50	0
Peperomia glazioui	*Peperomia glazioui*	220	50	50	0
Peperomia glazioui	*Peperomia glazioui*	221	100	0	0
Peperomia glazioui	*Peperomia glazioui*	156	50	50	0
Peperomia glazioui	*Peperomia glazioui*	255	100	0	0
Peperomia glazioui	*Peperomia glazioui*	128	100	0	0
Peperomia glazioui	*Peperomia glabripes*				
Peperomia gleicheniiformis	*Peperomia gleicheniiformis*	43	100	0	0
Peperomia gleicheniiformis	*Peperomia gleicheniiformis*	368	100	0	0
Peperomia gleicheniiformis	*Peperomia gleicheniiformis*	255	100	0	0
Peperomia gleicheniiformis	*Peperomia calimana*	373	100	0	0
Peperomia globosibacca	*Peperomia globosibacca*	255	0	50	50
Peperomia globulanthera	*Peperomia globulanthera*	387	50	0	50
Peperomia globulanthera	*Peperomia globulanthera*	255	100	0	0
Peperomia globulanthera	*Peperomia subnudilimba*	73	0	0	100
Peperomia globulifera	*Peperomia globulifera*				
Peperomia gloriosifolia	*Peperomia gloriosifolia*	96	100	0	0
Peperomia glutinosa	*Peperomia glutinosa*	328	70	15	15
Peperomia glutinosa	*Peperomia glutinosa*	176	100	0	0
Peperomia glutinosa	*Peperomia glutinosa*	215	0	50	50
Peperomia gorgonillana	*Peperomia gorgonillana*	43	100	0	0
Peperomia gorgonillana	*Peperomia gorgonillana*	374	100	0	0
Peperomia gorgonillana	*Peperomia gorgonillana*	373	100	0	0
Peperomia gorgonillana	*Peperomia gorgonillana*	255	100	0	0
Peperomia goudotii	*Peperomia goudotii*	255	50	50	0
Peperomia goudotii	*Peperomia penninervia*				
Peperomia gracieana	*Peperomia gracieana*	227	15	0	85
Peperomia gracieana	*Peperomia gracieana*	131	33.3	33.3	33.4
Peperomia gracieana	*Peperomia gracieana*	83	33.3	33.3	33.4
Peperomia gracieana	*Peperomia gracieana*	255	50	50	0
Peperomia gracilicaulis	*Peperomia gracilicaulis*	51	100	0	0
Peperomia gracilicaulis	*Peperomia gracilicaulis*	82	100	0	0
Peperomia gracilicaulis	*Peperomia gracilicaulis*	255	100	0	0
Peperomia gracilipeduncula	*Peperomia gracilipeduncula*	181	100	0	0
Peperomia gracilis	*Peperomia gracilis*	51	100	0	0
Peperomia gracilis	*Peperomia gracilis*	82	100	0	0
Peperomia gracilis	*Peperomia gracilis*	156	0	100	0
Peperomia gracilis	*Peperomia gracilis*	255	100	0	0
Peperomia gracilispica	*Peperomia gracilispica*	202	0	0	100
Peperomia gracillima	*Peperomia gracillima*	43	0	0	100
Peperomia gracillima	*Peperomia gracillima*	37	0	0	100
Peperomia grantii	*Peperomia grantii*	115	100	0	0
Peperomia grantii	*Peperomia grantii*	255	100	0	0
Peperomia granulata	*Peperomia granulata*	373	100	0	0
Peperomia granulata	*Peperomia granulata*	255	100	0	0
Peperomia granulatifolia	*Peperomia granulatifolia*	29	50	0	50
Peperomia granulatifolia	*Peperomia granulatifolia*	255	100	0	0
Peperomia granulatifolia	*Peperomia granulatifolia*	178	100	0	0
Peperomia granulatilimba	*Peperomia granulatilimba*	29	100	0	0
Peperomia granulatilimba	*Peperomia granulatilimba*	255	100	0	0
Peperomia granulatilimba	*Peperomia granulatilimba*	178	100	0	0
Peperomia granulosa	*Peperomia granulosa*	43	100	0	0
Peperomia granulosa	*Peperomia granulosa*	56	100	0	0
Peperomia granulosa	*Peperomia granulosa*	110	10	90	0
Peperomia granulosa	*Peperomia granulosa*	213	0	0	100
Peperomia granulosa	*Peperomia granulosa*	328	100	0	0
Peperomia granulosa	*Peperomia granulosa*	255	100	0	0
Peperomia granulosa	*Peperomia granulosa*	325	100	0	0
Peperomia granulosa	*Peperomia rio-cangrejalensis*	408	100	0	0
Peperomia granulosa	*Peperomia chicbulana*				
Peperomia granulosa	*Peperomia dantoana*	408	100	0	0
Peperomia granulosa	*Peperomia perplexa*	403	100	0	0
Peperomia graveolens	*Peperomia graveolens*	180	0	50	50

Peperomia grayumii	*Peperomia grayumii*	43	100	0	0
Peperomia grayumii	*Peperomia grayumii*	255	100	0	0
Peperomia griggsii	*Peperomia griggsii*	43	50	0	50
Peperomia griggsii	*Peperomia griggsii*	110	100	0	0
Peperomia griggsii	*Peperomia griggsii*	328	50	50	0
Peperomia griggsii	*Peperomia griggsii*	255	50	0	50
Peperomia grisarii	*Peperomia grisarii*	29	0	0	100
Peperomia grisarii	*Peperomia grisarii*	373	0	0	100
Peperomia grisebachii	*Peperomia grisebachii*	294	100	0	0
Peperomia grisebachii	*Peperomia bayajana*				
Peperomia grisebachii	*Peperomia obversa*				
Peperomia grisebachii	*Peperomia floridensis*				
Peperomia griseoargentea	*Peperomia griseoargentea*				
Peperomia gruendleri	*Peperomia gruendleri*	255	100	0	0
Peperomia guadaloupensis	*Peperomia piedrana*				
Peperomia guadaloupensis	*Peperomia guadaloupensis*	294	5	47.5	47.5
Peperomia guadaloupensis	*Peperomia guadaloupensis*	255	100	0	0
Peperomia guadaloupensis	*Peperomia guadaloupensis*	32	0	100	0
Peperomia guadaloupensis	*Peperomia monteverdensis*	94	0	100	0
Peperomia guadaloupensis	*Peperomia oblanceolata*	57	0	0	100
Peperomia guadaloupensis	*Peperomia cueroensis*	33	0	100	0
Peperomia guadalupana	*Peperomia guadalupana*	373	100	0	0
Peperomia guadalupana	*Peperomia guadalupana*	255	100	0	0
Peperomia guaiquinimana	*Peperomia guaiquinimana*	150	100	0	0
Peperomia guaiquinimana	*Peperomia guaiquinimana*	373	100	0	0
Peperomia guaiquinimana	*Peperomia guaiquinimana*	255	100	0	0
Peperomia guamana	*Peperomia guamana*	226	0	100	0
Peperomia guamana	*Peperomia saipana*				
Peperomia guamana	*Peperomia hoeferi*				
Peperomia guanensis	*Peperomia guanensis*	294	0	0	100
Peperomia guanensis	*Peperomia guanensis*	304	0	100	0
Peperomia guapilesiana	*Peperomia guapilesiana*	43	50	0	50
Peperomia guapilesiana	*Peperomia guapilesiana*	57	50	0	50
Peperomia guapilesiana	*Peperomia guapilesiana*	255	50	0	50
Peperomia guapilesiana	*Peperomia guapilesiana*	37	50	0	50
Peperomia guarujana	*Peperomia guarujana*	82	100	0	0
Peperomia guarujana	*Peperomia guarujana*	255	0	0	100
Peperomia guatemalensis	*Peperomia guatemalensis*	43	100	0	0
Peperomia guatemalensis	*Peperomia guatemalensis*	110	50	0	50
Peperomia guatemalensis	*Peperomia guatemalensis*	328	33.3	33.3	33.4
Peperomia guatemalensis	*Peperomia guatemalensis*	255	100	0	0
Peperomia guayrapurana	*Peperomia guayrapurana*	29	0	0	100
Peperomia guayrapurana	*Peperomia guayrapurana*	255	100	0	0
Peperomia gucayana	*Peperomia gucayana*	29	0	0	100
Peperomia gutierrezana	*Peperomia gutierrezana*	150	50	0	50
Peperomia gutierrezana	*Peperomia gutierrezana*	373	0	0	100
Peperomia gutierrezana	*Peperomia gutierrezana*	255	50	0	50
Peperomia gutierrezana	*Peperomia aguilae*	373	0	0	100
Peperomia guttulata	*Peperomia guttulata*	414	100	0	0
Peperomia guttulata	*Peperomia guttulata*	373	0	0	100
Peperomia guttulata	*Peperomia guttulata*	255	100	0	0
Peperomia gymnophylla	*Peperomia gymnophylla*	43	33	33	34
Peperomia gymnophylla	*Peperomia gymnophylla*	255	50	0	50
Peperomia hadrostachya	*Peperomia hadrostachya*	418	0	0	100
Peperomia haematolepis	*Peperomia haematolepis*	150	0	100	0
Peperomia haematolepis	*Peperomia haematolepis*	83	100	0	0
Peperomia haematolepis	*Peperomia haematolepis*	374	50	50	0
Peperomia haematolepis	*Peperomia haematolepis*	251	100	0	0
Peperomia haematolepis	*Peperomia haematolepis*	29	0	0	100
Peperomia haematolepis	*Peperomia haematolepis*	373	0	50	50
Peperomia haematolepis	*Peperomia haematolepis*	255	50	50	0
Peperomia haenkeana	*Peperomia haenkeana*	29	0	0	100
Peperomia haenkeana	*Phyllobryon haenkeanum*	203	0	0	100
Peperomia hallieri	*Peperomia hallieri*	78	100	0	0
Peperomia hamiltoniana	*Peperomia hamiltoniana*	29	0	0	100
Peperomia hamiltoniana	*Peperomia hamiltoniana*	32	0	100	0
Peperomia hamiltonianifolia	*Peperomia hamiltonianifolia*				
Peperomia hammelii	*Peperomia hammelii*	43	0	50	50
Peperomia hammelii	*Peperomia hammelii*	255	0	100	0
Peperomia hammelii	*Peperomia hammelii*	132	0	50	50
Peperomia hanaensis	*Peperomia hanaensis*	141	0	0	100
Peperomia harlingii	*Peperomia harlingii*	139	100	0	0
Peperomia harmandii	*Peperomia harmandii*	255	100	0	0
Peperomia harrisii	*Peperomia harrisii*	255	100	0	0
Peperomia harrisii	*Peperomia harrisii*	112	33.3	33.3	33.4
Peperomia hartmannii	*Peperomia hartmannii*	255	100	0	0
Peperomia hartwegiana	*Peperomia hartwegiana*	43	15	15	70
Peperomia hartwegiana	*Peperomia hartwegiana*	150	50	50	0
Peperomia hartwegiana	*Peperomia hartwegiana*	380	0	0	100
Peperomia hartwegiana	*Peperomia hartwegiana*	374	87.3	0	12.7

Peperomia hartwegiana	*Peperomia hartwegiana*	21	100	0	0
Peperomia hartwegiana	*Peperomia hartwegiana*	368	15	0	85
Peperomia hartwegiana	*Peperomia hartwegiana*	236	50	50	0
Peperomia hartwegiana	*Peperomia hartwegiana*	29	100	0	0
Peperomia hartwegiana	*Peperomia hartwegiana*	373	0	0	100
Peperomia hartwegiana	*Peperomia hartwegiana*	243	85	15	0
Peperomia hartwegiana	*Peperomia hartwegiana*	129	0	100	0
Peperomia hartwegiana	*Peperomia hartwegiana*	255	50	50	0
Peperomia hartwegiana	*Peperomia hartwegiana*	243	0	100	0
Peperomia haughtii	*Peperomia haughtii*	373	100	0	0
Peperomia haughtii	*Peperomia haughtii*	255	100	0	0
Peperomia haworthiana	*Peperomia hawarthii*				
Peperomia hebetata	*Peperomia hebetata*	373	100	0	0
Peperomia hebetata	*Peperomia hebetata*	255	100	0	0
Peperomia hedyotidea	*Peperomia hedyotidea*	255	100	0	0
Peperomia hedyotidea	*Peperomia angustilimba*				
Peperomia hedyotidea	*Peperomia linearifolia*				
Peperomia hemmendorffii	*Peperomia hemmendorffii*	51	0	0	100
Peperomia hemmendorffii	*Peperomia hemmendorffii*	82	0	0	100
Peperomia hemninieri	*Peperomia hemninieri*				
Peperomia hendersonensis	*Peperomia hendersonensis*	407	100	0	0
Peperomia heptaphylla	*Peperomia heptaphylla*	341	50	50	0
Peperomia heptaphylla	*Peperomia heptaphylla*	342	50	50	0
Peperomia heptaphylla	*Peperomia heptaphylla*	255	50	50	0
Peperomia hernandiifolia	*Peperomia hernandiifolia*	51	50	0	50
Peperomia hernandiifolia	*Peperomia hernandiifolia*	43	50	0	50
Peperomia hernandiifolia	*Peperomia hernandiifolia*	57	50	0	50
Peperomia hernandiifolia	*Peperomia hernandiifolia*	150	0	50	50
Peperomia hernandiifolia	*Peperomia hernandiifolia*	219	50	0	50
Peperomia hernandiifolia	*Peperomia hernandiifolia*	21	50	0	50
Peperomia hernandiifolia	*Peperomia hernandiifolia*	83	33.3	33.3	33.4
Peperomia hernandiifolia	*Peperomia hernandiifolia*	232	15	0	85
Peperomia hernandiifolia	*Peperomia hernandiifolia*	335	100	0	0
Peperomia hernandiifolia	*Peperomia hernandiifolia*	294	33.3	33.3	33.4
Peperomia hernandiifolia	*Peperomia hernandiifolia*	214	100	0	0
Peperomia hernandiifolia	*Peperomia hernandiifolia*	255	50	0	50
Peperomia hernandiifolia	*Peperomia hernandiifolia*	260	15	0	85
Peperomia hernandiifolia	*Peperomia hernandiifolia*	37	50	0	50
Peperomia hernandiifolia	*Peperomia hernandiifolia*	112	50	0	50
Peperomia hernandiifolia	*Peperomia conserta*	373	100	0	0
Peperomia hernandiifolia	*Peperomia ciliifera*				
Peperomia herrerae	*Peperomia herrerae*	29	0	0	100
Peperomia herzogii	*Peperomia herzogii*	412	100	0	0
Peperomia herzogii	*Peperomia herzogii*	255	100	0	0
Peperomia hesperomannii	*Peperomia hesperomannii*	387	15	0	85
Peperomia hesperomannii	*Peperomia hesperomannii*	255	50	0	50
Peperomia hesperomannii	*Peperomia hochreutineri*				
Peperomia hesperomannii	*Peperomia kauaiensis*				
Peperomia heterodoxa	*Peperomia heterodoxa*	43	0	25	75
Peperomia heterodoxa	*Peperomia heterodoxa*	110	90	10	0
Peperomia heterodoxa	*Peperomia heterodoxa*	255	100	0	0
Peperomia heterodoxa	*Peperomia heterodoxa*	382	0	50	50
Peperomia heterophylla	*Peperomia heterophylla*	43	50	0	50
Peperomia heterophylla	*Peperomia heterophylla*	57	100	0	0
Peperomia heterophylla	*Peperomia heterophylla*	110	100	0	0
Peperomia heterophylla	*Peperomia heterophylla*	150	50	50	0
Peperomia heterophylla	*Peperomia heterophylla*	219	0	0	100
Peperomia heterophylla	*Peperomia heterophylla*	374	83.3	0	16.7
Peperomia heterophylla	*Peperomia heterophylla*	29	100	0	0
Peperomia heterophylla	*Peperomia heterophylla*	335	100	0	0
Peperomia heterophylla	*Peperomia heterophylla*	255	50	0	50
Peperomia heterophylla	*Peperomia heterophylla*	178	100	0	0
Peperomia heterophylla	*Peperomia heterophylla*	191	100	0	0
Peperomia heterophylla	*Peperomia carrapana*	178	100	0	0
Peperomia heterophylla	*Peperomia aggravescens*	328	100	0	0
Peperomia heterophylla	*Peperomia rinconensis*	406	100	0	0
Peperomia heterophylla	*Peperomia vulcanicola*	373	0	0	100
Peperomia heterophylla	*Peperomia vulcanicola*	29	0	0	100
Peperomia heterophylla	*Peperomia dawsonii*				
Peperomia heterophylla	*Peperomia trumani*				
Peperomia heterostachya	*Peperomia heterostachya*	29	0	0	100
Peperomia heyneana	*Peperomia heyneana*	340	100	0	0
Peperomia heyneana	*Peperomia heyneana*	27	0	100	0
Peperomia heyneana	*Peperomia heyneana*	125	100	0	0
Peperomia heyneana	*Peperomia heyneana*	155	0	100	0
Peperomia heyneana	*Peperomia heyneana*	342	100	0	0
Peperomia heyneana	*Peperomia heyneana*	391	50	50	0
Peperomia heyneana	*Peperomia heyneana*	255	100	0	0
Peperomia heyneana	*Peperomia heyneana*	125	50	50	0
Peperomia heyneana	*Peperomia haycockii*				

Peperomia heyneana	*Peperomia wightiana*				
Peperomia heyneana	*Peperomia zeylanica*				
Peperomia heyneana	*Peperomia ceylanica*				
Peperomia heyneana	*Peperomia kurzii*				
Peperomia hilariana	*Peperomia hilariana*	51	34	33	33
Peperomia hilariana	*Peperomia hilariana*	80	0	100	0
Peperomia hilariana	*Peperomia hilariana*	220	0	50	50
Peperomia hilariana	*Peperomia hilariana*	179	0	0	100
Peperomia hilariana	*Peperomia hilariana*	85	33.3	33.3	33.4
Peperomia hilariana	*Peperomia hilariana*	161	15	0	85
Peperomia hilariana	*Peperomia apiahyna*				
Peperomia hildebrandtii	*Peperomia hildebrandtii*	255	100	0	0
Peperomia hintonii	*Peperomia hintonii*	293	15	85	0
Peperomia hintonii	*Peperomia hintonii*	279	100	0	0
Peperomia hirta	*Peperomia hirta*	31	100	0	0
Peperomia hirta	*Peperomia hirta*	43	45	10	45
Peperomia hirta	*Peperomia hirta*	57	50	0	50
Peperomia hirta	*Peperomia hirta*	110	90	10	0
Peperomia hirta	*Peperomia hirta*	219	50	0	50
Peperomia hirta	*Peperomia hirta*	335	90	5	5
Peperomia hirta	*Peperomia hirta*	294	0	0	100
Peperomia hirta	*Peperomia hirta*	255	50	0	50
Peperomia hirta	*Peperomia siguaneana*	104	0	100	0
Peperomia hirta	*Peperomia costaricensis*	15	50	0	50
Peperomia hirta	*Peperomia disparifolia*	326	100	0	0
Peperomia hirta	*Peperomia guttulatissima*				
Peperomia hirta	*Peperomia antillarum*				
Peperomia hirta	*Peperomia blepharilepida*				
Peperomia hirta	*Peperomia mollipubis*				
Peperomia hirta	*Peperomia tenuinervis*				
Peperomia hirta	*Peperomia fimbribractea*				
Peperomia hirta	*Peperomia nummularia*				
Peperomia hirtella	*Peperomia hirtella*	232	100	0	0
Peperomia hirtella	*Peperomia allorgeana*	198	100	0	0
Peperomia hirtella	*Peperomia subbracteiflora*	92	0	0	100
Peperomia hirtella	*Peperomia cataractigaudens*	332	50	0	50
Peperomia hirtella	*Peperomia belangeri*				
Peperomia hirtella	*Peperomia bracteiflora*				
Peperomia hirtella	*Peperomia subvillosa*				
Peperomia hirtella	*Peperomia cataractigaudens*				
Peperomia hirtella	*Peperomia dissitiflora*				
Peperomia hirtella	*Peperomia dussii*				
Peperomia hirtella	*Peperomia evadens*				
Peperomia hirtella	*Peperomia hahnii*				
Peperomia hirtellicaulis	*Peperomia hirtellicaulis*	373	0	0	100
Peperomia hirtellicaulis	*Peperomia hirtellicaulis*	255	100	0	0
Peperomia hirticaulis	*Peperomia hirticaulis*	78	100	0	0
Peperomia hirtipeduncula	*Peperomia hirtipeduncula*	373	0	0	100
Peperomia hirtipetiola	*Peperomia hirtipetiola*	73	0	0	100
Peperomia hirtipetiola	*Peperomia gracilescens*	73	0	0	100
Peperomia hirtipetiola	*Peperomia longilimba*	387	0	0	100
Peperomia hirtulicaulis	*Peperomia hirtulicaulis*	189	50	50	0
Peperomia hispidorhachis	*Peperomia hispidorhachis*	3	100	0	0
Peperomia hispidosa	*Peperomia hispidosa*	51	0	50	50
Peperomia hispidosa	*Peperomia hispidosa*	82	34	33	33
Peperomia hispidosa	*Peperomia hispidosa*	255	100	0	0
Peperomia hispidula	*Peperomia hispidula*	8	85	0	15
Peperomia hispidula	*Peperomia hispidula*	51	0	50	50
Peperomia hispidula	*Peperomia hispidula*	39	100	0	0
Peperomia hispidula	*Peperomia hispidula*	52	100	0	0
Peperomia hispidula	*Peperomia hispidula*	57	0	0	100
Peperomia hispidula	*Peperomia hispidula*	110	50	50	0
Peperomia hispidula	*Peperomia hispidula*	150	0	50	50
Peperomia hispidula	*Peperomia hispidula*	183	0	0	100
Peperomia hispidula	*Peperomia hispidula*	219	100	0	0
Peperomia hispidula	*Peperomia hispidula*	255	100	0	0
Peperomia hispidula	*Peperomia hispidula*	43	0	0	100
Peperomia hispidula	*Peperomia hispidula*	374	0	33.4	66.6
Peperomia hispidula	*Peperomia hispidula*	368	0	0	100
Peperomia hispidula	*Peperomia hispidula*	251	0	0	100
Peperomia hispidula	*Peperomia hispidula*	29	0	0	100
Peperomia hispidula	*Peperomia hispidula*	328	0	0	100
Peperomia hispidula	*Peperomia hispidula*	373	33.3	33.3	33.4
Peperomia hispidula	*Peperomia hispidula*	220	0	50	50
Peperomia hispidula	*Peperomia hispidula*	129	0	70	30
Peperomia hispidula	*Peperomia hispidula*	179	0	0	100
Peperomia hispidula	*Peperomia hispidula*	418	50	0	50
Peperomia hispidula	*Peperomia hispidula*	37	0	0	100
Peperomia hispidula	*Peperomia hispidula*	112	0	0	100
Peperomia hispidula	*Peperomia skutchii*	328	0	0	100

Peperomia hispidula	*Peperomia perhispidula*	29	0	0	100
Peperomia hispidula	*peperomia perhispidula*	178	0	50	50
Peperomia hispidula	*Peperomia barbensis*	28	100	0	0
Peperomia hispidula	*Peperomia hispidula*	90	0	0	100
Peperomia hispiduliformis	*Peperomia hispiduliformis*	110	33.3	33.3	33.4
Peperomia hispiduliformis	*Peperomia hispiduliformis*	213	0	0	100
Peperomia hispiduliformis	*Peperomia hispiduliformis*	255	50	0	50
Peperomia hispiduliformis	*Peperomia hispiduliformis*	412	50	0	50
Peperomia hispiduliformis	*Peperomia hispiduliformis*	29	0	0	100
Peperomia hispiduliformis	*Peperomia hispiduliformis*	293	50	50	0
Peperomia hispiduliformis	*Peperomia hispiduliformis*	418	50	0	50
Peperomia hobbitoides	*Peperomia hobbitoides*	110	10	90	0
Peperomia hobbitoides	*Peperomia hobbitoides*	389	0	100	0
Peperomia hodgei	*Peperomia hodgei*	43	0	50	50
Peperomia hodgei	*Peperomia hodgei*	255	50	0	50
Peperomia hodgei	*Peperomia hodgei*	373	0	0	100
Peperomia hoelscheri	*Peperomia hoelscheri*				
Peperomia hoffmannii	*Peperomia hoffmannii*	43	34	33	33
Peperomia hoffmannii	*Peperomia hoffmannii*	57	0	0	100
Peperomia hoffmannii	*Peperomia hoffmannii*	110	50	50	0
Peperomia hoffmannii	*Peperomia hoffmannii*	255	50	50	0
Peperomia hoffmannii	*Peperomia hoffmannii*	13	100	0	0
Peperomia hoffmannii	*Peperomia hoffmannii*	29	100	0	0
Peperomia hoffmannii	*Peperomia hoffmannii*	373	0	0	100
Peperomia hoffmannii	*Peperomia hoffmannii*	15	100	0	0
Peperomia hoffmannii	*Peperomia hoffmannii*	37	85	0	15
Peperomia hoffmannii	*Peperomia hoffmannii*	191	100	0	0
Peperomia hoffmannii	*Peperomia lindeniana*	52	100	0	0
Peperomia hoffmannii	*Peperomia lindeniana*	110	100	0	0
Peperomia hoffmannii	*Peperomia lindeniana*	255	100	0	0
Peperomia hoffmannii	*Peperomia obovata*				
Peperomia hombronii	*Peperomia hombronii*	115	95	5	0
Peperomia hombronii	*Peperomia hombronii*	255	50	50	0
Peperomia hombronii	*Peperomia setchellii*	303	50	50	0
Peperomia hondoana	*Peperomia hondoana*	328	0	100	0
Peperomia honigii	*Peperomia honigii*	150	0	0	100
Peperomia huacapistanana	*Peperomia huacapistanana*	178	100	0	0
Peperomia huacapistanana	*Peperomia erythrocorma*	29	0	0	100
Peperomia huallagana	*Peperomia huallagana*	255	100	0	0
Peperomia huallagana	*Peperomia huallagana*	29	100	0	0
Peperomia huallagana	*Peperomia huallagana*	178	100	0	0
Peperomia huallagana	*Peperomia huallagana*	29	0	0	100
Peperomia huantana	*Peperomia huantana*	29	0	0	100
Peperomia huantana	*Peperomia huantana*	29	0	0	100
Peperomia huanucoana	*Peperomia huanucoana*	29	0	50	50
Peperomia huatuscoana	*Peperomia huatuscoana*	110	50	50	0
Peperomia huberi	*Peperomia huberi*	51	0	0	100
Peperomia huberi	*Peperomia huberi*	82	0	0	100
Peperomia huberi	*Peperomia huberi*	42	100	0	0
Peperomia humbertii	*Peperomia humbertii*	199	50	0	50
Peperomia humbertii	*Peperomia humbertii*	255	100	0	0
Peperomia humifusa	*Peperomia humifusa*	255	100	0	0
Peperomia humilis	*Peperomia humilis*	43	0	0	100
Peperomia humilis	*Peperomia humilis*	328	50	0	50
Peperomia humilis	*Peperomia humilis*	153	0	0	100
Peperomia humilis	*Peperomia humilis*	32	0	100	0
Peperomia humilis	*Peperomia questeliana*	331	0	50	50
Peperomia humilis	*Peperomia ramosa*	102	0	0	100
Peperomia humilis	*Peperomia mornicola*				
Peperomia humilis	*Peperomia pilipeduncula*				
Peperomia humilis	*Peperomia hirsutifolia*				
Peperomia humilis	*Peperomia lentibacca*				
Peperomia hunteriana	*Peperomia hunteriana*	117	0	100	0
Peperomia hutchisonii	*Peperomia hutchisonii*	29	0	50	50
Peperomia hydnostachya	*Peperomia hydnostachya*	43	50	0	50
Peperomia hydrocotyloides	*Peperomia hydrocotyloides*	51	0	0	100
Peperomia hygrophiloides	*Peperomia hygrophiloides*	43	100	0	0
Peperomia hygrophiloides	*Peperomia hygrophiloides*	57	100	0	0
Peperomia hygrophiloides	*Peperomia hygrophiloides*	219	0	0	100
Peperomia hygrophiloides	*Peperomia hygrophiloides*	255	100	0	0
Peperomia hylophila	*Peperomia hylophila*	43	100	0	0
Peperomia hylophila	*Peperomia hylophila*	57	100	0	0
Peperomia hylophila	*Peperomia hylophila*	335	100	0	0
Peperomia hylophila	*Peperomia hylophila*	255	50	0	50
Peperomia hylophila	*Peperomia hylophila*	37	95	0	5
Peperomia hylophila	*Peperomia multifida*	317	100	0	0
Peperomia hylophila	*Peperomia porschiana*	372	100	0	0
Peperomia hylophila	*Peperomia cufodontii*				
Peperomia hylophila	*Peperomia fraijanesana*				
Peperomia hylophila	*Peperomia zurquiana*				

Peperomia hylophila	*Peperomia irazuana*				
Peperomia hypoleuca	*Peperomia hypoleuca*	387	50	0	50
Peperomia hypoleuca	*Peperomia hypoleuca*	255	100	0	0
Peperomia hypoleuca	*Peperomia nudipeduncula*				
Peperomia hypoleuca	*Peperomia pluvigaudens*				
Peperomia hyporhoda	*Peperomia hyporhoda*	29	0	0	100
Peperomia hyporhoda	*Peperomia hyporhoda*	255	100	0	0
Peperomia ibiramana	*Peperomia ibiramana*	51	34	33	33
Peperomia ibiramana	*Peperomia ibiramana*	82	33	33	34
Peperomia ilaloensis	*Peperomia ilaloensis*	150	33.3	33.3	33.4
Peperomia ilaloensis	*Peperomia ilaloensis*	374	100	0	0
Peperomia ilaloensis	*Peperomia ilaloensis*	368	0	0	100
Peperomia ilaloensis	*Peperomia ilaloensis*	373	0	0	100
Peperomia ilaloensis	*Peperomia ilaloensis*	255	50	0	50
Peperomia imbracteata	*Peperomia imbracteata*				
Peperomia imerinae	*Peperomia imerinae*	198	0	0	100
Peperomia immolata	*Peperomia immolata*	162	0	100	0
Peperomia inaequalifolia	*Peperomia inaequalifolia*	51	100	0	0
Peperomia inaequalifolia	*Peperomia inaequalifolia*	251	85	15	0
Peperomia inaequalifolia	*Peperomia inaequalifolia*	236	100	0	0
Peperomia inaequalifolia	*Peperomia inaequalifolia*	29	0	0	100
Peperomia inaequalifolia	*Peperomia inaequalifolia*	373	0	50	50
Peperomia inaequalifolia	*Peperomia inaequalifolia*	246	50	50	0
Peperomia inaequalifolia	*Peperomia inaequalifolia*	255	50	50	0
Peperomia inaequalifolia	*Peperomia atocongona*	29	0	0	100
Peperomia inaequalifolia	*Peperomia limaensis*	29	0	0	100
Peperomia inaequalifolia	*Peperomia pseudogalapagensis*	29	0	0	100
Peperomia inaequalifolia	*Peperomia ramulosa*	10	100	0	0
Peperomia inaequalifolia	*Peperomia snodgrassii*	68	100	0	0
Peperomia inaequalifolia	*Peperomia flagelliformis*				
Peperomia inaequalilimba	*Peperomia inaequalilimba*	418	0	0	100
Peperomia inaequilatera	*Peperomia inaequilatera*	29	0	0	100
Peperomia incana	*Peperomia incana*	51	50	50	0
Peperomia incana	*Peperomia incana*	82	50	50	0
Peperomia incana	*Peperomia incana*	84	0	100	0
Peperomia incana	*Peperomia incana*	85	33.3	33.4	33.3
Peperomia incana	*Peperomia incana*	11	50	50	0
Peperomia incisa	*Peperomia incisa*	43	100	0	0
Peperomia incisa	*Peperomia incisa*	255	100	0	0
Peperomia incisa	*Peperomia aneura*	373	100	0	0
Peperomia incognita	*Peperomia incognita*	43	0	0	100
Peperomia inconspicua	*Peperomia inconspicua*	373	0	0	100
Peperomia increscens	*Peperomia increscens*	183	15	42.5	42.5
Peperomia increscens	*Peperomia increscens*	255	50	0	50
Peperomia increscens	*Peperomia increscens*	214	100	0	0
Peperomia increscens	*Peperomia increscens*	418	50	0	50
Peperomia increscens	*Peperomia blanda var. pseudodindygulensis*	412	0	0	100
Peperomia increscens	*Peperomia saxigaudens*	142	0	100	0
Peperomia induratifolia	*Peperomia induratifolia*	29	0	0	100
Peperomia induratifolia	*Peperomia induratifolia*	255	100	0	0
Peperomia infralutea	*Peperomia infralutea*	373	100	0	0
Peperomia infralutea	*Peperomia infralutea*	255	100	0	0
Peperomia infravillosa	*Peperomia infravillosa*	229	0	100	0
Peperomia infravillosa	*Peperomia montazosana*				
Peperomia inquilina	*Peperomia inquilina*	255	100	0	0
Peperomia insueta	*Peperomia insueta*	43	0	33	67
Peperomia insueta	*Peperomia insueta*	57	0	0	100
Peperomia inversa	*Peperomia inversa*	210	0	100	0
Peperomia involucrata	*Peperomia involucrata*	373	0	0	100
Peperomia irrasa	*Peperomia irrasa*	208	0	0	100
Peperomia itatiaiana	*Peperomia itatiaiana*	82	50	50	0
Peperomia itayana	*Peperomia itayana*	29	0	0	100
Peperomia jalcaensis	*Peperomia jalcaensis*	292	0	0	100
Peperomia jamaicana	*Peperomia jamaicana*				
Peperomia jamesoniana	*Peperomia jamesoniana*	39	100	0	0
Peperomia jamesoniana	*Peperomia jamesoniana*	43	100	0	0
Peperomia jamesoniana	*Peperomia jamesoniana*	150	50	0	50
Peperomia jamesoniana	*Peperomia jamesoniana*	219	100	0	0
Peperomia jamesoniana	*Peperomia jamesoniana*	374	94	0	6
Peperomia jamesoniana	*Peperomia jamesoniana*	21	100	0	0
Peperomia jamesoniana	*Peperomia jamesoniana*	373	100	0	0
Peperomia jamesoniana	*Peperomia jamesoniana*	381	100	0	0
Peperomia jamesoniana	*Peperomia jamesoniana*	255	100	0	0
Peperomia jamesoniana	*Peperomia chimantana*	182	100	0	0
Peperomia jamesoniana	*Peperomia chimantana*	414	100	0	0
Peperomia jamesoniana	*Peperomia pseudojamesoniana*				
Peperomia jamesoniana	*Peperomia demissa*				
Peperomia japonica	*Peperomia japonica*	174	50	50	0

Peperomia japonica	*Peperomia okinawensis*	394	0	100	0
Peperomia jaraguana	*Peperomia jaraguana*				
Peperomia jarisiana	*Peperomia darisiana*				
Peperomia josei	*Peperomia josei*	29	100	0	0
Peperomia josei	*Peperomia josei*	373	100	0	0
Peperomia josei	*Peperomia josei*	255	100	0	0
Peperomia junghuhniana	*Peperomia junghuhniana*				
Peperomia juniniana	*Peperomia juniniana*	374	0	0	100
Peperomia juniniana	*Peperomia juniniana*	29	0	0	100
Peperomia juniniana	*Peperomia juniniana*	191	0	0	100
Peperomia juniniana	*Peperomia juniniana*	284	0	100	0
Peperomia juruana	*Peperomia juruana*	51	100	0	0
Peperomia juruana	*Peperomia juruana*	42	100	0	0
Peperomia juruana	*Peperomia juruana*	255	100	0	0
Peperomia kalimatina	*Peperomia kalimatina*	255	100	0	0
Peperomia kamerunana	*Peperomia kamerunana*	54	50	0	50
Peperomia kamerunana	*Peperomia kamerunana*	91	100	0	0
Peperomia kamerunana	*Peperomia kamerunana*	255	100	0	0
Peperomia kanalensis	*Peperomia kanalensis*	296	0	100	0
Peperomia kimnachii	*Peperomia kimnachii*	255	100	0	0
Peperomia kipahuluensis	*Peperomia kipahuluensis*	387	85	0	15
Peperomia kipahuluensis	*Peperomia kipahuluensis*	255	100	0	0
Peperomia kjellii	*Peperomia kjellii*	198	0	0	100
Peperomia kjellii	*Peperomia kjellii*	203	0	0	100
Peperomia klopfensteinii	*Peperomia klopfensteinii*	292	0	0	100
Peperomia klotzschiana	*Peperomia klotzschiana*	29	0	0	100
Peperomia klotzschiana	*Peperomia klotzschiana*	255	100	0	0
Peperomia klugiana	*Peperomia klugiana*	373	100	0	0
Peperomia klugiana	*Peperomia klugiana*	255	100	0	0
Peperomia kokeana	*Peperomia kokeana*	387	0	0	100
Peperomia kotana	*Peperomia kotana*	376	0	100	0
Peperomia kotana	*Peperomia kotana*	272	0	100	0
Peperomia kotana	*Peperomia kotana*	342	100	0	0
Peperomia kotana	*Peperomia kotana*	255	100	0	0
Peperomia kraemeri	*Peperomia kraemeri*	149	0	100	0
Peperomia kraemeri	*Peperomia tiniannensis*				
Peperomia kravangensis	*Peperomia kravangensis*	217	100	0	0
Peperomia kuhliana	*Peperomia kuhliana*				
Peperomia kuntzei	*Peperomia kuntzei*	357	0	0	100
Peperomia kusaiensis	*Peperomia kusaiensis*	149	100	0	0
Peperomia kusaiensis	*Peperomia kusaiensis*	118	100	0	0
Peperomia kusaiensis	*Peperomia kusaiensis*	255	100	0	0
Peperomia laeteviridis	*Peperomia laeteviridis*	91	100	0	0
Peperomia laevifolia	*Peperomia laevifolia*	340	50	50	0
Peperomia laevifolia	*Peperomia laevifolia*	12	33.3	33.3	33.4
Peperomia laevifolia	*Peperomia laevifolia*	342	50	50	0
Peperomia laevifolia	*Peperomia laevifolia*	255	50	50	0
Peperomia laevifolia	*Peperomia apoana*	198	100	0	0
Peperomia laevifolia	*Peperomia bilineata*				
Peperomia laevifolia	*Peperomia nativitatis*	79	50	50	0
Peperomia laevilimba	*Peperomia laevilimba*	307	15	85	0
Peperomia laevilimba	*Peperomia laevilimba*	255	50	50	0
Peperomia lagunaensis	*Peperomia lagunaensis*	238	33.3	33.3	33.4
Peperomia lagunaensis	*Peperomia lagunaensis*	255	100	0	0
Peperomia lanaoensis	*Peperomia lanaoensis*	238	0	100	0
Peperomia lanceolata	*Peperomia lanceolata*	43	0	50	50
Peperomia lanceolata	*Peperomia lanceolata*	219	50	0	50
Peperomia lanceolata	*Peperomia lanceolata*	373	0	0	100
Peperomia lanceolata	*Peperomia lanceolata*	255	0	50	50
Peperomia lanceolata	*Peperomia barbinodis*	57	0	0	100
Peperomia lanceolata	*Peperomia barbinodis*	37	50	0	50
Peperomia lanceolatopeltata	*Peperomia boomii*	339	0	100	0
Peperomia lanceolatopeltata	*Peperomia molithrix*	328	50	50	0
Peperomia lanceolatopeltata	*Peperomia muelleri*	110	100	0	0
Peperomia lanceolatopeltata	*Peperomia lanceolatopeltata*	338	0	100	0
Peperomia lanceolatopeltata	*Peperomia lanceolatopeltata*	43	34	33	33
Peperomia lanceolatopeltata	*Peperomia lanceolatopeltata*	57	100	0	0
Peperomia lanceolatopeltata	*Peperomia lanceolatopeltata*	110	50	50	0
Peperomia lanceolatopeltata	*Peperomia lanceolatopeltata*	150	0	100	0
Peperomia lanceolatopeltata	*Peperomia lanceolatopeltata*	51	0	50	50
Peperomia lanceolatopeltata	*Peperomia lanceolatopeltata*	29	100	0	0
Peperomia lanceolatopeltata	*Peperomia lanceolatopeltata*	373	0	50	50
Peperomia lanceolatopeltata	*Peperomia lanceolatopeltata*	335	5	47.5	47.5
Peperomia lanceolatopeltata	*Peperomia lanceolatopeltata*	113	0	100	0
Peperomia lanceolatopeltata	*Peperomia lanceolatopeltata*	255	100	0	0
Peperomia lanceolatopeltata	*Peperomia lanceolatopeltata*	37	33.3	33.3	33.4
Peperomia lanceolatapeltata	*Peperomia tecticola*				
Peperomia lanceolatapeltata	*Peperomia chambesyana*				
Peperomia lanceolatapeltata	*Peperomia tuberosa*				
Peperomia lancifolia	*Peperomia lancifolia*	51	0	0	100

Peperomia lancifolia	*Peperomia lancifolia*	43	33.3	33.3	33.4
Peperomia lancifolia	*Peperomia lancifolia*	57	0	0	100
Peperomia lancifolia	*Peperomia lancifolia*	110	33.3	33.3	33.4
Peperomia lancifolia	*Peperomia lancifolia*	150	33.3	33.3	33.4
Peperomia lancifolia	*Peperomia lancifolia*	213	0	0	100
Peperomia lancifolia	*Peperomia lancifolia*	219	50	0	50
Peperomia lancifolia	*Peperomia lancifolia*	374	38.4	0	61.6
Peperomia lancifolia	*Peperomia lancifolia*	21	100	0	0
Peperomia lancifolia	*Peperomia lancifolia*	83	33.3	33.3	33.4
Peperomia lancifolia	*Peperomia lancifolia*	29	100	0	0
Peperomia lancifolia	*Peperomia lancifolia*	373	0	0	100
Peperomia lancifolia	*Peperomia lancifolia*	214	0	0	100
Peperomia lancifolia	*Peperomia lancifolia*	255	50	0	50
Peperomia lancifolia	*Peperomia lancifolia*	37	0	0	100
Peperomia lancifolia	*Peperomia lancifolia*	191	0	0	100
Peperomia lancifolia	*Peperomia floribunda*	328	15	15	70
Peperomia lancifolia	*Peperomia macrophylla*				
Peperomia lancifolia	*Peperomia miradoresiana*				
Peperomia lancifolia	*Peperomia erasmia*				
Peperomia lancifolia	*Peperomia lancilimba*				
Peperomia lanosa	*Peperomia lanosa*	29	0	0	100
Peperomia lanuginosa	*Peperomia lanuginosa*	241	0	0	100
Peperomia lasierrana	*Peperomia lasierrana*	373	0	0	100
Peperomia lasiophylla	*Peperomia lasiophylla*	255	100	0	0
Peperomia lasiorhachis	*Peperomia lasiorhachis*	255	100	0	0
Peperomia lasiostigma	*Peperomia lasiostigma*	409	80	20	0
Peperomia lasiostigma	*Peperomia lasiostigma*	307	85	15	0
Peperomia lasiostigma	*Peperomia lasiostigma*	255	50	0	50
Peperomia lasiostigma	*Peperomia carnosa*				
Peperomia lasiostigma	*Peperomia gibbsiae*				
Peperomia lasiostigma	*Peperomia kandavuana*	409	50	50	0
Peperomia latibracteata	*Peperomia latibracteata*	255	100	0	0
Peperomia latifolia	*Peperomia latifolia*	255	100	0	0
Peperomia latifolia	*Peperomia latifolia*	387	0	50	50
Peperomia latifolia	*Peperomia waihoiana*				
Peperomia latifolia	*Peperomia dentulibractea*				
Peperomia latifolia	*Peperomia punaluuna*				
Peperomia latifolia	*Peperomia villipeduncula*				
Peperomia latilimba	*Peperomia latilimba*	373	0	0	100
Peperomia latimerana	*Peperomia latimerana*	255	100	0	0
Peperomia lauterbachii	*Peperomia lauterbachii*	255	100	0	0
Peperomia lawrancei	*Peperomia lawrancei*	373	100	0	0
Peperomia lawrancei	*Peperomia lawrancei*	255	100	0	0
Peperomia laxiflora	*Peperomia laxiflora*	57	0	0	100
Peperomia laxiflora	*Peperomia laxiflora*	374	0	0	100
Peperomia laxiflora	*Peperomia laxiflora*	373	0	0	100
Peperomia ledermannii	*Peperomia ledermannii*	255	100	0	0
Peperomia lehmannii	*Peperomia lehmannii*	373	0	0	100
Peperomia lenticularis	*Peperomia lenticularis*	328	100	0	0
Peperomia lenticularis	*Peperomia lenticularis*	15	100	0	0
Peperomia lenticularis	*Peperomia lenticularis*	408	100	0	0
Peperomia leptophylla	*Peperomia leptophylla*	110	50	50	0
Peperomia leptophylla	*Peperomia leptophylla*	293	50	50	0
Peperomia leptophylla	*Peperomia oajacensis*	170	100	0	0
Peperomia leptostachya	*Peperomia leptostachya*	388	50	50	0
Peperomia leptostachya	*Peperomia leptostachya*	409	33	67	0
Peperomia leptostachya	*Peperomia leptostachya*	118	85	15	0
Peperomia leptostachya	*Peperomia leptostachya*	387	5	95	0
Peperomia leptostachya	*Peperomia leptostachya*	307	15	85	0
Peperomia leptostachya	*Peperomia leptostachya*	81	0	100	0
Peperomia leptostachya	*Peperomia arabica*	1	10	45	45
Peperomia leptostachya	*Peperomia arabica*	16	0	45	55
Peperomia leptostachya	*Peperomia dindygulensis*	125	50	50	0
Peperomia leptostachya	*Peperomia brachytrichoides*				
Peperomia leptostachya	*Peperomia moerenhoutii*				
Peperomia leptostachya	*Peperomia thwaitesii*				
Peperomia leptostachya	*Peperomia candollei*				
Peperomia leptostachya	*Peperomia insularum*				
Peperomia leptostachya	*Peperomia kyimbilana*				
Peperomia leptostachya	*Peperomia bequaertii*				
Peperomia leptostachyoides	*Peperomia leptostachyoides*	255	100	0	0
Peperomia leucanthera	*Peperomia leucanthera*	373	0	0	100
Peperomia leucanthera	*Peperomia leucanthera*	255	100	0	0
Peperomia leucorrhachis	*Peperomia leucorrhachis*	373	0	0	100
Peperomia leucostachya	*Peperomia leucostachya*	373	0	0	100
Peperomia lewisii	*Peperomia lewisii*				
Peperomia liclicensis	*Peperomia liclicensis*	198	0	100	0
Peperomia liclicensis	*Peperomia liclicensis*	242	0	100	0
Peperomia liebmannii	*Peperomia liebmannii*	43	0	50	50
Peperomia liebmannii	*Peperomia liebmannii*	110	10	90	0

Peperomia liebmannii	*Peperomia liebmannii*	255	100	0	0
Peperomia liebmannii	*Peperomia liebmannii*	328	0	100	0
Peperomia liesneri	*Peperomia liesneri*	150	0	50	50
Peperomia liesneri	*Peperomia liesneri*	255	100	0	0
Peperomia lifuana	*Peperomia lifuana*	297	0	100	0
Peperomia lignescens	*Peperomia lignescens*	43	34	33	33
Peperomia lignescens	*Peperomia lignescens*	110	50	50	0
Peperomia lignescens	*Peperomia lignescens*	150	0	100	0
Peperomia lignescens	*Peperomia lignescens*	219	50	0	50
Peperomia lignescens	*Peperomia lignescens*	255	0	100	0
Peperomia lignescens	*Peperomia lignescens*	37	15	0	85
Peperomia lignescens	*Peperomia carlosiana*				
Peperomia lignescens	*Peperomia carthaginensis*				
Peperomia lignescens	*Peperomia jilotepequeana*	328	0	0	100
Peperomia ligustrina	*Peperomia ligustrina*	387	0	100	0
Peperomia lilliputiana	*Peperomia lilliputiana*	292	0	0	100
Peperomia limana	*Peperomia limana*	43	0	0	100
Peperomia limana	*Peperomia limana*	328	50	0	50
Peperomia linaresii	*Peperomia linaresii*	43	0	50	50
Peperomia linaresii	*Peperomia linaresii*	255	0	50	50
Peperomia lindmaniana	*Peperomia lindmaniana*	82	0	0	100
Peperomia litana	*Peperomia litana*				
Peperomia llewelynii	*Peperomia llewelynii*	373	0	0	100
Peperomia loefgrenii	*Peperomia loefgrenii*	51	0	0	100
Peperomia loefgrenii	*Peperomia loefgrenii*	82	0	0	100
Peperomia lonchophylloides	*Peperomia lonchophylloides*	267	100	0	0
Peperomia longepedunculata	*Peperomia longepedunculata*	29	0	0	100
Peperomia longibacca	*Peperomia longibacca*	364	100	0	0
Peperomia longifolia	*Peperomia multiplex*				
Peperomia longipetiolata	*Peperomia longipetiolata*	373	0	0	100
Peperomia longipila	*Peperomia longipila*	29	0	0	100
Peperomia longisetosa	*Peperomia longisetosa*	43	0	0	100
Peperomia lorentzii	*Peperomia lorentzii*	412	100	0	0
Peperomia lorentzii	*Peperomia lorentzii*	418	100	0	0
Peperomia lorentzii	*Peperomia lorentzii*	255	100	0	0
Peperomia loucoubeana	*Peperomia loucoubeana*	255	100	0	0
Peperomia loxensis	*Peperomia loxensis*	51	100	0	0
Peperomia loxensis	*Peperomia loxensis*	43	100	0	0
Peperomia loxensis	*Peperomia loxensis*	150	50	50	0
Peperomia loxensis	*Peperomia loxensis*	21	100	0	0
Peperomia loxensis	*Peperomia loxensis*	373	0	0	100
Peperomia loxensis	*Peperomia loxensis*	47	50	0	50
Peperomia loxensis	*Peperomia loxensis*	175	100	0	0
Peperomia loxensis	*Peperomia loxensis*	255	50	50	0
Peperomia luisana	*Peperomia luisana*	328	50	50	0
Peperomia lyallii	*Peperomia lyallii*	255	100	0	0
Peperomia lyman-smithii	*Peperomia lyman-smithii*	51	0	0	100
Peperomia lyman-smithii	*Peperomia lyman-smithii*	82	0	0	100
Peperomia macbrideana	*Peperomia macbrideana*	29	0	0	100
Peperomia macraeana	*Peperomia macraeana*	387	5	0	95
Peperomia macraeana	*Peperomia macraeana*	255	100	0	0
Peperomia macraeana	*Peperomia astigmata*				
Peperomia macraeana	*Peperomia longirama*				
Peperomia macraeana	*Peperomia molokaiensis*				
Peperomia macraeana	*Peperomia nudilimba*				
Peperomia macraeana	*Peperomia nudipetiola*				
Peperomia macraeana	*Peperomia pachycaulis*				
Peperomia macraeana	*Peperomia subnudipetiola*				
Peperomia macraeana	*Peperomia psilostigma*				
Peperomia macrandra	*Peperomia macrandra*	257	0	100	0
Peperomia macrocarpa	*Peperomia macrocarpa*				
Peperomia macrorhiza	*Peperomia macrorhiza*	29	0	0	100
Peperomia macrorhiza	*Peperomia macrorhiza*	386	0	100	0
Peperomia macrorhiza	*Peperomia macrorhiza*	244	0	100	0
Peperomia macrorostrum	*Peperomia macrorostrum*	43	100	0	0
Peperomia macrorostrum	*Peperomia macrorostrum*	255	100	0	0
Peperomia macrostachyos	*Peperomia macrostachyos*	39	100	0	0
Peperomia macrostachyos	*Peperomia macrostachyos*	43	50	50	0
Peperomia macrostachyos	*Peperomia macrostachyos*	57	0	0	100
Peperomia macrostachyos	*Peperomia macrostachyos*	60	100	0	0
Peperomia macrostachyos	*Peperomia macrostachyos*	89	100	0	0
Peperomia macrostachyos	*Peperomia macrostachyos*	150	50	0	50
Peperomia macrostachyos	*Peperomia macrostachyos*	227	100	0	0
Peperomia macrostachyos	*Peperomia macrostachyos*	271	100	0	0
Peperomia macrostachyos	*Peperomia macrostachyos*	83	100	0	0
Peperomia macrostachyos	*Peperomia macrostachyos*	116	7.5	7.5	85
Peperomia macrostachyos	*Peperomia macrostachyos*	29	100	0	0
Peperomia macrostachyos	*Peperomia macrostachyos*	373	60	0	40
Peperomia macrostachyos	*Peperomia macrostachyos*	381	100	0	0
Peperomia macrostachyos	*Peperomia macrostachyos*	86	50	0	50

Peperomia macrostachyos	*Peperomia macrostachyos*	335	100	0	0
Peperomia macrostachyos	*Peperomia macrostachyos*	15	100	0	0
Peperomia macrostachyos	*Peperomia macrostachyos*	223	100	0	0
Peperomia macrostachyos	*Peperomia macrostachyos*	214	100	0	0
Peperomia macrostachyos	*Peperomia macrostachyos*	42	100	0	0
Peperomia macrostachyos	*Peperomia macrostachyos*	37	100	0	0
Peperomia macrostachyos	*Peperomia macrostachyos*	191	100	0	0
Peperomia macrostachyos	*Peperomia macrostachyos*	276	100	0	0
Peperomia macrostachyos	*Peperomia macrostachyos*	51	100	0	0
Peperomia macrostachyos	*Peperomia macrostachyos*	255	100	0	0
Peperomia macrostachyos	*Peperomia apodostachya*	412	95	0	5
Peperomia macrostachyos	*Peperomia ciliatifolia var. iquitosana*	29	0	0	100
Peperomia macrostachyos	*Peperomia quatrometralis*	29	0	0	100
Peperomia macrostachyos	*Peperomia semisupina*	29	0	0	100
Peperomia macrostachyos	*peperomia quatrometralis*	178	100	0	0
Peperomia macrostachyos	*Peperomia oblongibacca*	254	100	0	0
Peperomia macrostachyos	*Peperomia pendula*	365	100	0	0
Peperomia macrostachyos	*Peperomia rupestris*	128	50	50	0
Peperomia macrostachyos	*Peperomia nematostachya*	42	100	0	0
Peperomia macrostachyos	*Peperomia stenocarpa*	156	0	100	0
Peperomia macrostachyos	*Peperomia myosuroides*				
Peperomia macrostachyos	*Peperomia nemostachya*				
Peperomia macrostachyos	*Peperomia circumscissa*				
Peperomia macrostachyos	*Peperomia cylindribacca*				
Peperomia macrostachyos	*Peperomia defluens*				
Peperomia macrostachyos	*Peperomia glaberrima*				
Peperomia macrostachyos	*Peperomia glabricaulis*				
Peperomia macrostachyos	*Peperomia glabriramea*				
Peperomia macrothyrsa	*Peperomia macrothyrsa*	29	0	0	100
Peperomia macrotricha	*Peperomia macrotricha*	57	0	0	100
Peperomia macrotricha	*Peperomia macrotricha*	373	0	0	100
Peperomia maculosa	*Peperomia maculosa*	43	100	0	0
Peperomia maculosa	*Peperomia maculosa*	57	0	0	100
Peperomia maculosa	*Peperomia maculosa*	110	10	45	45
Peperomia maculosa	*Peperomia maculosa*	150	0	50	50
Peperomia maculosa	*Peperomia maculosa*	219	100	0	0
Peperomia maculosa	*Peperomia maculosa*	374	50	0	50
Peperomia maculosa	*Peperomia maculosa*	29	0	0	100
Peperomia maculosa	*Peperomia maculosa*	328	70	20	10
Peperomia maculosa	*Peperomia maculosa*	373	0	0	100
Peperomia maculosa	*Peperomia maculosa*	335	100	0	0
Peperomia maculosa	*Peperomia maculosa*	294	5	80	15
Peperomia maculosa	*Peperomia maculosa*	260	50	0	50
Peperomia maculosa	*Peperomia maculosa*	255	33.3	33.3	33.4
Peperomia maculosa	*Peperomia maculosa*	37	15	0	85
Peperomia maculosa	*Peperomia maculosa*	112	0	85	15
Peperomia maculosa	*Peperomia maculosa*	90	0	50	50
Peperomia maculosa	*Peperomia tenebraegaudens*				
Peperomia maculosa	*Peperomia variegata*				
Peperomia maculosa	*Peperomia leridana*				
Peperomia maculosa	*Peperomia parmata*				
Peperomia maculosa	*Peperomia suavis*				
Peperomia madagascariensis	*Peperomia madagascariensis*				
Peperomia maestrana	*Peperomia maestrana*	294	0	0	100
Peperomia maestrana	*Peperomia maestrana*	255	100	0	0
Peperomia magnifoliiflora	*Peperomia magnifoliiflora*	203	0	0	100
Peperomia magnoliifolia	*Peperomia magnoliifolia*	51	100	0	0
Peperomia magnoliifolia	*Peperomia magnoliifolia*	43	50	50	0
Peperomia magnoliifolia	*Peperomia magnoliifolia*	57	0	0	100
Peperomia magnoliifolia	*Peperomia magnoliifolia*	110	50	0	50
Peperomia magnoliifolia	*Peperomia magnoliifolia*	150	50	50	0
Peperomia magnoliifolia	*Peperomia magnoliifolia*	374	71.4	0	28.6
Peperomia magnoliifolia	*Peperomia magnoliifolia*	21	100	0	0
Peperomia magnoliifolia	*Peperomia magnoliifolia*	83	33.3	33.3	33.4
Peperomia magnoliifolia	*Peperomia magnoliifolia*	172	33.3	33.3	33.4
Peperomia magnoliifolia	*Peperomia magnoliifolia*	259	0	50	50
Peperomia magnoliifolia	*Peperomia magnoliifolia*	29	100	0	0
Peperomia magnoliifolia	*Peperomia magnoliifolia*	373	33.3	33.3	33.4
Peperomia magnoliifolia	*Peperomia magnoliifolia*	171	50	50	0
Peperomia magnoliifolia	*Peperomia magnoliifolia*	86	47.5	47.5	5
Peperomia magnoliifolia	*Peperomia magnoliifolia*	335	100	0	0
Peperomia magnoliifolia	*Peperomia magnoliifolia*	294	33.3	33.3	33.4
Peperomia magnoliifolia	*Peperomia magnoliifolia*	223	50	50	0
Peperomia magnoliifolia	*Peperomia magnoliifolia*	153	5	0	95
Peperomia magnoliifolia	*Peperomia magnoliifolia*	42	100	0	0
Peperomia magnoliifolia	*Peperomia magnoliifolia*	293	50	50	0
Peperomia magnoliifolia	*Peperomia magnoliifolia*	224	50	50	0
Peperomia magnoliifolia	*Peperomia magnoliifolia*	255	33.3	33.3	33.4
Peperomia magnoliifolia	*Peperomia martini*	176	0	100	0

Peperomia magnoliifolia	*Peperomia pustulatibacca*	333	0	100	0
Peperomia magnoliifolia	*Peperomia romaensis*	408	0	0	100
Peperomia magnoliifolia	*Peperomia contraria*				
Peperomia magnoliifolia	*Peperomia conulifera*				
Peperomia magnoliifolia	*Peperomia petenensis*				
Peperomia magnoliifolia	*Peperomia pseudoamplexicaulis*				
Peperomia magnoliifolia	*Peperomia subamplexicaulis*				
Peperomia maguirei	*Peperomia maguirei*	227	100	0	0
Peperomia maguirei	*Peperomia maguirei*	83	50	50	0
Peperomia maguirei	*Peperomia maguirei*	255	50	50	0
Peperomia maguirei	*Peperomia maguirei*	373	100	0	0
Peperomia maguirei	*Peperomia maguirei*	261	100	0	0
Peperomia maijeri	*Peperomia maijeri*	247	0	50	50
Peperomia majalis	*Peperomia majalis*	29	0	0	100
Peperomia majalis	*Peperomia majalis*	178	100	0	0
Peperomia mameiana	*Peperomia mameiana*	43	0	33	67
Peperomia mameiana	*Peperomia mameiana*	60	50	50	0
Peperomia mameiana	*Peperomia mameiana*	255	33.3	33.3	33.4
Peperomia mameiana	*Peperomia mameiana*	37	50	0	50
Peperomia manabina	*Peperomia manabina*	374	25	0	75
Peperomia manabina	*Peperomia manabina*	373	0	0	100
Peperomia manarae	*Peperomia manarae*	150	0	0	100
Peperomia mandioccana	*Peperomia mandioccana*	51	50	50	0
Peperomia mandioccana	*Peperomia mandioccana*	82	50	50	0
Peperomia mandioccana	*Peperomia mandioccana*	160	0	0	100
Peperomia mandioccana	*Peperomia mandioccana*	255	50	50	0
Peperomia mandioccana	*Peperomia mandioccana*	220	0	100	0
Peperomia mandioccana	*Peperomia mandioccana*	221	50	50	0
Peperomia mangalbaria	*Peperomia mangalbaria*	263	50	50	0
Peperomia mantadiana	*Peperomia mantadiana*	199	0	0	100
Peperomia mantaroana	*Peperomia mantaroana*	247	0	50	50
Peperomia mantiquerana	*Peperomia mantiquerana*				
Peperomia mapulehuana	*Peperomia mapulehuana*				
Peperomia marahuacensis	*Peperomia marahuacensis*	150	0	50	50
Peperomia marahuacensis	*Peperomia marahuacensis*	255	0	50	50
Peperomia maransara	*Peperomia maransara*	29	0	0	100
Peperomia marcapatana	*Peperomia marcapatana*	29	0	0	100
Peperomia marcapatana	*Peperomia marcapatana*	255	100	0	0
Peperomia marcapatana	*Peperomia marcapatana*	358	0	100	0
Peperomia marchionensis	*Peperomia marchionensis*	388	33	33	34
Peperomia marchionensis	*Peperomia marchionensis*	115	33	33	34
Peperomia marchionensis	*Peperomia marchionensis*	255	50	50	0
Peperomia marchionensis	*Peperomia wilderi*				
Peperomia marcoana	*Peperomia marcoana*	51	0	100	0
Peperomia marcoana	*Peperomia marcoana*	82	0	100	0
Peperomia marcoana	*Peperomia marcoana*	255	100	0	0
Peperomia margaritifera	*Peperomia margaritifera*	412	0	0	100
Peperomia margaritifera	*Peperomia margaritifera*	306	0	90	10
Peperomia mariannensis	*Peperomia mariannensis*	118	50	0	50
Peperomia mariannensis	*Peperomia mariannensis*	255	100	0	0
Peperomia mariannensis	*Peperomia ladronica*				
Peperomia marivelesana	*Peperomia marivelesana*	70	100	0	0
Peperomia marivelesana	*Peperomia marivelesana*	238	50	50	0
Peperomia marivelesana	*Peperomia marivelesana*	255	100	0	0
Peperomia marmorata	*Peperomia marmorata*	29	0	0	100
Peperomia marshalliana	*Peperomia marshalliana*	29	0	0	100
Peperomia marshalliana	*Peperomia marshalliana*	255	100	0	0
Peperomia martiana	*Peperomia martiana*	18	100	0	0
Peperomia martiana	*Peperomia martiana*	24	100	0	0
Peperomia martiana	*Peperomia martiana*	51	50	50	0
Peperomia martiana	*Peperomia martiana*	43	70	30	0
Peperomia martiana	*Peperomia martiana*	57	100	0	0
Peperomia martiana	*Peperomia martiana*	110	100	0	0
Peperomia martiana	*Peperomia martiana*	150	50	50	0
Peperomia martiana	*Peperomia martiana*	374	75	0	25
Peperomia martiana	*Peperomia martiana*	29	100	0	0
Peperomia martiana	*Peperomia martiana*	373	0	0	100
Peperomia martiana	*Peperomia martiana*	220	50	50	0
Peperomia martiana	*Peperomia martiana*	335	100	0	0
Peperomia martiana	*Peperomia martiana*	179	50	0	50
Peperomia martiana	*Peperomia martiana*	221	50	50	0
Peperomia martiana	*Peperomia martiana*	418	50	0	50
Peperomia martiana	*Peperomia martiana*	161	50	0	50
Peperomia martiana	*Peperomia martiana*	255	50	50	0
Peperomia martiana	*Peperomia martiana*	252	50	50	0
Peperomia martiana	*Peperomia reptabunda*	37	50	0	50
Peperomia martiana	*Peperomia erythrophlebia*				
Peperomia martiana	*Peperomia defrenata*				
Peperomia masuthoniana	*Peperomia masuthoniana*	341	50	50	0

Peperomia masuthoniana	*Peperomia masuthoniana*	342	50	50	0
Peperomia masuthoniana	*Peperomia masuthoniana*	255	100	0	0
Peperomia mathewsiana	*Peperomia chachapoyasensis*	191	0	0	100
Peperomia mathewsiana	*Peperomia chachapoyasensis*	29	0	0	100
Peperomia mathewsiana	*Peperomia mathewsii*				
Peperomia mathieui	*Peperomia mathieui*	247	0	50	50
Peperomia matlalucaensis	*Peperomia matlalucaensis*	43	100	0	0
Peperomia matlalucaensis	*Peperomia matlalucaensis*	57	100	0	0
Peperomia matlalucaensis	*Peperomia matlalucaensis*	110	100	0	0
Peperomia matlalucaensis	*Peperomia matlalucaensis*	219	100	0	0
Peperomia matlalucaensis	*Peperomia matlalucaensis*	335	100	0	0
Peperomia matlalucaensis	*Peperomia punctatifolia*	321	100	0	0
Peperomia mauiensis	*Peperomia mahanana*	73	100	0	0
Peperomia mauiensis	*Peperomia mahanana*	255	100	0	0
Peperomia mauiensis	*Peperomia mahanana*	387	50	50	0
Peperomia mauiensis	*Peperomia maniensis*				
Peperomia maxonii	*Peperomia maxonii*	212	100	0	0
Peperomia maxwellana	*Peperomia maxwellana*	159	0	50	50
Peperomia maxwellana	*Peperomia maxwellana*	376	0	100	0
Peperomia maypurensis	*Peperomia maypurensis*	57	0	50	50
Peperomia maypurensis	*Peperomia maypurensis*	29	0	0	100
Peperomia maypurensis	*Peperomia maypurensis*	373	100	0	0
Peperomia maypurensis	*Peperomia maypurensis*	255	100	0	0
Peperomia maypurensis	*Peperomia ornata*	413	0	50	50
Peperomia meeboldii	*Peperomia meeboldii*	74	100	0	0
Peperomia megalepis	*Peperomia megalepis*	29	0	0	100
Peperomia megalepis	*Peperomia megalepis*	255	100	0	0
Peperomia megalepis	*Peperomia megalepis*	178	100	0	0
Peperomia megalopoda	*Peperomia megalopoda*	172	0	100	0
Peperomia megalopoda	*Peperomia megalopoda*	171	0	100	0
Peperomia megapotamica	*Peperomia megapotamica*	51	0	50	50
Peperomia megapotamica	*Peperomia megapotamica*	82	0	50	50
Peperomia megapotamica	*Peperomia megapotamica*	220	0	50	50
Peperomia megapotamica	*Peperomia mourae*	228	100	0	0
Peperomia melanokirrocarpa	*Peperomia melanokirrocarpa*	255	100	0	0
Peperomia melanosticta	*Peperomia melanosticta*	308	50	0	50
Peperomia melinii	*Peperomia melinii*	255	100	0	0
Peperomia membranacea	*Peperomia membranacea*	387	33.3	33.3	33.4
Peperomia membranacea	*Peperomia membranacea*	255	100	0	0
Peperomia membranacea	*Peperomia plinervata*				
Peperomia membranacea	*Peperomia waipioana*				
Peperomia menkeana	*Peperomia menkeana*	218	100	0	0
Peperomia mercedana	*Peperomia mercedana*	29	0	0	100
Peperomia mercedana	*Peperomia mercedana*	255	100	0	0
Peperomia mercedana	*Peperomia mercedana*	29	0	0	100
Peperomia meridana	*Peperomia meridana*	150	0	0	100
Peperomia meridana	*Peperomia meridana*	255	100	0	0
Peperomia merrillii	*Peperomia merrillii*	238	0	100	0
Peperomia mesitasana	*Peperomia mesitasana*	373	0	0	100
Peperomia metallica	*Peperomia metallica*	29	0	0	100
Peperomia metcalfii	*Peperomia metcalfii*	373	0	50	50
Peperomia mexicana	*Peperomia mexicana*	43	0	50	50
Peperomia mexicana	*Peperomia mexicana*	328	0	70	30
Peperomia mexicana	*Peperomia mexicana*	255	0	50	50
Peperomia mexicana	*Peperomia galeottiana*	110	10	90	0
Peperomia microlepis	*Peperomia microlepis*	29	0	0	100
Peperomia microlepis	*Peperomia microlepis*	255	100	0	0
Peperomia micromamillata	*Peperomia micromamillata*	29	0	0	100
Peperomia micromerioides	*Peperomia micromerioides*	373	0	0	100
Peperomia micromerioides	*Peperomia micromerioides*	255	100	0	0
Peperomia microphylla	*Peperomia microphylla*	43	0	0	100
Peperomia microphylla	*Peperomia microphylla*	150	0	50	50
Peperomia microphylla	*Peperomia microphylla*	380	0	0	100
Peperomia microphylla	*Peperomia microphylla*	374	50	12.5	37.5
Peperomia microphylla	*Peperomia microphylla*	368	50	0	50
Peperomia microphylla	*Peperomia microphylla*	251	85	15	0
Peperomia microphylla	*Peperomia microphylla*	29	0	0	100
Peperomia microphylla	*Peperomia microphylla*	373	85	15	0
Peperomia microphylla	*Peperomia microphylla*	255	100	0	0
Peperomia microphylla	*Peperomia gilberti*	29	0	0	100
Peperomia microphyllophora	*Peperomia microphyllophora*	150	0	0	100
Peperomia microphyllophora	*Peperomia microphyllophora*	373	0	0	100
Peperomia microstachya	*Peperomia microstachya*	301	100	0	0
Peperomia millei	*Peperomia millei*	373	0	0	100
Peperomia millei	*Peperomia millei*	255	100	0	0
Peperomia mindoroensis	*Peperomia mindoroensis*	238	100	0	0
Peperomia minensis	*Peperomia minensis*	51	100	0	0
Peperomia minensis	*Peperomia minensis*	82	100	0	0
Peperomia minensis	*Peperomia minensis*	255	100	0	0
Peperomia minuta	*Peperomia minuta*	29	0	0	100

Peperomia miqueliana	*Peperomia miqueliana*	374	0	0	100
Peperomia miqueliana	*Peperomia cordilimba*	373	0	0	100
Peperomia miqueliana	*Peperomia crassilimba*	373	0	0	100
Peperomia mishuyacana	*Peperomia mishuyacana*	29	0	0	100
Peperomia mishuyacana	*Peperomia mishuyacana*	255	100	0	0
Peperomia mishuyacana	*Peperomia mishuyacana*	191	50	0	50
Peperomia mishuyacana	*Peperomia mishuyacana*	276	100	0	0
Peperomia mitchelioides	*Peperomia mitchelioides*	373	0	0	100
Peperomia mitoensis	*Peperomia mitoensis*	247	0	50	50
Peperomia mixtifolia	*Peperomia mixtifolia*	43	0	0	100
Peperomia mocoana	*Peperomia mocoana*	373	0	10	90
Peperomia mocquerysii	*Peperomia mocquerysii*	255	100	0	0
Peperomia modicilimba	*Peperomia modicilimba*	29	0	0	100
Peperomia modicilimba	*Peperomia modicilimba*	178	0	100	0
Peperomia molleri	*Peperomia molleri*	144	15	0	85
Peperomia molleri	*Peperomia molleri*	384	15	15	70
Peperomia molleri	*Peperomia molleri*	91	30	0	70
Peperomia molleri	*Peperomia molleri*	255	50	0	50
Peperomia molleri	*Peperomia molleri*	88	0	0	100
Peperomia molleri	*Peperomia holstii*	16	50	0	50
Peperomia molleri	*Peperomia baumannii*				
Peperomia molleri	*Peperomia magilensis*				
Peperomia molleri	*Peperomia stolzii*				
Peperomia molleri	*Peperomia zenkeri*				
Peperomia molleri	*Peperomia winkleri*				
Peperomia mollicaulis	*Peperomia mollicaulis*				
Peperomia mollis	*Peperomia mollis*	57	100	0	0
Peperomia mollis	*Peperomia mollis*	219	100	0	0
Peperomia mollis	*Peperomia mollis*	374	100	0	0
Peperomia mollis	*Peperomia mollis*	21	100	0	0
Peperomia mollis	*Peperomia mollis*	373	100	0	0
Peperomia mollis	*Peperomia mollis*	255	100	0	0
Peperomia mollisoides	*Peperomia mollisoides*	150	50	0	50
Peperomia mollisoides	*Peperomia mollisoides*	373	50	0	50
Peperomia mollisoides	*Peperomia mollisoides*	255	100	0	0
Peperomia mollisoides	*Peperomia mollisoides*	90	50	0	50
Peperomia monostachya	*Peperomia monostachya*	29	0	0	100
Peperomia montana	*Peperomia montana*	29	0	0	100
Peperomia montecristana	*Peperomia montecristana*	43	100	0	0
Peperomia montecristana	*Peperomia montecristana*	57	100	0	0
Peperomia montecristana	*Peperomia montecristana*	335	100	0	0
Peperomia montecristana	*Peperomia montecristana*	255	100	0	0
Peperomia montecristana	*Peperomia montecristana*	37	100	0	0
Peperomia monticola	*Peperomia monticola*	124	0	100	0
Peperomia moralesii	*Peperomia moralesii*	43	0	0	100
Peperomia moreliana	*Peperomia moreliana*	255	100	0	0
Peperomia mosenii	*Peperomia mosenii*	255	100	0	0
Peperomia moulmeiniana	*Peperomia moulmeiniana*	340	100	0	0
Peperomia moulmeiniana	*Peperomia moulmeiniana*	342	100	0	0
Peperomia moulmeiniana	*Peperomia moulmeiniana*	255	100	0	0
Peperomia moyobambana	*Peperomia moyobambana*	255	100	0	0
Peperomia multifolia	*Peperomia multifolia*	45	0	0	100
Peperomia multiformis	*Peperomia multiformis*	29	0	50	50
Peperomia multiformis	*Peperomia multiformis*	255	100	0	0
Peperomia multispica	*Peperomia multispica*				
Peperomia multisurcula	*Peperomia multisurcula*	341	50	50	0
Peperomia multisurcula	*Peperomia multisurcula*	342	50	50	0
Peperomia multisurcula	*Peperomia multisurcula*	255	50	50	0
Peperomia muscicola	*Peperomia muscicola*	255	100	0	0
Peperomia muscigaudens	*Peperomia muscigaudens*	29	100	0	0
Peperomia muscigaudens	*Peperomia muscigaudens*	255	100	0	0
Peperomia muscigaudens	*Peperomia muscigaudens*	178	100	0	0
Peperomia muscipara	*Peperomia muscipara*				
Peperomia mutilata	*Peperomia mutilata*	255	100	0	0
Peperomia myrtifolia	*Peperomia myrtifolia*	172	0	100	0
Peperomia myrtifolia	*Peperomia myrtifolia*	171	0	100	0
Peperomia myrtifolia	*Peperomia myrtifolia*	153	0	0	100
Peperomia myrtifolia	*Peperomia myrtifolia*	173	0	100	0
Peperomia myrtifolia	*Peperomia auberyana*	198	0	50	50
Peperomia myrtifolia	*Peperomia broadwayi*	93	0	0	100
Peperomia myrtifolia	*Peperomia rupertiana*	32	0	50	50
Peperomia myrtifolia	*Peperomia doleana*	198	100	0	0
Peperomia myrtifolia	*Peperomia persuccosa*	77	0	100	0
Peperomia myrtifolia	*Peperomia barthelemyana*				
Peperomia myrtifolia	*Peperomia boldinghii*				
Peperomia myrtifolia	*Peperomia dolosa*				
Peperomia myrtifolia	*Peperomia dolosa*				
Peperomia myrtifolia	*Peperomia vanheurckii*				
Peperomia naevifolia	*Peperomia naevifolia*	29	0	0	100
Peperomia naevifolia	*Peperomia naevifolia*	178	0	0	100

Peperomia naitasiriensis	*Peperomia naitasiriensis*	409	100	0	0
Peperomia naitasiriensis	*Peperomia naitasiriensis*	307	100	0	0
Peperomia naitasiriensis	*Peperomia naitasiriensis*	255	100	0	0
Peperomia nakaharae	*Peperomia nakaharae*	340	100	0	0
Peperomia nakaharae	*Peperomia nakaharae*	342	100	0	0
Peperomia nakaharae	*Peperomia nakaharae*	255	100	0	0
Peperomia namosiana	*Peperomia namosiana*	415	100	0	0
Peperomia namosiana	*Peperomia namosiana*	307	100	0	0
Peperomia namosiana	*Peperomia namosiana*	255	100	0	0
Peperomia nandalana	*Peperomia nandalana*	409	0	100	0
Peperomia nandalana	*Peperomia nandalana*	307	0	100	0
Peperomia nandalana	*Peperomia nandalana*	255	0	100	0
Peperomia nandalana	*Peperomia nandalana*	307	50	50	0
Peperomia nandarivatensis	*Peperomia nandarivatensis*	409	0	100	0
Peperomia nandarivatensis	*Peperomia nandarivatensis*	307	0	100	0
Peperomia nandarivatensis	*Peperomia nandarivatensis*	255	0	100	0
Peperomia naranjoana	*Peperomia naranjoana*	43	50	50	0
Peperomia naranjoana	*Peperomia naranjoana*	255	50	50	0
Peperomia naranjoana	*Peperomia luisana*	328	50	50	0
Peperomia naranjoana	*Peperomia sisiana*	328	100	0	0
Peperomia naranjoana	*Peperomia orientalis*	318	100	0	0
Peperomia naranjoana	*Peperomia floresensis*	110	100	0	0
Peperomia naranjoana	*Peperomia floresensis*	328	33.3	33.3	33.4
Peperomia naranjoana	*Peperomia floresensis*	408	100	0	0
Peperomia naranjoana	*Peperomia siziana*				
Peperomia naranjoana	*Peperomia tilarana*				
Peperomia naviculifolia	*Peperomia naviculifolia*	29	0	0	100
Peperomia naviculifolia	*Peperomia naviculifolia*	286	0	100	0
Peperomia neblinana	*Peperomia neblinana*	150	100	0	0
Peperomia neblinana	*Peperomia neblinana*	255	100	0	0
Peperomia negrosensis	*Peperomia negrosensis*	70	0	100	0
Peperomia negrosensis	*Peperomia negrosensis*	238	0	100	0
Peperomia negrosensis	*Peperomia negrosensis*	255	100	0	0
Peperomia nequejahuirana	*Peperomia nequejahuirana*	255	100	0	0
Peperomia nervosovenosa	*Peperomia nervosovenosa*				
Peperomia nicolliae	*Peperomia nicolliae*	200	50	50	0
Peperomia nicolliae	*Peperomia nicolliae*	255	100	0	0
Peperomia nicoyana	*Peperomia nicoyana*				
Peperomia nigricans	*Peperomia nigricans*	29	0	0	100
Peperomia nigro-oculata	*Peperomia nigro-oculata*	29	100	0	0
Peperomia nigro-oculata	*Peperomia nigro-oculata*	255	100	0	0
Peperomia nigropunctata	*Peperomia nigropunctata*	383	100	0	0
Peperomia nigropunctata	*Peperomia balineorum*				
Peperomia nigropunctata	*Peperomia martinicensis*	330	100	0	0
Peperomia nigropunctata	*Peperomia nigrescens*	331	100	0	0
Peperomia nigropunctata	*Peperomia palpebrata*	331	100	0	0
Peperomia nigropunctata	*Peperomia stehleana*	329	100	0	0
Peperomia nigropunctata	*Peperomia houelmonte*				
Peperomia nigropunctata	*Peperomia thionvilleana*				
Peperomia nigropunctata	*Peperomia wilsonii*	198	100	0	0
Peperomia nigro-ungulata	*Peperomia nigro-ungulata*	373	100	0	0
Peperomia nigro-ungulata	*Peperomia nigro-ungulata*	255	100	0	0
Peperomia nitida	*Peperomia nitida*	24	100	0	0
Peperomia nitida	*Peperomia nitida*	51	33.3	33.4	33.3
Peperomia nitida	*Peperomia nitida*	21	100	0	0
Peperomia nitida	*Peperomia nitida*	273	50	50	0
Peperomia nitida	*Peperomia nitida*	85	50	50	0
Peperomia nitida	*Peperomia nitida*	417	0	50	50
Peperomia nitida	*Peperomia nitida*	418	0	50	50
Peperomia nitida	*Peperomia nitida*	252	33.3	33.3	33.4
Peperomia nivalis	*Peperomia nivalis*	29	0	0	100
Peperomia nivalis	*Peperomia nivalis*	246	0	50	50
Peperomia nivalis	*Peperomia nivalis*	243	0	50	50
Peperomia nivalis	*Peperomia nivalis*	255	0	50	50
Peperomia nivalis	*Peperomia nivalis*	242	0	10	90
Peperomia nivalis	*Peperomia nivalis*	243	0	0	100
Peperomia nivalis	*Peperomia lepadiphylla*	29	0	0	100
Peperomia nizaitoensis	*Peperomia amphioxys*	107	0	100	0
Peperomia nizaitoensis	*Peperomia moncionis*	97	0	0	100
Peperomia nodosa	*Peperomia nodosa*	307	0	0	100
Peperomia non-alata	*Peperomia non-alata*	29	0	0	100
Peperomia nopalana	*Peperomia nopalana*	110	100	0	0
Peperomia nopalana	*Peperomia nopalana*	157	100	0	0
Peperomia nopalana	*Peperomia nopalana*	255	100	0	0
Peperomia nopalana	*Peperomia nopalana*	43	100	0	0
Peperomia nossibeana	*Peperomia nossibeana*	71	0	100	0
Peperomia novemnervia	*Peperomia novemnervia*	268	0	0	100
Peperomia nudicaulis	*Peperomia nudicaulis*	255	100	0	0
Peperomia nudifolia	*Peperomia nudifolia*	255	100	0	0
Peperomia nummularioides	*Peperomia nummularioides*	255	100	0	0

Peperomia oahuensis	*Peperomia oahuensis*	73	100	0	0
Peperomia oahuensis	*Peperomia oahuensis*	255	100	0	0
Peperomia oahuensis	*Peperomia oahuensis*	387	85	0	15
Peperomia oahuensis	*Peperomia lonchophylla*				
Peperomia oahuensis	*Peperomia salmensis*				
Peperomia oahuensis	*Peperomia dextrolaeva*	198	100	0	0
Peperomia obcordata	*Peperomia obcordata*	29	0	0	100
Peperomia obcordata	*Peperomia obcordata*	255	100	0	0
Peperomia obcordatifolia	*Peperomia obcordatifolia*	29	0	0	100
Peperomia obcordatifolia	*Peperomia obcordatifolia*	255	100	0	0
Peperomia obex	*Peperomia obex*	29	100	0	0
Peperomia oblancifolia	*Peperomia oblancifolia*	255	0	100	0
Peperomia obliqua	*Peperomia obliqua*	29	0	0	100
Peperomia obliqua	*Peperomia obliqua*	255	100	0	0
Peperomia obovalifolia	*Peperomia obovalifolia*	43	50	0	50
Peperomia obovalifolia	*Peperomia obovalifolia*	255	50	0	50
Peperomia obovalis	*Peperomia obovalis*	43	100	0	0
Peperomia obovalis	*Peperomia obovalis*	373	0	0	100
Peperomia obovalis	*Peperomia obovalis*	255	100	0	0
Peperomia obovatilimba	*Peperomia obovatilimba*	73	0	0	100
Peperomia obovatilimba	*Peperomia obovatilimba*	255	100	0	0
Peperomia obovatilimba	*Peperomia obovatilimba*	387	50	0	50
Peperomia obovatilimba	*Peperomia acrostigma*	255	100	0	0
Peperomia obovatilimba	*Peperomia astrostigma*	73	0	0	100
Peperomia obovatilimba	*Peperomia astrostigma*	198	0	0	100
Peperomia obruenda	*Peperomia obruenda*	29	0	0	100
Peperomia obscurifolia	*Peperomia obscurifolia*	43	100	0	0
Peperomia obscurifolia	*Peperomia obscurifolia*	57	0	0	100
Peperomia obscurifolia	*Peperomia obscurifolia*	60	50	50	0
Peperomia obscurifolia	*Peperomia obscurifolia*	219	100	0	0
Peperomia obscurifolia	*Peperomia obscurifolia*	255	100	0	0
Peperomia obscurifolia	*Peperomia chrysleri*	57	0	0	100
Peperomia obtusifolia	*Peperomia obtusifolia*	24	100	0	0
Peperomia obtusifolia	*Peperomia obtusifolia*	51	50	50	0
Peperomia obtusifolia	*Peperomia obtusifolia*	31	100	0	0
Peperomia obtusifolia	*Peperomia obtusifolia*	43	45	10	45
Peperomia obtusifolia	*Peperomia obtusifolia*	52	100	0	0
Peperomia obtusifolia	*Peperomia obtusifolia*	56	100	0	0
Peperomia obtusifolia	*Peperomia obtusifolia*	57	0	0	100
Peperomia obtusifolia	*Peperomia obtusifolia*	60	50	0	50
Peperomia obtusifolia	*Peperomia obtusifolia*	110	50	50	0
Peperomia obtusifolia	*Peperomia obtusifolia*	150	33.3	33.3	33.4
Peperomia obtusifolia	*Peperomia obtusifolia*	213	0	0	100
Peperomia obtusifolia	*Peperomia obtusifolia*	219	50	0	50
Peperomia obtusifolia	*Peperomia obtusifolia*	227	33.3	33.3	33.4
Peperomia obtusifolia	*Peperomia obtusifolia*	374	94.1	0	5.9
Peperomia obtusifolia	*Peperomia obtusifolia*	21	100	0	0
Peperomia obtusifolia	*Peperomia obtusifolia*	83	33.3	33.3	33.4
Peperomia obtusifolia	*Peperomia obtusifolia*	412	50	0	50
Peperomia obtusifolia	*Peperomia obtusifolia*	13	100	0	0
Peperomia obtusifolia	*Peperomia obtusifolia*	368	50	0	50
Peperomia obtusifolia	*Peperomia obtusifolia*	116	7.5	85	7.5
Peperomia obtusifolia	*Peperomia obtusifolia*	273	15	42.5	42.5
Peperomia obtusifolia	*Peperomia obtusifolia*	259	0	0	100
Peperomia obtusifolia	*Peperomia obtusifolia*	328	70	15	15
Peperomia obtusifolia	*Peperomia obtusifolia*	29	100	0	0
Peperomia obtusifolia	*Peperomia obtusifolia*	373	33.3	33.3	33.4
Peperomia obtusifolia	*Peperomia obtusifolia*	261	50	0	50
Peperomia obtusifolia	*Peperomia obtusifolia*	171	0	50	50
Peperomia obtusifolia	*Peperomia obtusifolia*	134	0	50	50
Peperomia obtusifolia	*Peperomia obtusifolia*	335	50	0	50
Peperomia obtusifolia	*Peperomia obtusifolia*	15	50	0	50
Peperomia obtusifolia	*Peperomia obtusifolia*	294	40	20	40
Peperomia obtusifolia	*Peperomia obtusifolia*	153	50	0	50
Peperomia obtusifolia	*Peperomia obtusifolia*	135	100	0	0
Peperomia obtusifolia	*Peperomia obtusifolia*	136	0	100	0
Peperomia obtusifolia	*Peperomia obtusifolia*	214	100	0	0
Peperomia obtusifolia	*Peperomia obtusifolia*	293	50	50	0
Peperomia obtusifolia	*Peperomia obtusifolia*	224	100	0	0
Peperomia obtusifolia	*Peperomia obtusifolia*	156	0	100	0
Peperomia obtusifolia	*Peperomia obtusifolia*	260	50	0	50
Peperomia obtusifolia	*Peperomia obtusifolia*	255	50	0	50
Peperomia obtusifolia	*Peperomia obtusifolia*	37	85	0	15
Peperomia obtusifolia	*Peperomia obtusifolia*	191	0	0	100
Peperomia obtusifolia	*Peperomia obtusifolia*	90	42.5	15	42.5
Peperomia obtusifolia	*Peperomia cruciata*	29	0	0	100
Peperomia obtusifolia	*Peperomia fieldiana*	29	100	0	0
Peperomia obtusifolia	*Peperomia cangrejalana*	408	0	0	100
Peperomia obtusifolia	*Peperomia gollii*	325	85	0	15
Peperomia obtusifolia	*Peperomia puteolifera*	325	100	0	0

Peperomia obtusifolia	*Peperomia cuneata*				
Peperomia obtusifolia	*Peperomia macropoda*				
Peperomia obtusifolia	*Peperomia subgeminispica*				
Peperomia obtusifolia	*Peperomia emarginata*				
Peperomia obtusifolia	*Peperomia formonensis*				
Peperomia obtusilimba	*Peperomia obtusilimba*	373	50	50	0
Peperomia obtusilimba	*Peperomia obtusilimba*	390	50	50	0
Peperomia occulta	*Peperomia occulta*	43	0	0	100
Peperomia occulta	*Peperomia occulta*	385	0	100	0
Peperomia occulta	*Peperomia occulta*	204	0	100	0
Peperomia ocoana	*Peperomia ocoana*	98	0	100	0
Peperomia ocrosensis	*Peperomia ocrosensis*	292	0	0	100
Peperomia ocumarana	*Peperomia ocumarana*	43	100	0	0
Peperomia ocumarana	*Peperomia ocumarana*	150	100	0	0
Peperomia ocumarana	*Peperomia ocumarana*	219	100	0	0
Peperomia ocumarana	*Peperomia ocumarana*	373	100	0	0
Peperomia ocumarana	*Peperomia ocumarana*	255	50	0	50
Peperomia oerstedii	*Peperomia oerstedii*	43	100	0	0
Peperomia oerstedii	*Peperomia oerstedii*	255	100	0	0
Peperomia oerstedii	*Peperomia oerstedii*	37	50	0	50
Peperomia oerstedii	*Peperomia nievecitana*	57	100	0	0
Peperomia olens	*Peperomia olens*	344	0	0	100
Peperomia olivacea	*Peperomia olivacea*	43	100	0	0
Peperomia olivacea	*Peperomia olivacea*	57	0	0	100
Peperomia olivacea	*Peperomia olivacea*	110	50	50	0
Peperomia olivacea	*Peperomia olivacea*	219	50	0	50
Peperomia olivacea	*Peperomia olivacea*	335	50	0	50
Peperomia olivacea	*Peperomia olivacea*	255	50	0	50
Peperomia olivacea	*Peperomia olivacea*	37	50	0	50
Peperomia olivacea	*Peperomia barbana*				
Peperomia olivacea	*Peperomia novella*	402	100	0	0
Peperomia olivacea	*Peperomia copeyana*				
Peperomia olivacea	*Peperomia substrigosa*				
Peperomia oliveri	*Peperomia oliveri*	388	0	50	50
Peperomia oliveri	*Peperomia oliveri*	198	33.3	33.3	33.4
Peperomia oliveri	*Peperomia oliveri*	115	33.3	33.3	33.4
Peperomia oliveri	*Peperomia oliveri*	255	50	0	50
Peperomia ollantaitambona	*Peperomia ollantaitambona*	29	0	0	100
Peperomia ophistachyera	*Peperomia ophistachyera*	255	100	0	0
Peperomia orbiculimba	*Peperomia orbiculimba*	409	0	100	0
Peperomia orbiculimba	*Peperomia orbiculimba*	307	50	50	0
Peperomia orbiculimba	*Peperomia orbiculimba*	255	50	0	50
Peperomia oreophila	*Peperomia oreophila*	51	0	100	0
Peperomia oreophila	*Peperomia oreophila*	82	0	100	0
Peperomia oreophila	*Peperomia oreophila*	47	0	100	0
Peperomia oscarii	*Peperomia oscarii*	373	100	0	0
Peperomia oscarii	*Peperomia oscarii*	255	100	0	0
Peperomia ostolazae	*Peperomia ostolazae*	250	0	100	0
Peperomia ottoniana	*Peperomia ottoniana*				
Peperomia ouabianae	*Peperomia ouabianae*	150	50	50	0
Peperomia ouabianae	*Peperomia ouabianae*	227	50	50	0
Peperomia ouabianae	*Peperomia ouabianae*	83	50	50	0
Peperomia ouabianae	*Peperomia ouabianae*	373	70	30	0
Peperomia ouabianae	*Peperomia ouabianae*	214	100	0	0
Peperomia ouabianae	*Peperomia ouabianae*	255	50	50	0
Peperomia ouabianae	*Peperomia tafelbergensis*	373	70	30	0
Peperomia ouabianae	*Peperomia herbertsmithii*	373	100	0	0
Peperomia ouabianae	*Peperomia tafelbergensis*	261	100	0	0
Peperomia ouabianae	*Peperomia wurdackii*	414	100	0	0
Peperomia ouabianae	*Peperomia wurdackii*	182	100	0	0
Peperomia ovatolanceolata	*Peperomia ovatolanceolata*	255	100	0	0
Peperomia ovatopeltata	*Peperomia ovatopeltata*	293	0	50	50
Peperomia ovatopeltata	*Peperomia tuberosa*				
Peperomia oxyphylla	*Peperomia oxyphylla*	29	0	0	100
Peperomia pachiteana	*Peperomia pachiteana*	29	0	0	100
Peperomia pachiteana	*Peperomia pachiteana*	178	100	0	0
Peperomia pachydermis	*Peperomia pachydermis*	255	100	0	0
Peperomia pachydermis	*Peperomia pachydermis*	291	0	100	0
Peperomia pachyspadix	*Peperomia pachyspadix*	249	0	50	50
Peperomia pachystachya	*Peperomia pachystachya*	373	0	0	100
Peperomia pachystachya	*Peperomia phyllostachya*	203	0	0	100
Peperomia pacificicola	*Peperomia pacificicola*	151	100	0	0
Peperomia pacificicola	*Peperomia pacifica*				
Peperomia painteri	*Peperomia painteri*	274	0	0	100
Peperomia pakipski	*Peperomia pakipski*	29	100	0	0
Peperomia pakipski	*Peperomia pakipski*	255	100	0	0
Peperomia pakipski	*Peperomia pakipski*	178	100	0	0
Peperomia palcana	*Peperomia palcana*	29	0	0	100
Peperomia pallens	*Peperomia pallens*	255	100	0	0
Peperomia pallescens	*Peperomia pallescens*				

Peperomia pallida	*Peperomia pallida*	388	47.5	47.5	5
Peperomia pallida	*Peperomia pallida*	115	47.5	47.5	5
Peperomia pallida	*Peperomia pallida*	255	50	50	0
Peperomia pallida	*Peperomia anderssonii*				
Peperomia pallida	*Peperomia subglabra*	36	0	100	0
Peperomia pallidibacca	*Peperomia pallidibacca*	238	0	0	100
Peperomia pallidibacca	*Peperomia pallidibacca*	70	100	0	0
Peperomia pallidibacca	*Peperomia pallidibacca*	255	100	0	0
Peperomia pallidibacca	*Peperomia panaiana*				
Peperomia pallidibacca	*Peperomia ramosii*				
Peperomia pallidinervis	*Peperomia pallidinervis*				
Peperomia palmana	*Peperomia palmana*	43	33	33	34
Peperomia palmana	*Peperomia palmana*	57	0	0	100
Peperomia palmana	*Peperomia palmana*	219	50	0	50
Peperomia palmana	*Peperomia palmana*	255	50	0	50
Peperomia palmana	*Peperomia palmana*	37	50	0	50
Peperomia palmana	*Peperomia exuberantifolia*				
Peperomia palmiformis	*Peperomia palmiformis*	247	0	0	100
Peperomia palmiriensis	*Peperomia palmiriensis*	203	0	0	100
Peperomia palmiriensis	*Peperomia palmiriensis*	373	0	0	100
Peperomia pampalcana	*Peperomia pampalcana*	29	0	0	100
Peperomia pandiana	*Peperomia pandiana*	373	0	0	100
Peperomia pangerangoana	*Peperomia pangerangoana*	78	100	0	0
Peperomia papillispica	*Peperomia papillispica*	82	0	0	100
Peperomia papillispica	*Peperomia papillispica*	377	0	100	0
Peperomia papillosa	*Peperomia papillosa*	95	100	0	0
Peperomia paradoxa	*Peperomia paradoxa*	203	50	0	50
Peperomia paraguayensis	*Peperomia paraguayensis*	237	100	0	0
Peperomia paramuna	*Peperomia paramuna*	43	0	0	100
Peperomia paramuna	*Peperomia paramuna*	204	0	0	100
Peperomia parasitica	*Peperomia parasitica*	374	100	0	0
Peperomia parasitica	*Peperomia parasitica*	373	50	50	0
Peperomia parasitica	*Peperomia parasitica*	255	100	0	0
Peperomia parastriata	*Peperomia parastriata*	43	33	33	34
Peperomia parastriata	*Peperomia parastriata*	110	50	50	0
Peperomia parastriata	*Peperomia parastriata*	205	50	50	0
Peperomia parastriata	*Peperomia parastriata*	255	33.3	33.3	33.4
Peperomia parcicilia	*Peperomia parcicilia*	168	100	0	0
Peperomia parcifolia	*Peperomia parcifolia*	51	100	0	0
Peperomia parcifolia	*Peperomia parcifolia*	82	100	0	0
Peperomia parcipeltata	*Peperomia parcipeltata*	202	100	0	0
Peperomia parcipeltata	*Peperomia parcipeltata*	255	100	0	0
Peperomia parhamii	*Peperomia parhamii*	409	100	0	0
Peperomia parhamii	*Peperomia parhamii*	307	100	0	0
Peperomia parhamii	*Peperomia parhamii*	255	100	0	0
Peperomia pariensis	*Peperomia pariensis*	150	100	0	0
Peperomia pariensis	*Peperomia pariensis*	255	100	0	0
Peperomia parnassiifolia	*Peperomia parnassiifolia*	51	100	0	0
Peperomia parnassiifolia	*Peperomia parnassiifolia*	82	100	0	0
Peperomia parnassiifolia	*Peperomia parnassiifolia*	255	0	0	100
Peperomia parva	*Peperomia parva*	29	0	0	100
Peperomia parva	*Peperomia parva*	255	100	0	0
Peperomia parva	*Peperomia parva*	178	100	0	0
Peperomia parvibacca	*Peperomia parvibacca*	255	100	0	0
Peperomia parvibractea	*Peperomia parvibractea*				
Peperomia parvicaulis	*Peperomia parvicaulis*	78	100	0	0
Peperomia parvifolia	*Peperomia parvifolia*	29	0	50	50
Peperomia parvifolia	*Peperomia parvifolia*	418	0	0	100
Peperomia parvifolia	*Peperomia parvifolia*	255	0	50	50
Peperomia parvilimba	*Peperomia parvilimba*	373	0	0	100
Peperomia parvilimba	*Peperomia parvilimba*	255	100	0	0
Peperomia parvipunctulata	*Peperomia parvipunctulata*	29	0	0	100
Peperomia parvisagittata	*Peperomia parvisagittata*	292	0	0	100
Peperomia parvulifolia	*Peperomia parvulifolia*	255	100	0	0
Peperomia pasionana	*Peperomia pasionana*	209	0	50	50
Peperomia patula	*Peperomia patula*	150	33.3	33.3	33.4
Peperomia patula	*Peperomia patula*	373	100	0	0
Peperomia patula	*Peperomia patula*	255	50	0	50
Peperomia patula	*Peperomia purpurella*				
Peperomia pavoniana	*Peperomia pavoniana*	29	0	0	100
Peperomia pearcei	*Peperomia pearcei*	29	0	0	100
Peperomia pearcei	*Peperomia pearcei*	255	100	0	0
Peperomia peckelii	*Peperomia peckelii*				
Peperomia pecuniifolia	*Peperomia pecuniifolia*	43	50	50	0
Peperomia pecuniifolia	*Peperomia pecuniifolia*	110	90	10	0
Peperomia pecuniifolia	*Peperomia pecuniifolia*	255	100	0	0
Peperomia pecuniifolia	*Peperomia pecuniifolia*	328	50	50	0
Peperomia pedicellata	*Peperomia pedicellata*	43	0	0	100
Peperomia pedicellata	*Peperomia pedicellata*	110	50	50	0
Peperomia pedunculata	*Peperomia pedunculata*	255	50	0	50

Peperomia pedunculata	*Peperomia monini*				
Peperomia pellucida	*Peperomia pellucida*	51	0	50	50
Peperomia pellucida	*Peperomia pellucida*	43	0	0	100
Peperomia pellucida	*Peperomia pellucida*	57	0	0	100
Peperomia pellucida	*Peperomia pellucida*	89	0	0	100
Peperomia pellucida	*Peperomia pellucida*	110	33.3	33.3	33.4
Peperomia pellucida	*Peperomia pellucida*	150	0	0	100
Peperomia pellucida	*Peperomia pellucida*	154	0	0	100
Peperomia pellucida	*Peperomia pellucida*	213	0	0	100
Peperomia pellucida	*Peperomia pellucida*	227	0	0	100
Peperomia pellucida	*Peperomia pellucida*	374	0	0	100
Peperomia pellucida	*Peperomia pellucida*	27	0	50	50
Peperomia pellucida	*Peperomia pellucida*	83	0	0	100
Peperomia pellucida	*Peperomia pellucida*	255	100	0	0
Peperomia pellucida	*Peperomia pellucida*	172	0	50	50
Peperomia pellucida	*Peperomia pellucida*	118	0	50	50
Peperomia pellucida	*Peperomia pellucida*	144	0	0	100
Peperomia pellucida	*Peperomia pellucida*	328	0	0	100
Peperomia pellucida	*Peperomia pellucida*	376	0	0	100
Peperomia pellucida	*Peperomia pellucida*	29	0	0	100
Peperomia pellucida	*Peperomia pellucida*	373	0	0	100
Peperomia pellucida	*Peperomia pellucida*	384	0	50	50
Peperomia pellucida	*Peperomia pellucida*	125	0	0	100
Peperomia pellucida	*Peperomia pellucida*	171	0	0	100
Peperomia pellucida	*Peperomia pellucida*	91	7.5	7.5	85
Peperomia pellucida	*Peperomia pellucida*	16	15	0	85
Peperomia pellucida	*Peperomia pellucida*	272	0	0	100
Peperomia pellucida	*Peperomia pellucida*	86	0	0	100
Peperomia pellucida	*Peperomia pellucida*	12	33.3	33.3	33.4
Peperomia pellucida	*Peperomia pellucida*	335	5	15	80
Peperomia pellucida	*Peperomia pellucida*	155	33.3	33.3	33.4
Peperomia pellucida	*Peperomia pellucida*	15	0	0	100
Peperomia pellucida	*Peperomia pellucida*	294	0	50	50
Peperomia pellucida	*Peperomia pellucida*	387	0	0	100
Peperomia pellucida	*Peperomia pellucida*	307	15	0	85
Peperomia pellucida	*Peperomia pellucida*	119	0	0	100
Peperomia pellucida	*Peperomia pellucida*	136	0	0	100
Peperomia pellucida	*Peperomia pellucida*	214	0	0	100
Peperomia pellucida	*Peperomia pellucida*	42	0	0	100
Peperomia pellucida	*Peperomia pellucida*	418	0	0	100
Peperomia pellucida	*Peperomia pellucida*	224	0	0	100
Peperomia pellucida	*Peperomia pellucida*	115	0	0	100
Peperomia pellucida	*Peperomia pellucida*	260	0	0	100
Peperomia pellucida	*Peperomia pellucida*	37	5	0	95
Peperomia pellucida	*Peperomia pellucida*	32	0	50	50
Peperomia pellucida	*Peperomia pellucida*	112	0	0	100
Peperomia pellucida	*Peperomia pellucida*	191	0	0	100
Peperomia pellucida	*Peperomia pellucida*	216	0	100	0
Peperomia pellucida	*Peperomia pellucida*	325	0	0	100
Peperomia pellucida	*Peperomia pellucida*	138	50	0	50
Peperomia pellucida	*Peperomia pellucida*	88	0	50	50
Peperomia pellucida	*Peperomia praetenuis*	15	0	0	100
Peperomia pellucida	*Peperomia tenuiflora*	373	0	0	100
Peperomia pellucida	*Peperomia oleracea*				
Peperomia pellucida	*Peperomia translucens*				
Peperomia pellucida	*Peperomia yapensis*				
Peperomia pellucida	*Peperomia ephemera*				
Peperomia pellucida	*Peperomia knoblecheriana*				
Peperomia pellucida	*Peperomia triadophylla*				
Peperomia pellucida	*Peperomia vogelii*				
Peperomia pellucidoides	*Peperomia pellucidoides*	51	0	50	50
Peperomia pellucidoides	*Peperomia pellucidoides*	82	0	50	50
Peperomia pellucidopunctulata	*Peperomia pellucidopunctulata*	70	0	50	50
Peperomia pellucidopunctulata	*Peperomia pellucidopunctulata*	238	50	50	0
Peperomia pellucidopunctulata	*Peperomia pellucidopunctulata*	255	100	0	0
Peperomia peltaphylla	*Peperomia peltaphylla*	373	0	0	100
Peperomia peltifolia	*Peperomia peltifolia*	345	0	0	100
Peperomia peltigera	*Peperomia peltigera*	374	0	0	100
Peperomia peltigera	*Peperomia peltigera*	373	0	0	100
Peperomia peltilimba	*Peperomia peltilimba*	43	45	10	45
Peperomia peltilimba	*Peperomia peltilimba*	110	50	50	0
Peperomia peltilimba	*Peperomia peltilimba*	213	100	0	0
Peperomia peltilimba	*Peperomia peltilimba*	328	15	70	15
Peperomia peltilimba	*Peperomia peltilimba*	335	100	0	0
Peperomia peltilimba	*Peperomia peltilimba*	293	100	0	0
Peperomia peltilimba	*Peperomia peltilimba*	255	100	0	0
Peperomia peltilimba	*Peperomia peltilimba*	37	50	50	0
Peperomia peltilimba	*Peperomia agitata*	328	15	0	85
Peperomia peltilimba	*Peperomia vegana*	328	0	15	85
Peperomia peltilimba	*Peperomia vegana*	110	50	50	0

Peperomia peltoidea	*Peperomia peltoidea*	19	100	0	0
Peperomia peltoidea	*Peperomia peltoidea*	150	33.3	33.3	33.4
Peperomia peltoidea	*Peperomia peltoidea*	338	0	0	100
Peperomia peltoidea	*Peperomia peltoidea*	374	60	0	40
Peperomia peltoidea	*Peperomia peltoidea*	21	100	0	0
Peperomia peltoidea	*Peperomia peltoidea*	255	50	0	50
Peperomia peltoidea	*Peperomia peltoidea*	90	42.5	42.5	15
Peperomia peltoidea	*Peperomia pseudopeltoidea*				
Peperomia pendulicaulis	*Peperomia pendulicaulis*	373	100	0	0
Peperomia pendulicaulis	*Peperomia pendulicaulis*	255	100	0	0
Peperomia penduliramea	*Peperomia penduliramea*	373	100	0	0
Peperomia penduliramea	*Peperomia penduliramea*	255	100	0	0
Peperomia penicillata	*Peperomia penicillata*	140	0	100	0
Peperomia pentadactyla	*Peperomia pentadactyla*				
Peperomia peploides	*Peperomia peploides*	255	100	0	0
Peperomia percalvescens	*Peperomia percalvescens*	29	0	0	100
Peperomia percalvescens	*Peperomia percalvescens*	255	100	0	0
Peperomia perciliata	*Peperomia perciliata*	373	100	0	0
Peperomia perciliata	*Peperomia perciliata*	255	100	0	0
Peperomia pereneana	*Peperomia pereneana*	29	0	0	100
Peperomia pereneana	*Peperomia pereneana*	178	0	0	100
Peperomia pereskiifolia	*Peperomia pereskiifolia*	128	100	0	0
Peperomia pereskiifolia	*Peperomia pereskiifolia*	51	34	33	33
Peperomia pereskiifolia	*Peperomia pereskiifolia*	338	0	0	100
Peperomia pereskiifolia	*Peperomia pereskiifolia*	24	100	0	0
Peperomia pereskiifolia	*Peperomia pereskiifolia*	57	100	0	0
Peperomia pereskiifolia	*Peperomia pereskiifolia*	150	0	50	50
Peperomia pereskiifolia	*Peperomia pereskiifolia*	183	50	50	0
Peperomia pereskiifolia	*Peperomia pereskiifolia*	184	100	0	0
Peperomia pereskiifolia	*Peperomia pereskiifolia*	374	100	0	0
Peperomia pereskiifolia	*Peperomia pereskiifolia*	21	100	0	0
Peperomia pereskiifolia	*Peperomia pereskiifolia*	29	100	0	0
Peperomia pereskiifolia	*Peperomia pereskiifolia*	373	0	50	50
Peperomia pereskiifolia	*Peperomia pereskiifolia*	86	0	50	50
Peperomia pereskiifolia	*Peperomia pereskiifolia*	179	33.3	33.4	33.3
Peperomia pereskiifolia	*Peperomia pereskiifolia*	252	33.3	33.3	33.4
Peperomia pereskiifolia	*Peperomia pereskiifolia*	37	50	50	0
Peperomia pereskiifolia	*Peperomia morungavana*	255	50	0	50
Peperomia pereskiifolia	*Peperomia plicata*	255	50	50	0
Peperomia pereskiifolia	*Peperomia quicheensis*	176	0	100	0
Peperomia pereskiifolia	*Peperomia pereskiifolia*	375	30	60	10
Peperomia perforata	*Peperomia perforata*	43	100	0	0
Peperomia perforata	*Peperomia perforata*	110	100	0	0
Peperomia perforata	*Peperomia perforata*	255	100	0	0
Peperomia perglandulosa	*Peperomia perglandulosa*	43	100	0	0
Peperomia perglandulosa	*Peperomia perglandulosa*	57	100	0	0
Peperomia perglandulosa	*Peperomia perglandulosa*	255	100	0	0
Peperomia perlongicaulis	*Peperomia perlongicaulis*	51	0	0	100
Peperomia perlongicaulis	*Peperomia perlongicaulis*	82	100	0	0
Peperomia perlongicaulis	*Peperomia perlongicaulis*	255	100	0	0
Peperomia perlongipedunculata	*Peperomia perlongipedunculata*	373	0	0	100
Peperomia perlongipes	*Peperomia perlongipes*	381	100	0	0
Peperomia perlongipes	*Peperomia perlongipes*	351	50	50	0
Peperomia perlongispica	*Peperomia perlongispica*				
Peperomia pernambucensis	*Peperomia pernambucensis*	51	100	0	0
Peperomia pernambucensis	*Peperomia pernambucensis*	43	100	0	0
Peperomia pernambucensis	*Peperomia pernambucensis*	57	0	0	100
Peperomia pernambucensis	*Peperomia pernambucensis*	150	100	0	0
Peperomia pernambucensis	*Peperomia pernambucensis*	227	50	0	50
Peperomia pernambucensis	*Peperomia pernambucensis*	374	90.9	0	8.1
Peperomia pernambucensis	*Peperomia pernambucensis*	21	100	0	0
Peperomia pernambucensis	*Peperomia pernambucensis*	83	50	0	50
Peperomia pernambucensis	*Peperomia pernambucensis*	373	50	0	50
Peperomia pernambucensis	*Peperomia pernambucensis*	261	50	0	50
Peperomia pernambucensis	*Peperomia pernambucensis*	335	95	0	5
Peperomia pernambucensis	*Peperomia pernambucensis*	255	50	0	50
Peperomia pernambucensis	*Peperomia pernambucensis*	37	100	0	0
Peperomia pernambucensis	*Peperomia balsapuertana*	29	0	0	100
Peperomia pernambucensis	*Peperomia lechleriana*	29	0	0	100
Peperomia pernambucensis	*Peperomia balsapuertana*	178	100	0	0
Peperomia pernambucensis	*Peperomia atirroana*				
Peperomia pernambucensis	*Peperomia breviscapa*	323	100	0	0
Peperomia pernambucensis	*Peperomia subacaulis*				
Peperomia perodiniana	*Peperomia perodiniana*	255	100	0	0
Peperomia persucculenta	*Peperomia persucculenta*	111	0	0	100
Peperomia persulcata	*Peperomia persulcata*	368	0	0	100
Peperomia persulcata	*Peperomia persulcata*	373	0	0	100
Peperomia pertomentella	*Peperomia pertomentella*	29	0	0	100
Peperomia pertomentella	*Peperomia pertomentella*	255	100	0	0

Peperomia pertomentella	*Peperomia pertomentella*	178	0	0	100
Peperomia peruviana	*Peperomia peruviana*	150	0	0	100
Peperomia peruviana	*Peperomia peruviana*	380	0	0	100
Peperomia peruviana	*Peperomia peruviana*	368	0	0	100
Peperomia peruviana	*Peperomia peruviana*	29	0	0	100
Peperomia peruviana	*Peperomia peruviana*	373	0	100	0
Peperomia peruviana	*Peperomia peruviana*	418	0	0	100
Peperomia peruviana	*Peperomia peruviana*	178	0	50	50
Peperomia peruviana	*Peperomia peruviana*	121	0	85	15
Peperomia petiolaris	*Peperomia petiolaris*	294	0	30	70
Peperomia petiolaris	*Peperomia petiolaris*	255	100	0	0
Peperomia petiolaris	*Peperomia taco-tacoi*	105	0	100	0
Peperomia petiolaris	*Peperomia perherbacea*	99	0	100	0
Peperomia petiolata	*Peperomia petiolata*	373	10	75	15
Peperomia petiolata	*Peperomia petiolata*	390	33.3	33.4	33.3
Peperomia petiolata	*Peperomia petiolata*	255	100	0	0
Peperomia petraea	*Peperomia petraea*	373	0	0	100
Peperomia petraea	*Peperomia petraea*	65	0	100	0
Peperomia petrophila	*Peperomia petrophila*	43	0	100	0
Peperomia petrophila	*Peperomia petrophila*	57	100	0	0
Peperomia petrophila	*Peperomia petrophila*	110	10	45	45
Peperomia petrophila	*Peperomia petrophila*	150	100	0	0
Peperomia petrophila	*Peperomia petrophila*	213	100	0	0
Peperomia petrophila	*Peperomia petrophila*	338	0	100	0
Peperomia petrophila	*Peperomia petrophila*	328	0	50	50
Peperomia petrophila	*Peperomia petrophila*	373	0	50	50
Peperomia petrophila	*Peperomia petrophila*	335	0	0	100
Peperomia petrophila	*Peperomia petrophila*	294	0	50	50
Peperomia petrophila	*Peperomia petrophila*	255	100	0	0
Peperomia petrophila	*Peperomia petrophila*	37	0	0	100
Peperomia petrophila	*Peperomia petrophila*	90	0	0	100
Peperomia philipsonii	*Peperomia philipsonii*	57	0	0	100
Peperomia philipsonii	*Peperomia philipsonii*	416	0	0	100
Peperomia phyllantha	*Peperomia phyllantha*	299	0	50	50
Peperomia phyllanthopsis	*Peperomia phyllanthopsis*	203	0	0	100
Peperomia phyllanthopsis	*Peperomia phyllanthopsis*	373	0	0	100
Peperomia physostachya	*Peperomia physostachya*	207	0	50	50
Peperomia pichinchae	*Peperomia pichinchae*	373	0	0	100
Peperomia pichinchae	*Peperomia pichinchae*	255	100	0	0
Peperomia pichisensis	*Peperomia pichisensis*	29	0	0	100
Peperomia pichisensis	*Peperomia pichisensis*	255	100	0	0
Peperomia pichisensis	*Peperomia pichisensis*	178	100	0	0
Peperomia pilicaulis	*Peperomia pilicaulis*	51	100	0	0
Peperomia pilicaulis	*Peperomia pilicaulis*	57	100	0	0
Peperomia pilicaulis	*Peperomia pilicaulis*	374	92.3	0	7.7
Peperomia pilicaulis	*Peperomia pilicaulis*	21	100	0	0
Peperomia pilicaulis	*Peperomia pilicaulis*	373	100	0	0
Peperomia pilicaulis	*Peperomia pilicaulis*	335	100	0	0
Peperomia pilicaulis	*Peperomia pilicaulis*	255	100	0	0
Peperomia pilicaulis	*Peperomia pilicaulis*	191	100	0	0
Peperomia pilicaulis	*Peperomia pilicaulis*	90	100	0	0
Peperomia pilifera	*Peperomia pilifera*	29	0	0	100
Peperomia pilipetiolata	*Peperomia pilipetiolata*	43	100	0	0
Peperomia pilipetiolata	*Peperomia pilipetiolata*	255	100	0	0
Peperomia pillahuatana	*Peperomia pillahuatana*	29	0	0	100
Peperomia pilocarpa	*Peperomia pilocarpa*	251	85	0	15
Peperomia pilosa	*Peperomia pilosa*	29	0	0	100
Peperomia pilostigma	*Peperomia pilostigma*	143	0	100	0
Peperomia pilostigma	*Peperomia pilostigma*	307	0	100	0
Peperomia pilulifera	*Peperomia pilulifera*	319	100	0	0
Peperomia pinedoana	*Peperomia pinedoana*	29	0	0	100
Peperomia pinoi	*Peperomia pinoi*	198	0	0	100
Peperomia pinoi	*Peperomia pinoi*	203	0	0	100
Peperomia pitcairnensis	*Peperomia pitcairnensis*	255	100	0	0
Peperomia pitiguayana	*Peperomia pitiguayana*	255	100	0	0
Peperomia pittieri	*Peperomia pittieri*	43	100	0	0
Peperomia pittieri	*Peperomia pittieri*	57	0	0	100
Peperomia pittieri	*Peperomia pittieri*	219	100	0	0
Peperomia pittieri	*Peperomia pittieri*	255	33.3	33.3	33.4
Peperomia pittieri	*Peperomia pittieri*	37	100	0	0
Peperomia playapampana	*Peperomia playapampana*	29	0	0	100
Peperomia pleiomorpha	*Peperomia pleiomorpha*	29	0	0	100
Peperomia pleiomorpha	*Peperomia pleiomorpha*	255	100	0	0
Peperomia pleiomorpha	*Peperomia pleiomorpha*	178	100	0	0
Peperomia plicatifolia	*Peperomia plicatifolia*	29	0	0	100
Peperomia plicatifolia	*Peperomia plicatifolia*	255	100	0	0
Peperomia plurispica	*Peperomia plurispica*	29	0	0	100
Peperomia pluvisilvatica	*Peperomia pluvisilvatica*	199	100	0	0
Peperomia pluvisilvatica	*Peperomia pluvisilvatica*	255	100	0	0
Peperomia poasana	*Peperomia poasana*	43	15	0	85

Peperomia poasana	*Peperomia poasana*	57	100	0	0
Peperomia poasana	*Peperomia poasana*	219	50	0	50
Peperomia poasana	*Peperomia poasana*	255	50	0	50
Peperomia poasana	*Peperomia poasana*	37	50	0	50
Peperomia polybotrya	*Peperomia polybotrya*	373	0	0	100
Peperomia polybotrya	*Peperomia polybotrya*	191	0	0	100
Peperomia polycephala	*Peperomia polycephala*	29	0	0	100
Peperomia polymorpha	*Peperomia polymorpha*	29	0	0	100
Peperomia polymorpha	*Peperomia polymorpha*	255	100	0	0
Peperomia polymorpha	*Peperomia polymorpha*	178	100	0	0
Peperomia polystachyoides	*Peperomia polystachyoides*	51	100	0	0
Peperomia polystachyoides	*Peperomia polystachyoides*	82	50	50	0
Peperomia polystachyoides	*Peperomia polystachyoides*	255	100	0	0
Peperomia polystachyoides	*Peperomia variabilis*				
Peperomia polystachyos	*Peperomia polystachyos*	255	100	0	0
Peperomia polystachyos	*Peperomia polystachyos*	32	0	100	0
Peperomia polystachyos	*Peperomia polystachyos*	216	0	50	50
Peperomia polzii	*Peperomia polzii*	198	0	100	0
Peperomia ponapensis	*Peperomia ponapensis*	118	0	100	0
Peperomia ponapensis	*Peperomia gibbonsii*	198	0	100	0
Peperomia ponapensis	*Peperomia volkensii*				
Peperomia pongoana	*Peperomia pongoana*	29	0	0	100
Peperomia pongoana	*Peperomia pongoana*	255	100	0	0
Peperomia pongoana	*Peperomia pongoana*	191	0	0	100
Peperomia pontina	*Peperomia pontina*	29	0	0	100
Peperomia pontina	*Peperomia pontina*	255	100	0	0
Peperomia pontina	*Peperomia pontina*	178	100	0	0
Peperomia popayanensis	*Peperomia popayanensis*				
Peperomia porphyridea	*Peperomia porphyridea*	255	100	0	0
Peperomia porphyridea	*Peperomia porphyridea*	191	100	0	0
Peperomia portobellensis	*Peperomia portobellensis*	31	100	0	0
Peperomia portobellensis	*Peperomia portobellensis*	43	50	50	0
Peperomia portobellensis	*Peperomia portobellensis*	57	0	0	100
Peperomia portobellensis	*Peperomia portobellensis*	110	50	50	0
Peperomia portobellensis	*Peperomia portobellensis*	255	50	50	0
Peperomia portobellensis	*Peperomia caudulilimba*	57	0	0	100
Peperomia portobellensis	*Peperomia gatunensis*	253	100	0	0
Peperomia portobellensis	*Peperomia caudulilimba*	410	100	0	0
Peperomia portobellensis	*Peperomia faucium-bovis*				
Peperomia portobellensis	*Peperomia oxycarpa*				
Peperomia portoricensis	*Peperomia portoricensis*	294	50	0	50
Peperomia portoricensis	*Peperomia portoricensis*	255	100	0	0
Peperomia portoricensis	*Peperomia intermixta*				
Peperomia portoricensis	*Peperomia trichocaulis*				
Peperomia portuguesensis	*Peperomia portuguesensis*	150	100	0	0
Peperomia portuguesensis	*Peperomia portuguesensis*	255	100	0	0
Peperomia portuguesensis	*Peperomia portuguesensis*	90	100	0	0
Peperomia portulacoides	*Peperomia portulacoides*	340	0	50	50
Peperomia portulacoides	*Peperomia portulacoides*	125	50	50	0
Peperomia portulacoides	*Peperomia portulacoides*	272	95	5	0
Peperomia portulacoides	*Peperomia portulacoides*	391	50	50	0
Peperomia portulacoides	*Peperomia portulacoides*	255	100	0	0
Peperomia portulacoides	*Peperomia candolleana*	376	0	100	0
Peperomia portulacoides	*Peperomia candolleana*	155	50	50	0
Peperomia portulacoides	*Peperomia courtallensis*	391	50	50	0
Peperomia portulacoides	*Peperomia confusa*				
Peperomia portulacoides	*Peperomia perrottetiana*				
Peperomia potamophila	*Peperomia potamophila*	43	50	50	0
Peperomia potamophila	*Peperomia potamophila*	255	50	50	0
Peperomia ppucu-ppucu	*Peperomia ppucu-ppucu*	29	0	0	100
Peperomia ppucu-ppucu	*Peperomia ppucu-ppucu*	145	0	100	0
Peperomia praematura	*Peperomia praematura*	150	0	0	100
Peperomia praematura	*Peperomia praematura*	373	0	0	100
Peperomia praeruptorum	*Peperomia praeruptorum*	239	0	100	0
Peperomia praetenuis	*Peperomia praetenuis*				
Peperomia pringlei	*Peperomia pringlei*	43	100	0	0
Peperomia pringlei	*Peperomia pringlei*	110	100	0	0
Peperomia pringlei	*Peperomia pringlei*	255	100	0	0
Peperomia proctorii	*Peperomia proctorii*	258	0	100	0
Peperomia procumbens	*Peperomia procumbens*	311	0	0	100
Peperomia procumbens	*Peperomia procumbens*	287	0	100	0
Peperomia productamenta	*Peperomia productamenta*	100	0	100	0
Peperomia profissa	*Peperomia profissa*	29	0	0	100
Peperomia prolifera	*Peperomia prolifera*	29	0	0	100
Peperomia propugnaculi	*Peperomia propugnaculi*	370	0	100	0
Peperomia prostrata	*Peperomia prostrata*	89	100	0	0
Peperomia prostrata	*Peperomia prostrata*	255	100	0	0
Peperomia nivalis					
Peperomia nivalis					
Peperomia nivalis					

Peperomia pseudoalpina	*Peperomia pseudoalpina*	43	50	0	50
Peperomia pseudoalpina	*Peperomia pseudoalpina*	57	0	0	100
Peperomia pseudoalpina	*Peperomia pseudoalpina*	110	33.3	33.3	33.4
Peperomia pseudoalpina	*Peperomia pseudoalpina*	219	50	0	50
Peperomia pseudoalpina	*Peperomia pseudoalpina*	335	100	0	0
Peperomia pseudoalpina	*Peperomia pseudoalpina*	293	50	50	0
Peperomia pseudoalpina	*Peperomia pseudoalpina*	255	33.3	33.3	33.4
Peperomia pseudoalpina	*Peperomia pseudoalpina*	37	50	0	50
Peperomia pseudoalternifolia	*Peperomia pseudoalternifolia*	373	100	0	0
Peperomia pseudoalternifolia	*Peperomia pseudoalternifolia*	255	100	0	0
Peperomia pseudoasarifolia	*Peperomia pseudoasarifolia*	43	0	100	0
Peperomia pseudoasarifolia	*Peperomia pseudoasarifolia*	127	0	85	15
Peperomia pseudobcordata	*Peperomia pseudobcordata*	51	100	0	0
Peperomia pseudobcordata	*Peperomia pseudobcordata*	82	100	0	0
Peperomia pseudobcordata	*Peperomia pseudobcordata*	255	100	0	0
Peperomia pseudocasaretti	*Peperomia pseudocasaretti*	43	100	0	0
Peperomia pseudocasaretti	*Peperomia pseudocasaretti*	255	33.3	33.3	33.4
Peperomia pseudocasaretti	*Peperomia nervosa*	23	100	0	0
Peperomia pseudocobana	*Peperomia pseudocobana*	350	0	0	100
Peperomia pseudodependens	*Peperomia pseudodependens*	373	0	0	100
Peperomia pseudodependens	*Peperomia pseudodependens*	37	0	0	100
Peperomia pseudodependens	*Peperomia quirosii*				
Peperomia pseudoelata	*Peperomia pseudoelata*	43	100	0	0
Peperomia pseudoelata	*Peperomia pseudoelata*	255	100	0	0
Peperomia pseudofurcata	*Peperomia pseudofurcata*	29	0	0	100
Peperomia pseudoglabella	*Peperomia pseudoglabella*	150	100	0	0
Peperomia pseudoglabella	*Peperomia pseudoglabella*	255	100	0	0
Peperomia pseudohirta	*Peperomia pseudohirta*	43	100	0	0
Peperomia pseudohirta	*Peperomia pseudohirta*	255	100	0	0
Peperomia pseudohodgei	*Peperomia pseudohodgei*	43	34	33	33
Peperomia pseudohodgei	*Peperomia pseudohodgei*	255	33.3	33.3	33.4
Peperomia pseudomaculosa	*Peperomia pseudomaculosa*	43	0	0	100
Peperomia pseudopereskiifolia	*Peperomia pseudopereskiifolia*	51	50	0	50
Peperomia pseudopereskiifolia	*Peperomia pseudopereskiifolia*	43	33	33	34
Peperomia pseudopereskiifolia	*Peperomia pseudopereskiifolia*	110	50	50	0
Peperomia pseudopereskiifolia	*Peperomia pseudopereskiifolia*	374	100	0	0
Peperomia pseudopereskiifolia	*Peperomia pseudopereskiifolia*	255	50	50	0
Peperomia pseudopereskiifolia	*Peperomia chartacea*	29	100	0	0
Peperomia pseudopereskiifolia	*Peperomia pseudopereskiifolia*	335	50	0	50
Peperomia pseudopereskiifolia	*Peperomia pseudopereskiifolia*	294	0	0	100
Peperomia pseudopereskiifolia	*Peperomia pseudopereskiifolia*	42	100	0	0
Peperomia pseudopereskiifolia	*Peperomia pseudopereskiifolia*	293	100	0	0
Peperomia pseudopereskiifolia	*Peperomia chartacea*	178	100	0	0
Peperomia pseudopereskiifolia	*Peperomia jarisiana*				
Peperomia pseudopereskiifolia	*Peperomia nemoralis*				
Peperomia pseudopereskiifolia	*Peperomia guanacastana*				
Peperomia pseudoperuviana	*Peperomia pseudoperuviana*	292	0	0	100
Peperomia pseudophyllantha	*Peperomia pseudophyllantha*	203	0	0	100
Peperomia pseudorhombea	*Peperomia pseudorhombea*	155	0	100	0
Peperomia pseudorhombea	*Peperomia pseudorhombea*	255	100	0	0
Peperomia pseudorhynchophoros	*Peperomia pseudorhynchophoros*	29	100	0	0
Peperomia pseudorhynchophoros	*Peperomia pseudorhynchophoros*	335	100	0	0
Peperomia pseudorhynchophoros	*Peperomia pseudorhynchophoros*				
Peperomia pseudorhynchophoros	*Peperomia pseudorhynchophoros*	374	33.4	11.11	55.55
Peperomia pseudorhynchophoros	*Peperomia pergamentacea*	29	0	0	100
Peperomia pseudorhynchophoros	*Peperomia wrightiana*	294	50	0	50
Peperomia pseudorufescens	*Peperomia pseudorufescens*				
Peperomia pseudosalicifolia	*Peperomia pseudosalicifolia*	29	0	0	100
Peperomia pseudosalicifolia	*Peperomia pseudosalicifolia*	255	100	0	0
Peperomia pseudoserratirhachis	*Peperomia pseudoserratirhachis*	51	0	50	50
Peperomia pseudoserratirhachis	*Peperomia pseudoserratirhachis*	223	0	50	50
Peperomia pseudoumbilicata	*Peperomia pseudoumbilicata*	17	100	0	0
Peperomia pseudoumbilicata	*Peperomia pseudoumbilicata*	349	0	0	100
Peperomia pseudovariegata	*Peperomia pseudovariegata*	373	0	0	100
Peperomia pseudoverruculosa	*Peperomia pseudoverruculosa*	292	0	0	100
Peperomia psilophylla	*Peperomia psilophylla*	255	100	0	0
Peperomia psilostachya	*Peperomia psilostachya*	51	100	0	0
Peperomia psilostachya	*Peperomia psilostachya*	53	100	0	0
Peperomia psilostachya	*Peperomia psilostachya*	183	100	0	0
Peperomia psilostachya	*Peperomia psilostachya*	184	100	0	0
Peperomia psilostachya	*Peperomia psilostachya*	29	0	0	100
Peperomia psilostachya	*Peperomia psilostachya*	179	100	0	0
Peperomia psilostachya	*Peperomia psilostachya*	84	100	0	0

Peperomia psilostachya	*Peperomia psilostachya*	85	100	0	0
Peperomia psilostachya	*Peperomia trapezoidalis*	418	100	0	0
Peperomia psilostachya	*Peperomia psilostachya*	255	100	0	0
Peperomia psilostachya	*Peperomia psilostachya*	252	100	0	0
Peperomia psilostachya	*Peperomia psilostachya*	375	100	0	0
Peperomia pteroneura	*Peperomia pteroneura*	373	0	0	100
Peperomia puberulescens	*Peperomia puberulescens*	43	50	0	50
Peperomia puberulescens	*Peperomia puberulescens*	255	50	0	50
Peperomia puberulibacca	*Peperomia puberulibacca*	29	0	0	100
Peperomia puberulicaulis	*Peperomia puberulicaulis*	374	50	0	50
Peperomia puberulicaulis	*Peperomia puberulicaulis*	373	100	0	0
Peperomia puberulicaulis	*Peperomia puberulicaulis*	255	100	0	0
Peperomia puberuliformis	*Peperomia puberuliformis*	29	0	0	100
Peperomia puberuliformis	*Peperomia puberuliformis*	178	100	0	0
Peperomia puberulilimba	*Peperomia puberulilimba*	43	50	0	50
Peperomia puberulilimba	*Peperomia puberulilimba*	373	0	15	85
Peperomia puberulilimba	*Peperomia puberulilimba*	255	50	0	50
Peperomia puberulilimba	*Peperomia chlorostachya*				
Peperomia puberulipes	*Peperomia puberulipes*	29	0	0	100
Peperomia puberulirrhachis	*Peperomia puberulirrhachis*				
Peperomia puberulispica	*Peperomia puberulispica*	255	100	0	0
Peperomia pubescens	*Peperomia pubescens*				
Peperomia pubescens	*Peperomia vahlii*				
Peperomia pubescentinervis	*Peperomia pubescentinervis*	29	0	0	100
Peperomia pubicaulis	*Peperomia pubicaulis*				
Peperomia pubilimba	*Peperomia pubilimba*	255	100	0	0
Peperomia pubinervosa	*Peperomia pubinervosa*	29	0	0	100
Peperomia pubinervosa	*Peperomia pubinervosa*	165	0	100	0
Peperomia pubipeduncula	*Peperomia pubipeduncula*	51	100	0	0
Peperomia pubipeduncula	*Peperomia pubipeduncula*	82	100	0	0
Peperomia pubipeduncula	*Peperomia pubipeduncula*	255	100	0	0
Peperomia pubipetiola	*Peperomia pubipetiola*	255	100	0	0
Peperomia pubiramea	*Peperomia pubiramea*	29	0	0	100
Peperomia pubiramea	*Peperomia pubiramea*	255	100	0	0
Peperomia pubiramea	*Peperomia pubiramea*	178	100	0	0
Peperomia pubirhachis	*Peperomia pubirhachis*	373	0	0	100
Peperomia puerto-ospinana	*Peperomia puerto-ospinana*	373	100	0	0
Peperomia puerto-ospinana	*Peperomia puerto-ospinana*	255	100	0	0
Peperomia pugnicaudex	*Peperomia pugnicaudex*	292	0	0	100
Peperomia pullispica	*Peperomia pullispica*	101	0	100	0
Peperomia pululaguana	*Peperomia pululaguana*	255	100	0	0
Peperomia pumila	*Peperomia pumila*	29	0	0	100
Peperomia punctatilamina	*Peperomia punctatilamina*	373	100	0	0
Peperomia punctatilamina	*Peperomia punctatilamina*	255	100	0	0
Peperomia punctulatissima	*Peperomia punctulatissima*	255	100	0	0
Peperomia punicea	*Peperomia punicea*	51	0	0	100
Peperomia punicea	*Peperomia punicea*	82	0	50	50
Peperomia purpurea	*Peperomia purpurea*	29	0	0	100
Peperomia purpureonervosa	*Peperomia purpureonervosa*	292	0	50	50
Peperomia purpurinervis	*Peperomia purpurinervis*	51	100	0	0
Peperomia purpurinervis	*Peperomia purpurinervis*	52	100	0	0
Peperomia purpurinervis	*Peperomia purpurinervis*	150	33.3	33.3	33.4
Peperomia purpurinervis	*Peperomia purpurinervis*	83	33.3	33.3	33.4
Peperomia purpurinervis	*Peperomia purpurinervis*	373	100	0	0
Peperomia purpurinervis	*Peperomia purpurinervis*	214	100	0	0
Peperomia purpurinervis	*Peperomia purpurinervis*	255	33.3	33.3	33.4
Peperomia purpurinodis	*Peperomia purpurinodis*	198	100	0	0
Peperomia purpurinodis	*Peperomia purpurinodis*	415	100	0	0
Peperomia purpurinodis	*Peperomia purpurinodis*	307	100	0	0
Peperomia purpurinodis	*Peperomia purpurinodis*	255	100	0	0
Peperomia purpurispicata	*Peperomia purpurispicata*	43	50	0	50
Peperomia purpurispicata	*Peperomia purpurispicata*	255	50	0	50
Peperomia pusilla	*Peperomia pusilla*	43	100	0	0
Peperomia pusilla	*Peperomia pusilla*	255	100	0	0
Peperomia putlaensis	*Peperomia putlaensis*	204	0	100	0
Peperomia putumayoensis	*Peperomia putumayoensis*	373	100	0	0
Peperomia putumayoensis	*Peperomia putumayoensis*	255	100	0	0
Peperomia pyramidata	*Peperomia pyramidata*	43	15	0	85
Peperomia pyramidata	*Peperomia pyramidata*	57	100	0	0
Peperomia pyramidata	*Peperomia pyramidata*	219	0	0	100
Peperomia pyramidata	*Peperomia pyramidata*	374	25	0	75
Peperomia pyramidata	*Peperomia pyramidata*	373	0	100	0
Peperomia pyramidata	*Peperomia pyramidata*	255	50	0	50
Peperomia quadrangularis	*Peperomia quadrangularis*	51	50	50	0
Peperomia quadrangularis	*Peperomia quadrangularis*	43	50	50	0
Peperomia quadrangularis	*Peperomia quadrangularis*	57	0	0	100
Peperomia quadrangularis	*Peperomia quadrangularis*	150	50	50	0
Peperomia quadrangularis	*Peperomia quadrangularis*	83	50	50	0
Peperomia quadrangularis	*Peperomia quadrangularis*	373	85	15	0
Peperomia quadrangularis	*Peperomia quadrangularis*	261	85	15	0

Peperomia quadrangularis	*Peperomia quadrangularis*	86	50	50	0
Peperomia quadrangularis	*Peperomia quadrangularis*	294	50	50	0
Peperomia quadrangularis	*Peperomia quadrangularis*	214	100	0	0
Peperomia quadrangularis	*Peperomia muscosa*	42	100	0	0
Peperomia quadrangularis	*Peperomia quadrangularis*	255	50	50	0
Peperomia quadrangularis	*Peperomia angulata*				
Peperomia quadrangularis	*Peperomia atabapoensis*				
Peperomia quadrangularis	*Peperomia pirrisana*	371	100	0	0
Peperomia quadratifolia	*Peperomia quadratifolia*	29	0	0	100
Peperomia quadratifolia	*Peperomia subrenifolia*	373	0	0	100
Peperomia quadratifolia	*Peperomia quadratifolia*	294	50	50	0
Peperomia quadratifolia	*Peperomia quadratifolia*	260	100	0	0
Peperomia quadratifolia	*Peperomia quadratifolia*	255	100	0	0
Peperomia quadratifolia	*Peperomia quadratifolia*	178	100	0	0
Peperomia quadricoma	*Peperomia quadricoma*	29	0	0	100
Peperomia quadricoma	*Peperomia quadricoma*	255	100	0	0
Peperomia quadrifolia	*Peperomia quadrifolia*	24	100	0	0
Peperomia quadrifolia	*Peperomia quadrifolia*	25	100	0	0
Peperomia quadrifolia	*Peperomia quadrifolia*	51	50	50	0
Peperomia quadrifolia	*Peperomia quadrifolia*	43	100	0	0
Peperomia quadrifolia	*Peperomia quadrifolia*	52	100	0	0
Peperomia quadrifolia	*Peperomia quadrifolia*	57	0	0	100
Peperomia quadrifolia	*Peperomia quadrifolia*	110	33.3	33.3	33.4
Peperomia quadrifolia	*Peperomia quadrifolia*	110	100	0	0
Peperomia quadrifolia	*Peperomia quadrifolia*	213	100	0	0
Peperomia quadrifolia	*Peperomia quadrifolia*	219	100	0	0
Peperomia quadrifolia	*Peperomia quadrifolia*	374	91.6	0	8.4
Peperomia quadrifolia	*Peperomia quadrifolia*	83	100	0	0
Peperomia quadrifolia	*Peperomia quadrifolia*	273	50	50	0
Peperomia quadrifolia	*Peperomia quadrifolia*	172	100	0	0
Peperomia quadrifolia	*Peperomia quadrifolia*	328	100	0	0
Peperomia quadrifolia	*Peperomia quadrifolia*	29	0	0	100
Peperomia quadrifolia	*Peperomia schomburgkii*	373	0	0	100
Peperomia quadrifolia	*Peperomia stenocaulis*	373	0	0	100
Peperomia quadrifolia	*Peperomia quadrifolia*	373	100	0	0
Peperomia quadrifolia	*Peperomia portulacifolia*	373	0	0	100
Peperomia quadrifolia	*Peperomia quadrifolia*	220	50	50	0
Peperomia quadrifolia	*Peperomia quadrifolia*	171	100	0	0
Peperomia quadrifolia	*Peperomia quadrifolia*	335	100	0	0
Peperomia quadrifolia	*Peperomia quadrifolia*	47	100	0	0
Peperomia quadrifolia	*Peperomia quadrifolia*	293	100	0	0
Peperomia quadrifolia	*Peperomia quadrifolia*	255	50	50	0
Peperomia quadrifolia	*Peperomia quadrifolia*	37	100	0	0
Peperomia quadrifolia	*Peperomia quadrifolia*	112	50	50	0
Peperomia quadrifolia	*Peperomia quadrifolia*	128	100	0	0
Peperomia quadrifolia	*Peperomia cabaiana*	108	0	100	0
Peperomia quadrifolia	*Peperomia quadrifolia*	90	100	0	0
Peperomia quadrifolia	*Peperomia yaquena*	106	0	100	0
Peperomia quadrifolia	*Peperomia luxii*				
Peperomia quadrifolia	*Peperomia collicola*				
Peperomia quadrifolia	*Peperomia rubripetiola*				
Peperomia quadrifolia	*Peperomia santa-rosana*				
Peperomia quaerata	*Peperomia quaerata*	29	0	0	100
Peperomia quaerata	*Peperomia quaerata*	255	100	0	0
Peperomia quaerata	*Peperomia quaerata*	178	100	0	0
Peperomia quaesita	*Peperomia quaesita*	150	90	0	10
Peperomia quaesita	*Peperomia quaesita*	374	50	0	50
Peperomia quaesita	*Peperomia milvifolia*	29	0	0	100
Peperomia quaesita	*Peperomia quaesita*	29	100	0	0
Peperomia quaesita	*Peperomia santacruzana*	29	0	0	100
Peperomia quaesita	*Peperomia quaesita*	86	50	0	50
Peperomia quaesita	*Peperomia quaesita*	214	100	0	0
Peperomia quaesita	*Peperomia quaesita*	42	50	0	50
Peperomia quaesita	*Peperomia quaesita*	255	50	0	50
Peperomia quaesita	*Peperomia quaesita*	178	100	0	0
Peperomia quaifei	*Peperomia quaifei*				
Peperomia querocochana	*Peperomia querocochana*	292	0	0	100
Peperomia questionis	*Peperomia questionis*	204	0	100	0
Peperomia quetzal	*Peperomia quetzal*	43	0	0	100
Peperomia quetzal	*Peperomia quetzal*	382	50	50	0
Peperomia quimiriana	*Peperomia quimiriana*	29	0	0	100
Peperomia quindioensis	*Peperomia quindioensis*	240	100	0	0
Peperomia quispicanchiana	*Peperomia quispicanchiana*	29	0	0	100
Peperomia racemifolia	*Peperomia racemifolia*	29	0	0	100
Peperomia radiatinervosa	*Peperomia radiatinervosa*	292	0	0	100
Peperomia radicosa	*Peperomia radicosa*	51	100	0	0
Peperomia radicosa	*Peperomia radicosa*	42	100	0	0
Peperomia radicosa	*Peperomia radicosa*	255	100	0	0
Peperomia ramboi	*Peperomia ramboi*	51	0	0	100
Peperomia ramboi	*Peperomia ramboi*	82	0	0	100

Peperomia ranongensis	*Peperomia ranongensis*	342	100	0	0
Peperomia rapensis	*Peperomia rapensis*	115	33.3	33.3	33.4
Peperomia rapensis	*Peperomia rapensis*	255	50	0	50
Peperomia ratticaudata	*Peperomia ratticaudata*	199	50	50	0
Peperomia ratticaudata	*Peperomia ratticaudata*	255	100	0	0
Peperomia rauniensis	*Peperomia rauniensis*	255	100	0	0
Peperomia rechingerae	*Peperomia rechingerae*	269	0	0	100
Peperomia recurvata	*Peperomia vanoverberghii*				
Peperomia reflexa	*Peperomia reflexa*	138	100	0	0
Peperomia reflexa	*Peperomia reflexa*	412	100	0	0
Peperomia reflexa	*Peperomia reflexa*	328	100	0	0
Peperomia reflexa	*Peperomia reflexa*	373	100	0	0
Peperomia reflexa	*Peperomia reflexa*	125	100	0	0
Peperomia reflexa	*Peperomia reflexa*	16	100	0	0
Peperomia reflexa	*Peperomia reflexa*	174	50	50	0
Peperomia reflexa	*Peperomia reflexa*	134	100	0	0
Peperomia reflexa	*Peperomia reflexa*	391	100	0	0
Peperomia reflexa	*Peperomia reflexa*	414	100	0	0
Peperomia reflexa	*Peperomia reflexa*	30	100	0	0
Peperomia reflexa	*Peperomia reflexa*	255	100	0	0
Peperomia reflexa	*Peperomia reflexa*	112	50	50	0
Peperomia reflexa	*Peperomia reflexa*	122	100	0	0
Peperomia reflexa	*Peperomia fontinalis*				
Peperomia regelii	*Peperomia regelii*	51	50	50	0
Peperomia regelii	*Peperomia regelii*	82	50	50	0
Peperomia reineckei	*Peperomia reineckei*	255	50	0	50
Peperomia reineckei	*Peperomia umbricola*				
Peperomia remyi	*Peperomia trichostigma*	73	0	0	100
Peperomia remyi	*Peperomia remyi*	255	100	0	0
Peperomia remyi	*Peperomia remyi*	387	0	50	50
Peperomia renifolia	*Peperomia renifolia*	51	0	0	100
Peperomia renifolia	*Peperomia renifolia*	82	0	0	100
Peperomia renzopalmae	*Peperomia renzopalmae*	248	0	0	100
Peperomia reptans	*Peperomia reptans*	255	50	50	0
Peperomia reptans	*Peperomia duidana*	150	33.3	33.3	33.4
Peperomia reptilis	*Peperomia reptilis*	203	50	0	50
Peperomia reptilis	*Peperomia reptilis*	29	100	0	0
Peperomia reptilis	*Peperomia reptilis*	373	0	0	100
Peperomia reptilis	*Peperomia reptilis*	255	100	0	0
Peperomia reticulata	*Peperomia reticulata*	255	100	0	0
Peperomia retivenulosa	*Peperomia retivenulosa*	51	0	0	100
Peperomia retivenulosa	*Peperomia retivenulosa*	82	0	0	100
Peperomia retivenulosa	*Peperomia retivenulosa*	255	100	0	0
Peperomia retropuberula	*Peperomia retropuberula*	255	100	0	0
Peperomia retusa	*Peperomia retusa*	54	0	0	100
Peperomia retusa	*Peperomia retusa*	114	50	0	50
Peperomia retusa	*Peperomia retusa*	255	100	0	0
Peperomia retusa	*Peperomia retusa*	144	100	0	0
Peperomia retusa	*Peperomia subdichotoma*	29	0	0	100
Peperomia retusa	*Peperomia mannii*	1	100	0	0
Peperomia retusa	*Peperomia retusa*	384	33.3	33.3	33.4
Peperomia retusa	*Peperomia ulugurensis*	16	50	0	50
Peperomia retusa	*Peperomia ulugurensis*	122	0	0	100
Peperomia retusa	*Peperomia retusa*	30	100	0	0
Peperomia retusa	*Peperomia subdichotoma*	178	100	0	0
Peperomia retusa	*Peperomia bueana*	64	100	0	0
Peperomia retusa	*Peperomia retusa*	88	50	50	0
Peperomia retusa	*Peperomia rehmannii*				
Peperomia retusa	*Peperomia ukingensis*				
Peperomia retusa	*Peperomia usambarensis*				
Peperomia retusa	*Peperomia wilmsii*				
Peperomia rhexiifolia	*Peperomia turialvensis*	255	100	0	0
Peperomia rhexiifolia	*Peperomia rhexiifolia*	19	100	0	0
Peperomia rhexiifolia	*Peperomia rhexiifolia*	43	100	0	0
Peperomia rhexiifolia	*Peperomia rhexiifolia*	57	100	0	0
Peperomia rhexiifolia	*Peperomia rhexiifolia*	110	50	0	50
Peperomia rhexiifolia	*Peperomia rhexiifolia*	150	50	50	0
Peperomia rhexiifolia	*Peperomia rhexiifolia*	219	50	0	50
Peperomia rhexiifolia	*Peperomia rhexiifolia*	335	100	0	0
Peperomia rhexiifolia	*Peperomia rhexiifolia*	255	33.3	33.3	33.4
Peperomia rhexiifolia	*Peperomia rhexiifolia*	90	50	50	0
Peperomia rhexiifolia	*Peperomia martagonifolia*				
Peperomia rhexiifolia	*Peperomia carapasana*	29	100	0	0
Peperomia rhexiifolia	*Peperomia carapasana*	373	100	0	0
Peperomia rhexiifolia	*Peperomia tacticana*	328	50	0	50
Peperomia rhexiifolia	*Peperomia wercklei*				
Peperomia rhodophylla	*Peperomia rhodophylla*	29	0	50	50
Peperomia rhombea	*Peperomia rhombea*	51	50	50	0
Peperomia rhombea	*Peperomia rhombea*	31	100	0	0
Peperomia rhombea	*Peperomia rhombea*	43	100	0	0

Peperomia rhombea	*Peperomia rhombea*	150	100	0	0
Peperomia rhombea	*Peperomia rhombea*	21	100	0	0
Peperomia rhombea	*Peperomia rhombea*	83	100	0	0
Peperomia rhombea	*Peperomia rhombea*	172	50	50	0
Peperomia rhombea	*Peperomia rhombea*	29	100	0	0
Peperomia rhombea	*Peperomia rhombea*	373	0	0	100
Peperomia rhombea	*Peperomia rhombea*	171	50	50	0
Peperomia rhombea	*Peperomia rhombea*	381	100	0	0
Peperomia rhombea	*Peperomia rhombea*	335	100	0	0
Peperomia rhombea	*Peperomia rhombea*	15	100	0	0
Peperomia rhombea	*Peperomia rhombea*	294	33.3	33.3	33.4
Peperomia rhombea	*Peperomia rhombea*	42	100	0	0
Peperomia rhombea	*Peperomia rhombea*	260	50	50	0
Peperomia rhombea	*Peperomia rhombea*	255	100	0	0
Peperomia rhombea	*Peperomia rhombea*	37	100	0	0
Peperomia rhombea	*Peperomia rhombea*	191	50	0	50
Peperomia rhombea	*Peperomia myrtillus*	112	100	0	0
Peperomia rhombea	*Peperomia rhombea*	90	100	0	0
Peperomia rhombea	*Peperomia rubioides*				
Peperomia rhombea	*Peperomia filici-decorans*				
Peperomia rhombea	*Peperomia pseudoboliviensis*				
Peperomia rhombeifolia	*Peperomia rhombeifolia*	29	0	0	100
Peperomia rhombeifolia	*Peperomia rhombeifolia*	255	100	0	0
Peperomia rhombeifolia	*Peperomia rhombeifolia*	178	100	0	0
Peperomia rhombeo-elliptica	*Peperomia rhombeo-elliptica*	29	0	0	100
Peperomia rhombeo-elliptica	*Peperomia rhombeo-elliptica*	255	100	0	0
Peperomia rhombeo-elliptica	*Peperomia rhombeo-elliptica*	178	100	0	0
Peperomia rhombifolia	*Peperomia rhombifolia*				
Peperomia rhombiformis	*Peperomia rhombiformis*	29	0	0	100
Peperomia rhombilimba	*Peperomia rhombilimba*	29	0	0	100
Peperomia rhomboidea	*Peperomia rhomboidea*	255	100	0	0
Peperomia ricardofernandezii	*Peperomia ricardofernandezii*	248	0	0	100
Peperomia ricaurtensis	*Peperomia ricaurtensis*	21	100	0	0
Peperomia ricaurtensis	*Peperomia ricaurtensis*	255	100	0	0
Peperomia richardsonii	*Peperomia richardsonii*	201	100	0	0
Peperomia richardsonii	*Peperomia richardsonii*	255	100	0	0
Peperomia ridleyi	*Peperomia ridleyi*	255	100	0	0
Peperomia riedeliana	*Peperomia riedeliana*	51	100	0	0
Peperomia riedeliana	*Peperomia riedeliana*	82	100	0	0
Peperomia rigida	*Peperomia rigida*	237	100	0	0
Peperomia rigidicaulis	*Peperomia rigidicaulis*				
Peperomia rioblancoana	*Peperomia rioblancoana*	373	100	0	0
Peperomia rioblancoana	*Peperomia rioblancoana*	255	100	0	0
Peperomia riocaliensis	*Peperomia riocaliensis*	43	0	0	100
Peperomia riocaliensis	*Peperomia riocaliensis*	373	0	0	100
Peperomia riosaniensis	*Peperomia riosaniensis*	251	0	50	50
Peperomia riparia	*Peperomia riparia*	51	100	0	0
Peperomia riparia	*Peperomia riparia*	82	100	0	0
Peperomia riparia	*Peperomia riparia*	255	100	0	0
Peperomia ripicola	*Peperomia ripicola*	51	0	0	100
Peperomia ripicola	*Peperomia ripicola*	42	0	0	100
Peperomia ripicola	*Peperomia ripicola*	191	50	50	0
Peperomia rivulamans	*Peperomia rivulamans*	198	0	100	0
Peperomia rivulamans	*Peperomia rivulamans*	305	0	100	0
Peperomia rizzinii	*Peperomia rizzinii*	51	50	50	0
Peperomia rizzinii	*Peperomia rizzinii*	82	50	50	0
Peperomia robleana	*Peperomia robleana*	373	0	0	100
Peperomia robusta	*Peperomia robusta*	208	15	0	85
Peperomia robustior	*Peperomia robustior*	172	100	0	0
Peperomia robustior	*Peperomia robustior*	171	100	0	0
Peperomia robustior	*Peperomia robustior*	255	100	0	0
Peperomia rockii	*Peperomia rockii*	387	0	50	50
Peperomia rockii	*Peperomia parvanthera*				
Peperomia rockii	*Peperomia subglabricaulis*	255	0	0	100
Peperomia rodriguesiana	*Peperomia rodriguesiana*				
Peperomia rodriguesiana	*Peperomia rodriguezii*				
Peperomia rosea	*Peperomia rosea*	29	0	0	100
Peperomia rosea	*Peperomia rosea*	255	100	0	0
Peperomia rosea	*Peperomia rosea*	178	100	0	0
Peperomia roseopetiolata	*Peperomia roseopetiolata*	43	0	100	0
Peperomia roseopetiolata	*Peperomia roseopetiolata*	255	0	100	0
Peperomia rossii	*Peperomia rossii*	255	100	0	0
Peperomia rostulatiformis	*Peperomia rostulatiformis*	51	0	100	0
Peperomia rostulatiformis	*Peperomia rostulatiformis*	82	0	100	0
Peperomia rotumaensis	*Peperomia rotumaensis*	315	0	0	100
Peperomia rotundata	*Peperomia rotundata*	19	100	0	0
Peperomia rotundata	*Peperomia rotundata*	51	100	0	0
Peperomia rotundata	*Peperomia rotundata*	39	100	0	0
Peperomia rotundata	*Peperomia rotundata*	57	100	0	0
Peperomia rotundata	*Peperomia rotundata*	150	33.3	33.3	33.4

Peperomia rotundata	*Peperomia rotundata*	233	0	0	100
Peperomia rotundata	*Peperomia quindioensis*	380	0	0	100
Peperomia rotundata	*Peperomia rotundata*	374	33.4	0	66.6
Peperomia rotundata	*Peperomia rotundata*	368	50	0	50
Peperomia rotundata	*Peperomia rotundata*	251	0	0	100
Peperomia rotundata	*Peperomia rotundata*	29	0	50	50
Peperomia rotundata	*Peperomia rotundata*	373	0	0	100
Peperomia rotundata	*Peperomia quindioensis*	373	0	0	100
Peperomia rotundata	*Peperomia ioeides*	373	0	0	100
Peperomia rotundata	*Peperomia rotundata*	255	50	0	50
Peperomia rotundata	*Peperomia anomala*	150	33.3	33.3	33.4
Peperomia rotundata	*Peperomia epilobioides*	368	0	0	100
Peperomia rotundata	*Peperomia rotundata*	90	33.3	33.3	33.4
Peperomia rotundata	*Peperomia moritzii*				
Peperomia rotundata	*Peperomia purpurascens*				
Peperomia rotundifolia	*Peperomia rotundifolia*	18	100	0	0
Peperomia rotundifolia	*Peperomia rotundifolia*	20	100	0	0
Peperomia rotundifolia	*Peperomia rotundifolia*	25	100	0	0
Peperomia rotundifolia	*Peperomia rotundifolia*	51	100	0	0
Peperomia rotundifolia	*Peperomia rotundifolia*	43	100	0	0
Peperomia rotundifolia	*Peperomia rotundifolia*	53	100	0	0
Peperomia rotundifolia	*Peperomia rotundifolia*	56	100	0	0
Peperomia rotundifolia	*Peperomia rotundifolia*	57	0	0	100
Peperomia rotundifolia	*Peperomia rotundifolia*	60	100	0	0
Peperomia rotundifolia	*Peperomia rotundifolia*	110	100	0	0
Peperomia rotundifolia	*Peperomia rotundifolia*	114	85	0	15
Peperomia rotundifolia	*Peperomia rotundifolia*	150	50	50	0
Peperomia rotundifolia	*Peperomia rotundifolia*	213	100	0	0
Peperomia rotundifolia	*Peperomia rotundifolia*	219	100	0	0
Peperomia rotundifolia	*Peperomia rotundifolia*	227	50	50	0
Peperomia rotundifolia	*Peperomia rotundifolia*	275	100	0	0
Peperomia rotundifolia	*Peperomia rotundifolia*	374	85	0	15
Peperomia rotundifolia	*Peperomia rotundifolia*	21	100	0	0
Peperomia rotundifolia	*Peperomia rotundifolia*	83	50	50	0
Peperomia rotundifolia	*Peperomia rotundifolia*	138	100	0	0
Peperomia rotundifolia	*Peperomia rotundifolia*	273	100	0	0
Peperomia rotundifolia	*Peperomia rotundifolia*	412	70	0	30
Peperomia rotundifolia	*Peperomia rotundifolia*	172	100	0	0
Peperomia rotundifolia	*Peperomia rotundifolia*	144	100	0	0
Peperomia rotundifolia	*Peperomia rotundifolia*	328	100	0	0
Peperomia rotundifolia	*Peperomia rotundifolia*	29	100	0	0
Peperomia rotundifolia	*Peperomia guayaquilensis*	373	0	0	100
Peperomia rotundifolia	*Peperomia rotundifolia*	373	30	0	70
Peperomia rotundifolia	*Peperomia rotundifolia*	384	70	30	0
Peperomia rotundifolia	*Peperomia rotundifolia*	220	100	0	0
Peperomia rotundifolia	*Peperomia rotundifolia*	171	100	0	0
Peperomia rotundifolia	*Peperomia rotundifolia*	91	50	50	0
Peperomia rotundifolia	*Peperomia rotundifolia*	381	100	0	0
Peperomia rotundifolia	*Peperomia rotundifolia*	134	50	50	0
Peperomia rotundifolia	*Peperomia rotundifolia*	335	100	0	0
Peperomia rotundifolia	*Peperomia rotundifolia*	15	100	0	0
Peperomia rotundifolia	*Peperomia rotundifolia*	294	70	30	0
Peperomia rotundifolia	*Peperomia rotundifolia*	221	100	0	0
Peperomia rotundifolia	*Peperomia rotundifolia*	214	100	0	0
Peperomia rotundifolia	*Peperomia rotundifolia*	42	100	0	0
Peperomia rotundifolia	*Peperomia rotundifolia*	85	100	0	0
Peperomia rotundifolia	*Peperomia rotundifolia*	418	100	0	0
Peperomia rotundifolia	*Peperomia rotundifolia*	224	100	0	0
Peperomia rotundifolia	*Peperomia rotundifolia*	414	15	0	85
Peperomia rotundifolia	*Peperomia rotundifolia*	30	15	0	85
Peperomia rotundifolia	*Peperomia rotundifolia*	260	33.3	33.3	33.4
Peperomia rotundifolia	*Peperomia rotundifolia*	255	50	50	0
Peperomia rotundifolia	*Peperomia rotundifolia*	252	100	0	0
Peperomia rotundifolia	*Peperomia rotundifolia*	178	100	0	0
Peperomia rotundifolia	*Peperomia rotundifolia*	37	100	0	0
Peperomia rotundifolia	*Peperomia rotundifolia*	191	100	0	0
Peperomia rotundifolia	*Peperomia rotundifolia*	325	85	0	15
Peperomia rotundifolia	*Peperomia rotundifolia*	276	100	0	0
Peperomia rotundifolia	*Peperomia bartlettii*	373	100	0	0
Peperomia rotundifolia	*Peperomia nummularifolia*	134	50	0	50
Peperomia rotundifolia	*Peperomia rotundifolia f. ovata*	86	50	50	0
Peperomia rotundifolia	*Peperomia koepperi*	408	100	0	0
Peperomia rotundifolia	*Peperomia lanjouwii*	261	0	0	100
Peperomia rotundifolia	*Peperomia rotundifolia*	88	50	50	0
Peperomia rotundifolia	*Peperomia rotundifolia*	375	100	0	0
Peperomia rotundifolia	*Peperomia mararyna*				
Peperomia rotundilimba	*Peperomia rotundilimba*	255	100	0	0
Peperomia roxburghiana	*Peperomia roxburghiana*				
Peperomia roxburghiana	*Peperomia angustifolia*				
Peperomia rubea	*Peperomia rubea*	29	0	0	100

Peperomia rubea	*Peperomia rubea*	178	0	0	100
Peperomia rubens	*Peperomia rubens*	29	100	0	0
Peperomia rubescens	*Peperomia rubescens*	29	0	0	100
Peperomia rubescens	*Peperomia rubescens*	255	100	0	0
Peperomia rubramenta	*Peperomia rubramenta*	373	0	0	100
Peperomia rubricaulis	*Peperomia rubricaulis*	51	50	50	0
Peperomia rubricaulis	*Peperomia rubricaulis*	80	0	100	0
Peperomia rubricaulis	*Peperomia rubricaulis*	220	0	100	0
Peperomia rubricaulis	*Peperomia rubricaulis*	189	50	50	0
Peperomia rubricaulis	*Peperomia rubricaulis*	84	0	100	0
Peperomia rubricaulis	*Peperomia rubricaulis*	85	50	50	0
Peperomia rubricaulis	*Peperomia rubricaulis*	161	0	50	50
Peperomia rubricaulis	*Peperomia rubricaulis*	418	0	50	50
Peperomia rubricaulis	*Peperomia rubricaulis*	255	50	0	50
Peperomia rubricaulis	*Peperomia pereirae*	51	50	50	0
Peperomia rubricaulis	*Peperomia pereirae*	82	50	50	0
Peperomia rubricaulis	*Peperomia pereirae*	255	100	0	0
Peperomia rubricaulis	*Peperomia rubricaulis*	375	100	0	0
Peperomia rubricaulis	*Peperomia longiflora*				
Peperomia rubrifolia	*Peperomia rubrifolia*	29	0	0	100
Peperomia rubrimaculata	*Peperomia rubrimaculata*	255	100	0	0
Peperomia rubrinodis	*Peperomia rubrinodis*				
Peperomia rubrivenosa	*Peperomia rubrivenosa*	27	50	50	0
Peperomia rubrivenosa	*Peperomia rubrivenosa*	238	50	50	0
Peperomia rubrivenosa	*Peperomia rubrivenosa*	174	50	50	0
Peperomia rubrivenosa	*Peperomia rubrivenosa*	255	50	50	0
Peperomia rubrivenosa	*Peperomia macgregorii*				
Peperomia rubrivenosa	*Peperomia rivulorum*	70	0	100	0
Peperomia rubropunctulata	*Peperomia rubropunctulata*	373	0	0	100
Peperomia rubropunctulata	*Peperomia rubropunctulata*	255	100	0	0
Peperomia rufescens	*Peperomia rufescens*	29	0	0	100
Peperomia rufescentifolia	*Peperomia rufescentifolia*	29	0	0	100
Peperomia rufispica	*Peperomia rufispica*	51	100	0	0
Peperomia rufispica	*Peperomia rufispica*	82	100	0	0
Peperomia rufispica	*Peperomia rufispica*	255	100	0	0
Peperomia rugatifolia	*Peperomia rugatifolia*	29	0	0	100
Peperomia rugatifolia	*Peperomia rugatifolia*	255	100	0	0
Peperomia rugatifolia	*Peperomia rugatifolia*	178	100	0	0
Peperomia rugosa	*Peperomia rugosa*	255	100	0	0
Peperomia rupicola	*Peperomia rupicola*	373	0	0	100
Peperomia rupicola	*Peperomia rupicola*	65	0	100	0
Peperomia rupigaudens	*Peperomia rupigaudens*	110	10	90	0
Peperomia rupigaudens	*Peperomia rupigaudens*	255	100	0	0
Peperomia rupigaudens	*Peperomia rupigaudens*	78	0	100	0
Peperomia rupiseda	*Peperomia rupiseda*	29	0	0	100
Peperomia rurrenabaqueana	*Peperomia rurrenabaqueana*	412	100	0	0
Peperomia rurrenabaqueana	*Peperomia rurrenabaqueana*	255	100	0	0
Peperomia rusbyi	*Peperomia rusbyi*	255	100	0	0
Peperomia rusbyi	*Peperomia rusbyi*	348	0	0	100
Peperomia ruscifolia	*Peperomia ruscifolia*	255	100	0	0
Peperomia sabaletasana	*Peperomia sabaletasana*	255	100	0	0
Peperomia sachatzinzumba	*Peperomia sachatzinzumba*	374	42.8	0	57.2
Peperomia sagasteguii	*Peperomia sagasteguii*	251	33.3	33.3	33.3
Peperomia sagittata	*Peperomia sagittata*	202	100	0	0
Peperomia sagittata	*Peperomia sagittata*	255	100	0	0
Peperomia saintpauliella	*Peperomia saintpauliella*	43	0	50	50
Peperomia saintpauliella	*Peperomia saintpauliella*	255	34	33	33
Peperomia saintpauliella	*Peperomia saintpauliella*	132	0	50	50
Peperomia salaminana	*Peperomia salaminana*	373	0	0	100
Peperomia salangonis	*Peperomia salangonis*	373	0	0	100
Peperomia salicifolia	*Peperomia salicifolia*	255	100	0	0
Peperomia saligna	*Peperomia saligna*	43	15	15	70
Peperomia saligna	*Peperomia saligna*	57	0	0	100
Peperomia saligna	*Peperomia saligna*	110	50	0	50
Peperomia saligna	*Peperomia saligna*	150	50	0	50
Peperomia saligna	*Peperomia saligna*	219	50	0	50
Peperomia saligna	*Peperomia saligna*	380	0	0	100
Peperomia saligna	*Peperomia saligna*	374	36	0	64
Peperomia saligna	*Peperomia saligna*	21	100	0	0
Peperomia saligna	*Peperomia saligna*	368	50	0	50
Peperomia saligna	*Peperomia saligna*	373	50	0	50
Peperomia saligna	*Peperomia saligna*	255	50	0	50
Peperomia saligna	*Peperomia saligna*	37	50	0	50
Peperomia saligna	*Peperomia aspergillus*	198	100	0	0
Peperomia saligna	*Peperomia gentianifolia*				
Peperomia salmonicolor	*Peperomia salmonicolor*	29	0	0	100
Peperomia salmonicolor	*Peperomia salmonicolor*	255	100	0	0
Peperomia salmonicolor	*Peperomia salmonicolor*	178	100	0	0
Peperomia samainiae	*Peperomia samainiae*	247	0	50	50
Peperomia samoensis	*Peperomia samoensis*				

Peperomia samoensis	*Peperomia cililimba*				
Peperomia sanblasensis	*Peperomia sanblasensis*	43	100	0	0
Peperomia sanblasensis	*Peperomia sanblasensis*	255	100	0	0
Peperomia san-buenaventurana	*Peperomia san-buenaventurana*	29	0	0	100
Peperomia san-carlosiana	*Peperomia cooperi*	37	33.3	33.3	33.4
Peperomia san-carlosiana	*Peperomia san-carlosiana*	43	0	50	50
Peperomia san-carlosiana	*Peperomia san-carlosiana*	110	10	45	45
Peperomia san-carlosiana	*Peperomia san-carlosiana*	150	0	100	0
Peperomia san-carlosiana	*Peperomia san-carlosiana*	373	0	0	100
Peperomia san-carlosiana	*Peperomia san-carlosiana*	335	70	15	15
Peperomia san-carlosiana	*Peperomia san-carlosiana*	255	50	50	0
Peperomia san-carlosiana	*Peperomia praeteruentifolia*	328	15	0	85
Peperomia san-carlosiana	*Peperomia praeteruentifolia*	404	0	0	100
Peperomia san-carlosiana	*Peperomia filispica*				
Peperomia san-carlosiana	*Peperomia yunckeri*				
Peperomia sandemanii	*Peperomia sandemanii*	29	0	0	100
Peperomia sandemanii	*Peperomia sandemanii*	411	0	0	100
Peperomia sandwicensis	*Peperomia sandwicensis*	255	100	0	0
Peperomia sandwicensis	*Peperomia pachyphylla*				
Peperomia san-felipensis	*Peperomia san-felipensis*	43	100	0	0
Peperomia san-felipensis	*Peperomia san-felipensis*	110	100	0	0
Peperomia san-felipensis	*Peperomia san-felipensis*	255	100	0	0
Peperomia sangabanensis	*Peperomia sangabanensis*	29	0	0	100
Peperomia san-joseana	*Peperomia coarctata*	328	95	0	5
Peperomia san-joseana	*Peperomia san-joseana*	43	50	0	50
Peperomia san-joseana	*Peperomia san-joseana*	110	90	10	0
Peperomia san-joseana	*Peperomia san-joseana*	255	34	33	33
Peperomia san-joseana	*Peperomia san-joseana*	90	50	0	50
Peperomia san-joseana	*Peperomia rivi-vetusti*	4	100	0	0
Peperomia san-joseana	*Peperomia pascuicola*	219	50	0	50
Peperomia san-joseana	*Peperomia storkii*	326	100	0	0
Peperomia san-joseana	*Peperomia siguatepequensis*	324	0	0	100
Peperomia san-joseana	*Peperomia pennellii*	373	33.3	33.3	33.4
Peperomia san-joseana	*Peperomia pennellii*	164	100	0	0
Peperomia san-joseana	*Peperomia nebuligaudens*	400	100	0	0
Peperomia san-joseana	*Peperomia mala*	398	100	0	0
Peperomia san-joseana	*Peperomia munyecoana*				
Peperomia san-joseana	*Peperomia psiloclada*				
Peperomia san-joseana	*Peperomia chrysocarpa*				
Peperomia san-joseana	*Peperomia incrassata*				
Peperomia san-joseana	*Peperomia leucosticta*				
Peperomia san-joseana	*Peperomia appellator*				
Peperomia san-joseana	*Peperomia argumentosa*				
Peperomia san-joseana	*Peperomia brachypus*				
Peperomia san-joseana	*Peperomia tressis*				
Peperomia sanquininiana	*Peperomia sanquininiana*	373	0	0	100
Peperomia sanquininiana	*Peperomia sanquininiana*	255	100	0	0
Peperomia san-roqueana	*Peperomia san-roqueana*	29	0	0	100
Peperomia san-roqueana	*Peperomia san-roqueana*	178	100	0	0
Peperomia sansalvadorana	*Peperomia sansalvadorana*	255	100	0	0
Peperomia santa-elisae	*Peperomia santa-elisae*	418	0	50	50
Peperomia santa-elisae	*Peperomia lilloi*	412	0	0	100
Peperomia santa-elisae	*Peperomia lilloi*	375	0	100	0
Peperomia santa-helenea	*Peperomia santa-helenea*	43	50	50	0
Peperomia santa-helenea	*Peperomia santa-helenea*	110	90	0	10
Peperomia santa-helenea	*Peperomia santa-helenea*	328	95	0	5
Peperomia santa-helenea	*Peperomia santa-helenea*	255	50	50	0
Peperomia santanderana	*Peperomia santanderana*	373	0	50	50
Peperomia santiagoana	*Peperomia santiagoana*	29	0	0	100
Peperomia santiagoana	*Peperomia santiagoana*	255	100	0	0
Peperomia santiagoana	*Peperomia santiagoana*	191	50	0	50
Peperomia sarasinii	*Peperomia sarasinii*	298	0	100	0
Peperomia saxicola	*Peperomia saxicola*	29	0	0	100
Peperomia saxicola	*Peperomia saxicola*	185	0	100	0
Peperomia scabiosa	*Peperomia scabiosa*	29	0	0	100
Peperomia scabiosa	*Peperomia scabiosa*	255	100	0	0
Peperomia scabiosa	*Peperomia scabiosa*	178	100	0	0
Peperomia schenckeana	*Peperomia schenckeana*				
Peperomia schenkiana	*Peperomia schenkiana*	255	100	0	0
Peperomia schiedei	*Peperomia schiedei*	110	100	0	0
Peperomia schizandra	*Peperomia schizandra*	198	0	50	50
Peperomia schlechteri	*Peperomia schlechteri*				
Peperomia schmidtii	*Peperomia schmidtii*	64	0	100	0
Peperomia schneepeana	*Peperomia schneepeana*				
Peperomia schomburgkii	*Peperomia schomburgkii*				
Peperomia schultzei	*Peperomia schultzei*	373	0	0	100
Peperomia schultzei	*Peperomia schultzei*	255	100	0	0
Peperomia schunkeana	*Peperomia schunkeana*	29	0	0	100
Peperomia schwackei	*Peperomia schwackei*	51	100	0	0

Peperomia schwackei	*Peperomia schwackei*	82	100	0	0
Peperomia schwackei	*Peperomia schwackei*	255	100	0	0
Peperomia sclerophylla	*Peperomia sclerophylla*	29	0	0	100
Peperomia scopulorum	*Peperomia scopulorum*	370	0	100	0
Peperomia scutaleifolia	*Peperomia scutaleifolia*	29	0	0	100
Peperomia scutaleifolia	*Peperomia scutaleifolia*	255	100	0	0
Peperomia scutaleifolia	*Peperomia scutaleifolia*	178	100	0	0
Peperomia scutellariifolia	*Peperomia scutellariifolia*	374	0	0	100
Peperomia scutellariifolia	*Peperomia scutellariifolia*	373	0	0	100
Peperomia scutellifolia	*Peperomia scutellifolia*	29	0	50	50
Peperomia scutellifolia	*Peperomia scutellifolia*				
Peperomia scutifolia	*Peperomia scutifolia*	51	0	0	100
Peperomia scutifolia	*Peperomia scutifolia*	42	50	0	50
Peperomia scutifolia	*Peperomia scutifolia*	255	100	0	0
Peperomia scutilimba	*Peperomia scutilimba*	412	0	0	100
Peperomia secunda	*Peperomia secunda*	29	0	0	100
Peperomia secunda	*Peperomia secundiflora*				
Peperomia seemanniana	*Peperomia seemanniana*	43	100	0	0
Peperomia seemanniana	*Peperomia seemanniana*	57	0	0	100
Peperomia seemanniana	*Peperomia seemanniana*	255	100	0	0
Peperomia seemanniana	*Peperomia seemanniana*	37	100	0	0
Peperomia segregata	*Peperomia segregata*	51	0	0	100
Peperomia segregata	*Peperomia segregata*	63	5	0	95
Peperomia segregata	*Peperomia segregata*	82	0	0	100
Peperomia seibertii	*Peperomia seibertii*	43	0	0	100
Peperomia seibertii	*Peperomia seibertii*	255	100	0	0
Peperomia selenophylla	*Peperomia selenophylla*	247	0	50	50
Peperomia seleri	*Peperomia seleri*	29	0	0	100
Peperomia seleri	*Peperomia seleri*	178	0	50	50
Peperomia semimetralis	*Peperomia semimetralis*	75	0	0	100
Peperomia semipuberula	*Peperomia semipuberula*	373	0	0	100
Peperomia semipuberula	*Peperomia semipuberula*	255	100	0	0
Peperomia seposita	*Peperomia seposita*	29	0	0	100
Peperomia septemnervis	*Peperomia septemnervis*	29	100	0	0
Peperomia septemnervis	*Peperomia septemnervis*	294	42.5	15	42.5
Peperomia septemnervis	*Peperomia septemnervis*	255	100	0	0
Peperomia septemnervis	*Peperomia septemnervis*	112	33.3	55.3	11.4
Peperomia septemnervis	*Peperomia montefrionis*				
Peperomia septentrionalis	*Peperomia septentrionalis*	35	0	100	0
Peperomia serpens	*Peperomia serpens*	51	50	50	0
Peperomia serpens	*Peperomia serpens*	57	0	0	100
Peperomia serpens	*Peperomia serpens*	150	50	50	0
Peperomia serpens	*Peperomia serpens*	158	50	0	50
Peperomia serpens	*Peperomia serpens*	219	0	0	100
Peperomia serpens	*Peperomia serpens*	227	50	50	0
Peperomia serpens	*Peperomia serpens*	374	100	0	0
Peperomia serpens	*Peperomia serpens*	21	100	0	0
Peperomia serpens	*Peperomia serpens*	83	50	50	0
Peperomia serpens	*Peperomia serpens*	412	50	0	50
Peperomia serpens	*Peperomia scandens*	29	0	0	100
Peperomia serpens	*Peperomia serpens*	29	33.3	33.3	33.4
Peperomia serpens	*Peperomia serpens*	373	50	0	50
Peperomia serpens	*Peperomia serpens*	261	50	0	50
Peperomia serpens	*Peperomia serpens*	171	0	0	100
Peperomia serpens	*Peperomia serpens*	381	100	0	0
Peperomia serpens	*Peperomia serpens*	86	50	50	0
Peperomia serpens	*Peperomia serpens*	335	100	0	0
Peperomia serpens	*Peperomia serpens*	294	33.3	33.3	33.4
Peperomia serpens	*Peperomia serpens*	153	100	0	0
Peperomia serpens	*Peperomia serpens*	214	100	0	0
Peperomia serpens	*Peperomia scandens*	42	100	0	0
Peperomia serpens	*Peperomia serpens*	42	100	0	0
Peperomia serpens	*Peperomia serpens*	255	50	50	0
Peperomia serpens	*Peperomia serpens*	37	100	0	0
Peperomia serpens	*Peperomia serpens*	112	50	50	0
Peperomia serpens	*Peperomia serpens*	191	33.3	33.3	33.4
Peperomia serpens	*Peperomia serpens*	276	100	0	0
Peperomia serpens	*Peperomia ayacuchana*	29	100	0	0
Peperomia serpens	*Peperomia ionophylla*				
Peperomia serpentarioides	*Peperomia serpentarioides*	51	0	0	100
Peperomia serpentarioides	*Peperomia serpentarioides*	82	0	0	100
Peperomia serratirhachis	*Peperomia serratirhachis*	51	0	50	50
Peperomia serratirhachis	*Peperomia serratirhachis*	82	0	50	50
Peperomia sierpeana	*Peperomia sierpeana*	43	0	100	0
Peperomia sierpeana	*Peperomia sierpeana*	255	0	100	0
Peperomia silvarum	*Peperomia silvarum*	355	100	0	0
Peperomia silvicola	*Peperomia silvicola*	255	100	0	0
Peperomia silvivaga	*Peperomia silvivaga*	43	100	0	0
Peperomia silvivaga	*Peperomia silvivaga*	219	100	0	0
Peperomia silvivaga	*Peperomia silvivaga*	255	100	0	0

Peperomia silvivaga	*Peperomia albescens*	6	100	0	0
Peperomia silvivaga	*Peperomia montium*	57	100	0	0
Peperomia silvivaga	*Peperomia montium*	150	100	0	0
Peperomia silvivaga	*Peperomia montium*	335	100	0	0
Peperomia silvivaga	*Peperomia panamensis*	373	100	0	0
Peperomia silvivaga	*Peperomia panamensis*	37	100	0	0
Peperomia silvivaga	*Peperomia calyculata*	198	100	0	0
Peperomia silvivaga	*Peperomia megalanthera*				
Peperomia silvivaga	*Peperomia sanramonensis*				
Peperomia silvivaga	*Peperomia congestifolia*				
Peperomia silvivaga	*Peperomia cryptolepida*				
Peperomia silvivaga	*Peperomia delecta*				
Peperomia silvivaga	*Peperomia saltivagans*				
Peperomia simplex	*Peperomia simplex*	259	0	50	50
Peperomia simplex	*Peperomia hamiltoniana*	152	0	50	50
Peperomia simulans	*Peperomia simulans*	82	0	0	100
Peperomia simulans	*Peperomia simulans*	42	0	0	100
Peperomia simuliformis	*Peperomia simuliformis*	43	0	0	100
Peperomia sincorana	*Peperomia sincorana*	51	0	100	0
Peperomia sincorana	*Peperomia sincorana*	82	0	100	0
Peperomia sincorana	*Peperomia dincorana*				
Peperomia sintenesii	*Peperomia sintenesii*				
Peperomia sirindhorniana	*Peperomia sirindhorniana*	341	0	100	0
Peperomia sirindhorniana	*Peperomia sirindhorniana*	342	0	100	0
Peperomia sirupayana	*Peperomia sirupayana*	255	100	0	0
Peperomia skottsbergii	*Peperomia skottsbergii*	412	0	0	100
Peperomia skottsbergii	*Peperomia skottsbergii*	29	0	0	100
Peperomia skottsbergii	*Peperomia skottsbergii*	306	0	100	0
Peperomia smithiana	*Peperomia smithiana*	153	100	0	0
Peperomia smithii	*Peperomia smithii*	29	0	0	100
Peperomia smithii	*Peperomia smithii*	255	100	0	0
Peperomia smithii	*Peperomia smithii*	178	100	0	0
Peperomia sneidernii	*Peperomia sneidernii*	374	83.3	0	16.7
Peperomia sneidernii	*Peperomia sneidernii*	373	0	0	100
Peperomia sneidernii	*Peperomia sneidernii*	255	100	0	0
Peperomia societatis	*Peperomia societatis*	115	50	50	0
Peperomia societatis	*Peperomia societatis*	255	50	50	0
Peperomia societatis	*Peperomia boraborensis*	198	100	0	0
Peperomia societatis	*Peperomia raiateensis*	225	0	100	0
Peperomia societatis	*Peperomia huahinensis*				
Peperomia socorronis	*Peperomia socorronis*	110	100	0	0
Peperomia socorronis	*Peperomia socorronis*	255	100	0	0
Peperomia sodiroi	*Peperomia sodiroi*	374	0	0	100
Peperomia sodiroi	*Peperomia sodiroi*	373	0	0	100
Peperomia soukupii	*Peperomia soukupii*	29	0	0	100
Peperomia soukupii	*Peperomia soukupii*	255	100	0	0
Peperomia spathophylla	*Peperomia spathophylla*	294	0	0	100
Peperomia spathophylla	*Peperomia spathophylla*	255	100	0	0
Peperomia spathulifolia	*Peperomia spathulifolia*	34	0	0	100
Peperomia spathulifolia	*Peperomia lancetillana*				
Peperomia spathulifolia	*Peperomia yousei*				
Peperomia sphaerostachya	*Peperomia sphaerostachya*	207	0	0	100
Peperomia spiculata	*Peperomia spiculata*	29	0	0	100
Peperomia spiritus-sancti	*Peperomia spiritus-sancti*	51	0	100	0
Peperomia spiritus-sancti	*Peperomia spiritus-sancti*	50	0	100	0
Peperomia spiritus-sancti	*Peperomia spiritus-sancti*	82	0	100	0
Peperomia spruceana	*Peperomia spruceana*	51	0	0	100
Peperomia spruceana	*Peperomia spruceana*	150	0	0	100
Peperomia spruceana	*Peperomia spruceana*	42	0	0	100
Peperomia sprucei	*Peperomia sprucei*	29	0	0	100
Peperomia sprucei	*Peperomia sprucei*	191	50	50	0
Peperomia steinbachii	*Peperomia steinbachii*	359	0	100	0
Peperomia stelechophila	*Peperomia roseocaulis*	373	0	0	100
Peperomia stelechophila	*Peperomia stelechophila*	374	42.1	5.2	52.7
Peperomia stelechophila	*Peperomia stelechophila*	255	100	0	0
Peperomia stellata	*Peperomia stellata*	255	100	0	0
Peperomia stellata	*Peperomia stellata*	112	0	50	50
Peperomia stellata	*Peperomia rhomboides*				
Peperomia stellata	*Peperomia turfosa*				
Peperomia stenostachya	*Peperomia stenostachya*	373	0	0	100
Peperomia stevensii	*Peperomia stevensii*	369	100	0	0
Peperomia steyermarkii	*Peperomia steyermarkii*	150	0	100	0
Peperomia steyermarkii	*Peperomia steyermarkii*	255	0	100	0
Peperomia stilifera	*Peperomia stilifera*	29	0	0	100
Peperomia stilifera	*Peperomia stilifera*	373	0	0	100
Peperomia stilifera	*Peperomia stilifera*	191	0	0	100
Peperomia stipitifolia	*Peperomia stipitifolia*	43	50	0	50
Peperomia stipitifolia	*Peperomia stipitifolia*	255	50	0	50
Peperomia stolonifera	*Peperomia stolonifera*	373	0	0	100
Peperomia stolonifera	*Peperomia stolonifera*	255	100	0	0

Peperomia strawii	*Peperomia strawii*	29	0	0	100
Peperomia strawii	*Peperomia strawii*	243	0	0	100
Peperomia striata	*Peperomia striata*	39	100	0	0
Peperomia striata	*Peperomia striata*	43	15	0	85
Peperomia striata	*Peperomia striata*	150	50	0	50
Peperomia striata	*Peperomia striata*	219	50	0	50
Peperomia striata	*Peperomia striata*	374	33.4	0	66.6
Peperomia striata	*Peperomia striata*	21	100	0	0
Peperomia striata	*Peperomia frigidula*	328	100	0	0
Peperomia striata	*Peperomia spectabilis*	29	0	0	100
Peperomia striata	*Peperomia helminthostachya*	373	0	0	100
Peperomia striata	*Peperomia striata*	255	33.3	33.3	33.4
Peperomia striata	*Peperomia striata*	191	50	0	50
Peperomia striata	*Peperomia omnicola*	233	0	0	100
Peperomia striata	*Peperomia omnicola*	29	0	0	100
Peperomia striata	*Peperomia omnicola*	373	50	0	50
Peperomia striata	*Peperomia omnicola*	37	50	0	50
Peperomia striata	*Peperomia pothifolia*	320	100	0	0
Peperomia striata	*Peperomia substriata*	57	0	0	100
Peperomia striata	*Peperomia huitzensis*				
Peperomia stroemfeltii	*Peperomia stroemfeltii*	51	100	0	0
Peperomia stroemfeltii	*Peperomia stroemfeltii*	82	50	50	0
Peperomia stroemfeltii	*Peperomia stroemfeltii*	255	100	0	0
Peperomia stuebelii	*Peperomia stuebelii*	412	0	0	100
Peperomia stuebelii	*Peperomia stuebelii*	356	0	100	0
Peperomia subalata	*Peperomia subalata*	373	0	0	100
Peperomia subamplexicaulis	*Peperomia subamplexicaulis*				
Peperomia subandina	*Peperomia subandina*	29	100	0	0
Peperomia subandina	*Peperomia subandina*	255	100	0	0
Peperomia subblanda	*Peperomia subblanda*	43	100	0	0
Peperomia subblanda	*Peperomia subblanda*	110	100	0	0
Peperomia subblanda	*Peperomia subblanda*	293	0	0	100
Peperomia subblanda	*Peperomia subblanda*	255	100	0	0
Peperomia subcalvescens	*Peperomia subcalvescens*	29	0	0	100
Peperomia subelongata	*Peperomia subelongata*	255	100	0	0
Peperomia subemarginata	*Peperomia subemarginata*	51	100	0	0
Peperomia subemarginata	*Peperomia subemarginata*	82	100	0	0
Peperomia subemarginata	*Peperomia subemarginata*	255	100	0	0
Peperomia subemarginulata	*Peperomia subemarginulata*				
Peperomia subflaccida	*Peperomia subflaccida*	29	0	0	100
Peperomia subpallescens	*Peperomia subpallescens*	255	100	0	0
Peperomia subpetiolata	*Peperomia subpetiolata*	387	0	0	100
Peperomia subpilosa	*Peperomia subpilosa*	51	100	0	0
Peperomia subpilosa	*Peperomia subpilosa*	82	100	0	0
Peperomia subpilosa	*Peperomia subpilosa*	255	100	0	0
Peperomia subrenifolia	*Peperomia subrenifolia*				
Peperomia subretusa	*Peperomia subretusa*	51	100	0	0
Peperomia subretusa	*Peperomia subretusa*	82	100	0	0
Peperomia subretusa	*Peperomia subretusa*	255	100	0	0
Peperomia subroseispica	*Peperomia subroseispica*	143	0	100	0
Peperomia subroseispica	*Peperomia subroseispica*	307	85	15	0
Peperomia subroseispica	*Peperomia subroseispica*	255	100	0	0
Peperomia subroseispica	*Peperomia flexuosa*				
Peperomia subrotundifolia	*Peperomia subrotundifolia*	294	0	50	50
Peperomia subrotundifolia	*Peperomia subrotundifolia*	255	100	0	0
Peperomia subrubescens	*Peperomia subrubescens*	43	0	0	100
Peperomia subrubescens	*Peperomia subrubescens*	374	50	0	50
Peperomia subrubescens	*Peperomia subrubescens*	373	0	50	50
Peperomia subrubricaulis	*Peperomia subrubricaulis*	51	100	0	0
Peperomia subrubricaulis	*Peperomia subrubricaulis*	82	50	50	0
Peperomia subrubricaulis	*Peperomia subrubricaulis*	255	100	0	0
Peperomia subrubricaulis	*Peperomia subrubricaulis*	47	0	0	100
Peperomia subrubricaulis	*Peperomia subrubricaulis*	48	0	50	50
Peperomia subrubrispica	*Peperomia subrubrispica*	51	0	100	0
Peperomia subrubrispica	*Peperomia subrubrispica*	82	0	100	0
Peperomia subrubrispica	*Peperomia subrubrispica*	179	0	100	0
Peperomia subrubrispica	*Peperomia subrubrispica*	47	0	100	0
Peperomia subsericata	*Peperomia subsericata*	29	100	0	0
Peperomia subsericata	*Peperomia subsericata*	255	100	0	0
Peperomia subsericata	*Peperomia subsericata*	178	100	0	0
Peperomia subsetifolia	*Peperomia subsetifolia*	255	100	0	0
Peperomia subspathulata	*Peperomia subspathulata*	368	0	0	100
Peperomia subspathulata	*Peperomia subspathulata*	373	0	0	100
Peperomia subternifolia	*Peperomia subternifolia*	51	50	50	0
Peperomia subternifolia	*Peperomia subternifolia*	82	50	0	50
Peperomia subternifolia	*Peperomia subternifolia*	255	100	0	0
Peperomia subternifolia	*Peperomia subternifolia*	220	50	0	50
Peperomia subvillicaulis	*Peperomia subvillicaulis*	29	0	0	100
Peperomia subvillicaulis	*Peperomia subvillicaulis*	178	100	0	0
Peperomia succulenta	*Peperomia succulenta*	43	100	0	0

Peperomia succulenta	*Peperomia succulenta*	52	100	0	0
Peperomia succulenta	*Peperomia succulenta*	57	50	0	50
Peperomia succulenta	*Peperomia succulenta*	110	100	0	0
Peperomia succulenta	*Peperomia succulenta*	150	100	0	0
Peperomia succulenta	*Peperomia succulenta*	219	100	0	0
Peperomia succulenta	*Peperomia succulenta*	335	50	0	50
Peperomia succulenta	*Peperomia succulenta*	255	33.3	33.3	33.4
Peperomia succulenta	*Peperomia flagitans*	328	33.3	33.3	33.4
Peperomia succulenta	*Peperomia castanoensis*	373	0	0	100
Peperomia succulenta	*Peperomia gracilipeduncula*	414	100	0	0
Peperomia succulenta	*Peperomia succulenta*	90	50	0	50
Peperomia succulenta	*Peperomia laudabilis*				
Peperomia suchitanensis	*Peperomia suchitanensis*	43	0	0	100
Peperomia suchitanensis	*Peperomia suchitanensis*	110	50	50	0
Peperomia suchitanensis	*Peperomia suchitanensis*	255	100	0	0
Peperomia sucumbiosensis	*Peperomia sucumbiosensis*	255	100	0	0
Peperomia sulbahiensis	*Peperomia sulbahiensis*	51	0	100	0
Peperomia sulbahiensis	*Peperomia sulbahiensis*	82	0	100	0
Peperomia sulbahiensis	*Peperomia sulbahiensis*	222	0	100	0
Peperomia sulcata	*Peperomia sulcata*	51	0	50	50
Peperomia sulcata	*Peperomia sulcata*	82	0	50	50
Peperomia sulcata	*Peperomia sulcata*	42	100	0	0
Peperomia sumidoriana	*Peperomia sumidoriana*	51	100	0	0
Peperomia sumidoriana	*Peperomia sumidoriana*	82	100	0	0
Peperomia sumidoriana	*Peperomia sumidoriana*	42	100	0	0
Peperomia suratana	*Peperomia suratana*	368	50	0	50
Peperomia suratana	*Peperomia suratana*	29	100	0	0
Peperomia suratana	*Peperomia suratana*	373	70	30	0
Peperomia suratana	*Peperomia suratana*	255	100	0	0
Peperomia suspensa	*Peperomia suspensa*	255	100	0	0
Peperomia swartziana	*Peperomia swartziana*	150	0	0	100
Peperomia swartziana	*Peperomia swartziana*	374	66.6	0	33.4
Peperomia swartziana	*Peperomia swartziana*	21	100	0	0
Peperomia swartziana	*Peperomia swartziana*	373	0	0	100
Peperomia swartziana	*Peperomia swartziana*	294	33.3	33.3	33.4
Peperomia swartziana	*Peperomia swartziana*	255	100	0	0
Peperomia swartziana	*Peperomia linearis*				
Peperomia swartziana	*Peperomia olafiana*				
Peperomia sylvatica	*Peperomia sylvatica*	29	0	0	100
Peperomia sylvestris	*Peperomia sylvestris*	374	100	0	0
Peperomia sylvestris	*Peperomia induta*	373	0	0	100
Peperomia symmankii	*Peperomia symmankii*	251	0	50	50
Peperomia sympodialis	*Peperomia sympodialis*	373	50	0	50
Peperomia sympodialis	*Peperomia sympodialis*	255	100	0	0
Peperomia syringifolia	*Peperomia syringifolia*	43	10	45	45
Peperomia syringifolia	*Peperomia syringifolia*	57	100	0	0
Peperomia syringifolia	*Peperomia syringifolia*	150	33.3	33.3	33.4
Peperomia syringifolia	*Peperomia syringifolia*	374	50	50	0
Peperomia syringifolia	*Peperomia syringifolia*	21	100	0	0
Peperomia syringifolia	*Peperomia syringifolia*	373	0	0	100
Peperomia syringifolia	*Peperomia syringifolia*	255	33.3	33.3	33.4
Peperomia syringifolia	*Peperomia syringifolia*	37	50	50	0
Peperomia syringifolia	*Peperomia multiplinervia*				
Peperomia tablahuasiana	*Peperomia tablahuasiana*	373	0	0	100
Peperomia talinifolia	*Peperomia talinifolia*	57	100	0	0
Peperomia talinifolia	*Peperomia talinifolia*	150	0	0	100
Peperomia talinifolia	*Peperomia talinifolia*	219	50	0	50
Peperomia talinifolia	*Peperomia talinifolia*	43	85	0	15
Peperomia talinifolia	*Peperomia talinifolia*	374	50	0	50
Peperomia talinifolia	*Peperomia talinifolia*	29	0	0	100
Peperomia talinifolia	*Peperomia talinifolia*	373	0	0	100
Peperomia talinifolia	*Peperomia talinifolia*	255	50	0	50
Peperomia talinifolia	*Peperomia talinifolia*	112	100	0	0
Peperomia talinifolia	*Peperomia umbellifera*	380	0	0	100
Peperomia talinifolia	*Peperomia umbellifera*	29	0	50	50
Peperomia talinifolia	*Peperomia umbellifera*	373	0	50	50
Peperomia talinifolia	*Peperomia talinifolia*	90	0	0	100
Peperomia tamayoi	*Peperomia tamayoi*	150	0	100	0
Peperomia tamayoi	*Peperomia tamayoi*	373	0	0	100
Peperomia tamayoi	*Peperomia tamayoi*	255	0	100	0
Peperomia tambitoensis	*Peperomia tambitoensis*	373	0	0	100
Peperomia tamboana	*Peperomia tamboana*	373	0	100	0
Peperomia tancitaroana	*Peperomia tancitaroana*	209	0	50	50
Peperomia tarapotana	*Peperomia tarapotana*	29	0	0	100
Peperomia tatei	*Peperomia tatei*				
Peperomia tejana	*Peperomia tejana*	150	100	0	0
Peperomia tejana	*Peperomia tejana*	373	85	0	15
Peperomia tejana	*Peperomia tejana*	255	100	0	0
Peperomia tenae	*Peperomia tenae*	373	0	0	100
Peperomia tenella	*Peperomia tenella*	18	100	0	0

Peperomia tenella	*Peperomia tenella*	51	0	100	0
Peperomia tenella	*Peperomia tenella*	43	100	0	0
Peperomia tenella	*Peperomia tenella*	57	100	0	0
Peperomia tenella	*Peperomia tenella*	110	100	0	0
Peperomia tenella	*Peperomia tenella*	150	33.3	33.3	33.4
Peperomia tenella	*Peperomia tenella*	219	100	0	0
Peperomia tenella	*Peperomia tenella*	21	100	0	0
Peperomia tenella	*Peperomia tenella*	83	33.3	33.3	33.4
Peperomia tenella	*Peperomia tenella*	232	100	0	0
Peperomia tenella	*Peperomia tenella*	172	100	0	0
Peperomia tenella	*Peperomia tenella*	29	0	0	100
Peperomia tenella	*Peperomia tenella*	373	100	0	0
Peperomia tenella	*Peperomia tenella*	220	0	100	0
Peperomia tenella	*Peperomia tenella*	171	100	0	0
Peperomia tenella	*Peperomia tenella*	335	100	0	0
Peperomia tenella	*Peperomia tenella*	294	50	50	0
Peperomia tenella	*Peperomia tenella*	48	0	100	0
Peperomia tenella	*Peperomia tenella*	153	50	0	50
Peperomia tenella	*Peperomia tenella*	214	0	0	100
Peperomia tenella	*Peperomia tenella*	260	100	0	0
Peperomia tenella	*Peperomia tenella*	255	33.3	33.3	33.4
Peperomia tenella	*Peperomia tenella*	37	100	0	0
Peperomia tenella	*Peperomia tenella*	112	100	0	0
Peperomia tenella	*Peperomia tenella*	128	100	0	0
Peperomia tenella	*Peperomia palcipila*	67	0	100	0
Peperomia tenella	*Peperomia tenella*	90	33.3	33.3	33.4
Peperomia tenelliformis	*Peperomia tenelliformis*	43	100	0	0
Peperomia tenelliformis	*Peperomia tenelliformis*	57	0	0	100
Peperomia tenelliformis	*Peperomia tenelliformis*	219	100	0	0
Peperomia tenelliformis	*Peperomia tenelliformis*	255	33.3	33.3	33.4
Peperomia tenelliformis	*Peperomia tenelliformis*	37	85	0	15
Peperomia tenerrima	*Peperomia tenerrima*	43	100	0	0
Peperomia tenerrima	*Peperomia tenerrima*	52	100	0	0
Peperomia tenerrima	*Peperomia tenerrima*	213	100	0	0
Peperomia tenerrima	*Peperomia tenerrima*	335	100	0	0
Peperomia tenerrima	*Peperomia tenerrima*	255	100	0	0
Peperomia tenerrima	*Peperomia matagalpensis*	38	100	0	0
Peperomia tenerrima	*Peperomia schiedeana*	300	100	0	0
Peperomia tenuicaulis	*Peperomia tenuicaulis*	43	100	0	0
Peperomia tenuicaulis	*Peperomia tenuicaulis*	255	100	0	0
Peperomia tenuifolia	*Peperomia tenuifolia*	43	50	50	0
Peperomia tenuifolia	*Peperomia killipi*	60	0	0	100
Peperomia tenuifolia	*Peperomia aguacatensis*				
Peperomia tenuilimba	*Peperomia tenuilimba*	51	0	0	100
Peperomia tenuilimba	*Peperomia tenuilimba*	82	0	0	100
Peperomia tenuilimba	*Peperomia tenuilimba*	42	100	0	0
Peperomia tenuimarginata	*Peperomia tenuimarginata*	198	0	0	100
Peperomia tenuipeduncula	*Peperomia tenuipeduncula*	255	100	0	0
Peperomia tenuipeduncula	*Peperomia tenuipeduncula*	360	0	100	0
Peperomia tenuipes	*Peperomia tenuipes*	43	50	0	50
Peperomia tenuipes	*Peperomia tenuipes*	150	33.3	33.3	33.4
Peperomia tenuipes	*Peperomia tenuipes*	219	50	0	50
Peperomia tenuipes	*Peperomia tenuipes*	214	100	0	0
Peperomia tenuipes	*Peperomia tenuipes*	255	50	0	50
Peperomia tenuipes	*Peperomia tyleri*	51	50	0	50
Peperomia tenuipes	*Peperomia tyleri*	373	50	0	50
Peperomia tenuipes	*Peperomia sphagnicola*				
Peperomia tenuipes	*Peperomia coliblancoana*				
Peperomia tenuipes	*Peperomia inaudax*				
Peperomia tenuipila	*Peperomia tenuipila*				
Peperomia tenuiramea	*Peperomia tenuiramea*	29	100	0	0
Peperomia tenuiramea	*Peperomia tenuiramea*	255	100	0	0
Peperomia tenuiramea	*Peperomia tenuiramea*	178	100	0	0
Peperomia tenuissima	*Peperomia pseudoestrellensis*	18	100	0	0
Peperomia tepoztecoana	*Peperomia tepoztecoana*	204	0	100	0
Peperomia tequendamana	*Peperomia tequendamana*	43	50	0	50
Peperomia tequendamana	*Peperomia tequendamana*	150	33.3	33.3	33.4
Peperomia tequendamana	*Peperomia tequendamana*	368	0	0	100
Peperomia tequendamana	*Peperomia tequendamana*	373	0	50	50
Peperomia tequendamana	*Peperomia tequendamana*	255	50	0	50
Peperomia tequendamana	*Peperomia tequendamana*	90	50	0	50
Peperomia terebinthina	*Peperomia terebinthina*	199	100	0	0
Peperomia terebinthina	*Peperomia terebinthina*	255	100	0	0
Peperomia teresitensis	*Peperomia teresitensis*	29	0	0	100
Peperomia ternata	*Peperomia donata*	57	0	0	100
Peperomia ternata	*Peperomia ternata*	150	0	0	100
Peperomia ternata	*Peperomia ternata*	219	50	0	50
Peperomia ternata	*Peperomia ternata*	374	28.6	0	71.4
Peperomia ternata	*Peperomia ternata*	335	100	0	0
Peperomia ternata	*Peperomia ternata*	255	50	0	50

Peperomia ternata	*Peperomia abrupteacutata*				
Peperomia terraegaudens	*Peperomia terraegaudens*	373	0	0	100
Peperomia tetragona	*Peperomia tetragona*	374	100	0	0
Peperomia tetragona	*Peperomia puteolata*	29	0	0	100
Peperomia tetragona	*Peperomia tetragona*	189	50	50	0
Peperomia tetragona	*Peperomia tetragona*	29	100	0	0
Peperomia tetragona	*Peperomia tetragona*	255	100	0	0
Peperomia tetragona	*Peperomia albostriata*	198	0	0	100
Peperomia tetragona	*Peperomia albostriata*	375	50	0	50
Peperomia tetraphylla	*Peperomia tetraphylla*	18	100	0	0
Peperomia tetraphylla	*Peperomia tetraphylla*	19	100	0	0
Peperomia tetraphylla	*Peperomia tetraphylla*	20	100	0	0
Peperomia tetraphylla	*Peperomia tetraphylla*	24	100	0	0
Peperomia tetraphylla	*Peperomia tetraphylla*	51	33.3	33.3	33.4
Peperomia tetraphylla	*Peperomia tetraphylla*	39	100	0	0
Peperomia tetraphylla	*Peperomia tetraphylla*	43	50	0	50
Peperomia tetraphylla	*Peperomia tetraphylla*	57	100	0	0
Peperomia tetraphylla	*Peperomia tetraphylla*	110	100	0	0
Peperomia tetraphylla	*Peperomia tetraphylla*	114	95	0	5
Peperomia tetraphylla	*Peperomia tetraphylla*	150	50	50	0
Peperomia tetraphylla	*Peperomia tetraphylla*	167	100	0	0
Peperomia tetraphylla	*Peperomia tetraphylla*	183	50	50	0
Peperomia tetraphylla	*Peperomia tetraphylla*	184	100	0	0
Peperomia tetraphylla	*Peperomia tetraphylla*	213	100	0	0
Peperomia tetraphylla	*Peperomia tetraphylla*	219	100	0	0
Peperomia tetraphylla	*Peperomia tetraphylla*	234	50	50	0
Peperomia tetraphylla	*Peperomia tetraphylla*	275	100	0	0
Peperomia tetraphylla	*Peperomia tetraphylla*	374	100	0	0
Peperomia tetraphylla	*Peperomia tetraphylla*	27	50	0	50
Peperomia tetraphylla	*Peperomia tetraphylla*	21	100	0	0
Peperomia tetraphylla	*Peperomia tetraphylla*	83	95	5	0
Peperomia tetraphylla	*Peperomia tetraphylla*	255	33	34	33
Peperomia tetraphylla	*Peperomia tetraphylla*	58	50	50	0
Peperomia tetraphylla	*Peperomia tetraphylla*	14	50	50	0
Peperomia tetraphylla	*Peperomia tetraphylla*	273	50	50	0
Peperomia tetraphylla	*Peperomia tetraphylla*	123	100	0	0
Peperomia tetraphylla	*Peperomia diehliana*	29	0	0	100
Peperomia tetraphylla	*Peperomia tetraphylla*	144	100	0	0
Peperomia tetraphylla	*Peperomia tetraphylla*	133	50	50	0
Peperomia tetraphylla	*Peperomia tetraphylla*	29	0	0	100
Peperomia tetraphylla	*Peperomia tetraphylla*	1	100	0	0
Peperomia tetraphylla	*Peperomia tetraphylla*	384	85	5	10
Peperomia tetraphylla	*Peperomia tetraphylla*	238	0	100	0
Peperomia tetraphylla	*Peperomia tetraphylla*	220	50	50	0
Peperomia tetraphylla	*Peperomia tetraphylla*	171	100	0	0
Peperomia tetraphylla	*Peperomia tetraphylla*	91	47.5	47.5	5
Peperomia tetraphylla	*Peperomia tetraphylla*	12	100	0	0
Peperomia tetraphylla	*Peperomia tetraphylla*	335	100	0	0
Peperomia tetraphylla	*Peperomia tetraphylla*	155	50	50	0
Peperomia tetraphylla	*Peperomia tetraphylla*	15	100	0	0
Peperomia tetraphylla	*Peperomia tetraphylla*	294	33.3	33.3	33.4
Peperomia tetraphylla	*Peperomia tetraphylla*	387	50	50	0
Peperomia tetraphylla	*Peperomia tetraphylla*	179	100	0	0
Peperomia tetraphylla	*Peperomia tetraphylla*	221	50	0	50
Peperomia tetraphylla	*Peperomia tetraphylla*	48	100	0	0
Peperomia tetraphylla	*Peperomia tetraphylla*	119	85	15	0
Peperomia tetraphylla	*Peperomia tetraphylla*	84	100	0	0
Peperomia tetraphylla	*Peperomia tetraphylla*	136	100	0	0
Peperomia tetraphylla	*Peperomia tetraphylla*	214	100	0	0
Peperomia tetraphylla	*Peperomia tetraphylla*	85	100	0	0
Peperomia tetraphylla	*Peperomia tetraphylla*	293	50	50	0
Peperomia tetraphylla	*Peperomia tetraphylla*	418	100	0	0
Peperomia tetraphylla	*Peperomia tetraphylla*	175	100	0	0
Peperomia tetraphylla	*Peperomia tetraphylla*	156	100	0	0
Peperomia tetraphylla	*Peperomia tetraphylla*	115	95	5	0
Peperomia tetraphylla	*Peperomia tetraphylla*	252	33.3	33.3	33.4
Peperomia tetraphylla	*Peperomia tetraphylla*	37	100	0	0
Peperomia tetraphylla	*Peperomia tetraphylla*	90	85	0	15
Peperomia tetraphylla	*Peperomia chrysolepida*	110	100	0	0
Peperomia tetraphylla	*Peperomia quaternata*	57	0	0	100
Peperomia tetraphylla	*Peperomia tacuariana*	146	0	100	0
Peperomia tetraphylla	*Peperomia tetraphylla*	88	100	0	0
Peperomia tetraphylla	*Peperomia tetraphylla*	375	90	10	0
Peperomia tetraphylla	*Peperomia aemula*				
Peperomia tetraphylla	*Peperomia affinis*				
Peperomia tetraphylla	*Peperomia novae-zelandiae*				
Peperomia tetraphylla	*Peperomia opaca*				
Peperomia tetraphylla	*Peperomia trichoclada*				
Peperomia tetraquetra	*Peperomia tetraquetra*	39	100	0	0
Peperomia tetraquetra	*Peperomia tetraquetra*	150	50	0	50

Peperomia tetraquetra	*Peperomia tetraquetra*	374	80	0	20
Peperomia tetraquetra	*Peperomia tetraquetra*	373	0	0	100
Peperomia tetraquetra	*Peperomia tetraquetra*	255	50	0	50
Peperomia tetraquetra	*Peperomia larana*	373	100	0	0
Peperomia teysmannii	*Peperomia teysmannii*				
Peperomia theodori	*Peperomia theodori*	412	100	0	0
Peperomia theodori	*Peperomia theodori*	418	100	0	0
Peperomia theodori	*Peperomia theodori*	255	100	0	0
Peperomia thienii	*Peperomia thienii*				
Peperomia thollonii	*Peperomia thollonii*	76	0	100	0
Peperomia thomeana	*Peperomia thomeana*	91	33.3	33.4	33.3
Peperomia thomeana	*Peperomia thomeana*	255	50	0	50
Peperomia thomeana	*Peperomia vaccinifolia*				
Peperomia thomsonii	*Peperomia thomsonii*	125	100	0	0
Peperomia thorelii	*Peperomia thorelii*	255	100	0	0
Peperomia ticunhuayana	*Peperomia ticunhuayana*	353	0	0	100
Peperomia tillettii	*Peperomia tillettii*	150	0	0	100
Peperomia timbuchiana	*Peperomia timbuchiana*	29	0	0	100
Peperomia tjibodasana	*Peperomia tjibodasana*				
Peperomia tlapacoyoensis	*Peperomia tlapacoyoensis*	110	50	50	0
Peperomia tlapacoyoensis	*Peperomia tlapacoyoensis*	255	100	0	0
Peperomia toledoana	*Peperomia toledoana*	43	0	100	0
Peperomia toledoana	*Peperomia toledoana*	255	0	50	50
Peperomia toledoana	*Peperomia toledoana*	204	0	100	0
Peperomia tolimensis	*Peperomia tolimensis*	373	0	0	100
Peperomia tolimensis	*Peperomia tolimensis*	255	100	0	0
Peperomia tomentella	*Peperomia tomentella*	373	0	0	100
Peperomia tomentosa	*Peperomia tomentosa*	12	100	0	0
Peperomia tominana	*Peperomia tominana*	412	100	0	0
Peperomia tominana	*Peperomia tominana*	418	50	50	0
Peperomia tominana	*Peperomia tominana*	255	50	50	0
Peperomia tonduzii	*Peperomia tonduzii*	43	100	0	0
Peperomia tonduzii	*Peperomia tonduzii*	219	100	0	0
Peperomia tonduzii	*Peperomia tonduzii*	255	100	0	0
Peperomia tooviiana	*Peperomia tooviiana*	388	85	7.5	7.5
Peperomia tooviiana	*Peperomia tooviiana*	115	100	0	0
Peperomia tooviiana	*Peperomia tooviiana*	255	100	0	0
Peperomia topoensis	*Peperomia topoensis*	373	100	0	0
Peperomia topoensis	*Peperomia topoensis*	255	100	0	0
Peperomia toroi	*Peperomia toroi*	255	100	0	0
Peperomia tovariana	*Peperomia tovariana*	43	100	0	0
Peperomia tovariana	*Peperomia tovariana*	57	100	0	0
Peperomia tovariana	*Peperomia tovariana*	150	50	50	0
Peperomia tovariana	*Peperomia tovariana*	213	0	0	100
Peperomia tovariana	*Peperomia tovariana*	219	100	0	0
Peperomia tovariana	*Peperomia tovariana*	29	100	0	0
Peperomia tovariana	*Peperomia tovariana*	373	100	0	0
Peperomia tovariana	*Peperomia tovariana*	255	50	50	0
Peperomia tovariana	*Peperomia tovariana*	90	50	50	0
Peperomia tradescantiifolia	*Peperomia tradescantiifolia*				
Peperomia transparens	*Peperomia transparens*	255	0	100	0
Peperomia trianae	*Peperomia trianae*	373	0	0	100
Peperomia trianae	*Peperomia discistilla*	373	0	0	100
Peperomia trianae	*Peperomia trianae*	255	100	0	0
Peperomia trianae	*Peperomia enantiostachya*	39	100	0	0
Peperomia trianae	*Peperomia enantiostachya*	150	100	0	0
Peperomia trianae	*Peperomia enantiostachya*	373	100	0	0
Peperomia trichobracteata	*Peperomia trichobracteata*	43	100	0	0
Peperomia trichobracteata	*Peperomia trichobracteata*	157	100	0	0
Peperomia trichobracteata	*Peperomia trichobracteata*	255	100	0	0
Peperomia trichocarpa	*Peperomia trichocarpa*	51	100	0	0
Peperomia trichocarpa	*Peperomia trichocarpa*	82	100	0	0
Peperomia trichocarpa	*Peperomia trichocarpa*	255	100	0	0
Peperomia trichomanoides	*Peperomia trichomanoides*	43	0	100	0
Peperomia trichomanoides	*Peperomia trichomanoides*	132	0	100	0
Peperomia trichomanoides	*Peperomia trichomanoides*	255	0	100	0
Peperomia trichophylla	*Peperomia trichophylla*	255	100	0	0
Peperomia trichopodus	*Peperomia trichopodus*	7	50	50	0
Peperomia trichopus	*Peperomia trichopus*	39	100	0	0
Peperomia trichopus	*Peperomia trichopus*	150	100	0	0
Peperomia trichopus	*Peperomia trichopus*	374	100	0	0
Peperomia trichopus	*Peperomia trichopus*	29	100	0	0
Peperomia trichopus	*Peperomia trichopus*	373	100	0	0
Peperomia trichopus	*Peperomia trichopus*	255	100	0	0
Peperomia trichopus	*Peperomia trichopus*	178	100	0	0
Peperomia trichopus	*Peperomia trichopus*	90	100	0	0
Peperomia tricolor	*Peperomia tricolor*	29	33.3	33.3	33.4
Peperomia tricolor	*Peperomia tricolor*	255	100	0	0
Peperomia tricolor	*Peperomia tricolor*	178	100	0	0
Peperomia trifolia	*Peperomia trifolia*	29	100	0	0

Peperomia trifolia	*Peperomia trifolia*	373	0	0	100
Peperomia trifolia	*Peperomia trifolia*	134	100	0	0
Peperomia trifolia	*Peperomia trifolia*	153	100	0	0
Peperomia trifolia	*Peperomia trifolia*	255	100	0	0
Peperomia trifolia	*Peperomia caespitiformans*				
Peperomia trifolia	*Peperomia obversa*				
Peperomia trifolia	*Peperomia fimbriata*				
Peperomia trifolia	*Peperomia caespitiformans*				
Peperomia trifolia	*Peperomia balbisii*				
Peperomia trinervis	*Peperomia trinervis*	51	50	50	0
Peperomia trinervis	*Peperomia trinervis*	110	100	0	0
Peperomia trinervis	*Peperomia trinervis*	150	100	0	0
Peperomia trinervis	*Peperomia trinervis*	374	66.6	0	33.4
Peperomia trinervis	*Peperomia trinervis*	21	100	0	0
Peperomia trinervis	*Peperomia leuconeura*	29	0	0	100
Peperomia trinervis	*Peperomia trinervis*	29	100	0	0
Peperomia trinervis	*Peperomia trinervis*	373	100	0	0
Peperomia trinervis	*Peperomia trinervis*	220	50	50	0
Peperomia trinervis	*Peperomia trinervis*	221	100	0	0
Peperomia trinervis	*Peperomia trinervis*	255	100	0	0
Peperomia trinervula	*Peperomia trinervula*	43	5	0	95
Peperomia trinervula	*Peperomia trinervula*	150	0	50	50
Peperomia trinervula	*Peperomia trinervula*	380	0	0	100
Peperomia trinervula	*Peperomia trinervula*	373	30	0	70
Peperomia trinervula	*Peperomia trinervula*	214	100	0	0
Peperomia trinervula	*Peperomia trinervula*	255	33.3	33.3	33.4
Peperomia trinervula	*Peperomia roraimana*	378	100	0	0
Peperomia trinervula	*Peperomia trinervula*	90	33.3	33.3	33.4
Peperomia trineura	*Peperomia trineura*	51	50	50	0
Peperomia trineura	*Peperomia trineura*	220	70	30	0
Peperomia trineura	*Peperomia trineura*	113	45	33	22
Peperomia trineura	*Peperomia trineura*	48	0	100	0
Peperomia trineura	*Peperomia trineura*	255	100	0	0
Peperomia trineura	*Peperomia trinervia*				
Peperomia trineuroides	*Peperomia trineuroides*	51	50	50	0
Peperomia trineuroides	*Peperomia trineuroides*	183	42.5	42.5	15
Peperomia trineuroides	*Peperomia trineuroides*	179	100	0	0
Peperomia trineuroides	*Peperomia trineuroides*	220	0	100	0
Peperomia trineuroides	*Peperomia trineuroides*	418	100	0	0
Peperomia trineuroides	*Peperomia trineuroides*	255	33.3	33.3	33.4
Peperomia trineuroides	*Peperomia arechavaletae*	198	100	0	0
Peperomia triplinervis	*Peperomia triplinervis*	373	0	0	100
Peperomia tristachya	*Peperomia tristachya*	43	0	0	100
Peperomia trollii	*Peperomia trollii*	29	0	0	100
Peperomia trollii	*Peperomia trollii*	243	0	80	20
Peperomia tropeoloides	*Peperomia tropeoloides*	374	50	0	50
Peperomia tropeoloides	*Peperomia gazauntana*	373	100	0	0
Peperomia trujilloi	*Peperomia trujilloi*	150	100	0	0
Peperomia trujilloi	*Peperomia trujilloi*	255	100	0	0
Peperomia trukensis	*Peperomia trukensis*	362	0	50	50
Peperomia trullifolia	*Peperomia trullifolia*	29	0	0	100
Peperomia truncicola	*Peperomia truncicola*	255	100	0	0
Peperomia truncigaudens	*Peperomia truncigaudens*	153	100	0	0
Peperomia truncigaudens	*Peperomia truncigaudens*	255	100	0	0
Peperomia trunciseda	*Peperomia trunciseda*	374	100	0	0
Peperomia trunciseda	*Peperomia trunciseda*	373	100	0	0
Peperomia trunciseda	*Peperomia trunciseda*	255	100	0	0
Peperomia truncivaga	*Peperomia truncivaga*	373	100	0	0
Peperomia truncivaga	*Peperomia truncivaga*	255	100	0	0
Peperomia tsakiana	*Peperomia tsakiana*	43	85	7.5	7.5
Peperomia tsakiana	*Peperomia tsakiana*	57	100	0	0
Peperomia tsakiana	*Peperomia tsakiana*	219	100	0	0
Peperomia tsakiana	*Peperomia tsakiana*	255	100	0	0
Peperomia tsakiana	*Peperomia tsakiana*	37	100	0	0
Peperomia tuberculata	*Peperomia tuberculata*				
Peperomia tubericordata	*Peperomia tubericordata*	206	0	100	0
Peperomia tuerckheimii	*Peperomia tuerckheimii*	43	33	34	33
Peperomia tuerckheimii	*Peperomia tuerckheimii*	110	50	50	0
Peperomia tuerckheimii	*Peperomia tuerckheimii*	328	0	100	0
Peperomia tuerckheimii	*Peperomia tuerckheimii*	255	33.3	33.3	33.4
Peperomia tuerckheimii	*Peperomia fugax*	43	0	0	100
Peperomia tuisana	*Peperomia tuisana*	43	50	0	50
Peperomia tuisana	*Peperomia tuisana*	110	10	90	0
Peperomia tuisana	*Peperomia tuisana*	335	100	0	0
Peperomia tuisana	*Peperomia tuisana*	255	33.3	33.3	33.4
Peperomia tuisana	*Peperomia tuisana*	37	100	0	0
Peperomia tuisana	*Peperomia pililimba*	328	50	50	0
Peperomia tuisana	*Peperomia pililimba*	302	0	100	0
Peperomia tuisana	*Peperomia pililimba*	325	100	0	0
Peperomia tuisana	*Peperomia matapalo*				

Peperomia tumida	*Peperomia tumida*				
Peperomia tungurahuae	*Peperomia tungurahuae*	373	50	0	50
Peperomia tungurahuae	*Peperomia tungurahuae*	255	100	0	0
Peperomia turbinata	*Peperomia turbinata*	51	50	0	50
Peperomia turbinata	*Peperomia turbinata*	82	33	34	33
Peperomia turboensis	*Peperomia turboensis*	43	0	0	100
Peperomia turboensis	*Peperomia turboensis*	150	0	0	100
Peperomia turboensis	*Peperomia turboensis*	373	0	0	100
Peperomia turboensis	*Peperomia buntingii*				
Peperomia tutensis	*Peperomia tutensis*	43	100	0	0
Peperomia tutensis	*Peperomia tutensis*	255	100	0	0
Peperomia tutuilana	*Peperomia tutuilana*	126	0	100	0
Peperomia tutunendoana	*Peperomia tutunendoana*	29	100	0	0
Peperomia tutunendoana	*Peperomia tutunendoana*	373	0	0	100
Peperomia tutunendoana	*Peperomia tutunendoana*	255	100	0	0
Peperomia uaupesensis	*Peperomia uaupesensis*	51	100	0	0
Peperomia uaupesensis	*Peperomia uaupesensis*	150	100	0	0
Peperomia uaupesensis	*Peperomia uaupesensis*	223	50	50	0
Peperomia uaupesensis	*Peperomia uaupesensis*	42	100	0	0
Peperomia uaupesensis	*Peperomia uaupesensis*	255	100	0	0
Peperomia ubate-susanensis	*Peperomia ubate-susanensis*	373	0	0	100
Peperomia udimontana	*Peperomia udimontana*	255	100	0	0
Peperomia udisilvestris	*Peperomia udisilvestris*	255	100	0	0
Peperomia umbilicata	*Peperomia hillii*	29	0	0	100
Peperomia umbilicata	*Peperomia umbilicata*	29	0	50	50
Peperomia umbilicata	*Peperomia hillii*	178	0	100	0
Peperomia umbrigaudens	*Peperomia umbrigaudens*	57	0	0	100
Peperomia umbrigaudens	*Peperomia umbrigaudens*	255	100	0	0
Peperomia umbrigaudens	*Peperomia umbrigaudens*	43	100	0	0
Peperomia umbrosa	*Peperomia umbrosa*	292	0	0	100
Peperomia uncatispica	*Peperomia uncatispica*	29	0	0	100
Peperomia undeninervia	*Peperomia undeninervia*	29	0	0	100
Peperomia undeninervia	*Peperomia undeninervia*	255	100	0	0
Peperomia unduavina	*Peperomia unduavina*	51	100	0	0
Peperomia unifoliata	*Peperomia unifoliata*	43	0	100	0
Peperomia unifoliata	*Peperomia unifoliata*	255	50	50	0
Peperomia unifoliata	*Peperomia unifoliata*	204	50	50	0
Peperomia unispicata	*Peperomia unispicata*	43	100	0	0
Peperomia unispicata	*Peperomia unispicata*	255	100	0	0
Peperomia urbanii	*Peperomia urbanii*	294	0	50	50
Peperomia urocarpa	*Peperomia urocarpa*	9	0	100	0
Peperomia urocarpa	*Peperomia urocarpa*	51	33.3	33.3	33.4
Peperomia urocarpa	*Peperomia urocarpa*	43	50	0	50
Peperomia urocarpa	*Peperomia urocarpa*	80	100	0	0
Peperomia urocarpa	*Peperomia urocarpa*	110	50	0	50
Peperomia urocarpa	*Peperomia urocarpa*	150	45	45	10
Peperomia urocarpa	*Peperomia urocarpa*	219	0	0	100
Peperomia urocarpa	*Peperomia urocarpa*	275	100	0	0
Peperomia urocarpa	*Peperomia urocarpa*	374	33.4	0	66.6
Peperomia urocarpa	*Peperomia urocarpa*	21	100	0	0
Peperomia urocarpa	*Peperomia urocarpa*	83	50	50	0
Peperomia urocarpa	*Peperomia urocarpa*	273	15	42.5	42.5
Peperomia urocarpa	*Peperomia urocarpa*	172	0	50	50
Peperomia urocarpa	*Peperomia urocarpa*	29	100	0	0
Peperomia urocarpa	*Peperomia condotoana*	373	0	0	100
Peperomia urocarpa	*Peperomia urocarpa*	373	50	0	50
Peperomia urocarpa	*Peperomia urocarpa*	220	50	50	0
Peperomia urocarpa	*Peperomia urocarpa*	171	0	100	0
Peperomia urocarpa	*Peperomia urocarpa*	335	33.3	33.3	33.4
Peperomia urocarpa	*Peperomia urocarpa*	294	50	0	50
Peperomia urocarpa	*Peperomia urocarpa*	179	33.3	33.3	33.4
Peperomia urocarpa	*Peperomia urocarpa*	221	0	50	50
Peperomia urocarpa	*Peperomia urocarpa*	47	33.3	33.3	33.4
Peperomia urocarpa	*Peperomia urocarpa*	153	50	0	50
Peperomia urocarpa	*Peperomia urocarpa*	84	33.3	33.3	33.4
Peperomia urocarpa	*Peperomia urocarpa*	42	100	0	0
Peperomia urocarpa	*Peperomia urocarpa*	85	33.3	33.3	33.4
Peperomia urocarpa	*Peperomia urocarpa*	418	50	0	50
Peperomia urocarpa	*Peperomia urocarpa*	224	100	0	0
Peperomia urocarpa	*Peperomia urocarpa*	156	0	100	0
Peperomia urocarpa	*Peperomia urocarpa*	260	50	50	0
Peperomia urocarpa	*Peperomia urocarpa*	255	33.3	33.3	33.4
Peperomia urocarpa	*Peperomia urocarpa*	252	33.3	33.3	33.4
Peperomia urocarpa	*Peperomia urocarpa*	128	50	50	0
Peperomia urocarpa	*Peperomia major*	328	50	50	0
Peperomia urocarpa	*Peperomia novae-hispaniae*	401	100	0	0
Peperomia urocarpa	*Peperomia pseudomajor*	66	0	100	0
Peperomia urocarpa	*Peperomia tacanana*	198	50	0	50
Peperomia urocarpa	*Peperomia tacanana*	328	42.5	15	42.5
Peperomia urocarpa	*Peperomia urocarpa*	375	80	0	20

Peperomia urocarpa	*Peperomia negotiosa*				
Peperomia urocarpa	*Peperomia osana*				
Peperomia urocarpa	*Peperomia pilosula*				
Peperomia urocarpa	*Peperomia tremuliformis*				
Peperomia urocarpa	*Peperomia davisii*				
Peperomia urocarpa	*Peperomia fumeana*				
Peperomia urocarpa	*Peperomia hederacea*				
Peperomia urocarpa	*Peperomia praecox*				
Peperomia urocarpoides	*Peperomia urocarpoides*	57	0	0	100
Peperomia urocarpoides	*Peperomia urocarpoides*	280	100	0	0
Peperomia urocarpoides	*Peperomia cataratasensis*	326	100	0	0
Peperomia ursina	*Peperomia ursina*	43	100	0	0
Peperomia ursina	*Peperomia ursina*	255	50	50	0
Peperomia ursina	*Peperomia ursina*	132	50	50	0
Peperomia urvilleana	*Peperomia urvilleana*	167	100	0	0
Peperomia urvilleana	*Peperomia urvilleana*	234	50	50	0
Peperomia urvilleana	*Peperomia urvilleana*	143	0	100	0
Peperomia urvilleana	*Peperomia urvilleana*	343	85	15	0
Peperomia urvilleana	*Peperomia urvilleana*	58	33.3	33.3	33.4
Peperomia urvilleana	*Peperomia urvilleana*	133	50	50	0
Peperomia urvilleana	*Peperomia endlicheri*	307	0	100	0
Peperomia urvilleana	*Peperomia urvilleana*	255	50	0	50
Peperomia urvilleana	*Peperomia baueriana*				
Peperomia urvilleana	*Peperomia muricatulata*				
Peperomia valdezii	*Peperomia valdezii*	43	100	0	0
Peperomia valdezii	*Peperomia valdezii*	382	100	0	0
Peperomia valladolidana	*Peperomia valladolidana*	373	0	0	100
Peperomia vallensis	*Peperomia vallensis*	374	100	0	0
Peperomia vallensis	*Peperomia vallensis*	373	0	0	100
Peperomia vallensis	*Peperomia vallensis*	255	100	0	0
Peperomia valliculae	*Peperomia valliculae*	43	100	0	0
Peperomia valliculae	*Peperomia valliculae*	57	0	0	100
Peperomia valliculae	*Peperomia valliculae*	255	100	0	0
Peperomia vana	*Peperomia vana*	29	0	0	100
Peperomia vana	*Peperomia vana*	255	100	0	0
Peperomia vareschii	*Peperomia vareschii*	150	0	0	100
Peperomia vareschii	*Peperomia vareschii*	380	0	0	100
Peperomia variculata	*Peperomia variculata*	29	0	0	100
Peperomia variculata	*Peperomia variculata*	166	100	0	0
Peperomia variculata	*Peperomia variculata*	178	100	0	0
Peperomia variifolia	*Peperomia variifolia*	29	0	0	100
Peperomia variilimba	*Peperomia variilimba*	208	100	0	0
Peperomia vazquezii	*Peperomia vazquezii*	43	0	50	50
Peperomia vazquezii	*Peperomia vazquezii*	205	0	100	0
Peperomia vazquezii	*Peperomia vazquezii*	255	0	50	50
Peperomia vellarimalica	*Peperomia vellarimalica*	192	0	0	100
Peperomia velloziana	*Peperomia velloziana*	51	33.3	33.3	33.4
Peperomia velloziana	*Peperomia velloziana*	82	33	33	34
Peperomia velloziana	*Peperomia velloziana*	179	50	50	0
Peperomia velloziana	*Peperomia flavidinervis*				
Peperomia velutina	*Peperomia velutina*	43	100	0	0
Peperomia velutina	*Peperomia velutina*	374	100	0	0
Peperomia velutina	*Peperomia velutina*	373	0	0	100
Peperomia velutina	*Peperomia velutina*	255	100	0	0
Peperomia venabulifolia	*Peperomia venabulifolia*	43	50	0	50
Peperomia venabulifolia	*Peperomia venabulifolia*	219	50	0	50
Peperomia venabulifolia	*Peperomia venabulifolia*	255	50	0	50
Peperomia venabulifolia	*Peperomia venabulifolia*	326	100	0	0
Peperomia veneciana	*Peperomia veneciana*	373	100	0	0
Peperomia veneciana	*Peperomia veneciana*	255	100	0	0
Peperomia venezueliana	*Peperomia venezueliana*	150	33.3	33.3	33.4
Peperomia venezueliana	*Peperomia venezueliana*	373	0	15	85
Peperomia venezueliana	*Peperomia venezueliana*	255	50	0	50
Peperomia venezueliana	*Peperomia ernstiana*				
Peperomia venosa	*Peperomia venosa*	373	0	0	100
Peperomia ventenatii	*Peperomia ventenatii*	255	100	0	0
Peperomia venticosicarpa	*Peperomia venticosicarpa*	29	0	0	100
Peperomia venulosa	*Peperomia venulosa*	374	25	0	75
Peperomia venulosa	*Peperomia venulosa*	191	100	0	0
Peperomia venusta	*Peperomia venusta*	150	0	0	100
Peperomia venusta	*Peperomia venusta*	414	0	0	100
Peperomia veraguana	*Peperomia veraguana*	43	100	0	0
Peperomia veraguana	*Peperomia veraguana*	255	100	0	0
Peperomia verediana	*Peperomia verediana*	29	0	0	100
Peperomia vernouana	*Peperomia vernouana*	334	100	0	0
Peperomia verruculosa	*Peperomia verruculosa*	29	0	50	50
Peperomia verruculosa	*Peperomia verruculosa*	121	0	100	0
Peperomia verschaffeltii	*Peperomia verschaffeltii*	277	0	0	100
Peperomia versicolor	*Peperomia versicolor*	31	100	0	0
Peperomia versicolor	*Peperomia versicolor*	43	50	50	0

Peperomia versicolor	*Peperomia versicolor*	57	100	0	0
Peperomia versicolor	*Peperomia versicolor*	374	100	0	0
Peperomia versicolor	*Peperomia versicolor*	21	100	0	0
Peperomia versicolor	*Peperomia versicolor*	335	50	50	0
Peperomia versicolor	*Peperomia versicolor*	255	50	50	0
Peperomia versicolor	*Peperomia niveopunctulata*				
Peperomia versteegii	*Peperomia versteegii*				
Peperomia verticillata	*Peperomia verticillata*	294	5	47.5	47.5
Peperomia verticillata	*Peperomia verticillata*	255	100	0	0
Peperomia verticillata	*Peperomia verticillata*	112	0	50	50
Peperomia verticillata	*Peperomia rubella*				
Peperomia verticillata	*Peperomia roigana*				
Peperomia verticillata	*Peperomia orbiculata*				
Peperomia verticillata	*Peperomia papulata*				
Peperomia verticillata	*Peperomia pulchella*				
Peperomia verticillata	*Peperomia subpulchella*				
Peperomia verticillatispica	*Peperomia verticillatispica*	374	75	0	25
Peperomia verticillatispica	*Peperomia verticillatispica*	373	100	0	0
Peperomia verticillatispica	*Peperomia verticillatispica*	255	100	0	0
Peperomia vestita	*Peperomia vestita*	412	0	0	100
Peperomia vidaliana	*Peperomia vidaliana*	29	0	0	100
Peperomia villarrealii	*Peperomia villarrealii*	43	100	0	0
Peperomia villarrealii	*Peperomia villarrealii*	219	0	0	100
Peperomia villarrealii	*Peperomia villarrealii*	374	40	20	40
Peperomia villarrealii	*Peperomia villarrealii*	21	100	0	0
Peperomia villarrealii	*Peperomia villarrealii*	373	100	0	0
Peperomia villarrealii	*Peperomia villarrealii*	255	50	50	0
Peperomia villicaulis	*Peperomia villicaulis*	29	0	0	100
Peperomia villicaulis	*Peperomia villicaulis*	178	0	50	50
Peperomia villilimba	*Peperomia villilimba*				
Peperomia villipetiola	*Peperomia villipetiola*				
Peperomia villosa	*Peperomia villosa*	373	0	0	100
Peperomia vinasiana	*Peperomia vinasiana*	43	100	0	0
Peperomia vinasiana	*Peperomia vinasiana*	255	50	50	0
Peperomia vinasiana	*Peperomia vinasiana*	37	100	0	0
Peperomia vincentiana	*Peperomia vincentiana*	153	100	0	0
Peperomia vincentiana	*Peperomia vincentiana*	255	100	0	0
Peperomia violacea	*Peperomia violacea*	373	0	0	100
Peperomia viracochana	*Peperomia viracochana*	29	0	0	100
Peperomia vitiana	*Peperomia vitiana*	307	100	0	0
Peperomia vitiana	*Peperomia vitiana*	255	100	0	0
Peperomia vitilevuensis	*Peperomia vitilevuensis*	409	100	0	0
Peperomia vitilevuensis	*Peperomia vitilevuensis*	307	100	0	0
Peperomia vitilevuensis	*Peperomia vitilevuensis*	255	100	0	0
Peperomia vivipara	*Peperomia vivipara*	251	33.3	33.3	33.3
Peperomia vueltasana	*Peperomia vueltasana*	43	100	0	0
Peperomia vueltasana	*Peperomia vueltasana*	255	100	0	0
Peperomia vulcanica	*Peperomia vulcanica*	54	100	0	0
Peperomia vulcanica	*Peperomia vulcanica*	91	5	95	0
Peperomia vulcanica	*Peperomia annobonensis*				
Peperomia vulcanica	*Peperomia hygrophila*				
Peperomia warmingii	*Peperomia warmingii*	82	0	100	0
Peperomia warmingii	*Peperomia warmingii*	113	0	50	50
Peperomia weberbaueri	*Peperomia weberbaueri*	29	0	0	100
Peperomia weberbaueri	*Peperomia weberbaueri*	178	0	50	50
Peperomia weberbaueri	*Peperomia weberbaueri*	379	0	100	0
Peperomia wernerrauhii	*Peperomia wernerrauhii*	292	0	100	0
Peperomia wheeleri	*Peperomia wheeleri*	172	0	100	0
Peperomia wheeleri	*Peperomia wheeleri*	171	0	100	0
Peperomia wibomii	*Peperomia wibomii*	255	100	0	0
Peperomia williamsii	*Peperomia williamsii*	255	100	0	0
Peperomia wolfgang-krahnii	*Peperomia wolfgang-krahnii*	198	0	0	100
Peperomia woytkowskii	*Peperomia woytkowskii*	29	0	0	100
Peperomia wrayi	*Peperomia wrayi*	376	100	0	0
Peperomia wrayi	*Peperomia wrayi*	272	100	0	0
Peperomia wrayi	*Peperomia wrayi*	255	100	0	0
Peperomia xalana	*Peperomia xalana*	43	100	0	0
Peperomia xalana	*Peperomia xalana*	157	100	0	0
Peperomia xalana	*Peperomia xalana*	255	100	0	0
Peperomia yabucoana	*Peperomia yabucoana*	172	0	100	0
Peperomia yabucoana	*Peperomia yabucoana*	171	0	100	0
Peperomia yanacachiana	*Peperomia yanacachiana*	352	0	0	100
Peperomia yananoensis	*Peperomia yananoensis*	29	0	0	100
Peperomia yapasana	*Peperomia yapasana*	29	0	0	100
Peperomia yapasana	*Peperomia yapasana*	178	100	0	0
Peperomia yatuensis	*Peperomia yatuensis*	150	50	50	0
Peperomia yatuensis	*Peperomia yatuensis*	255	50	50	0
Peperomia yeracuiana	*Peperomia yeracuiana*	373	100	0	0
Peperomia yeracuiana	*Peperomia yeracuiana*	255	100	0	0
Peperomia yungasana	*Peperomia yungasana*	309	0	100	0

Peperomia yungasana	*Peperomia yungasana*	169	0	0	100
Peperomia yutajensis	*Peperomia yutajensis*	150	33.3	33.3	33.4
Peperomia yutajensis	*Peperomia yutajensis*	255	50	0	50
Peperomia zarzalana	*Peperomia zarzalana*	150	0	50	50
Peperomia zarzalana	*Peperomia zarzalana*	373	0	50	50
Peperomia zarzalana	*Peperomia zarzalana*	255	0	50	50
Peperomia zipaquirana	*Peperomia zipaquirana*	374	0	33.4	66.6
Peperomia zipaquirana	*Peperomia zipaquirana*	373	0	0	100
Peperomia zongolicana	*Peperomia zongolicana*	157	0	100	0
