# Supplementary material for: “On a tree”, “terrestrial”, or “on the rocks”? Habit diversity in the megadiverse genus Peperomia
Source: Plant Biol (Stuttg). 2026 May 13;28(5):1373–84. doi: 10.1111/plb.70214 (PMC13358651; doi:10.1111/plb.70214)
Supplement: Supplementary file 3 — Table S2. Mean substrate preference value of 1375 species of Peperomia for epiphytic (EV), lithophytic (LV) and terrestrial (TV) growth, including the number of sources for each species and their subgenus (if applicable). Fig. S3. Workflow for data compilation and analysis. An annotated R script for creating the pivot table from the database, and to plot the triangle ordination is included as an upload into the supplementary materials (ELT analysis). [file PLB-28-1373-s002.pdf]

Table S2: Mean substrate preference values for 1375 species of *Peperomia* for epiphytic (EV), lithophytic (LV) and terrestrial growth (TV), including the number of sources for each species and their subgenus (if applicable).

Valid species name	EV (%)	LV (%)	TV (%)	No. of sources	Subgenus
Peperomia abbreviatipes	100,0	0,0	0,0	1	Leptorhynchum
Peperomia abdita	0,0	100,0	0,0	1	NA
Peperomia abnormis	92,5	0,0	7,5	5	Micropiper
Peperomia abscondita	0,0	75,0	25,0	2	Pseudocupula
Peperomia abyssinica	47,6	32,1	20,3	19	Micropiper
Peperomia acaulis	0,0	0,0	100,0	2	NA
Peperomia aceramarcana	50,0	50,0	0,0	2	Pseudocupula
Peperomia aceroana	97,2	0,0	2,8	3	Pseudocupula
Peperomia acreana	75,0	0,0	25,0	4	Oxyrhynchum
Peperomia acuminata	22,4	12,3	65,3	19	Oxyrhynchum
Peperomia adamsonia	50,0	50,0	0,0	3	Micropiper
Peperomia adenocarpa	0,0	0,0	100,0	1	Micropiper
Peperomia adscendens	88,2	0,0	11,8	11	Leptorhynchum
Peperomia adsurgens	50,0	0,0	50,0	2	Micropiper
Peperomia aerea	50,0	0,0	50,0	2	Micropiper
Peperomia aggregata	0,0	98,3	1,7	3	NA
Peperomia aguabonitensis	0,0	0,0	100,0	1	Micropiper
Peperomia aguaditana	0,0	0,0	100,0	1	Micropiper
Peperomia ainana	0,0	0,0	100,0	1	Perlucida
Peperomia alata	53,7	30,9	15,4	29	Micropiper
Peperomia alatiscapa	100,0	0,0	0,0	3	Micropiper
Peperomia albertiae	50,0	50,0	0,0	1	NA
Peperomia albertiana	100,0	0,0	0,0	3	Micropiper
Peperomia albert-smithii	0,0	0,0	100,0	3	Perlucida
Peperomia albidiflora	100,0	0,0	0,0	2	Micropiper
Peperomia albonervosa	0,0	0,0	100,0	2	Tildenia
Peperomia albopilosa	0,0	100,0	0,0	3	NA
Peperomia albovittata	0,0	0,0	100,0	1	Multipalmata
Peperomia aldrinii	100,0	0,0	0,0	1	NA
Peperomia alibacophylla	42,5	7,5	50,0	2	Micropiper
Peperomia alismifolia	0,0	0,0	100,0	2	Multipalmata
Peperomia alpina	36,4	0,0	63,6	11	Oxyrhynchum
Peperomia alternifolia	75,0	25,0	0,0	2	Micropiper
Peperomia alwynii	7,5	0,0	92,5	2	Leptorhynchum
Peperomia ambiguifolia	0,0	0,0	100,0	1	Micropiper
Peperomia amnicola	100,0	0,0	0,0	1	NA
Peperomia amphitricha	37,5	0,0	62,5	4	NA
Peperomia ampla	0,0	0,0	100,0	1	Tildenia
Peperomia amplexifolia	0,0	100,0	0,0	1	NA
Peperomia andicola	100,0	0,0	0,0	3	Pseudocupula
Peperomia andina	0,0	50,0	50,0	1	Tildenia
Peperomia andrei	0,0	50,0	50,0	1	NA

Peperomia angularis	52,3	4,2	43,6	8	Micropiper
Peperomia angustata	74,7	17,0	8,3	26	Micropiper
Peperomia anisophylla	0,0	0,0	100,0	1	Micropiper
Peperomia ankaranensis	0,0	0,0	100,0	1	Peperomia
Peperomia antioquiensis	0,0	100,0	0,0	1	Multipalmata
Peperomia antoniana	0,0	0,0	100,0	2	Multipalmata
Peperomia apiahyensis	0,0	0,0	100,0	2	Micropiper
Peperomia apodophylla	0,0	0,0	100,0	2	Micropiper
Peperomia apurimacana	100,0	0,0	0,0	3	Micropiper
Peperomia arboricola	96,7	3,3	0,0	3	Micropiper
Peperomia arborigaudens	100,0	0,0	0,0	3	Leptorhynchum
Peperomia arboriseda	100,0	0,0	0,0	4	Micropiper
Peperomia arctebaccata	50,0	0,0	50,0	2	Pseudocupula
Peperomia arcuatisicpa	0,0	0,0	100,0	1	NA
Peperomia arenillasensis	100,0	0,0	0,0	2	Micropiper
Peperomia areolata	0,0	50,0	50,0	2	Multipalmata
Peperomia argenteobracteata	50,0	0,0	50,0	2	Pseudocupula
Peperomia argyraea	50,0	0,0	50,0	1	NA
Peperomia argyreia	60,0	0,0	40,0	5	NA
Peperomia argyroneura	0,0	100,0	0,0	1	NA
Peperomia arifolia	14,9	52,3	32,8	14	Multipalmata
Peperomia aristeguietae	50,0	0,0	50,0	2	NA
Peperomia armadana	100,0	0,0	0,0	1	NA
Peperomia armondii	68,8	31,3	0,0	8	Leptorhynchum
Peperomia armstrongii	0,0	0,0	100,0	2	Multipalmata
Peperomia aroensis	100,0	0,0	0,0	2	Leptorhynchum
Peperomia artatiflora	0,0	0,0	100,0	1	Micropiper
Peperomia arthurii	100,0	0,0	0,0	2	Micropiper
Peperomia asarifolia	14,4	32,8	52,8	8	Oxyrhynchum
Peperomia asarifolioides	0,0	66,7	33,3	3	NA
Peperomia asperula	0,0	50,0	50,0	2	Fenestratae
Peperomia asplundii	50,0	0,0	50,0	2	NA
Peperomia asterophylla	100,0	0,0	0,0	2	Pseudocupula
Peperomia attenuata	50,0	0,0	50,0	3	Micropiper
Peperomia augescens	33,4	23,4	43,2	5	Micropiper
Peperomia aurorana	0,0	0,0	100,0	2	Multipalmata
Peperomia austin-smithii	50,0	0,0	50,0	2	Erasmia
Peperomia australana	50,0	37,5	12,5	4	Micropiper
Peperomia ayacuchoana	0,0	0,0	100,0	1	Tildenia
Peperomia bajana	0,0	0,0	100,0	1	Micropiper
Peperomia balansana	11,9	20,8	67,3	4	Multipalmata
Peperomia balfourii	100,0	0,0	0,0	1	Micropiper
Peperomia bamleri	100,0	0,0	0,0	2	NA
Peperomia bangii	0,0	0,0	100,0	1	Micropiper
Peperomia bangroana	75,0	0,0	25,0	2	Micropiper
Peperomia barahonana	50,0	50,0	0,0	1	Micropiper
Peperomia barbarana	24,0	2,0	74,0	5	Peperomia

Peperomia barbaranoides	100,0	0,0	0,0	1	NA
Peperomia barbata	0,0	100,0	0,0	1	Micropiper
Peperomia barbulata	50,0	0,0	50,0	2	NA
Peperomia baronii	100,0	0,0	0,0	1	Micropiper
Peperomia barryana	0,0	0,0	100,0	1	NA
Peperomia basiradicans	0,0	50,0	50,0	2	Tildenia
Peperomia bavina	0,0	50,0	50,0	2	Pseudocupula
Peperomia bella	100,0	0,0	0,0	1	Leptorhynchum
Peperomia bellatula	0,0	50,0	50,0	1	NA
Peperomia bellendenkerensis	15,0	85,0	0,0	1	NA
Peperomia berlandieri	60,0	20,0	20,0	5	Pseudocupula
Peperomia bermudezana	100,0	0,0	0,0	3	Micropiper
Peperomia bernhardiana	0,0	50,0	50,0	3	NA
Peperomia bernieriana	100,0	0,0	0,0	1	Micropiper
Peperomia bernoullii	0,0	41,7	58,3	3	NA
Peperomia berryi	50,0	25,0	25,0	2	Pseudocupula
Peperomia berteroana	0,0	30,0	70,0	2	Micropiper
Peperomia biamenta	100,0	0,0	0,0	1	Leptorhynchum
Peperomia bicolor	33,3	33,3	33,3	3	Micropiper
Peperomia biformis	0,0	0,0	100,0	1	Micropiper
Peperomia bilobulata	0,0	0,0	100,0	1	Multipalmata
Peperomia bismarckiana	100,0	0,0	0,0	1	Micropiper
Peperomia blackii	100,0	0,0	0,0	3	NA
Peperomia blanda	28,0	28,7	43,3	38	Micropiper
Peperomia blephariphylla	71,7	0,0	28,3	3	Leptorhynchum
Peperomia blepharipus	100,0	0,0	0,0	3	NA
Peperomia boekei	0,0	0,0	100,0	1	Micropiper
Peperomia boivinii	100,0	0,0	0,0	1	Micropiper
Peperomia boliviensis	100,0	0,0	0,0	1	Pseudocupula
Peperomia boninsimensis	50,0	50,0	0,0	1	Micropiper
Peperomia bopiana	100,0	0,0	0,0	1	Micropiper
Peperomia borbonensis	100,0	0,0	0,0	1	Micropiper
Peperomia borburatensis	0,0	0,0	100,0	1	Leptorhynchum
Peperomia botterii	83,3	0,0	16,7	3	Micropiper
Peperomia bourneae	100,0	0,0	0,0	1	Micropiper
Peperomia brachypoda	100,0	0,0	0,0	2	Micropiper
Peperomia bracteata	13,8	31,9	54,4	4	Tildenia
Peperomia bracteispica	100,0	0,0	0,0	3	Micropiper
Peperomia bradei	100,0	0,0	0,0	3	Micropiper
Peperomia brasiliensis	50,0	0,0	50,0	4	NA
Peperomia breedlovei	0,0	50,0	50,0	2	NA
Peperomia brevihirtella	100,0	0,0	0,0	1	Micropiper
Peperomia breviramula	100,0	0,0	0,0	2	Micropiper
Peperomia brittonii	0,0	0,0	100,0	1	NA
Peperomia brouetiana	100,0	0,0	0,0	1	Pseudocupula
Peperomia bryophila	100,0	0,0	0,0	1	Micropiper
Peperomia buchtienii	50,0	0,0	50,0	2	Micropiper

Peperomia buxifolia	0,0	0,0	100,0	1	Micropiper
Peperomia cacaophila	70,0	20,0	10,0	5	Micropiper
Peperomia cachabiana	68,8	0,0	31,3	4	NA
Peperomia caducifolia	0,0	0,0	100,0	1	Micropiper
Peperomia caducipilosa	100,0	0,0	0,0	1	Leptorhynchum
Peperomia caespitosa	64,3	0,0	35,7	3	Pleurocarpidium
Peperomia cainarachiana	62,5	0,0	37,5	4	NA
Peperomia calcicola	33,3	66,7	0,0	3	NA
Peperomia caledonica	100,0	0,0	0,0	1	Micropiper
Peperomia caliginigaudens	0,0	33,3	66,7	3	Leptorhynchum
Peperomia callana	0,0	50,0	50,0	1	Fenestratae
Peperomia callejasii	0,0	0,0	100,0	1	NA
Peperomia callitrichoides	100,0	0,0	0,0	1	NA
Peperomia calophylla	0,0	0,0	100,0	2	NA
Peperomia calvescens	0,0	0,0	100,0	1	Micropiper
Peperomia calvicaulis	66,7	33,3	0,0	3	Leptorhynchum
Peperomia campana	100,0	0,0	0,0	2	NA
Peperomia campinasana	70,0	30,0	0,0	5	Pseudocupula
Peperomia camposii	0,0	0,0	100,0	1	Multipalmata
Peperomia camptotricha	50,0	50,0	0,0	1	Micropiper
Peperomia canalensis	0,0	100,0	0,0	1	Micropiper
Peperomia canaminana	100,0	0,0	0,0	1	Pseudocupula
Peperomia candelaber	83,3	0,0	16,7	3	Micropiper
Peperomia candida	50,0	50,0	0,0	1	Micropiper
Peperomia caniana	0,0	0,0	100,0	1	Micropiper
Peperomia canlaonensis	100,0	0,0	0,0	2	NA
Peperomia caperata	0,0	0,0	100,0	1	Multipalmata
Peperomia capitis-bovis	100,0	0,0	0,0	1	Leptorhynchum
Peperomia caraboboensis	0,0	0,0	100,0	2	NA
Peperomia cardenasii	100,0	0,0	0,0	2	Pseudocupula
Peperomia carnevalii	0,0	0,0	100,0	1	Micropiper
Peperomia carnifolia	100,0	0,0	0,0	2	Pseudocupula
Peperomia carpapatana	0,0	0,0	100,0	1	Micropiper
Peperomia carpinterana	66,7	0,0	33,3	3	Micropiper
Peperomia casapiana	50,0	0,0	50,0	2	Micropiper
Peperomia casarettoi	100,0	0,0	0,0	1	Leptorhynchum
Peperomia castelosensis	100,0	0,0	0,0	3	Pseudocupula
Peperomia castilloi	100,0	0,0	0,0	1	NA
Peperomia catesbaeifolia	100,0	0,0	0,0	1	NA
Peperomia catharinae	92,8	7,2	0,0	9	Micropiper
Peperomia catharinensis	50,0	0,0	50,0	1	Micropiper
Peperomia caucana	50,0	0,0	50,0	2	Micropiper
Peperomia cavaleriei	75,0	25,0	0,0	4	Micropiper
Peperomia cavispicata	50,0	50,0	0,0	2	Tildenia
Peperomia celiae	33,3	0,0	66,7	3	Leptorhynchum
Peperomia cerea	0,0	50,0	50,0	1	Micropiper
Peperomia cereoides	0,0	100,0	0,0	2	Fenestratae

Peperomia ceroderma	75,0	25,0	0,0	2	Leptorhynchum
Peperomia cerrateae	0,0	0,0	100,0	2	Tildenia
Peperomia chalhuapuquiana	100,0	0,0	0,0	1	Micropiper
Peperomia chanchamayana	100,0	0,0	0,0	3	Micropiper
Peperomia chapensis	100,0	0,0	0,0	2	Oxyrhynchum
Peperomia chazaroi	0,0	100,0	0,0	1	NA
Peperomia chicamochana	0,0	50,0	50,0	1	Pleurocarpidium
Peperomia chigorodoana	100,0	0,0	0,0	2	Leptorhynchum
Peperomia chimboana	50,0	0,0	50,0	2	Micropiper
Peperomia chlorodisca	100,0	0,0	0,0	2	Micropiper
Peperomia choritana	57,5	0,0	42,5	2	Micropiper
Peperomia choroniana	44,3	10,0	45,7	10	Leptorhynchum
Peperomia christophersenii	0,0	0,0	100,0	1	Micropiper
Peperomia chrysotricha	0,0	0,0	100,0	1	Micropiper
Peperomia chutanka	0,0	100,0	0,0	1	Tildenia
Peperomia ciezae	0,0	100,0	0,0	1	Fenestratae
Peperomia ciliaris	80,0	0,0	20,0	5	Micropiper
Peperomia ciliatifolia	75,0	0,0	25,0	4	Leptorhynchum
Peperomia ciliatocaespitosa	0,0	62,5	37,5	4	NA
Peperomia ciliifolia	0,0	0,0	100,0	1	Micropiper
Peperomia ciliolibractea	61,3	12,5	26,3	4	Oxyrhynchum
Peperomia ciliosa	100,0	0,0	0,0	1	Leptorhynchum
Peperomia circinnata	87,5	6,3	6,3	16	Pseudocupula
Peperomia circulifolia	100,0	0,0	0,0	1	Pseudocupula
Peperomia cirillii-nelsonii	0,0	0,0	100,0	2	NA
Peperomia cladara	0,0	100,0	0,0	4	Pseudocupula
Peperomia claudii	100,0	0,0	0,0	3	Pseudocupula
Peperomia claussenii	0,0	0,0	100,0	2	NA
Peperomia clavatispica	0,0	0,0	100,0	2	Multipalmata
Peperomia clavigera	0,0	50,0	50,0	4	Leptorhynchum
Peperomia claytonioides	16,7	46,7	36,7	5	Tildenia
Peperomia clivicola	62,5	37,5	0,0	4	Micropiper
Peperomia clivigaudens	0,0	0,0	100,0	1	Multipalmata
Peperomia clusiifolia	66,7	16,7	16,7	2	Oxyrhynchum
Peperomia coatzacoalcosensis	100,0	0,0	0,0	3	Micropiper
Peperomia cobana	86,0	9,0	5,0	5	Oxyrhynchum
Peperomia cochinensis	100,0	0,0	0,0	3	NA
Peperomia cocleana	66,7	0,0	33,3	3	NA
Peperomia coenosa	0,0	0,0	100,0	1	NA
Peperomia cogniauxii	75,0	12,5	12,5	4	Micropiper
Peperomia collinsii	92,5	0,0	7,5	2	Micropiper
Peperomia collocata	85,0	0,0	15,0	1	Micropiper
Peperomia coloniae	0,0	0,0	100,0	1	Micropiper
Peperomia colorata	0,0	0,0	100,0	2	Micropiper
Peperomia columella	0,0	25,0	75,0	2	Fenestratae
Peperomia columnaris	0,0	50,0	50,0	1	Fenestratae
Peperomia comaltitlanensis	0,0	50,0	50,0	2	NA

Peperomia comarapana	11,2	0,0	88,8	5	Micropiper
Peperomia commersonii	100,0	0,0	0,0	1	Micropiper
Peperomia concava	0,0	0,0	100,0	1	Micropiper
Peperomia condoris	0,0	0,0	100,0	1	Micropiper
Peperomia condormiens	85,0	0,0	15,0	1	NA
Peperomia confertispica	0,0	0,0	100,0	3	Micropiper
Peperomia congerro	0,0	0,0	100,0	1	NA
Peperomia congesta	0,0	50,0	50,0	2	Fenestratae
Peperomia congestispica	0,0	0,0	100,0	1	Micropiper
Peperomia conjugata	0,0	50,0	50,0	1	NA
Peperomia connixa	0,0	15,0	85,0	2	Multipalmata
Peperomia conocarpa	71,7	0,0	28,3	3	Pseudocupula
Peperomia consoquitlana	66,7	16,7	16,7	3	Micropiper
Peperomia convexa	83,3	16,7	0,0	3	NA
Peperomia cookiana	26,7	6,7	66,7	5	Micropiper
Peperomia coquimbensis	100,0	0,0	0,0	1	Micropiper
Peperomia corcovadensis	68,5	24,1	7,4	18	Micropiper
Peperomia cordata	0,0	0,0	100,0	1	Multipalmata
Peperomia cordigera	66,7	33,3	0,0	3	Pseudocupula
Peperomia cordovana	100,0	0,0	0,0	2	Micropiper
Peperomia cordulata	100,0	0,0	0,0	5	Oxyrhynchum
Peperomia cordulatiformis	50,0	0,0	50,0	6	NA
Peperomia cordulilimba	100,0	0,0	0,0	3	Micropiper
Peperomia coroicoensis	50,0	0,0	50,0	1	Micropiper
Peperomia costata	75,0	0,0	25,0	2	Micropiper
Peperomia cotoneasterifolia	50,0	0,0	50,0	2	Micropiper
Peperomia cotyledon	0,0	0,0	100,0	1	Panicularia
Peperomia coulteri	100,0	0,0	0,0	1	Micropiper
Peperomia cowanii	100,0	0,0	0,0	1	Pseudocupula
Peperomia crassicaulis	75,0	0,0	25,0	2	Oxyrhynchum
Peperomia crassispica	100,0	0,0	0,0	1	Pseudocupula
Peperomia crassulicaulis	0,0	0,0	100,0	1	Micropiper
Peperomia crinicaulis	75,0	25,0	0,0	6	Pseudocupula
Peperomia crinigera	0,0	0,0	100,0	1	Micropiper
Peperomia crispa	0,0	0,0	100,0	2	Perlucida
Peperomia crispipetiola	100,0	0,0	0,0	4	Micropiper
Peperomia croizatiana	100,0	0,0	0,0	2	Micropiper
Peperomia crotalophora	20,0	0,0	80,0	5	Leptorhynchum
Peperomia cruentata	100,0	0,0	0,0	2	NA
Peperomia crusculibacca	0,0	0,0	100,0	1	Micropiper
Peperomia cruzeirensis	100,0	0,0	0,0	3	NA
Peperomia crypticola	0,0	100,0	0,0	3	Oxyrhynchum
Peperomia cryptostachya	100,0	0,0	0,0	2	Leptorhynchum
Peperomia crystallina	7,1	21,4	71,4	7	Phyllobryon
Peperomia cuatrecasasana	100,0	0,0	0,0	1	Micropiper
Peperomia cubensis	50,0	25,0	25,0	2	NA
Peperomia cubugonana	100,0	0,0	0,0	2	Pseudocupula

Peperomia cuchumatanica	0,0	0,0	100,0	2	Tildenia
Peperomia cumbreana	100,0	0,0	0,0	2	NA
Peperomia cundinamarcana	0,0	0,0	100,0	1	Oxyrhynchum
Peperomia cuprea	33,3	0,0	66,7	3	Leptorhynchum
Peperomia curruciformis	0,0	0,0	100,0	2	Leptorhynchum
Peperomia curticaulis	0,0	0,0	100,0	1	Multipalmata
Peperomia curtipes	0,0	0,0	100,0	3	Multipalmata
Peperomia curtispica	100,0	0,0	0,0	3	Micropiper
Peperomia cushiana	50,0	0,0	50,0	2	Micropiper
Peperomia cusilluyocana	0,0	0,0	100,0	1	Micropiper
Peperomia cuspidata	50,0	0,0	50,0	2	NA
Peperomia cuspidilimba	50,0	0,0	50,0	2	Pseudocupula
Peperomia cyclaminoides	0,0	0,0	100,0	1	Tildenia
Peperomia cyclophylla	68,2	4,5	27,3	11	Pseudocupula
Peperomia cymbifolia	0,0	87,5	12,5	4	Fenestratae
Peperomia daguana	100,0	0,0	0,0	1	Leptorhynchum
Peperomia dahlstedtii	75,0	12,5	12,5	4	Pseudocupula
Peperomia damazioi	100,0	0,0	0,0	1	Micropiper
Peperomia darienensis	50,0	50,0	0,0	2	NA
Peperomia dasystachya	0,0	0,0	100,0	1	Micropiper
Peperomia dauleana	50,0	0,0	50,0	2	Micropiper
Peperomia davidsoniae	0,0	0,0	100,0	2	NA
Peperomia debilipes	75,0	0,0	25,0	4	Micropiper
Peperomia deceptrix	100,0	0,0	0,0	3	Pseudocupula
Peperomia decipiens	0,0	50,0	50,0	1	NA
Peperomia decora	33,3	66,7	0,0	3	Pseudocupula
Peperomia decumbens	50,0	0,0	50,0	2	Micropiper
Peperomia decurrens	0,0	75,0	25,0	2	Oxyrhynchum
Peperomia deficiens	0,0	0,0	100,0	1	NA
Peperomia defoliata	50,0	0,0	50,0	2	Micropiper
Peperomia degeneri	50,0	50,0	0,0	2	Micropiper
Peperomia delascioi	33,3	0,0	66,7	3	Pleurocarpidium
Peperomia delicatula	100,0	0,0	0,0	7	Pseudocupula
Peperomia dendrophila	67,3	3,8	28,8	13	Micropiper
Peperomia densifolia	0,0	0,0	100,0	1	NA
Peperomia dependens	20,8	20,8	58,4	4	Multipalmata
Peperomia deppeana	95,8	4,2	0,0	12	Pseudocupula
Peperomia diamantinensis	16,7	33,3	50,0	3	NA
Peperomia diaphanoides	75,0	0,0	25,0	6	Micropiper
Peperomia dichotoma	100,0	0,0	0,0	3	NA
Peperomia diffusa	0,0	0,0	100,0	1	Micropiper
Peperomia dimota	50,0	0,0	50,0	2	Micropiper
Peperomia discifolia	50,0	0,0	50,0	2	Micropiper
Peperomia discilimba	25,0	0,0	75,0	2	Multipalmata
Peperomia disjunctiflora	50,0	0,0	50,0	2	Micropiper
Peperomia distachyos	50,5	20,6	29,0	23	NA
Peperomia disticha	100,0	0,0	0,0	3	Micropiper

Peperomia divaricata	0,0	100,0	0,0	1	Pseudocupula
Peperomia diversifolia	0,0	100,0	0,0	1	Micropiper
Peperomia dolabella	0,0	0,0	100,0	2	Tildenia
Peperomia dolabriformis	0,0	42,9	57,1	7	Fenestratae
Peperomia dominicana	100,0	0,0	0,0	1	Micropiper
Peperomia donaguiana	55,5	22,2	22,3	3	Micropiper
Peperomia donnell-smithii	100,0	0,0	0,0	1	NA
Peperomia dorstenioides	0,0	50,0	50,0	3	Leptorhynchum
Peperomia dotana	37,5	0,0	62,5	4	Oxyrhynchum
Peperomia drapeta	66,7	0,0	33,3	3	Micropiper
Peperomia drusophila	100,0	0,0	0,0	2	Micropiper
Peperomia dryadica	5,0	95,0	0,0	1	NA
Peperomia dryadum	100,0	0,0	0,0	1	Micropiper
Peperomia duartei	0,0	0,0	100,0	2	NA
Peperomia dubia	100,0	0,0	0,0	3	Micropiper
Peperomia duendensis	50,0	0,0	50,0	2	Micropiper
Peperomia duidana	100,0	0,0	0,0	2	NA
Peperomia durandii	0,0	0,0	100,0	1	Oxyrhynchum
Peperomia duricaulis	50,0	0,0	50,0	3	NA
Peperomia dusenii	100,0	0,0	0,0	2	Micropiper
Peperomia dyscrita	50,0	50,0	0,0	2	NA
Peperomia ebingeri	85,7	0,0	14,3	7	Micropiper
Peperomia eburnea	60,0	0,0	40,0	1	Leptorhynchum
Peperomia ecuadorensis	0,0	0,0	100,0	1	Multipalmata
Peperomia edulis	100,0	0,0	0,0	1	Pseudocupula
Peperomia eekana	100,0	0,0	0,0	2	Micropiper
Peperomia effusa	50,0	0,0	50,0	2	Micropiper
Peperomia efimbriata	100,0	0,0	0,0	1	Pseudocupula
Peperomia eggersii	92,5	0,0	7,5	2	Micropiper
Peperomia egleri	100,0	0,0	0,0	2	NA
Peperomia ekakesara	0,0	100,0	0,0	2	NA
Peperomia elata	40,0	20,0	40,0	5	Micropiper
Peperomia elatior	0,0	100,0	0,0	1	Tildenia
Peperomia elegantifolia	0,0	0,0	100,0	1	Oxyrhynchum
Peperomia elliptica	100,0	0,0	0,0	1	Micropiper
Peperomia ellipticibacca	50,0	0,0	50,0	2	Micropiper
Peperomia ellipticorhombea	50,0	0,0	50,0	2	NA
Peperomia ellsworthii	50,0	0,0	50,0	2	Multipalmata
Peperomia elmeri	100,0	0,0	0,0	3	Micropiper
Peperomia elongata	85,2	4,6	10,2	18	Leptorhynchum
Peperomia elsana	0,0	0,0	100,0	1	Multipalmata
Peperomia elsieae	69,0	4,8	26,2	7	NA
Peperomia emarginatifolia	100,0	0,0	0,0	1	NA
Peperomia emarginella	81,3	17,4	1,3	23	Pleurocarpidium
Peperomia emarginulata	55,9	11,4	32,7	7	Micropiper
Peperomia emiliana	93,5	5,0	1,5	10	Pseudocupula
Peperomia endlichii	100,0	0,0	0,0	2	NA

Peperomia enenyasensis	66,7	0,0	33,3	3	Leptorhynchum
Peperomia enervis	50,0	50,0	0,0	1	Micropiper
Peperomia epidendron	66,7	0,0	33,3	3	Micropiper
Peperomia epipetrica	0,0	50,0	50,0	2	NA
Peperomia epipremnifolia	10,0	0,0	90,0	3	NA
Peperomia eripipunctulata	100,0	0,0	0,0	1	Micropiper
Peperomia erosa	0,0	50,0	50,0	1	Fenestratae
Peperomia erythrocaulis	0,0	0,0	100,0	1	Micropiper
Peperomia erythropremna	33,3	50,0	16,7	3	Micropiper
Peperomia erythrospicata	100,0	0,0	0,0	2	NA
Peperomia erythrostachya	60,0	0,0	40,0	5	Leptorhynchum
Peperomia esmeraldana	50,0	0,0	50,0	2	NA
Peperomia esperanzana	83,8	0,0	16,3	4	Micropiper
Peperomia espinosae	0,0	50,0	50,0	2	NA
Peperomia estaminea	100,0	0,0	0,0	1	Micropiper
Peperomia estrellana	100,0	0,0	0,0	3	Pseudocupula
Peperomia ewanii	50,0	0,0	50,0	2	Micropiper
Peperomia exclamationis	0,0	100,0	0,0	1	NA
Peperomia exigua	0,0	96,7	3,3	3	NA
Peperomia exiguispica	50,0	0,0	50,0	2	Leptorhynchum
Peperomia exilamenta	50,0	0,0	50,0	3	Pseudocupula
Peperomia exiliramea	66,7	0,0	33,3	3	Leptorhynchum
Peperomia expallescens	75,0	0,0	25,0	2	NA
Peperomia fagerlindii	100,0	0,0	0,0	1	Leptorhynchum
Peperomia falanana	0,0	0,0	100,0	1	Perlucida
Peperomia falcata	100,0	0,0	0,0	3	Micropiper
Peperomia falconensis	0,0	50,0	50,0	2	Multipalmata
Peperomia falsa	0,0	25,0	75,0	2	NA
Peperomia famelica	0,0	0,0	100,0	2	Micropiper
Peperomia farctifolia	100,0	0,0	0,0	2	Leptorhynchum
Peperomia fawcettii	100,0	0,0	0,0	2	Micropiper
Peperomia fendleriana	33,3	33,3	33,3	3	Micropiper
Peperomia fernandeziana	44,4	11,1	44,5	3	Micropiper
Peperomia fernandopoiana	70,5	13,0	16,5	10	Micropiper
Peperomia ferreyrae	16,7	50,0	33,3	3	Fenestratae
Peperomia ficta	66,7	0,0	33,3	3	Pseudocupula
Peperomia filicaulis	100,0	0,0	0,0	2	Micropiper
Peperomia filiformis	83,3	0,0	16,7	3	Pseudocupula
Peperomia fissicola	0,0	50,0	50,0	2	Fenestratae
Peperomia fissispica	100,0	0,0	0,0	3	NA
Peperomia flabilis	50,0	0,0	50,0	2	Pseudocupula
Peperomia flavamenta	50,0	0,0	50,0	2	Pseudocupula
Peperomia flavescens	33,3	0,0	66,7	3	Leptorhynchum
Peperomia flavescentifolia	66,7	0,0	33,3	3	Leptorhynchum
Peperomia flavida	100,0	0,0	0,0	2	Micropiper
Peperomia flexicaulis	100,0	0,0	0,0	3	Micropiper
Peperomia flexinervia	50,0	0,0	50,0	2	NA

Peperomia fluviatilis	100,0	0,0	0,0	3	Pseudocupula
Peperomia foliata	0,0	0,0	100,0	1	Micropiper
Peperomia foliiflora	0,0	33,3	66,7	3	Phyllobryon
Peperomia foliosa	0,0	0,0	100,0	1	Micropiper
Peperomia folsomii	100,0	0,0	0,0	2	NA
Peperomia foraminum	0,0	100,0	0,0	2	Micropiper
Peperomia fosbergii	72,5	27,5	0,0	2	Micropiper
Peperomia fournieri	50,0	17,0	33,0	2	Micropiper
Peperomia fragilis	50,0	0,0	50,0	2	Micropiper
Peperomia fragilissima	0,0	0,0	100,0	1	Multipalmata
Peperomia fragrans	0,0	50,0	50,0	3	Micropiper
Peperomia franciscoi	0,0	50,0	50,0	2	NA
Peperomia fraseri	18,5	20,0	61,6	9	Panicularia
Peperomia fruticetorum	15,0	0,0	85,0	2	NA
Peperomia fulvescens	100,0	0,0	0,0	2	Leptorhynchum
Peperomia fundacionensis	44,4	11,1	44,5	3	Leptorhynchum
Peperomia fundus-oculi	0,0	50,0	50,0	1	Tildenia
Peperomia furcata	100,0	0,0	0,0	1	Leptorhynchum
Peperomia fuscipunctata	25,0	0,0	75,0	2	Micropiper
Peperomia fuscispica	100,0	0,0	0,0	3	Micropiper
Peperomia gabinetensis	100,0	0,0	0,0	2	Micropiper
Peperomia galapagensis	66,7	16,7	16,7	3	Micropiper
Peperomia galioides	32,8	19,2	48,0	40	Micropiper
Peperomia garcia-barrigana	0,0	0,0	100,0	1	Micropiper
Peperomia gardneriana	0,0	50,0	50,0	3	Multipalmata
Peperomia gaultheriifolia	50,0	0,0	50,0	2	NA
Peperomia gayi	50,0	0,0	50,0	2	Micropiper
Peperomia gedehana	100,0	0,0	0,0	1	NA
Peperomia gehrigeri	50,0	0,0	50,0	3	Pseudocupula
Peperomia gemella	65,0	35,0	0,0	3	NA
Peperomia geminispica	66,7	33,3	0,0	3	NA
Peperomia gentryi	0,0	0,0	100,0	2	Leptorhynchum
Peperomia gerardoi	0,0	100,0	0,0	2	NA
Peperomia gibba	0,0	0,0	100,0	1	NA
Peperomia gigantea	0,0	0,0	100,0	1	Tildenia
Peperomia giralana	0,0	0,0	100,0	1	Multipalmata
Peperomia glabella	70,8	14,3	14,8	51	Micropiper
Peperomia glabrilimba	100,0	0,0	0,0	1	Micropiper
Peperomia glabrior	50,0	0,0	50,0	1	NA
Peperomia glabrirhachis	50,0	0,0	50,0	2	Pseudocupula
Peperomia glandulosa	50,0	0,0	50,0	2	Micropiper
Peperomia glareosa	66,7	0,0	33,3	3	Pseudocupula
Peperomia glassmanii	93,8	6,3	0,0	4	Micropiper
Peperomia glauca	0,0	50,0	50,0	2	Micropiper
Peperomia glazioui	68,8	31,3	0,0	8	Micropiper
Peperomia gleicheniiformis	100,0	0,0	0,0	4	Micropiper
Peperomia globosibacca	0,0	50,0	50,0	1	NA

Peperomia globulanthera	50,0	0,0	50,0	3	Micropiper
Peperomia gloriosifolia	100,0	0,0	0,0	1	Micropiper
Peperomia glutinosa	56,7	21,7	21,7	3	NA
Peperomia gorgonillana	100,0	0,0	0,0	4	Micropiper
Peperomia goudotii	50,0	50,0	0,0	1	Pseudocupula
Peperomia gracieana	32,9	29,2	38,0	4	NA
Peperomia gracilicaulis	100,0	0,0	0,0	3	NA
Peperomia gracilis	75,0	25,0	0,0	4	Pseudocupula
Peperomia gracilispica	0,0	0,0	100,0	1	Leptorhynchum
Peperomia gracillima	0,0	0,0	100,0	2	Tildenia
Peperomia grantii	100,0	0,0	0,0	2	Micropiper
Peperomia granulata	100,0	0,0	0,0	2	Leptorhynchum
Peperomia granulatifolia	83,3	0,0	16,7	3	Micropiper
Peperomia granulatilimba	100,0	0,0	0,0	3	Leptorhynchum
Peperomia granulosa	81,0	9,0	10,0	10	Leptorhynchum
Peperomia graveolens	0,0	50,0	50,0	1	NA
Peperomia grayumii	100,0	0,0	0,0	2	NA
Peperomia griggsii	62,5	12,5	25,0	4	Leptorhynchum
Peperomia grisarii	0,0	0,0	100,0	2	Multipalmata
Peperomia grisebachii	100,0	0,0	0,0	1	Pseudocupula
Peperomia gruendleri	100,0	0,0	0,0	1	Micropiper
Peperomia guadaloupensis	17,5	57,9	24,6	6	Micropiper
Peperomia guadalupana	100,0	0,0	0,0	2	Pseudocupula
Peperomia guaiquinimana	100,0	0,0	0,0	3	Micropiper
Peperomia guamana	0,0	100,0	0,0	1	Micropiper
Peperomia guanensis	0,0	50,0	50,0	2	Micropiper
Peperomia guapilesiana	50,0	0,0	50,0	4	Erasmia
Peperomia guatemalensis	70,8	8,3	20,9	4	Micropiper
Peperomia guayrapurana	50,0	0,0	50,0	2	Pseudocupula
Peperomia gucayana	0,0	0,0	100,0	1	Micropiper
Peperomia gutierrezana	25,0	0,0	75,0	4	Leptorhynchum
Peperomia guttulata	66,7	0,0	33,3	3	NA
Peperomia gymnophylla	41,5	16,5	42,0	2	Multipalmata
Peperomia hadrostachya	0,0	0,0	100,0	1	Multipalmata
Peperomia haematolepis	42,9	35,7	21,4	7	Oxyrhynchum
Peperomia haenkeana	0,0	0,0	100,0	2	Phyllobryon
Peperomia hallieri	100,0	0,0	0,0	1	NA
Peperomia hammelii	0,0	66,7	33,3	3	Multipalmata
Peperomia hanaensis	0,0	0,0	100,0	1	Micropiper
Peperomia harlingii	100,0	0,0	0,0	1	NA
Peperomia harmandii	100,0	0,0	0,0	1	Micropiper
Peperomia harrisii	66,7	16,7	16,7	2	Leptorhynchum
Peperomia hartmannii	100,0	0,0	0,0	1	NA
Peperomia hartwegiana	42,5	29,2	28,3	13	Pseudocupula
Peperomia haughtii	100,0	0,0	0,0	2	Pseudocupula
Peperomia hebetata	100,0	0,0	0,0	2	NA
Peperomia hedyotidea	100,0	0,0	0,0	1	Micropiper

Peperomia hemmendorffii	0,0	0,0	100,0	2	Micropiper
Peperomia hendersonensis	100,0	0,0	0,0	1	Micropiper
Peperomia heptaphylla	50,0	50,0	0,0	3	NA
Peperomia hernandiifolia	49,8	7,3	42,9	16	Leptorhynchum
Peperomia herrerae	0,0	0,0	100,0	1	Micropiper
Peperomia herzogii	100,0	0,0	0,0	2	Pseudocupula
Peperomia hesperomannii	32,5	0,0	67,5	2	Micropiper
Peperomia heterodoxa	47,5	21,3	31,3	4	Micropiper
Peperomia heterophylla	70,8	3,1	26,0	16	Micropiper
Peperomia heterostachya	0,0	0,0	100,0	1	NA
Peperomia heyneana	62,5	37,5	0,0	8	Micropiper
Peperomia hilariana	13,7	36,1	50,2	6	Micropiper
Peperomia hildebrandtii	100,0	0,0	0,0	1	Micropiper
Peperomia hintonii	57,5	42,5	0,0	2	Hispidula
Peperomia hirta	59,2	10,4	30,4	12	Oxyrhynchum
Peperomia hirtella	62,5	0,0	37,5	4	Micropiper
Peperomia hirtellicaulis	50,0	0,0	50,0	2	Micropiper
Peperomia hirticaulis	100,0	0,0	0,0	1	Micropiper
Peperomia hirtipeduncula	0,0	0,0	100,0	1	Micropiper
Peperomia hirtipetiola	0,0	0,0	100,0	3	Micropiper
Peperomia hirtulicaulis	50,0	50,0	0,0	1	NA
Peperomia hispidorhachis	100,0	0,0	0,0	1	Oxyrhynchum
Peperomia hispidosa	44,7	27,7	27,7	3	Pseudocupula
Peperomia hispidula	25,7	13,8	60,5	28	Hispidula
Peperomia hispiduliformis	33,3	11,9	54,8	7	Hispidula
Peperomia hobbitoides	5,0	95,0	0,0	2	Oxyrhynchum
Peperomia hodgei	16,7	16,7	66,7	3	Multipalmata
Peperomia hoffmannii	70,7	10,2	19,1	13	Pseudocupula
Peperomia hombronii	65,0	35,0	0,0	3	NA
Peperomia hondoana	0,0	100,0	0,0	1	Micropiper
Peperomia honigii	0,0	0,0	100,0	1	NA
Peperomia huacapistanana	50,0	0,0	50,0	2	Multipalmata
Peperomia huallagana	75,0	0,0	25,0	4	Micropiper
Peperomia huantana	0,0	0,0	100,0	2	Pseudocupula
Peperomia huanucoana	0,0	50,0	50,0	1	Micropiper
Peperomia huatuscoana	50,0	50,0	0,0	1	NA
Peperomia huberi	33,3	0,0	66,7	3	Multipalmata
Peperomia humbertii	75,0	0,0	25,0	2	Micropiper
Peperomia humifusa	100,0	0,0	0,0	1	Leptorhynchum
Peperomia humilis	8,3	25,0	66,7	6	Micropiper
Peperomia hunteriana	0,0	100,0	0,0	1	Micropiper
Peperomia hutchisonii	0,0	50,0	50,0	1	Fenestratae
Peperomia hydnostachya	50,0	0,0	50,0	1	NA
Peperomia hydrocotyloides	0,0	0,0	100,0	1	Multipalmata
Peperomia hygrophiloides	75,0	0,0	25,0	4	NA
Peperomia hylophila	92,1	0,0	7,9	7	Micropiper
Peperomia hypoleuca	75,0	0,0	25,0	2	Micropiper

Peperomia hyporhoda	50,0	0,0	50,0	2	Micropiper
Peperomia ibiramana	33,5	33,0	33,5	2	Multipalmata
Peperomia ilaloensis	36,7	6,7	56,7	5	Micropiper
Peperomia imerinae	0,0	0,0	100,0	1	Micropiper
Peperomia immolata	0,0	100,0	0,0	1	Multipalmata
Peperomia inaequalifolia	48,8	13,8	37,5	12	Micropiper
Peperomia inaequalilimba	0,0	0,0	100,0	1	Micropiper
Peperomia inaequilatera	0,0	0,0	100,0	1	Micropiper
Peperomia incana	36,7	56,7	6,7	5	Leptorhynchum
Peperomia incisa	100,0	0,0	0,0	3	Micropiper
Peperomia incognita	0,0	0,0	100,0	1	NA
Peperomia inconspicua	0,0	0,0	100,0	1	NA
Peperomia increscens	35,8	23,8	40,4	6	NA
Peperomia induratifolia	50,0	0,0	50,0	2	Micropiper
Peperomia infralutea	100,0	0,0	0,0	2	Micropiper
Peperomia infravillosa	0,0	100,0	0,0	1	Micropiper
Peperomia inquilina	100,0	0,0	0,0	1	Pseudocupula
Peperomia insueta	0,0	16,5	83,5	2	Multipalmata
Peperomia inversa	0,0	100,0	0,0	1	NA
Peperomia involucrata	0,0	0,0	100,0	1	NA
Peperomia irrasa	0,0	0,0	100,0	1	NA
Peperomia itatiaiana	50,0	50,0	0,0	1	Micropiper
Peperomia itayana	0,0	0,0	100,0	1	Multipalmata
Peperomia jalcaensis	0,0	0,0	100,0	1	Tildenia
Peperomia jamesoniana	94,9	0,0	5,1	11	Micropiper
Peperomia japonica	25,0	75,0	0,0	2	NA
Peperomia josei	100,0	0,0	0,0	3	Micropiper
Peperomia juniniana	0,0	25,0	75,0	4	Perlucida
Peperomia juruana	100,0	0,0	0,0	3	Leptorhynchum
Peperomia kalimatina	100,0	0,0	0,0	1	Micropiper
Peperomia kamerunana	83,3	0,0	16,7	3	Micropiper
Peperomia kanalensis	0,0	100,0	0,0	1	Micropiper
Peperomia kimnachii	100,0	0,0	0,0	1	Pseudocupula
Peperomia kipahuluensis	92,5	0,0	7,5	2	Micropiper
Peperomia kjellii	0,0	0,0	100,0	2	Peperomia
Peperomia klopfensteinii	0,0	0,0	100,0	1	Tildenia
Peperomia klotzschiana	50,0	0,0	50,0	2	Micropiper
Peperomia klugiana	100,0	0,0	0,0	2	Leptorhynchum
Peperomia kokeana	0,0	0,0	100,0	1	Micropiper
Peperomia kotana	50,0	50,0	0,0	4	NA
Peperomia kraemeri	0,0	100,0	0,0	1	NA
Peperomia kravangensis	100,0	0,0	0,0	1	NA
Peperomia kuntzei	0,0	0,0	100,0	1	Peperomia
Peperomia kusaiensis	100,0	0,0	0,0	3	Micropiper
Peperomia laeteviridis	100,0	0,0	0,0	1	Micropiper
Peperomia laevifolia	55,6	38,9	5,6	6	Micropiper
Peperomia laevilimba	32,5	67,5	0,0	2	Micropiper

Peperomia lagunaensis	66,7	16,7	16,7	2	Micropiper
Peperomia lanaoensis	0,0	100,0	0,0	1	NA
Peperomia lanceolata	16,7	16,7	66,7	6	Micropiper
Peperomia lanceolatopeltata	38,2	47,6	14,3	15	Oxyrhynchum
Peperomia lancifolia	28,6	8,7	62,7	17	Erasmia
Peperomia lanosa	0,0	0,0	100,0	1	Micropiper
Peperomia lanuginosa	0,0	0,0	100,0	1	Micropiper
Peperomia lasierrana	0,0	0,0	100,0	1	NA
Peperomia lasiophylla	100,0	0,0	0,0	1	Micropiper
Peperomia lasiorhachis	100,0	0,0	0,0	1	Micropiper
Peperomia lasiostigma	66,3	21,3	12,5	4	Micropiper
Peperomia latibracteata	100,0	0,0	0,0	1	NA
Peperomia latifolia	50,0	25,0	25,0	2	Micropiper
Peperomia latilimba	0,0	0,0	100,0	1	Multipalmata
Peperomia latimerana	100,0	0,0	0,0	1	Micropiper
Peperomia lauterbachii	100,0	0,0	0,0	1	NA
Peperomia lawrancei	100,0	0,0	0,0	2	Leptorhynchum
Peperomia laxiflora	0,0	0,0	100,0	3	NA
Peperomia ledermannii	100,0	0,0	0,0	1	Micropiper
Peperomia lehmannii	0,0	0,0	100,0	1	Multipalmata
Peperomia lenticularis	100,0	0,0	0,0	3	NA
Peperomia leptophylla	66,7	33,3	0,0	3	Micropiper
Peperomia leptostachya	22,9	65,5	11,5	13	Micropiper
Peperomia leptostachyoides	100,0	0,0	0,0	1	Micropiper
Peperomia leucanthera	50,0	0,0	50,0	2	Micropiper
Peperomia leucorrhachis	0,0	0,0	100,0	1	NA
Peperomia leucostachya	0,0	0,0	100,0	1	Multipalmata
Peperomia liclicensis	0,0	100,0	0,0	2	Fenestratae
Peperomia liebmannii	27,5	60,0	12,5	4	Micropiper
Peperomia liesneri	50,0	25,0	25,0	2	Leptorhynchum
Peperomia lifuana	0,0	100,0	0,0	1	Micropiper
Peperomia lignescens	21,3	40,4	38,3	7	Oxyrhynchum
Peperomia ligustrina	0,0	100,0	0,0	1	NA
Peperomia lilliputiana	0,0	0,0	100,0	1	Tildenia
Peperomia limana	25,0	0,0	75,0	2	NA
Peperomia linaresii	0,0	50,0	50,0	2	Tildenia
Peperomia lindmaniana	0,0	0,0	100,0	1	Oxyrhynchum
Peperomia llewelynii	0,0	0,0	100,0	1	NA
Peperomia loefgrenii	0,0	0,0	100,0	2	NA
Peperomia lonchophylloides	100,0	0,0	0,0	1	Micropiper
Peperomia longepedunculata	0,0	0,0	100,0	1	Multipalmata
Peperomia longibacca	100,0	0,0	0,0	1	Leptorhynchum
Peperomia longipetiolata	0,0	0,0	100,0	1	Leptorhynchum
Peperomia longipila	0,0	0,0	100,0	1	NA
Peperomia longisetosa	0,0	0,0	100,0	1	NA
Peperomia lorentzii	100,0	0,0	0,0	3	Pseudocupula
Peperomia loucoubeana	100,0	0,0	0,0	1	Micropiper

Peperomia loxensis	68,8	12,5	18,8	8	Micropiper
Peperomia luisana	50,0	50,0	0,0	1	Leptorhynchum
Peperomia lyallii	100,0	0,0	0,0	1	Micropiper
Peperomia lyman-smithii	0,0	0,0	100,0	2	Multipalmata
Peperomia macbrideana	0,0	0,0	100,0	1	NA
Peperomia macraeana	52,5	0,0	47,5	2	Pseudocupula
Peperomia macrandra	0,0	100,0	0,0	1	Tildenia
Peperomia macrorhiza	0,0	66,7	33,3	3	Tildenia
Peperomia macrorostrum	100,0	0,0	0,0	2	NA
Peperomia macrostachyos	75,4	6,1	18,5	34	NA
Peperomia macrothyrsa	0,0	0,0	100,0	1	NA
Peperomia macrotricha	0,0	0,0	100,0	2	Multipalmata
Peperomia maculosa	33,3	22,7	44,0	16	Leptorhynchum
Peperomia maestrana	50,0	0,0	50,0	2	Micropiper
Peperomia magnifoliiflora	0,0	0,0	100,0	1	Phyllobryon
Peperomia magnoliifolia	45,6	30,6	23,8	25	Oxyrhynchum
Peperomia maguirei	80,0	20,0	0,0	5	Micropiper
Peperomia maijeri	0,0	50,0	50,0	1	Fenestratae
Peperomia majalis	50,0	0,0	50,0	2	Leptorhynchum
Peperomia mameiana	33,3	29,1	37,6	4	Oxyrhynchum
Peperomia manabina	12,5	0,0	87,5	2	NA
Peperomia manarae	0,0	0,0	100,0	1	Leptorhynchum
Peperomia mandioccana	33,3	50,0	16,7	6	Micropiper
Peperomia mangalbaria	50,0	50,0	0,0	1	NA
Peperomia mantadiana	0,0	0,0	100,0	1	Micropiper
Peperomia mantaroana	0,0	50,0	50,0	1	Fenestratae
Peperomia marahuacensis	0,0	50,0	50,0	2	Multipalmata
Peperomia maransara	0,0	0,0	100,0	1	Leptorhynchum
Peperomia marcapatana	33,3	33,3	33,3	3	Micropiper
Peperomia marchionensis	38,7	38,7	22,7	3	Micropiper
Peperomia marcoana	33,3	66,7	0,0	3	NA
Peperomia margaritifera	0,0	45,0	55,0	2	Micropiper
Peperomia mariannensis	75,0	0,0	25,0	2	NA
Peperomia marivelesana	83,3	16,7	0,0	3	Micropiper
Peperomia marmorata	0,0	0,0	100,0	1	Multipalmata
Peperomia marshalliana	50,0	0,0	50,0	2	Pseudocupula
Peperomia martiana	65,5	17,4	17,1	19	Micropiper
Peperomia masuthoniana	66,7	33,3	0,0	3	NA
Peperomia mathewsiana	0,0	0,0	100,0	2	Micropiper
Peperomia mathieui	0,0	50,0	50,0	1	Fenestratae
Peperomia matlalucaensis	100,0	0,0	0,0	6	Micropiper
Peperomia mauiensis	83,3	16,7	0,0	3	Micropiper
Peperomia maxonii	100,0	0,0	0,0	1	NA
Peperomia maxwellana	0,0	75,0	25,0	2	NA
Peperomia maypurensis	40,0	20,0	40,0	5	NA
Peperomia meeboldii	100,0	0,0	0,0	1	NA
Peperomia megalepis	66,7	0,0	33,3	3	Micropiper

Peperomia megalopoda	0,0	100,0	0,0	2	Micropiper
Peperomia megapotamica	25,0	37,5	37,5	4	Pseudocupula
Peperomia melanokirrocarpa	100,0	0,0	0,0	1	Micropiper
Peperomia melanosticta	50,0	0,0	50,0	1	Micropiper
Peperomia melinii	100,0	0,0	0,0	1	Pseudocupula
Peperomia membranacea	66,7	16,7	16,7	2	Micropiper
Peperomia menkeana	100,0	0,0	0,0	1	Pseudocupula
Peperomia mercedana	33,3	0,0	66,7	3	Micropiper
Peperomia meridana	50,0	0,0	50,0	2	NA
Peperomia merrillii	0,0	100,0	0,0	1	NA
Peperomia mesitasana	0,0	0,0	100,0	1	Hispidula
Peperomia metallica	0,0	0,0	100,0	1	Peperomia
Peperomia metcalfii	0,0	50,0	50,0	1	Leptorhynchum
Peperomia mexicana	2,5	65,0	32,5	4	Tildenia
Peperomia microlepis	50,0	0,0	50,0	2	Micropiper
Peperomia micromamillata	0,0	0,0	100,0	1	Micropiper
Peperomia micromerioides	50,0	0,0	50,0	2	NA
Peperomia microphylla	37,0	9,3	53,8	10	Micropiper
Peperomia microphyllophora	0,0	0,0	100,0	2	NA
Peperomia microstachya	100,0	0,0	0,0	1	Micropiper
Peperomia millei	50,0	0,0	50,0	2	Micropiper
Peperomia mindoroensis	100,0	0,0	0,0	1	Micropiper
Peperomia minensis	100,0	0,0	0,0	3	Pseudocupula
Peperomia minuta	0,0	0,0	100,0	1	Tildenia
Peperomia miqueliana	0,0	0,0	100,0	3	Multipalmata
Peperomia mishuyacana	62,5	0,0	37,5	4	Pseudocupula
Peperomia mitchelioides	0,0	0,0	100,0	1	NA
Peperomia mitoensis	0,0	50,0	50,0	1	Fenestratae
Peperomia mixtifolia	0,0	0,0	100,0	1	NA
Peperomia mocoana	0,0	10,0	90,0	1	Leptorhynchum
Peperomia mocquerysii	100,0	0,0	0,0	1	Micropiper
Peperomia modicilimba	0,0	50,0	50,0	2	Micropiper
Peperomia molleri	26,7	2,5	70,8	6	Micropiper
Peperomia mollis	100,0	0,0	0,0	6	Micropiper
Peperomia mollisoides	62,5	0,0	37,5	4	Micropiper
Peperomia monostachya	0,0	0,0	100,0	1	NA
Peperomia montana	0,0	0,0	100,0	1	NA
Peperomia montecristana	100,0	0,0	0,0	5	Micropiper
Peperomia monticola	0,0	100,0	0,0	1	Tildenia
Peperomia moralesii	0,0	0,0	100,0	1	Tildenia
Peperomia moreliana	100,0	0,0	0,0	1	Micropiper
Peperomia morungavana	50,0	0,0	50,0	1	NA
Peperomia mosenii	100,0	0,0	0,0	1	Micropiper
Peperomia moulmeiniana	100,0	0,0	0,0	3	Micropiper
Peperomia moyobambana	100,0	0,0	0,0	1	NA
Peperomia multifolia	0,0	0,0	100,0	1	Micropiper
Peperomia multiformis	50,0	25,0	25,0	2	Micropiper

Peperomia multisurcula	50,0	50,0	0,0	3	NA
Peperomia muscicola	100,0	0,0	0,0	1	Micropiper
Peperomia muscigaudens	100,0	0,0	0,0	3	Micropiper
Peperomia mutilata	100,0	0,0	0,0	1	Micropiper
Peperomia myrtifolia	11,1	55,6	33,3	9	Micropiper
Peperomia naevifolia	0,0	0,0	100,0	2	Pseudocupula
Peperomia naitasiriensis	100,0	0,0	0,0	3	NA
Peperomia nakaharae	100,0	0,0	0,0	3	NA
Peperomia namosiana	100,0	0,0	0,0	3	NA
Peperomia nandalana	12,5	87,5	0,0	4	Micropiper
Peperomia nandarivatensis	0,0	100,0	0,0	3	Micropiper
Peperomia naranjoana	72,9	22,9	4,2	8	NA
Peperomia naviculifolia	0,0	50,0	50,0	2	Fenestratae
Peperomia neblinana	100,0	0,0	0,0	2	Pleurocarpidium
Peperomia negrosensis	33,3	66,7	0,0	3	Micropiper
Peperomia nequejahuirana	100,0	0,0	0,0	1	Micropiper
Peperomia nicolliae	75,0	25,0	0,0	2	Micropiper
Peperomia nigricans	0,0	0,0	100,0	1	Multipalmata
Peperomia nigro-oculata	100,0	0,0	0,0	2	Pseudocupula
Peperomia nigropunctata	100,0	0,0	0,0	6	Micropiper
Peperomia nigro-ungulata	100,0	0,0	0,0	2	Erasmia
Peperomia nitida	45,8	33,3	20,8	8	Leptorhynchum
Peperomia nivalis	0,0	22,9	77,1	7	Fenestratae
Peperomia nizaitoensis	0,0	50,0	50,0	2	Micropiper
Peperomia nodosa	0,0	0,0	100,0	1	Micropiper
Peperomia non-alata	0,0	0,0	100,0	1	Micropiper
Peperomia nopalana	100,0	0,0	0,0	4	NA
Peperomia nossibeana	0,0	100,0	0,0	1	Micropiper
Peperomia novemnervia	0,0	0,0	100,0	1	Micropiper
Peperomia nudicaulis	100,0	0,0	0,0	1	Micropiper
Peperomia nudifolia	100,0	0,0	0,0	1	Pseudocupula
Peperomia nummularioides	100,0	0,0	0,0	1	Micropiper
Peperomia oahuensis	96,3	0,0	3,8	4	Micropiper
Peperomia obcordata	50,0	0,0	50,0	2	Pseudocupula
Peperomia obcordatifolia	50,0	0,0	50,0	2	Micropiper
Peperomia obex	100,0	0,0	0,0	1	Micropiper
Peperomia oblancifolia	0,0	100,0	0,0	1	Micropiper
Peperomia obliqua	50,0	0,0	50,0	2	Oxyrhynchum
Peperomia obovalifolia	50,0	0,0	50,0	2	NA
Peperomia obovalis	66,7	0,0	33,3	3	Micropiper
Peperomia obovatilimba	41,7	0,0	58,3	6	NA
Peperomia obruenda	0,0	0,0	100,0	1	Oxyrhynchum
Peperomia obscurifolia	58,3	8,3	33,3	6	Micropiper
Peperomia obtusifolia	51,4	16,1	32,5	48	Oxyrhynchum
Peperomia obtusilimba	50,0	50,0	0,0	2	NA
Peperomia occulta	0,0	66,7	33,3	3	Tildenia
Peperomia ocoana	0,0	100,0	0,0	1	Micropiper

Peperomia ocrosensis	0,0	0,0	100,0	1	Tildenia
Peperomia ocumarana	90,0	0,0	10,0	5	Micropiper
Peperomia oerstedii	87,5	0,0	12,5	4	Micropiper
Peperomia olens	0,0	0,0	100,0	1	Micropiper
Peperomia olivacea	56,3	6,3	37,5	8	Micropiper
Peperomia oliveri	29,2	29,2	41,7	4	Micropiper
Peperomia ollantaitambona	0,0	0,0	100,0	1	Micropiper
Peperomia ophistachyera	100,0	0,0	0,0	1	Micropiper
Peperomia orbiculimba	33,3	50,0	16,7	3	NA
Peperomia oreophila	0,0	100,0	0,0	3	Pseudocupula
Peperomia oscarii	100,0	0,0	0,0	2	Leptorhynchum
Peperomia ostolazae	0,0	100,0	0,0	1	NA
Peperomia ouabianae	76,4	23,6	0,0	11	Micropiper
Peperomia ovatopeltata	0,0	50,0	50,0	1	Tildenia
Peperomia oxyphylla	0,0	0,0	100,0	1	Micropiper
Peperomia pachiteana	50,0	0,0	50,0	2	Leptorhynchum
Peperomia pachydermis	50,0	50,0	0,0	2	NA
Peperomia pachyspadix	0,0	50,0	50,0	1	NA
Peperomia pachystachya	0,0	0,0	100,0	2	Peperomia
Peperomia pacificicola	100,0	0,0	0,0	1	Micropiper
Peperomia painteri	0,0	0,0	100,0	1	Tildenia
Peperomia pakipski	100,0	0,0	0,0	3	Micropiper
Peperomia palcana	0,0	0,0	100,0	1	Micropiper
Peperomia pallens	100,0	0,0	0,0	1	Micropiper
Peperomia pallida	36,3	61,3	2,5	4	Micropiper
Peperomia pallidibacca	66,7	0,0	33,3	3	Micropiper
Peperomia palmana	36,6	6,6	56,8	5	Micropiper
Peperomia palmiformis	0,0	0,0	100,0	1	Fenestratae
Peperomia palmiriensis	0,0	0,0	100,0	2	Peperomia
Peperomia pampalcana	0,0	0,0	100,0	1	Pseudocupula
Peperomia pandiana	0,0	0,0	100,0	1	Multipalmata
Peperomia pangerangoana	100,0	0,0	0,0	1	NA
Peperomia papillispica	0,0	50,0	50,0	2	Micropiper
Peperomia papillosa	100,0	0,0	0,0	1	NA
Peperomia paradoxa	50,0	0,0	50,0	1	Peperomia
Peperomia paraguayensis	100,0	0,0	0,0	1	NA
Peperomia paramuna	0,0	0,0	100,0	2	NA
Peperomia parasitica	83,3	16,7	0,0	3	Micropiper
Peperomia parastriata	41,6	41,6	16,9	4	NA
Peperomia parcicilia	100,0	0,0	0,0	1	NA
Peperomia parcifolia	100,0	0,0	0,0	2	Micropiper
Peperomia parcipeltata	100,0	0,0	0,0	2	Leptorhynchum
Peperomia parhamii	100,0	0,0	0,0	3	Micropiper
Peperomia pariensis	100,0	0,0	0,0	2	Micropiper
Peperomia parnassiifolia	66,7	0,0	33,3	3	Multipalmata
Peperomia parva	66,7	0,0	33,3	3	Micropiper
Peperomia parvibacca	100,0	0,0	0,0	1	Micropiper

Peperomia parvicaulis	100,0	0,0	0,0	1	NA
Peperomia parvifolia	0,0	33,3	66,7	3	Tildenia
Peperomia parvilimba	50,0	0,0	50,0	2	Micropiper
Peperomia parvipunctulata	0,0	0,0	100,0	1	Micropiper
Peperomia parvisagittata	0,0	0,0	100,0	1	Tildenia
Peperomia parvulifolia	100,0	0,0	0,0	1	NA
Peperomia pasionana	0,0	50,0	50,0	1	NA
Peperomia patula	61,1	11,1	27,8	3	Micropiper
Peperomia pavoniana	0,0	0,0	100,0	1	NA
Peperomia pearcei	50,0	0,0	50,0	2	Pseudocupula
Peperomia pecuniifolia	72,5	27,5	0,0	4	Pseudocupula
Peperomia pedicellata	25,0	25,0	50,0	2	Tildenia
Peperomia pedunculata	50,0	0,0	50,0	1	Micropiper
Peperomia pellucida	5,7	12,0	82,3	52	Peperomia
Peperomia pellucidoides	0,0	50,0	50,0	2	Peperomia
Peperomia pellucidopunctulata	50,0	33,3	16,7	3	NA
Peperomia peltaphylla	0,0	0,0	100,0	1	Multipalmata
Peperomia peltifolia	0,0	0,0	100,0	1	Leptorhynchum
Peperomia peltigera	0,0	0,0	100,0	2	Multipalmata
Peperomia peltilimba	56,8	22,3	20,9	11	Leptorhynchum
Peperomia peltoidea	55,1	10,8	34,1	7	Leptorhynchum
Peperomia pendulicaulis	100,0	0,0	0,0	2	Micropiper
Peperomia penduliramea	100,0	0,0	0,0	2	Leptorhynchum
Peperomia penicillata	0,0	100,0	0,0	1	Micropiper
Peperomia peploides	100,0	0,0	0,0	1	NA
Peperomia percalvescens	50,0	0,0	50,0	2	Leptorhynchum
Peperomia perciliata	100,0	0,0	0,0	2	Leptorhynchum
Peperomia pereneana	0,0	0,0	100,0	2	Leptorhynchum
Peperomia pereskiifolia	51,5	30,5	18,0	20	Pseudocupula
Peperomia perforata	100,0	0,0	0,0	3	Micropiper
Peperomia perglandulosa	100,0	0,0	0,0	3	Leptorhynchum
Peperomia perlongicaulis	66,7	0,0	33,3	3	NA
Peperomia perlongipedunculata	0,0	0,0	100,0	1	Multipalmata
Peperomia perlongipes	75,0	25,0	0,0	2	Leptorhynchum
Peperomia pernambucensis	66,8	0,0	33,2	17	Oxyrhynchum
Peperomia perodiniana	100,0	0,0	0,0	1	Pseudocupula
Peperomia persucculenta	0,0	0,0	100,0	1	NA
Peperomia persulcata	0,0	0,0	100,0	2	NA
Peperomia pertomentella	33,3	0,0	66,7	3	Leptorhynchum
Peperomia peruviana	0,0	29,4	70,6	8	Tildenia
Peperomia petiolaris	25,0	57,5	17,5	4	Micropiper
Peperomia petiolata	47,8	36,1	16,1	3	Micropiper
Peperomia petraea	0,0	50,0	50,0	2	Micropiper
Peperomia petrophila	31,5	30,4	38,1	13	NA
Peperomia philipsonii	0,0	0,0	100,0	2	NA
Peperomia phyllantha	0,0	50,0	50,0	1	Phyllobryon
Peperomia phyllanthopsis	0,0	0,0	100,0	2	Peperomia

Peperomia physostachya	0,0	50,0	50,0	1	NA
Peperomia pichinchae	50,0	0,0	50,0	2	NA
Peperomia pichisensis	66,7	0,0	33,3	3	Micropiper
Peperomia pilicaulis	99,1	0,0	0,9	9	Micropiper
Peperomia pilifera	0,0	0,0	100,0	1	Micropiper
Peperomia pilipetiolata	100,0	0,0	0,0	2	NA
Peperomia pillahuatana	0,0	0,0	100,0	1	Micropiper
Peperomia pilocarpa	85,0	0,0	15,0	1	NA
Peperomia pilosa	0,0	0,0	100,0	1	NA
Peperomia pilostigma	0,0	100,0	0,0	2	Micropiper
Peperomia pilulifera	100,0	0,0	0,0	1	Micropiper
Peperomia pinedoana	0,0	0,0	100,0	1	Multipalmata
Peperomia pinoi	0,0	0,0	100,0	2	Phyllobryon
Peperomia pitcairnensis	100,0	0,0	0,0	1	Micropiper
Peperomia pitiguayana	100,0	0,0	0,0	1	Micropiper
Peperomia pittieri	66,7	6,7	26,7	5	Micropiper
Peperomia playapampana	0,0	0,0	100,0	1	Micropiper
Peperomia pleiomorpha	66,7	0,0	33,3	3	Micropiper
Peperomia plicatifolia	50,0	0,0	50,0	2	Pleurocarpidium
Peperomia plurispica	0,0	0,0	100,0	1	Micropiper
Peperomia pluvisilvatica	100,0	0,0	0,0	2	Micropiper
Peperomia poasana	53,0	0,0	47,0	5	Erasmia
Peperomia polybotrya	0,0	0,0	100,0	2	NA
Peperomia polycephala	0,0	0,0	100,0	1	Tildenia
Peperomia polymorpha	66,7	0,0	33,3	3	Micropiper
Peperomia polystachyoides	83,3	16,7	0,0	3	Micropiper
Peperomia polystachyos	33,3	50,0	16,7	3	NA
Peperomia polzii	0,0	100,0	0,0	1	Micropiper
Peperomia ponapensis	0,0	100,0	0,0	2	Micropiper
Peperomia pongoana	33,3	0,0	66,7	3	Micropiper
Peperomia pontina	66,7	0,0	33,3	3	Multipalmata
Peperomia porphyridea	100,0	0,0	0,0	2	Leptorhynchum
Peperomia portobellensis	56,3	18,8	25,0	8	Leptorhynchum
Peperomia portoricensis	75,0	0,0	25,0	2	Micropiper
Peperomia portuguesensis	100,0	0,0	0,0	3	NA
Peperomia portulacoides	49,4	44,4	6,3	8	Micropiper
Peperomia potamophila	50,0	50,0	0,0	2	NA
Peperomia ppucu-ppucu	0,0	50,0	50,0	2	Pseudocupula
Peperomia praematura	0,0	0,0	100,0	2	Leptorhynchum
Peperomia praeruptorum	0,0	100,0	0,0	1	Micropiper
Peperomia pringlei	100,0	0,0	0,0	3	Micropiper
Peperomia proctorii	0,0	100,0	0,0	1	Micropiper
Peperomia procumbens	0,0	50,0	50,0	2	Multipalmata
Peperomia productamenta	0,0	100,0	0,0	1	Leptorhynchum
Peperomia profissa	0,0	0,0	100,0	1	Pseudocupula
Peperomia prolifera	0,0	0,0	100,0	1	Micropiper
Peperomia propugnaculi	0,0	100,0	0,0	1	NA

Peperomia prostrata	100,0	0,0	0,0	2	Micropiper
Peperomia pruinosifolia	33,3	33,3	33,3	3	NA
Peperomia pseudoalpina	45,8	14,6	39,6	8	Oxyrhynchum
Peperomia pseudoalternifolia	100,0	0,0	0,0	2	Micropiper
Peperomia pseudoasarifolia	0,0	92,5	7,5	2	NA
Peperomia pseudobcordata	100,0	0,0	0,0	3	NA
Peperomia pseudocasaretti	77,8	11,1	11,1	3	NA
Peperomia pseudocobana	0,0	0,0	100,0	1	Oxyrhynchum
Peperomia pseudodependens	0,0	0,0	100,0	2	Multipalmata
Peperomia pseudoelata	100,0	0,0	0,0	2	NA
Peperomia pseudofurcata	0,0	0,0	100,0	1	Peperomia
Peperomia pseudoglabella	100,0	0,0	0,0	2	Micropiper
Peperomia pseudohirta	100,0	0,0	0,0	2	NA
Peperomia pseudohodgei	33,7	33,2	33,2	2	NA
Peperomia pseudomaculosa	0,0	0,0	100,0	1	NA
Peperomia pseudopereskiifolia	66,6	12,1	21,3	11	Pseudocupula
Peperomia pseudoperuviana	0,0	0,0	100,0	1	Tildenia
Peperomia pseudophyllantha	0,0	0,0	100,0	1	Phyllobryon
Peperomia pseudorhombea	50,0	50,0	0,0	2	NA
Peperomia pseudorhynchophoros	56,7	2,2	41,1	5	NA
Peperomia pseudosalicifolia	50,0	0,0	50,0	2	Micropiper
Peperomia pseudoserratirhachis	0,0	50,0	50,0	2	NA
Peperomia pseudoumbilicata	50,0	0,0	50,0	2	Multipalmata
Peperomia pseudovariegata	0,0	0,0	100,0	1	Leptorhynchum
Peperomia pseudoverruculosa	0,0	0,0	100,0	1	Tildenia
Peperomia psilophylla	100,0	0,0	0,0	1	Leptorhynchum
Peperomia psilostachya	91,7	0,0	8,3	12	Pseudocupula
Peperomia pteroneura	0,0	0,0	100,0	1	Multipalmata
Peperomia puberulescens	50,0	0,0	50,0	2	NA
Peperomia puberulibacca	0,0	0,0	100,0	1	Micropiper
Peperomia puberulicaulis	83,3	0,0	16,7	3	Micropiper
Peperomia puberuliformis	50,0	0,0	50,0	2	NA
Peperomia puberulilimba	33,3	5,0	61,7	3	Leptorhynchum
Peperomia puberulipes	0,0	0,0	100,0	1	Pseudocupula
Peperomia puberulispica	100,0	0,0	0,0	1	Micropiper
Peperomia pubescentinervis	0,0	0,0	100,0	1	Micropiper
Peperomia pubilimba	100,0	0,0	0,0	1	Micropiper
Peperomia pubinervosa	0,0	50,0	50,0	2	Multipalmata
Peperomia pubipeduncula	100,0	0,0	0,0	3	NA
Peperomia pubipetiola	100,0	0,0	0,0	1	Micropiper
Peperomia pubiramea	66,7	0,0	33,3	3	Leptorhynchum
Peperomia pubirhachis	0,0	0,0	100,0	1	Multipalmata
Peperomia puerto-ospinana	100,0	0,0	0,0	2	Pseudocupula
Peperomia pugnicaudex	0,0	0,0	100,0	1	Tildenia
Peperomia pullispica	0,0	100,0	0,0	1	Pseudocupula
Peperomia pululaguana	100,0	0,0	0,0	1	Multipalmata
Peperomia pumila	0,0	0,0	100,0	1	Micropiper

Peperomia punctatilamina	100,0	0,0	0,0	2	Micropiper
Peperomia punctulatissima	100,0	0,0	0,0	1	Micropiper
Peperomia punicea	0,0	25,0	75,0	2	Micropiper
Peperomia purpurea	0,0	0,0	100,0	1	Micropiper
Peperomia purpureonervosa	0,0	50,0	50,0	1	Tildenia
Peperomia purpurinervis	71,4	14,3	14,3	7	Micropiper
Peperomia purpurinodis	100,0	0,0	0,0	4	Micropiper
Peperomia purpurispicata	50,0	0,0	50,0	2	NA
Peperomia pusilla	100,0	0,0	0,0	2	NA
Peperomia putlaensis	0,0	100,0	0,0	1	Tildenia
Peperomia putumayoensis	100,0	0,0	0,0	2	Micropiper
Peperomia pyramidata	31,7	16,7	51,7	6	Erasmia
Peperomia quadrangularis	63,1	29,2	7,7	13	Pseudocupula
Peperomia quadratifolia	70,0	10,0	20,0	5	Pseudocupula
Peperomia quadricoma	50,0	0,0	50,0	2	Pseudocupula
Peperomia quadrifolia	65,9	14,6	19,4	33	Pseudocupula
Peperomia quaerata	66,7	0,0	33,3	3	Pseudocupula
Peperomia quaesita	59,0	0,0	41,0	10	Pseudocupula
Peperomia querocochana	0,0	0,0	100,0	1	Tildenia
Peperomia questionis	0,0	100,0	0,0	1	Tildenia
Peperomia quetzal	25,0	25,0	50,0	2	NA
Peperomia quimiriana	0,0	0,0	100,0	1	Oxyrhynchum
Peperomia quindioensis	100,0	0,0	0,0	1	Micropiper
Peperomia quispicanchiana	0,0	0,0	100,0	1	Pseudocupula
Peperomia racemifolia	0,0	0,0	100,0	1	Micropiper
Peperomia radiatinervosa	0,0	0,0	100,0	1	Tildenia
Peperomia radicosa	100,0	0,0	0,0	3	NA
Peperomia ramboi	0,0	0,0	100,0	2	Micropiper
Peperomia ranongensis	100,0	0,0	0,0	1	NA
Peperomia rapensis	41,7	16,7	41,7	2	NA
Peperomia ratticaudata	75,0	25,0	0,0	2	Micropiper
Peperomia rauniensis	100,0	0,0	0,0	1	NA
Peperomia rechingerae	0,0	0,0	100,0	1	Micropiper
Peperomia reflexa	92,9	7,1	0,0	14	Pseudocupula
Peperomia regelii	50,0	50,0	0,0	2	NA
Peperomia reineckei	50,0	0,0	50,0	1	Micropiper
Peperomia remyi	33,3	16,7	50,0	3	Micropiper
Peperomia renifolia	0,0	0,0	100,0	2	NA
Peperomia renzopalmae	0,0	0,0	100,0	1	Panicularia
Peperomia reptans	41,7	41,7	16,7	2	Leptorhynchum
Peperomia reptilis	62,5	0,0	37,5	4	Peperomia
Peperomia reticulata	100,0	0,0	0,0	1	Micropiper
Peperomia retivenulosa	33,3	0,0	66,7	3	Micropiper
Peperomia retropuberula	100,0	0,0	0,0	1	Pseudocupula
Peperomia retusa	60,3	6,4	33,3	13	Micropiper
Peperomia rhexiifolia	75,6	10,3	14,1	13	Pseudocupula
Peperomia rhodophylla	0,0	50,0	50,0	1	Micropiper

Peperomia rhombea	80,2	11,1	8,7	21	Pseudocupula
Peperomia rhombeifolia	66,7	0,0	33,3	3	Pseudocupula
Peperomia rhombeo-elliptica	66,7	0,0	33,3	3	Pseudocupula
Peperomia rhombiformis	0,0	0,0	100,0	1	Micropiper
Peperomia rhombilimba	0,0	0,0	100,0	1	Micropiper
Peperomia rhomboidea	100,0	0,0	0,0	1	Micropiper
Peperomia ricardofernandezii	0,0	0,0	100,0	1	Panicularia
Peperomia ricaurtensis	100,0	0,0	0,0	2	Micropiper
Peperomia richardsonii	100,0	0,0	0,0	2	Micropiper
Peperomia ridleyi	100,0	0,0	0,0	1	NA
Peperomia riedeliana	100,0	0,0	0,0	2	NA
Peperomia rigida	100,0	0,0	0,0	1	NA
Peperomia rioblancoana	100,0	0,0	0,0	2	NA
Peperomia riocaliensis	0,0	0,0	100,0	2	NA
Peperomia riosaniensis	0,0	50,0	50,0	1	Fenestratae
Peperomia riparia	100,0	0,0	0,0	3	Pseudocupula
Peperomia ripicola	16,7	16,7	66,7	3	NA
Peperomia rivulamans	0,0	100,0	0,0	2	NA
Peperomia rizzinii	50,0	50,0	0,0	2	NA
Peperomia robleana	0,0	0,0	100,0	1	Pseudocupula
Peperomia robusta	15,0	0,0	85,0	1	NA
Peperomia robustior	100,0	0,0	0,0	3	Micropiper
Peperomia rockii	0,0	25,0	75,0	2	Micropiper
Peperomia rosea	66,7	0,0	33,3	3	Pseudocupula
Peperomia roseopetiolata	0,0	100,0	0,0	2	NA
Peperomia rossii	100,0	0,0	0,0	1	Micropiper
Peperomia rostulatiformis	0,0	100,0	0,0	2	Multipalmata
Peperomia rotumaensis	0,0	0,0	100,0	1	NA
Peperomia rotundata	33,3	7,9	58,8	19	Micropiper
Peperomia rotundifolia	80,1	8,2	11,6	60	Micropiper
Peperomia rotundilimba	100,0	0,0	0,0	1	Micropiper
Peperomia rubea	0,0	0,0	100,0	2	Micropiper
Peperomia rubens	100,0	0,0	0,0	1	Pseudocupula
Peperomia rubescens	50,0	0,0	50,0	2	Pseudocupula
Peperomia rubramenta	0,0	0,0	100,0	1	Multipalmata
Peperomia rubricaulis	38,5	50,0	11,5	13	NA
Peperomia rubrifolia	0,0	0,0	100,0	1	Multipalmata
Peperomia rubrimaculata	100,0	0,0	0,0	1	Micropiper
Peperomia rubrivenosa	40,0	60,0	0,0	5	NA
Peperomia rubropunctulata	50,0	0,0	50,0	2	Micropiper
Peperomia rufescens	0,0	0,0	100,0	1	Multipalmata
Peperomia rufescentifolia	0,0	0,0	100,0	1	Micropiper
Peperomia rufispica	100,0	0,0	0,0	3	Pseudocupula
Peperomia rugatifolia	66,7	0,0	33,3	3	Pseudocupula
Peperomia rugosa	100,0	0,0	0,0	1	Multipalmata
Peperomia rupicola	0,0	50,0	50,0	2	Multipalmata
Peperomia rupigaudens	36,7	63,3	0,0	3	Pseudocupula

Peperomia rupiseda	0,0	0,0	100,0	1	Tildenia
Peperomia rurrenabaqueana	100,0	0,0	0,0	2	Micropiper
Peperomia rusbyi	50,0	0,0	50,0	2	Micropiper
Peperomia ruscifolia	100,0	0,0	0,0	1	Micropiper
Peperomia sabaletasana	100,0	0,0	0,0	1	Leptorhynchum
Peperomia sachatzinzumba	42,8	0,0	57,2	1	NA
Peperomia sagasteguii	33,3	33,3	33,4	1	NA
Peperomia sagittata	100,0	0,0	0,0	2	Leptorhynchum
Peperomia saintpauliella	11,3	44,3	44,3	3	Multipalmata
Peperomia salaminana	0,0	0,0	100,0	1	Perlucida
Peperomia salangonis	0,0	0,0	100,0	1	Multipalmata
Peperomia salicifolia	100,0	0,0	0,0	1	Leptorhynchum
Peperomia saligna	46,2	1,2	52,6	13	Micropiper
Peperomia salmonicolor	66,7	0,0	33,3	3	Pseudocupula
Peperomia samainiae	0,0	50,0	50,0	1	Fenestratae
Peperomia sanblasensis	100,0	0,0	0,0	2	NA
Peperomia san-buenaventurana	0,0	0,0	100,0	1	NA
Peperomia san-carlosiana	19,8	32,6	47,6	9	NA
Peperomia sandemanii	0,0	0,0	100,0	2	Micropiper
Peperomia sandwicensis	100,0	0,0	0,0	1	Micropiper
Peperomia san-felipensis	100,0	0,0	0,0	3	NA
Peperomia sangabanensis	0,0	0,0	100,0	1	Micropiper
Peperomia san-joseana	69,4	5,9	24,7	13	NA
Peperomia sanquininiana	50,0	0,0	50,0	2	Micropiper
Peperomia san-roqueana	50,0	0,0	50,0	2	NA
Peperomia sansalvadorana	100,0	0,0	0,0	1	Micropiper
Peperomia santa-elisae	0,0	50,0	50,0	3	NA
Peperomia santa-helenea	71,3	25,0	3,8	4	NA
Peperomia santanderana	0,0	50,0	50,0	1	NA
Peperomia santiagoana	50,0	0,0	50,0	3	Leptorhynchum
Peperomia sarasinii	0,0	100,0	0,0	1	Micropiper
Peperomia saxicola	0,0	50,0	50,0	2	Micropiper
Peperomia scabiosa	66,7	0,0	33,3	3	Pseudocupula
Peperomia schenkiana	100,0	0,0	0,0	1	Leptorhynchum
Peperomia schiedei	100,0	0,0	0,0	1	Leptorhynchum
Peperomia schizandra	0,0	50,0	50,0	1	Tildenia
Peperomia schmidtii	0,0	100,0	0,0	1	Micropiper
Peperomia schultzei	50,0	0,0	50,0	2	Pseudocupula
Peperomia schunkeana	0,0	0,0	100,0	1	Oxyrhynchum
Peperomia schwackei	100,0	0,0	0,0	3	Leptorhynchum
Peperomia sclerophylla	0,0	0,0	100,0	1	Pseudocupula
Peperomia scopulorum	0,0	100,0	0,0	1	Oxyrhynchum
Peperomia scutaleifolia	66,7	0,0	33,3	3	Micropiper
Peperomia scutellariifolia	0,0	0,0	100,0	2	Multipalmata
Peperomia scutellifolia	0,0	50,0	50,0	1	Tildenia
Peperomia scutifolia	50,0	0,0	50,0	3	NA
Peperomia scutilimba	0,0	0,0	100,0	1	Multipalmata

Peperomia secunda	0,0	0,0	100,0	1	Panicularia
Peperomia seemanniana	75,0	0,0	25,0	4	NA
Peperomia segregata	1,7	0,0	98,3	3	NA
Peperomia seibertii	50,0	0,0	50,0	2	Micropiper
Peperomia selenophylla	0,0	50,0	50,0	1	Fenestratae
Peperomia seleri	0,0	25,0	75,0	2	Tildenia
Peperomia semimetralis	0,0	0,0	100,0	1	Micropiper
Peperomia semipuberula	50,0	0,0	50,0	2	Micropiper
Peperomia seposita	0,0	0,0	100,0	1	Micropiper
Peperomia septemnervis	69,0	17,6	13,5	4	Pseudocupula
Peperomia septentrionalis	0,0	100,0	0,0	1	Oxyrhynchum
Peperomia serpens	60,3	15,5	24,1	29	Leptorhynchum
Peperomia serpentarioides	0,0	0,0	100,0	2	Multipalmata
Peperomia serratirhachis	0,0	50,0	50,0	2	Hispidula
Peperomia sessilifolia	24,7	24,7	50,6	3	NA
Peperomia sierpeana	0,0	100,0	0,0	2	NA
Peperomia silvarum	100,0	0,0	0,0	1	Micropiper
Peperomia silvicola	100,0	0,0	0,0	1	Micropiper
Peperomia silvivaga	100,0	0,0	0,0	10	Micropiper
Peperomia simplex	0,0	50,0	50,0	4	Micropiper
Peperomia simulans	0,0	0,0	100,0	2	Peperomia
Peperomia simuliformis	0,0	0,0	100,0	1	NA
Peperomia sincorana	0,0	100,0	0,0	2	Micropiper
Peperomia sirindhorniana	0,0	100,0	0,0	2	NA
Peperomia sirupayana	100,0	0,0	0,0	1	Leptorhynchum
Peperomia skottsbergii	0,0	33,3	66,7	3	Micropiper
Peperomia smithiana	100,0	0,0	0,0	1	Micropiper
Peperomia smithii	66,7	0,0	33,3	3	Pseudocupula
Peperomia sneidernii	61,1	0,0	38,9	3	Micropiper
Peperomia societatis	50,0	50,0	0,0	4	Micropiper
Peperomia socorronis	100,0	0,0	0,0	2	Micropiper
Peperomia sodiroi	0,0	0,0	100,0	2	Multipalmata
Peperomia soukupii	50,0	0,0	50,0	2	Pseudocupula
Peperomia spathophylla	50,0	0,0	50,0	2	NA
Peperomia spathulifolia	0,0	0,0	100,0	1	Oxyrhynchum
Peperomia sphaerostachya	0,0	0,0	100,0	1	NA
Peperomia spiculata	0,0	0,0	100,0	1	Micropiper
Peperomia spiritus-sancti	0,0	100,0	0,0	3	NA
Peperomia spruceana	0,0	0,0	100,0	3	Multipalmata
Peperomia sprucei	25,0	25,0	50,0	2	Multipalmata
Peperomia steinbachii	0,0	100,0	0,0	1	Micropiper
Peperomia stelechophila	47,4	1,7	50,9	3	Leptorhynchum
Peperomia stellata	50,0	25,0	25,0	2	NA
Peperomia stenostachya	0,0	0,0	100,0	1	NA
Peperomia stevensii	100,0	0,0	0,0	1	NA
Peperomia steyermarkii	0,0	100,0	0,0	2	NA
Peperomia stilifera	0,0	0,0	100,0	3	Phyllobryon

Peperomia stipitifolia	50,0	0,0	50,0	2	NA
Peperomia stolonifera	50,0	0,0	50,0	2	Multipalmata
Peperomia strawii	0,0	0,0	100,0	2	Fenestratae
Peperomia striata	43,0	2,0	55,0	17	Leptorhynchum
Peperomia stroemfeltii	83,3	16,7	0,0	3	Micropiper
Peperomia stuebelii	0,0	50,0	50,0	2	Pseudocupula
Peperomia subalata	0,0	0,0	100,0	1	NA
Peperomia subandina	100,0	0,0	0,0	2	Pseudocupula
Peperomia subblanda	75,0	0,0	25,0	4	Micropiper
Peperomia subcalvescens	0,0	0,0	100,0	1	Micropiper
Peperomia subelongata	100,0	0,0	0,0	1	Leptorhynchum
Peperomia subemarginata	100,0	0,0	0,0	3	NA
Peperomia subflaccida	0,0	0,0	100,0	1	NA
Peperomia subpallescens	100,0	0,0	0,0	1	Micropiper
Peperomia subpetiolata	0,0	0,0	100,0	1	NA
Peperomia subpilosa	100,0	0,0	0,0	3	Leptorhynchum
Peperomia subretusa	100,0	0,0	0,0	3	Pseudocupula
Peperomia subroseispica	61,7	38,3	0,0	3	Micropiper
Peperomia subrotundifolia	50,0	25,0	25,0	2	NA
Peperomia subrubescens	16,7	16,7	66,7	3	Multipalmata
Peperomia subrubricaulis	50,0	20,0	30,0	5	Micropiper
Peperomia subrubrispica	0,0	100,0	0,0	4	NA
Peperomia subsericata	100,0	0,0	0,0	3	Micropiper
Peperomia subsetifolia	100,0	0,0	0,0	1	Multipalmata
Peperomia subspathulata	0,0	0,0	100,0	2	Pseudocupula
Peperomia subternifolia	62,5	12,5	25,0	4	Pseudocupula
Peperomia subvillicaulis	50,0	0,0	50,0	2	Micropiper
Peperomia succulenta	70,5	5,1	24,4	13	Micropiper
Peperomia suchitanensis	50,0	16,7	33,3	3	NA
Peperomia sucumbiosensis	100,0	0,0	0,0	1	Leptorhynchum
Peperomia sulbahiensis	0,0	100,0	0,0	3	NA
Peperomia sulcata	33,3	33,3	33,3	3	Multipalmata
Peperomia sumidoriana	100,0	0,0	0,0	3	NA
Peperomia suratana	80,0	7,5	12,5	4	Micropiper
Peperomia suspensa	100,0	0,0	0,0	1	Pseudocupula
Peperomia swartziana	50,0	5,6	44,5	6	Micropiper
Peperomia sylvatica	0,0	0,0	100,0	1	Micropiper
Peperomia sylvestris	50,0	0,0	50,0	2	NA
Peperomia symmankii	0,0	50,0	50,0	1	Panicularia
Peperomia sympodialis	75,0	0,0	25,0	2	Leptorhynchum
Peperomia syringifolia	47,1	26,5	26,5	8	Multipalmata
Peperomia tablahuasiana	0,0	0,0	100,0	1	Micropiper
Peperomia talinifolia	33,5	7,7	58,8	13	Oxyrhynchum
Peperomia tamayoi	0,0	66,7	33,3	3	Multipalmata
Peperomia tambitoensis	0,0	0,0	100,0	1	Multipalmata
Peperomia tamboana	0,0	100,0	0,0	1	Multipalmata
Peperomia tancitaroana	0,0	50,0	50,0	1	NA

Peperomia tarapotana	0,0	0,0	100,0	1	NA
Peperomia tejana	95,0	0,0	5,0	3	Micropiper
Peperomia tenae	0,0	0,0	100,0	1	Multipalmata
Peperomia tenella	64,2	21,6	14,2	27	Pleurocarpidium
Peperomia tenelliformis	63,7	6,7	29,7	5	Pleurocarpidium
Peperomia tenerrima	100,0	0,0	0,0	7	Pseudocupula
Peperomia tenuicaulis	100,0	0,0	0,0	2	Micropiper
Peperomia tenuifolia	25,0	25,0	50,0	2	Oxyrhynchum
Peperomia tenuilimba	33,3	0,0	66,7	3	Peperomia
Peperomia tenuimarginata	0,0	0,0	100,0	1	Leptorhynchum
Peperomia tenuipeduncula	50,0	50,0	0,0	2	Pseudocupula
Peperomia tenuipes	54,8	4,8	40,5	7	Pleurocarpidium
Peperomia tenuiramea	100,0	0,0	0,0	3	NA
Peperomia tenuissima	100,0	0,0	0,0	1	Micropiper
Peperomia tepoztecoana	0,0	100,0	0,0	1	Tildenia
Peperomia tequendamana	30,6	13,9	55,6	6	Micropiper
Peperomia terebinthina	100,0	0,0	0,0	2	Micropiper
Peperomia teresitensis	0,0	0,0	100,0	1	NA
Peperomia ternata	38,1	0,0	61,9	6	Oxyrhynchum
Peperomia terraegaudens	0,0	0,0	100,0	1	Leptorhynchum
Peperomia tetragona	57,1	7,1	35,7	7	Pseudocupula
Peperomia tetraphylla	75,2	15,2	9,7	64	Pseudocupula
Peperomia tetraquetra	63,3	0,0	36,7	6	NA
Peperomia theodori	100,0	0,0	0,0	3	Pseudocupula
Peperomia thollonii	0,0	100,0	0,0	1	NA
Peperomia thomeana	41,7	16,7	41,7	2	Micropiper
Peperomia thomsonii	100,0	0,0	0,0	1	NA
Peperomia thorelii	100,0	0,0	0,0	1	Micropiper
Peperomia ticunhuayana	0,0	0,0	100,0	1	Hispidula
Peperomia tillettii	0,0	0,0	100,0	1	Leptorhynchum
Peperomia timbuchiana	0,0	0,0	100,0	1	Leptorhynchum
Peperomia tlapacoyoensis	75,0	25,0	0,0	2	Leptorhynchum
Peperomia toledoana	0,0	83,3	16,7	3	Tildenia
Peperomia tolimensis	50,0	0,0	50,0	2	Pseudocupula
Peperomia tomentella	0,0	0,0	100,0	1	Multipalmata
Peperomia tomentosa	100,0	0,0	0,0	1	NA
Peperomia tominana	66,7	33,3	0,0	3	Pseudocupula
Peperomia tonduzii	100,0	0,0	0,0	3	Pleurocarpidium
Peperomia tooviiana	95,0	2,5	2,5	3	Micropiper
Peperomia topoensis	100,0	0,0	0,0	2	Leptorhynchum
Peperomia toroi	100,0	0,0	0,0	1	Micropiper
Peperomia tovariana	72,2	16,7	11,1	9	Pleurocarpidium
Peperomia transparens	0,0	100,0	0,0	1	NA
Peperomia trianae	66,7	0,0	33,3	6	Oxyrhynchum
Peperomia trichobracteata	100,0	0,0	0,0	3	NA
Peperomia trichocarpa	100,0	0,0	0,0	3	NA
Peperomia trichomanoides	0,0	100,0	0,0	3	Multipalmata

Peperomia trichophylla	100,0	0,0	0,0	1	Micropiper
Peperomia trichopodus	50,0	50,0	0,0	1	NA
Peperomia trichopus	100,0	0,0	0,0	8	Pseudocupula
Peperomia tricolor	77,8	11,1	11,1	3	Micropiper
Peperomia trifolia	80,0	0,0	20,0	5	Pseudocupula
Peperomia trinervis	78,8	9,1	12,1	11	Micropiper
Peperomia trinervula	37,7	14,6	47,7	8	Micropiper
Peperomia trineura	53,0	42,6	4,4	5	Pseudocupula
Peperomia trineuroides	60,8	32,3	6,9	7	Pseudocupula
Peperomia triplinervis	0,0	0,0	100,0	1	NA
Peperomia tristachya	0,0	0,0	100,0	1	NA
Peperomia trollii	0,0	40,0	60,0	2	Fenestratae
Peperomia tropeoloides	75,0	0,0	25,0	2	Multipalmata
Peperomia trujilloi	100,0	0,0	0,0	2	NA
Peperomia trukensis	0,0	50,0	50,0	1	Micropiper
Peperomia trullifolia	0,0	0,0	100,0	1	Micropiper
Peperomia truncicola	100,0	0,0	0,0	1	Micropiper
Peperomia truncigaudens	100,0	0,0	0,0	2	Micropiper
Peperomia trunciseda	100,0	0,0	0,0	3	Oxyrhynchum
Peperomia truncivaga	100,0	0,0	0,0	2	Micropiper
Peperomia tsakiana	97,0	1,5	1,5	5	NA
Peperomia tubericordata	0,0	100,0	0,0	1	NA
Peperomia tuerckheimii	23,3	43,5	33,3	5	Oxyrhynchum
Peperomia tuisana	55,4	34,2	10,4	8	Micropiper
Peperomia tungurahuae	75,0	0,0	25,0	2	Micropiper
Peperomia turbinata	41,5	17,0	41,5	2	Oxyrhynchum
Peperomia turboensis	0,0	0,0	100,0	3	Perlucida
Peperomia tutensis	100,0	0,0	0,0	2	NA
Peperomia tutuilana	0,0	100,0	0,0	1	Micropiper
Peperomia tutunendoana	66,7	0,0	33,3	3	Leptorhynchum
Peperomia uaupesensis	90,0	10,0	0,0	5	NA
Peperomia ubate-susanensis	0,0	0,0	100,0	1	NA
Peperomia udimontana	100,0	0,0	0,0	1	Erasmia
Peperomia udisilvestris	100,0	0,0	0,0	1	NA
Peperomia umbilicata	0,0	50,0	50,0	3	Tildenia
Peperomia umbrigaudens	66,7	0,0	33,3	3	Multipalmata
Peperomia umbrosa	0,0	0,0	100,0	1	Tildenia
Peperomia uncatispica	0,0	0,0	100,0	1	Erasmia
Peperomia undeninervia	50,0	0,0	50,0	2	Micropiper
Peperomia unduavina	100,0	0,0	0,0	1	Micropiper
Peperomia unifoliata	33,3	66,7	0,0	3	Tildenia
Peperomia unispicata	100,0	0,0	0,0	2	NA
Peperomia urbanii	0,0	50,0	50,0	1	NA
Peperomia urocarpa	44,6	28,0	27,5	40	Leptorhynchum
Peperomia urocarpoides	66,7	0,0	33,3	3	Leptorhynchum
Peperomia ursina	66,7	33,3	0,0	3	Micropiper
Peperomia urvilleana	46,0	43,5	10,4	8	Micropiper

Peperomia valdezii	100,0	0,0	0,0	2	NA
Peperomia valladolidana	0,0	0,0	100,0	1	Multipalmata
Peperomia vallensis	66,7	0,0	33,3	3	Micropiper
Peperomia valliculae	66,7	0,0	33,3	3	Micropiper
Peperomia vana	50,0	0,0	50,0	2	Micropiper
Peperomia vareschii	0,0	0,0	100,0	2	NA
Peperomia variculata	66,7	0,0	33,3	3	Micropiper
Peperomia variifolia	0,0	0,0	100,0	1	Micropiper
Peperomia variilimba	100,0	0,0	0,0	1	NA
Peperomia vazquezii	0,0	66,7	33,3	3	NA
Peperomia vellarimalica	0,0	0,0	100,0	1	NA
Peperomia velloziana	38,8	38,8	22,5	3	Micropiper
Peperomia velutina	75,0	0,0	25,0	4	Micropiper
Peperomia venabulifolia	62,5	0,0	37,5	4	NA
Peperomia veneciana	100,0	0,0	0,0	2	NA
Peperomia venezueliana	27,8	16,1	56,1	3	Leptorhynchum
Peperomia venosa	0,0	0,0	100,0	1	Multipalmata
Peperomia ventenatii	100,0	0,0	0,0	1	Micropiper
Peperomia venticosicarpa	0,0	0,0	100,0	1	NA
Peperomia venulosa	62,5	0,0	37,5	2	NA
Peperomia venusta	0,0	0,0	100,0	2	NA
Peperomia veraguana	100,0	0,0	0,0	2	NA
Peperomia verediana	0,0	0,0	100,0	1	Multipalmata
Peperomia vernouana	100,0	0,0	0,0	1	Micropiper
Peperomia verruculosa	0,0	75,0	25,0	2	Tildenia
Peperomia verschaffeltii	0,0	0,0	100,0	1	Multipalmata
Peperomia versicolor	78,6	21,4	0,0	7	Micropiper
Peperomia verticillata	35,0	32,5	32,5	3	Micropiper
Peperomia verticillatispica	91,7	0,0	8,3	3	NA
Peperomia vestita	0,0	0,0	100,0	1	Leptorhynchum
Peperomia vidaliana	0,0	0,0	100,0	1	NA
Peperomia villarrealii	65,0	11,7	23,3	6	Micropiper
Peperomia villicaulis	0,0	25,0	75,0	2	Micropiper
Peperomia villosa	0,0	0,0	100,0	1	Multipalmata
Peperomia vinasiana	83,3	16,7	0,0	3	Leptorhynchum
Peperomia vincentiana	100,0	0,0	0,0	2	Micropiper
Peperomia violacea	0,0	0,0	100,0	1	Micropiper
Peperomia viracochana	0,0	0,0	100,0	1	Micropiper
Peperomia vitiana	100,0	0,0	0,0	2	Micropiper
Peperomia vitilevuensis	100,0	0,0	0,0	3	Micropiper
Peperomia vivipara	33,3	33,3	33,4	1	NA
Peperomia vueltasana	100,0	0,0	0,0	2	NA
Peperomia vulcanica	52,5	47,5	0,0	2	Micropiper
Peperomia warmingii	0,0	75,0	25,0	2	Micropiper
Peperomia weberbaueri	0,0	50,0	50,0	3	Fenestratae
Peperomia wernerrauhii	0,0	100,0	0,0	1	Tildenia
Peperomia wheeleri	0,0	100,0	0,0	2	Micropiper

Peperomia wibomii	100,0	0,0	0,0	1	Pseudocupula
Peperomia williamsii	100,0	0,0	0,0	1	NA
Peperomia wolfgang-krahnii	0,0	0,0	100,0	1	Fenestratae
Peperomia woytkowskii	0,0	0,0	100,0	1	Leptorhynchum
Peperomia wrayi	100,0	0,0	0,0	3	NA
Peperomia xalana	100,0	0,0	0,0	3	NA
Peperomia yabucoana	0,0	100,0	0,0	2	Micropiper
Peperomia yanacachiana	0,0	0,0	100,0	1	Micropiper
Peperomia yananoensis	0,0	0,0	100,0	1	NA
Peperomia yapasana	50,0	0,0	50,0	2	Leptorhynchum
Peperomia yatuensis	50,0	50,0	0,0	2	Leptorhynchum
Peperomia yeracuiana	100,0	0,0	0,0	2	Leptorhynchum
Peperomia yungasana	0,0	50,0	50,0	2	Micropiper
Peperomia yutajensis	41,7	16,7	41,7	2	Micropiper
Peperomia zarzalana	0,0	50,0	50,0	3	Micropiper
Peperomia zipaquirana	0,0	16,7	83,3	2	Micropiper
Peperomia zongolicana	0,0	100,0	0,0	1	NA
