# Supplementary material for: “On a tree”, “terrestrial”, or “on the rocks”? Habit diversity in the megadiverse genus Peperomia
Source: Plant Biol (Stuttg). 2026 May 13;28(5):1373–84. doi: 10.1111/plb.70214 (PMC13358651; doi:10.1111/plb.70214)
Supplement: Supplementary file 4 — Table S4. Database showing data curation process detailed in the method section. Full references can be found in Table S10. [file PLB-28-1373-s003.pdf]

Table S4: Database showing data curation process detailed in the method section. Full references can be found in Table S10.

Taxon ID	Name in publication	Reference	Curation process
	Peperomia adenocarpa	310	Peperomia adenocarpa C.DC.
wfo-0000478171	*Peperomia aggravescens*	328	move to Peperomia heterophylla Miq.
wfo-0001245266.	*Peperomia aguilae*	373	move to Peperomia gutierrezana Yunck.
wfo-0000478160	*Peperomia albostriata*	198	move to Peperomia tetragona Ruiz & Pav.
wfo-0000478160	*Peperomia albostriata*	375	move to Peperomia tetragona Ruiz & Pav.
wfo-0000478183	*Peperomia allorgeana*	198	move to Peperomia hirtella Miq.
wfo-0000478291	*Peperomia anomala*	150	move to Peperomia rotundata
wfo-0000478283	*Peperomia apoana*	198	move to Peperomia laevifolia (Blume) Miq.
wfo-0000478304	*Peperomia aspergillus*	198	move to Peperomia saligna Kunth
wfo-0000478306	*Peperomia asperulata*	29	move to Peperomia cookiana C.DC.
	Peperomia astrostigma	73	move to Peperomia obovatilimba C.DC.
wfo-0000478303	*Peperomia astrostigma*	198	move to Peperomia obovatilimba C.DC.
wfo-0000478312	*Peperomia atocongona*	29	move to Peperomia inaequalifolia Ruiz & Pav.
wfo-0000478280	*Peperomia auberyana*	198	move to Peperomia myrtifolia (Vahl) A.Dietr.
wfo-0001093836	*Peperomia ayacuchana*	29	move to Peperomia serpens (Sw.) Loudon
wfo-0000478240	*Peperomia bangroana*	1	corrected from bangrooana to bangroana
	Peperomia bangroana	255	corrected from bangrooana to bangroana
wfo-0001093032	*Peperomia barbinodis*	57	move to Peperomia lanceolata C.DC.
	Peperomia barbinodis	37	move to Peperomia lanceolata C.DC.
wfo-0000478250	*Peperomia bartlettii*	373	move to Peperomia rotundifolia (L.) Kunth
wfo-0000478264	*Peperomia basellifolia*	112	move to Peperomia acuminata Ruiz & Pav.
wfo-0000478263	*Peperomia bethaniana*	373	move to Peperomia angularis C.DC.
wfo-0000478260	*Peperomia boomii*	339	move to Peperomia lanceolatapeltata C.DC.
wfo-0000478046	*Peperomia boraborensis*	198	move to Peperomia societatis Moore
wfo-0000478043	*Peperomia brachyiula*	29	move to Peperomia galioides Kunth
wfo-0000478070	*Peperomia brevicaulis*	29	move to Peperomia curtipes Trel.
wfo-0000478073	*Peperomia butaguensis*	1	move to Peperomia fernandopoiana C.DC.
	Peperomia butaguensis	16	move to Peperomia fernandopoiana C.DC.
wfo-0000478062	*Peperomia caespitiformans*		move to Peperomia trifolia (L.) A.Dietr.
wfo-0000478037	*Peperomia calimana*	373	move to Peperomia gleicheniiformis Trel.
wfo-0001091788.	*Peperomia calvifolia*	255	move to Peperomia sessilifolia Hook.
wfo-0000478022	*Peperomia calyculata*	198	move to Peperomia silvivaga C.DC.
wfo-0000478026	*Peperomia campylotropa*	328	move to Peperomia bracteata A.W. Hill
wfo-0000478025	*Peperomia candollei*		move to Peperomia leptostachya Hook. & Arn.
wfo-0000478024	*Peperomia cangrejalana*	408	move to Peperomia obtusifolia (L.) A.Dietr.
wfo-0000478015	*Peperomia carapasana*	29	move to Peperomia rhexiifolia C.DC.
	Peperomia carapasana	373	move to Peperomia rhexiifolia C.DC.
wfo-0000478017	*Peperomia carlosiana*		move to Peperomia lignescens C.DC.
wfo-0000478014	*Peperomia carnosa*		move to Peperomia lasiostigma
wfo-0000478032	*Peperomia carthaginensis*		move to Peperomia lignescens C.DC.
wfo-0000478034	*Peperomia casitana*	393	move to Peperomia adscendens C.DC.
wfo-0000478030	*Peperomia castanoensis*	373	move to Peperomia succulenta C.DC.
wfo-0000490340	*Peperomia cataractigaudens*		move to Peperomia hirtella Miq. (corrected from cataractaegaudens to cataractigaudens)
wfo-0000478035	*Peperomia cataractigaudens*	332	move to Peperomia hirtella Miq.
wfo-0000478036	*Peperomia cataratasensis*	326	move to Peperomia urocarpoides C.DC.
wfo-0000482351	*Peperomia cattii*		move to Peperomia glabella (Sw.) A.Dietr.
wfo-0000482392	*Peperomia caulibarbis*		move to Peperomia glabella (Sw.) A.Dietr.
wfo-0000482398	*Peperomia ceylanica*		move to Peperomia heyneana Miq.
wfo-0000482388	*Peperomia chambesyana*		move to Peperomia lanceolatopeltata Trel.
wfo-0000490380	*Peperomia chicbulana*		move to Peperomia granulosa Beurl.
wfo-0000482391	*Peperomia chillonensis*	29	move to Peperomia galioides Kunth
	Peperomia chillonensis	178	move to Peperomia galioides Kunth
wfo-0000482387	*Peperomia chimantana*	414	move to Peperomia jamesoniana C.DC.
wfo-0000482389	*Peperomia chlorostachya*		move to Peperomia puberulilimba C.DC
wfo-0001093038	*Peperomia chrysleri*	57	move to Peperomia obscurifolia C.DC.
wfo-0000482411	*Peperomia chrysocarpa*		move to Peperomia sanjoseana C.DC.
wfo-0001249098	*Peperomia chrysolepida*	110	move to Peperomia tetraphylla Hook. & Arn.
wfo-0000482414	*Peperomia chucanebana*	176	move to Peperomia conocarpa Trel.
	Peperomia chucanebana	325	move to Peperomia conocarpa Trel.
wfo-0000482410	*Peperomia ciliata*		move to Peperomia blanda (Jacq.) Kunth
wfo-0000482416	*Peperomia cililimba*		move to Peperomia samoensis
wfo-0000482404	*Peperomia circumscissa*		move to Peperomia macrostachyos (Vahl) A.Dietr.
wfo-0000482363	*Peperomia coarctata*	328	move to Peperomia sanjoseana C.DC.
wfo-0000482362	*Peperomia coliblancoana*		move to Peperomia tenuipes Trel.
wfo-0000482367	*Peperomia collicola*		move to Peperomia quadrifolia (L.) Kunth
wfo-0000478138	*Peperomia congestifolia*		move to Peperomia silvivaga C.DC.
wfo-0000478142	*Peperomia constanzana*		move to Peperomia blanda (Jacq.) Kunth
wfo-0000478144	*Peperomia contraria*		move to Peperomia magnoliifolia (Jacq.) A.Dietr.
wfo-0000478145	*Peperomia controversa*	42	move to Peperomia elongata Kunth
wfo-0000478087	*Peperomia conulifera*		move to Peperomia magnoliifolia (Jacq.) A.Dietr.
wfo-0000478090	*Peperomia cooperi*	37	move to Peperomia sancarlosiana C.DC.
wfo-0000478092	*Peperomia copeyana*		move to Peperomia olivacea C.DC.

wfo-0000478081	*Peperomia corozosana*		move to Peperomia foraminum C.DC.
wfo-0000478082	*Peperomia costaricensis*	15	move to Peperomia hirta C.DC.
wfo-0000478086	*Peperomia courtallensis*	391	move to Peperomia portulacoides (Lam.) A.Dietr.
wfo-0000478106	*Peperomia crassiuscula*	328	move to Peperomia angustata Kunth
wfo-0000478108	*Peperomia crispipila*		move to Peperomia cogniauxii Urb.
wfo-0000478109	*Peperomia cryptolepida*		move to Peperomia silvivaga C.DC.
wfo-0000478100	*Peperomia cubilquitziana*	176	nomen nudum, delete because not formally described or assigned to any species
wfo-0000478101	*Peperomia cueroensis*	33	move to Peperomia guadaloupensis C.DC.
wfo-0000478095	*Peperomia cufodontii*		move to Peperomia hylophila C.DC.
wfo-0000477296	*Peperomia cylindribacca*		move to Peperomia macrostachyos (Vahl) A.Dietr.
wfo-0000478508	*Peperomia dantoana*	408	move to Peperomia granulosa Trel.
wfo-0000478509	*Peperomia darisiana*		please correct to Peperomia jarisiana C.DC.
wfo-0000478506	*Peperomia davisii*		move to Peperomia urocarpa Fisch. & Mey.
wfo-0000478512	*Peperomia dawsonii*		move Peperomia heterophylla Miq.
wfo-0000478514	*Peperomia decaisnei*		move to Peperomia elliptica (Lam.) A.Dietr.
wfo-0000477306	*Peperomia defluens*		move to Peperomia macrostachyos (Vahl) A.Dietr.
wfo-0000477307	*Peperomia defrenata*		move to Peperomia martiana Miq.
wfo-0000477311	*Peperomia delecta*		move to Peperomia silvivaga C.DC.
wfo-0000477312	*Peperomia demissa*		move to Peperomia jamesoniana C.DC.
wfo-0000477313	*Peperomia dendroides*	29	move to Peperomia galioides Kunth
wfo-0000477314	*Peperomia dendromorphis*	29	move to Peperomia galioides Kunth
wfo-0000477310	*Peperomia dentulibractea*		move to Peperomia latifolia Miq.
wfo-0001041059	*Peperomia desfontainesii*		move to Peperomia circinnata Link
wfo-0000477301	*Peperomia dextrolaeva*	198	move to Peperomia oahuensis C.DC.
wfo-0000477304	*Peperomia dincorana*		to correct to Peperomia sincorana C.DC.
wfo-0001094051.	*Peperomia dindygulensis*	125	move to Peperomia leptostachya Hook. & Arn.
wfo-0000478486	*Peperomia disparifolia*	326	move to Peperomia hirta C.DC.
wfo-0000478488	*Peperomia dissitiflora*		move to Peperomia hirtella Miq.
wfo-0000478478	*Peperomia doleana*	198	move to Peperomia myrtifolia (Vahl) A.Dietr.
wfo-0000478479	*Peperomia dolosa*		move to Peperomia myrtifolia (Vahl) A.Dietr.
wfo-0000478480	*Peperomia dolosa*		move to Peperomia myrtifolia (Vahl) A.Dietr.
wfo-0000478503	*Peperomia duidana*	150	move to Peperomia reptans C.DC.
wfo-0000478493	*Peperomia dussii*		move to Peperomia hirtella Miq.
wfo-0000478561	*Peperomia ellipticifolia*		move to Peperomia blanda (Jacq.) Kunth
wfo-0000478566	*Peperomia emarginata*		move to Peperomia obtusifolia (L.) A.Dietr.
	Peperomia emarginulata	29	you mention twice the same reference for this species?
wfo-0000478552	*Peperomia enantiostachya*	39	move to Peperomia trianae C.DC.
wfo-0001092498	*Peperomia enckeaefolia*	29	this is a Piper species, delete
wfo-0000478550	*Peperomia ephemera*		move to Peperomia pellucida (L.) Kunth
wfo-0001093881	*Peperomia epilobioides*	368	move to Peperomia rotundata Kunth
wfo-0000478557	*Peperomia erasmia*		move to Peperomia lancifolia Hook.
wfo-0000478567	*Peperomia ernstiana*		move to Peperomia venezueliana C.DC.
wfo-0000478575	*Peperomia erythrophlebia*		move to Peperomia martiana Miq,
wfo-0000478571	*Peperomia evadens*		move to Peperomia hirtella Miq.
wfo-0000478568	*Peperomia exuberantifolia*		move to Peperomia palmana C.DC.
wfo-0000478528	*Peperomia faucium-bovis*		move to Peperomia portobellensis Beurl.
wfo-0001093980	*Peperomia ferreyrae*	29	epiphytic very doubtful
wfo-0000478534	*Peperomia filici-decorans*		move to Peperomia rhombea Ruiz & Pav.
wfo-0000478518	*Peperomia filispica*		move to Peperomia sancarlosiana C.DC.
wfo-0000478521	*Peperomia fimbriata*		move to Peperomia trifolia (L.) A.Dietr.
wfo-0000478522	*Peperomia fimbribractea*		move to Peperomia hirta C.DC.
wfo-0000478517	*Peperomia flagelliformis*		move to Peperomia inaequalifolia Ruiz & Pav.
wfo-0000478524	*Peperomia flagitans*	328	move to Peperomia succulenta C.DC.
wfo-0000478535	*Peperomia flavidinervis*		move to Peperomia velloziana Miq.
wfo-0000478543	*Peperomia flavinerva*		move to Peperomia cookiana C.DC.
wfo-0000478544	*Peperomia flexuosa*		move to Peperomia subroseispica C.DC.
wfo-0000478549	*Peperomia floribunda*	328	move to Peperomia lancifolia Hook.
wfo-0000478542	*Peperomia floridensis*		move to Peperomia grisebachii C.DC.
	Peperomia foliiflora	29	twice the same reference?, phyllantha is not as synonym of P. foliiflora
wfo-0000478548	*Peperomia fontinalis*		move to Peperomia reflexa Kunth
wfo-0000478546	*Peperomia formonensis*		move to Peperomia obtusifolia
wfo-0000478538	*Peperomia foveolata*	150	move to Peperomia distachyos (L.) A.Dietr.
wfo-0000478541	*Peperomia fraijanesana*		move to Peperomia hylophila C.DC.
wfo-0000478560	*Peperomia friabilis*		move to Peperomia angustata Kunth
	Peperomia fugax	43	move to Peperomia tuerckheimii C.DC.
wfo-0000478376	*Peperomia fumeana*		move to Peperomia urocarpa Fisch. & Mey.
wfo-0000478382	*Peperomia galeottiana*	110	move to Peperomia mexicana Miq.
wfo-0000478369	*Peperomia galiifolia*		move to Peperomia galioides Kunth
wfo-0000478379	*Peperomia gallitoensis*		move to Peperomia galioides Kunth
wfo-0000478367	*Peperomia garrapatilla*		move to Peperomia galioides Kunth
wfo-0000478386	*Peperomia gentianifolia*		move to Peperomia saligna Kunth
wfo-0000478393	*Peperomia gibbonsii*	198	move to Peperomia ponapensis C.DC.
wfo-0000478394	*Peperomia gibbsiae*		move to Peperomia lasiostigma C.DC.
wfo-0000478398	*Peperomia glaberrima*		move to Peperomia macrostachyos (Vahl) A.Dietr.
wfo-0000478392	*Peperomia glabra*		move to Peperomia adscendens C.DC.
wfo-0000478399	*Peperomia glabricaulis*		move to Peperomia macrostachyos (Vahl) A.Dietr.
wfo-0000478400	*Peperomia glabripes*		move to Peperomia glazioui C.DC.
wfo-0000478401	*Peperomia glabriramea*		move to Peperomia macrostachyos (Vahl) A.Dietr.

wfo-0000478389	*Peperomia goetzeana*	90	move to Peperomia abyssinica Miq.
wfo-0000478390	*Peperomia gollii*	325	move to Peperomia obtusifolia (L.) A.Dietr.
wfo-0000478337	*Peperomia gracilipeduncula*	414	move to Peperomia succulenta C.DC.
wfo-0000478338	*Peperomia granata*		move to Peperomia galioides Kunth
wfo-0000478329	*Peperomia guanacastana*		move to Peperomia pseudopereskiifolia C.DC.
	Peperomia guarujana	51	move to Peperomia corcovadensis Gardn.
wfo-0000478334	*Peperomia guayabillosana*		move to Peperomia galioides Kunth
wfo-0000478355	*Peperomia hahnii*		move to Peperomia hirtella Miq.
wfo-0000478363	*Peperomia hamiltoniana*	152	move to Peperomia simplex Ham.
wfo-0000478347	*Peperomia hawarthii*		move to Peperomia haworthiana A.Dietr.
wfo-0000478350	*Peperomia hederacea*		move to Peperomia urocarpa Fisch. & Mey.
wfo-0000478447	*Peperomia herbertsmithii*	373	move to Peperomia ouabianae C.DC.
wfo-0000478456	*Peperomia heydei*		move to Peperomia asarifolia Schltdl. & Cham.
wfo-0000478459	*Peperomia hiloana*		move to Peperomia cookiana C.DC.
wfo-0000478407	*Peperomia hircina*		move to Peperomia borbonensis Miq.
wfo-0000478411	*Peperomia hirsutifolia*		move to Peperomia humilis A.Dietr.
wfo-0000478405	*Peperomia hochreutineri*		move to Peperomia hesperomannii Wawra
wfo-0000478416	*Peperomia hoeferi*		move to Peperomia guamana C.DC.
wfo-0000478404	*Peperomia houelmonte*		move to Peperomia nigropunctata Miq.
wfo-0000478406	*Peperomia huahinensis*		move to Peperomia societatis Moore
wfo-0000478422	*Peperomia huitzensis*		move to Peperomia striata Ruiz. & Pav.
wfo-0000478429	*Peperomia hygrophila*		move to Peperomia vulcanica Baker & Wright
wfo-0000478423	*Peperomia ilerrerae*		orthographic error in IPNI, this is Peperomia herrerae Trel.
wfo-0000478425	*Peperomia inaudax*		move to Peperomia tenuipes Trel.
wfo-0000478426	*Peperomia incrassata*		move to Peperomia sanjoseana C.DC.
wfo-0000477586	*Peperomia insularum*		move to Peperomia leptostachya Hook. & Arn.
wfo-0000477587	*Peperomia intermixta*		move to Peperomia portoricensis Urb.
wfo-0000477581	*Peperomia ionophylla*		move to Peperomia serpens (Sw.) Loudon
wfo-0000477590	*Peperomia isidroana*		move to Peperomia dotana Trel.
wfo-0000477574	*Peperomia izalcoana*		move to Peperomia bernoullii C.DC.
wfo-0000477573	*Peperomia jamesonii*		move to Peperomia galioides Kunth
wfo-0000477576	*Peperomia jarisiana*		move to Peperomia pseudopereskiifolia C.DC.
wfo-0000477577	*Peperomia javanica*		move to Peperomia cookiana Miq.
wfo-0000477578	*Peperomia jilotepequeana*	328	move to Peperomia lignescens C.DC.
wfo-0000477579	*Peperomia johnsonii*		move to Peperomia enervis von Mueller & C.DC.
wfo-0000477607	*Peperomia kandavuana*	409	move to Peperomia lasiostigma C.DC.
wfo-0000477603	*Peperomia kauaiensis*		move to Peperomia hesperomannii Wawra
	Peperomia killipi	60	move to Peperomia tenuifolia C.DC.
wfo-0000477598	*Peperomia knoblecheriana*		move to Peperomia pellucida (L.) Kunth
wfo-0000477608	*Peperomia knudsenii*		move to Peperomia cookiana C.DC.
wfo-0000477606	*Peperomia koepperi*	408	move to Peperomia rotundifolia (L.) Kunth
wfo-0000477601	*Peperomia kyimbilana*		move to Peperomia leptostachya Hook. & Arn.
wfo-0000477542	*Peperomia laevis*		move to Peperomia alata Ruiz & Pav.
wfo-0000477549	*Peperomia lancetillana*		move to Peperomia spathulifolia Small
wfo-0001093050	*Peperomia lanifolioidea*	57	could not find this name as formally published
	Peperomia lanifolioidea	37	could not find this name as formally published
wfo-0000477532	*Peperomia lanjouwii*	261	move to Peperomia rotundifolia
wfo-0000477534	*Peperomia larana*	373	move to Peperomia tetraquetra Sodiro
wfo-0000477561	*Peperomia laudabilis*		move to Peperomia succulenta C.DC.
wfo-0000477566	*Peperomia lentibacca*		move to Peperomia humilis A.Dietr.
wfo-0000477568	*Peperomia leoclemerocana*		move to Peperomia crassicaulis Fawc. & Rendl.
wfo-0001092549	*Peperomia lepadiphylla*	29	move to Peperomia nivalis Miq.
wfo-0000477554	*Peperomia leridana*		move to Peperomia maculosa (L.) Hook.
wfo-0000477556	*Peperomia leucosticta*		move to Peperomia sanjoseana C.DC.
wfo-0000477678	*Peperomia lilloi*	412	move to Peperomia santaelisae C.DC.
wfo-0000477679	*Peperomia limaensis*	29	move to Peperomia inaequalifolia Ruiz & Pav.
wfo-0000477682	*Peperomia linatifolia*		move to Peperomia glabella (Sw.) A.Dietr.
wfo-0000477690	*Peperomia lindeniana*	52	move to Peperomia hoffmannii (L. f.) A.Dietr.
wfo-0000477690	*Peperomia lindeniana*	110	move to Peperomia hoffmannii (L. f.) A.Dietr.
	Peperomia lindeniana	255	move to Peperomia hoffmannii (L. f.) A.Dietr.
wfo-0000477685	*Peperomia linearifolia*		move to Peperomia hedyotidea Ridley
wfo-0000477686	*Peperomia linearis*		move to Peperomia swartziana Miq.
wfo-0000477684	*Peperomia lonchophylla*		move to Peperomia oahuensis C.DC.
wfo-0000477668	*Peperomia longeacuminata*	325	move to Peperomia glabella (Sw.) A.Dietr., corrected from longiacuminata to longeacuminata
wfo-0000477667	*Peperomia longiflora*		move to Peperomia rubricaulis (Nees) A.Dietr.
wfo-0000477669	*Peperomia longemucronata*		move to Peperomia alpina (Sw.) A.Dietr, corrected from longimucronata to longemucronata
wfo-0000477697	*Peperomia longirama*		move to Peperomia macraeana C.DC.
wfo-0000477671	*Peperomia longirostrata*	373	move to Peperomia alpina (Sw.) A.Dietr.
wfo-0000477689	*Peperomia longispica*	29	move to Peperomia galioides Kunth
	Peperomia longispica	177	move to Peperomia galioides Kunth
wfo-0000477670	*Peperomia longispicata*		move to Peperomia angustata Kunth
wfo-0000477706	*Peperomia lundellii*	176	move to Peperomia angustata Kunth
wfo-0000477703	*Peperomia lundii*		move to Peperomia corcovadensis Gardn.
wfo-0000477709	*Peperomia luxii*		move to Peperomia quadrifolia (L.) Kunth
wfo-0000477206	*Peperomia macedoana*	255	move to Peperomia elongata Kunth
wfo-0000477212	*Peperomia macgregorii*		move to Peperomia rubrivenosa C.DC.
wfo-0000477213	*Peperomia machaerodonta*	163	move to Peperomia alpina (Sw.) A.Dietr. , corrected from machaemdonta to machaerodonta
wfo-0000477214	*Peperomia macorisiana*		move to Peperomia glabella (Sw.) A.Dietr.

wfo-0000477211	*Peperomia macrophylla*		move to Peperomia lancifolia Hook. f.
wfo-0000477209	*Peperomia macropoda*		move to Peperomia obtusifolia (L.) A.Dietr.
wfo-0000477201	*Peperomia magilensis*		move to Peperomia molleri C.DC.
wfo-0000477198	*Peperomia major*	328	move to Peperomia urocarpa Fisch. & Mey
wfo-0000477226	*Peperomia malaccensis*	376	move to Peperomia gemella Miq.
	Peperomia malaccensis	272	move to Peperomia gemella Miq.
wfo-0000477227	*Peperomia maleuvreana*	230	move to Peperomia glabella (Sw.) A.Dietr.
wfo-0000477225	*Peperomia maniensis*		move to Peperomia mauiensis Wawra
wfo-0000477218	*Peperomia mararyna*		move to Peperomia rotundifolia
	Peperomia marcapatana	255	I don
wfo-0000477223	*Peperomia martagonifolia*		move to Peperomia rhexiifolia C.DC.
wfo-0000477162	*Peperomia martini*	176	move to P. magnoliifolia
wfo-0000477205	*Peperomia martinicensis*	330	move to Peperomia nigropunctata Miq.
wfo-0000477164	*Peperomia matagalpensis*	38	move to Peperomia tenerrima Schltdl. & Cham.
wfo-0000477165	*Peperomia matapalo*		move to Peperomia tuisana C.DC.
wfo-0000477169	*Peperomia mauritiana*		move to Peperomia elliptica (Lam.) A.Dietr.
wfo-0000477166	*Peperomia megalanthera*		move to Peperomia silvivaga C.DC.
wfo-0000477506	*Peperomia microreticulata*	337	move to Peperomia alata Ruiz & Pav.
wfo-0000477495	*Peperomia minarum*	150	nom. illeg. hom., but no new name has been assigned
	Peperomia minarum	328	nom. illeg. hom., but no new name has been assigned
wfo-0000477499	*Peperomia miradoresiana*		move to Peperomia lancifolia Hook.
wfo-0000477500	*Peperomia misionense*	235	move to Peperomia arifolia Miq.; corrected from misionensis to misionense
wfo-0000477520	*Peperomia moerenhoutii*		move to Peperomia leptostachya Hook. & Arn.
wfo-0000477521	*Peperomia molithrix*	328	move to Peperomia lanceolatopeltata C.DC.
wfo-0000477524	*Peperomia mollipubis*		move to Peperomia hirta C.DC.
wfo-0000477526	*Peperomia molokaiensis*		move to Peperomia macraeana C.DC.
wfo-0000477528	*Peperomia moncionis*	97	move to Peperomia nizaitoensis C.DC.
wfo-0000477529	*Peperomia monini*		move to Peperomia pedunculata C.DC.
wfo-0000477523	*Peperomia montazosana*		move to Peperomia infravillosa Trel.
wfo-0000477509	*Peperomia montefrionis*		move to Peperomia septemnervis Ruiz & Pav.
wfo-0000477510	*Peperomia monteverdensis*	94	move to Peperomia guadaloupensis C.DC.
wfo-0000477516	*Peperomia montis-verticis*	399	move to Peperomia dendrophila Schltdl. & Cham.
wfo-0000477511	*Peperomia montium*	57	move to Peperomia silvivaga C.DC.
wfo-0000477511	*Peperomia montium*	150	move to Peperomia silvivaga C.DC.
	Peperomia montium	335	move to Peperomia silvivaga C.DC.
wfo-0000477513	*Peperomia moritzii*		move to Peperomia rotundata var. trinervula (C.DC.) Steyerm.
wfo-0000477514	*Peperomia mornicola*		move to Peperomia humilis A.Dietr.
wfo-0000477491	*Peperomia mourae*	228	move to Peperomia megapotamica Dahlst.
wfo-0001249111	*Peperomia muelleri*	110	move to Peperomia lanceolatopeltata C.DC.
wfo-0000477244	*Peperomia multifida*	317	move to Peperomia hylophila C.DC.
wfo-0000477245	*Peperomia multiplex*		move to Peperomia longifolia C.DC.
wfo-0000477246	*Peperomia multiplinervia*		move to Peperomia syringifolia C.DC.
wfo-0000477248	*Peperomia munyecoana*		move to Peperomia sanjoseana C.DC.
wfo-0000477237	*Peperomia myosuroides*		move to Peperomia macrostachyos (Vahl) A.Dietr.
wfo-0000477240	*Peperomia myrtillus*	112	move to Peperomia rhombea Ruiz & Pav.
wfo-0000477241	*Peperomia nahikuensis*	313	move to Peperomia eekana C.DC.
wfo-0000477484	*Peperomia nativitatis*	79	move to Peperomia laevifolia (Blume) Miq.
wfo-0000477481	*Peperomia negotiosa*		move to Peperomia urocarpa Fisch. & Mey.
wfo-0000477489	*Peperomia nematostachya*	42	move to Peperomia macrostachyos (Vahl) A.Dietr.
wfo-0000477490	*Peperomia nemoralis*		move to Peperomia pseudopereskiifolia C. DC.
wfo-0000477476	*Peperomia nemostachya*		move to Peperomia macrostachyos (Vahl) A.Dietr.
wfo-0000477486	*Peperomia nervosa*	23	move to Peperomia pseudocasarettoi C.DC.
wfo-0001093054	*Peperomia nievecitana*	57	move to Peperomia oerstedii C.DC.
wfo-0000477474	*Peperomia nigrescens*	331	move to Peperomia nigropunctata Miq.
wfo-0000477477	*Peperomia nilssonii*	336	move to Peperomia alata Ruiz & Pav.
wfo-0000477745	*Peperomia niveopunctulata*		move to Peperomia versicolor Trel.
wfo-0000477888	*Peperomia novae-helvetiae*	3	move to Peperomia dendrophila Schltdl. & Cham.
wfo-0000477889	*Peperomia novae-hispaniae*	401	move to Peperomia urocarpa Fisch. & Mey.
wfo-0000477897	*Peperomia novae-zelandiae*		move to Peperomia tetraphylla Hook. & Arn.
wfo-0000477891	*Peperomia novella*	402	move to Peperomia olivacea C.DC.
wfo-0000477895	*Peperomia nudilimba*		move to Peperomia macraeana C.DC.
wfo-0000477880	*Peperomia nudipeduncula*		move to Peperomia hypoleuca Miq.
wfo-0000477892	*Peperomia nudipetiola*		move to Peperomia macraeana C.DC.
wfo-0000477890	*Peperomia nummularia*		move to Peperomia hirta C.DC.
wfo-0000477878	*Peperomia nummularifolia*	134	move to Peperomia rotundifolia (L.) Kunth
wfo-0000477879	*Peperomia oajacensis*	170	move to Peperomia leptophylla Miq.
wfo-0000477882	*Peperomia oblanceolata*	57	move to Peperomia guadaloupensis C.DC.
wfo-0000477884	*Peperomia oblongibacca*	254	move to Peperomia macrostachyos (Vahl) A.Dietr.
wfo-0000477904	*Peperomia oblongifolia*		move to Peperomia galioides Kunth
wfo-0000477896	*Peperomia obovata*		move to Peperomia hoffmannii C.DC.
wfo-0000477913	*Peperomia obversa*		move to Peperomia grisebachii C.DC.
wfo-0000477918	*Peperomia obversa*		move to Peperomia trifolia (L.) A.Dietr.
	Peperomia occulta	43	I don
wfo-0000477923	*Peperomia okarana*		move to Peperomia galioides Kunth
wfo-0000477906	*Peperomia okinawensis*	394	move to Peperomia japonica
wfo-0000477919	*Peperomia olafiana*		move to Peperomia swartziana Miq.
wfo-0000477899	*Peperomia oleracea*		move to Peperomia pellucida (L.) Kunth
wfo-0000477902	*Peperomia omnicola*	233	move to Peperomia striata Ruiz & Pav.

	Peperomia omnicola	29	move to Peperomia striata Ruiz & Pav.
	Peperomia omnicola	373	move to Peperomia striata Ruiz & Pav.
	Peperomia omnicola	37	move to Peperomia striata Ruiz & Pav.
wfo-0000477910	*Peperomia opaca*		move to Peperomia tetraphylla Hook. & Arn.
wfo-0000477905	*Peperomia opacilimba*		move to Peperomia cookiana C.DC.
wfo-0001092581	*Peperomia opiziana*	29	move to Peperomia dependens Ruiz & Pav.
wfo-0000477908	*Peperomia orbiculata*		move to Peperomia verticillata (L.) A.Dietr. (
wfo-0000477875	*Peperomia orientalis*	318	move to Peperomia naranjoana C.DC.
wfo-0000477847	*Peperomia ornata*	413	move to Peperomia maypurensis Kunth
wfo-0000477885	*Peperomia osana*		move to Peperomia urocarpa Fisch. & C.A. Mey.
wfo-0001223653.	*Peperomia ovatolanceolata*	373	move to Peperomia distachyos (L.) A.Dietr.
wfo-0000477854	*Peperomia oxycarpa*		move to Peperomia portobellensis Beurl.
wfo-0000477843	*Peperomia pachycaulis*		move to Peperomia macraeana C.DC.
wfo-0000477851	*Peperomia pachyphlebia*		move to Peperomia alpina (Sw.) A.Dietr.
wfo-0000477837	*Peperomia pachyphylla*		move to Peperomia sandwicensis Miq.
wfo-0000477838	*Peperomia pacifica*		move to Peperomia pacificicola Hosokawa
wfo-0000477844	*Peperomia palcipila*	67	move to Peperomia tenella (Sw.) A.Dietr.
wfo-0000477877	*Peperomia palpebrata*	331	move to Peperomia nigropunctata Miq.
wfo-0000477866	*Peperomia panaiana*		move to Peperomia pallidibacca C.DC.
wfo-0000477873	*Peperomia panamensis*	373	move to Peperomia silvivaga C.DC.
	Peperomia panamensis	37	move to Peperomia silvivaga C.DC.
wfo-0001249112	*Peperomia papantlacensis*	110	move to Peperomia berlandieri Miq.
wfo-0000477871	*Peperomia papulata*		move to Peperomia verticillata (L.) A.Dietr.
wfo-0000477861	*Peperomia parmata*		move to Peperomia maculosa (L.) Hook.
wfo-0000477864	*Peperomia parvanthera*		move to Peperomia rockii C.DC.
wfo-0000477979	*Peperomia pascuicola*	219	move to Peperomia sanjoseana C.DC.
	Peperomia pelucida	138	wrongly spelled, move to Peperomia pellucida
wfo-0000477978	*Peperomia pendula*	365	move to Peperomia macrostachyos (Vahl) A.Dietr.
wfo-0000478005	*Peperomia pennellii*	373	terrestrial or epiphytic according to protologue
wfo-0000478006	*Peperomia penninervia*		move to Peperomia goudotii Miq.
wfo-0000477994	*Peperomia perherbacea*	99	move to Peperomia petiolaris C.DC.
wfo-0000477932	*Peperomia perinduta*	231	move to Peperomia foraminum C.DC.
wfo-0000477930	*Peperomia perplexa*	403	move to Peperomia granulosa Trel.
wfo-0000477931	*Peperomia perrottetiana*		move to Peperomia portulacoides (Lam.) A.Dietr.
wfo-0000477937	*Peperomia perrottetiana*		name uncertain
wfo-0000477926	*Peperomia persuccosa*	77	move to Peperomia myrtifolia (Vahl) A.Dietr.
wfo-0000477934	*Peperomia petenensis*		move to Peperomia magnoliifolia (Jacq.) A.Dietr.
wfo-0000477966	*Peperomia piedrana*		move to Peperomia guadaloupensis C.DC.
wfo-0000477967	*Peperomia pililimba*	328	move to Peperomia tuisana C.DC.
	Peperomia pililimba	325	move to Peperomia tuisana C.DC.
	Peperomia pililimba	302	move to Peperomia tuisana C.DC.
wfo-0000477965	*Peperomia pilipeduncula*		move to Peperomia humilis A.Dietr.
wfo-0000490504	*Peperomia pilipedunculata*		name uncertain
wfo-0000477954	*Peperomia pilosula*		move to Peperomia urocarpa Fisch. & Mey.
wfo-0000477957	*Peperomia piresii*	42	move to Peperomia elongata Kunth
	Peperomia piresii	255	move to Peperomia elongata Kunth
wfo-0000477958	*Peperomia pirrisana*	371	move to Peperomia quadrangularis (Thomps.) A.Dietr.
wfo-0000477764	*Peperomia pleistostachya*	148	move to Peperomia cookiana C.DC
wfo-0000477765	*Peperomia plinervata*		move to Peperomia membranacea Hook. & Arn.
wfo-0000477771	*Peperomia pluvigaudens*		move to Peperomia hypoleuca Miq.
wfo-0000477768	*Peperomia polochicana*	176	move to Peperomia deppeana Schltdl. & Cham.
wfo-0000477769	*Peperomia pololensis*		move to Peperomia glabella (Sw.) A.Dietr.
wfo-0000477757	*Peperomia porriginifera*	150	move to Peperomia blanda
	Peperomia porriginifera	373	move to Peperomia blanda
wfo-0000477758	*Peperomia porschiana*	372	move to Peperomia hylophila C.DC.
wfo-0000477770	*Peperomia pothifolia*	320	move to Peperomia striata Ruiz. & Pav.
wfo-0000477781	*Peperomia praecox*		move to Peperomia urocarpa Fisch. & C.A. Mey.
wfo-0000477789	*Peperomia preussii*	256	move to Peperomia fernandopoiana C.DC.
	Peperomia pruinosifolia	178	epiphytic seems very unlikely
	Peperomia pseudoestrellensis	51	move to Peperomia elsiae Marcusso
	Peperomia pseudoestrellensis	82	move to Peperomia elsiae Marcusso
	Peperomia pseudoestrellensis	255	move to Peperomia elsiae Marcusso
	Peperomia pseudoestrellensis	220	move to Peperomia elsiae Marcusso
	Peperomia pseudoestrellensis	221	move to Peperomia elsiae Marcusso
	Peperomia pseudoestrellensis	85	move to Peperomia elsiae Marcusso
	Peperomia pseudoestrellensis	128	move to Peperomia elsiae Marcusso
wfo-0000477780	*Peperomia pseudogalapagensis*	29	move to Peperomia inaequalifolia Ruiz & Pav.
wfo-0000477727	*Peperomia pseudojamesoniana*		move to Peperomia jamesoniana C.DC.
wfo-0000490513	*Peperomia pseudomajor*	66	move to Peperomia urocarpa Fisch. & Mey.
wfo-0000477732	*Peperomia pseudopeltoidea*		move to Peperomia peltoidea Kunth
wfo-0000477719	*Peperomia psiloclada*		move to Peperomia sanjoseana C.DC.
wfo-0000477715	*Peperomia psilostigma*		move to Peperomia macraeana C.DC.
wfo-0000477750	*Peperomia pukooana*		move to Peperomia cookiana C.DC.
wfo-0000477746	*Peperomia pulchella*		move to Peperomia verticillata (L.) A.Dietr.
wfo-0000477739	*Peperomia punaluuna*		move to Peperomia latifolia Miq.
wfo-0000477749	*Peperomia punctatifolia*	321	move to Peperomia matlalucaensis C.DC.
wfo-0000477738	*Peperomia purpurascens*		move to Peperomia rotundata Kunth
wfo-0000477741	*Peperomia purpurella*		move to Peperomia patula C.DC.
wfo-0000750082	*Peperomia purpureonervosa*	292	I would say 100% lithophyte
wfo-0000477744	*Peperomia pustulatibacca*	333	move to Peperomia magnoliifolia (Jacq.) A.Dietr.

wfo-0000477806	*Peperomia puteolifera*	325	move to Peperomia obtusifolia (L.) A.Dietr.
wfo-0000477282	*Peperomia pyrifolia*	386	move to Peperomia acuminata Ruiz & Pav.
wfo-0000477287	*Peperomia quadrivii*	370	move to Peperomia glabella (Sw.) A.Dietr.
wfo-0001093059	*Peperomia quaternata*	57	move to Peperomia tetraphylla Hook. & Arn.
wfo-0000477286	*Peperomia queserana*	366	move to Peperomia adscendens C.DC.
wfo-0000477285	*Peperomia questeliana*	331	move to Peperomia humilis A.Dietr.
wfo-0000477273	*Peperomia quicheensis*	176	move to Peperomia pereskiifolia (Jacq.) Kunth
wfo-0000477280	*Peperomia quiriguana*	176	move to Peperomia glabella (Sw.) A.Dietr.
wfo-0000477277	*Peperomia quirosii*		move to Peperomia pseudodependens C.DC.
wfo-0000477276	*Peperomia raiateensis*	225	move to Peperomia societatis Moore
wfo-0000477819	*Peperomia raivavaeana*	314	move to Peperomia australana Yunck.
wfo-0000477826	*Peperomia ramosa*	102	move to Peperomia humilis A.Dietr.
wfo-0000477827	*Peperomia ramosii*		move to Peperomia pallidibacca C.DC.
wfo-0000477828	*Peperomia ramulosa*	10	move to Peperomia inaequalifolia Ruiz & Pav.
wfo-0000477831	*Peperomia recurvata*	238	move to Peperomia convexa Miq.
wfo-0000477832	*Peperomia redondoana*		move to Peperomia galioides Kunth
wfo-0000477823	*Peperomia refractifolia*		move to Peperomia cookiana C.DC.
wfo-0000477820	*Peperomia rehmannii*		move to Peperomia retusa (L.f.) A.Dietr.
wfo-0000477821	*Peperomia reinwardtiana*	217	move to Peperomia convexa Miq.
wfo-0000477814	*Peperomia rhodophlebia*	405	move to Peperomia angustata Kunth
wfo-0000477810	*Peperomia rhomboides*		move to Peperomia stellata (Sw.) A.Dietr.
wfo-0000477797	*Peperomia rinconensis*	406	move to Peperomia heterophylla Miq.
wfo-0000477798	*Peperomia rio-cangrejalensis*	408	move to Peperomia granulosa Trel.
wfo-0000477256	*Peperomia rivi-vetusti*	4	move to Peperomia san-joseana C. DC.
wfo-0000477257	*Peperomia rivulorum*	70	move to Peperomia rubrivenosa C.DC.
wfo-0000479395	*Peperomia rodriguezii*		move to Peperomia rodriguesiana Balf. f.
wfo-0000490461	*Peperomia roigana*		move to Peperomia verticillata (L.) A.Dietr.
wfo-0000479396	*Peperomia rollingo*		unplaced name, delete
wfo-0000479397	*Peperomia romaensis*	408	move to Peperomia magnoliifolia (Jacq.) A. Dietr.
wfo-0000479405	*Peperomia roraimana*	378	move to Peperomia trinervula C.DC.
wfo-0001094193.	*Peperomia roseocaulis*	373	move to Peperomia stelechophila C.DC.
wfo-0000490459	*Peperomia rotundifolia f. ovata*	86	move to Peperomia rotundifolia (L.) Kunth
wfo-0000479391	*Peperomia rubefacta*	408	move to Peperomia glabella (Sw.) A.Dietr.
wfo-0000479408	*Peperomia rubioides*		move to Peperomia rhombea Ruiz & Pav.
wfo-0000479416	*Peperomia rubripetiola*		move to Peperomia quadrifolia (L.) Kunth
wfo-0000490444	*Peperomia rubripetiolata*		uncertain this is a correct name
wfo-0000479421	*Peperomia ruiziana*		move to Peperomia acuminata Ruiz & Pav.
wfo-0000479410	*Peperomia rungwensis*		move to Peperomia abyssinica Miq.
wfo-0000479418	*Peperomia rupertiana*	32	move to Peperomia myrtifolia (Vahl) A.Dietr.
wfo-0000479417	*Peperomia rupestris*	128	move to Peperomia macrostachyos (Vahl) A.Dietr.
wfo-0000479409	*Peperomia rurutana*	316	move to Peperomia australana Yunck.
wfo-0000479412	*Peperomia ruwenzoriensis*	270	move to Peperomia fernandopoiana C.DC.
wfo-0000479413	*Peperomia saipana*		move to Peperomia guamana C.DC.
wfo-0000479361	*Peperomia saldasiana*		move to Peperomia corcovadensis Gardn.
wfo-0000479363	*Peperomia salmensis*		move to Peperomia oahuensis C.DC.
wfo-0000479370	*Peperomia saltivagans*		move to Peperomia silvivaga C.DC.
wfo-0000479364	*Peperomia salvaje*		move to Peperomia blanda (Jacq.) Kunth
wfo-0000479353	*Peperomia sandersii*		move to Peperomia argyreia Morr.
wfo-0000479358	*Peperomia sanramonensis*		move to Peperomia silvivaga C.DC.
wfo-0000479357	*Peperomia santa-rosana*		move to Peperomia quadrifolia (L.) Kunth
wfo-0000479369	*Peperomia sarcocarpa*	392	move to Peperomia angustata Kunth
wfo-0000479380	*Peperomia sarcodes*	5	move to Peperomia adscendens C.DC.
wfo-0000479381	*Peperomia sarcostigma*		move to Peperomia eekana C.DC.
wfo-0000479385	*Peperomia saxigaudens*	142	move to Peperomia increscens Miq.
wfo-0000479375	*Peperomia schiedeana*	300	move to Peperomia tenerrima Schltdl. & Cham.
wfo-0000479371	*Peperomia schizostachya*		move to Peperomia claytonioides Kunth
wfo-0000479376	*Peperomia sciaphila*		move to Peperomia claytonioides Kunth
wfo-0000479466	*Peperomia secundiflora*		move to Peperomia secunda Ruiz & Pav.
wfo-0000479470	*Peperomia semidecurrens*	103	move to Peperomia erythropremna Trel.
wfo-0000479471	*Peperomia semielongata*	29	move to Peperomia crotalophora Trel.
wfo-0000479458	*Peperomia serpyllifolia*		move to Peperomia elliptica (Lam.) A.Dietr.
wfo-0000479461	*Peperomia sessilifolia*	43	these data correspond to the species in the next entry, and the name in this entry should be deleted as it is not valid
wfo-0000490440	*Peperomia sessilifoliodes*		superfluous name for the species above, delete
wfo-0000479463	*Peperomia setchellii*	303	move to Peperomia hombronii C.DC.
wfo-0000479474	*Peperomia siguaneana*	104	move to Peperomia hirta C.DC.
wfo-0000479492	*Peperomia siziana*		move to Peperomia naranjoana C. DC.
wfo-0000479432	*Peperomia snodgrassii*	68	move to Peperomia inaequalifolia Ruiz & Pav.
wfo-0000479438	*Peperomia speciosa*		this is a Piper species, delete
wfo-0000479439	*Peperomia sphagnicola*		move to Peperomia tenuipes Trel.
wfo-0000479422	*Peperomia staminea*	328	move to Peperomia emiliana C.DC.
	Peperomia staminea	325	move to Peperomia emiliana C.DC.
wfo-0000479423	*Peperomia standleyi*		move to Peperomia deppeana Schltdl. & Cham.
wfo-0000479424	*Peperomia staudtii*	109	move to Peperomia fernandopoiana C.DC.
wfo-0000479429	*Peperomia stehleana*	329	move to Peperomia nigropunctata Miq.
	Peperomia stenocarpa	156	move to Peperomia macrostachyos (Vahl) A.Dietr.
wfo-0000479427	*Peperomia stenophyllopsis*	322	move to Peperomia angularis C.DC.
wfo-0000479446	*Peperomia stolzii*		move to Peperomia molleri C.DC
wfo-0000479447	*Peperomia storkii*	326	move to Peperomia san-joseana C. DC.
wfo-0000479451	*Peperomia stuhlmannii*		move to Peperomia abyssinica Miq.

wfo-0000479452	*Peperomia suaveolens*		move to Peperomia galioides Kunth
wfo-0000479453	*Peperomia suavis*		move to Peperomia maculosa Hook.
wfo-0000479454	*Peperomia subacaulis*		move to Peperomia pernambucensis Miq.
wfo-0000479441	*Peperomia subbracteiflora*	92	move to Peperomia hirtella Miq.
wfo-0000479442	*Peperomia subcorymbosa*		move to Peperomia galioides Kunth
wfo-0000479238	*Peperomia subgeminispica*		move to Peperomia obtusifolia (L.) A. Dietr.
wfo-0000479378	*Peperomia subglabra*	36	move to Peperomia pallida (Forst. f.) A.Dietr.
wfo-0000479259	*Peperomia subnudilimba*	73	move to Peperomia globulanthera C.DC.
wfo-0000479260	*Peperomia subnudipetiola*		move to Peperomia macraeana C.DC.
wfo-0000479263	*Peperomia subpulchella*		move to Peperomia verticillata (L.) A.Dietr.
	Peperomia subsessilifolia	70	move to Peperomia corcovadensis Gardn.
wfo-0001091974	*Peperomia substriata*	57	move to Peperomia striata Ruiz. & Pav.
wfo-0000479256	*Peperomia substrigosa*		move to Peperomia olivacea C.DC.
wfo-0000479270	*Peperomia subvillosa*		move to Peperomia hirtella Miq.
wfo-0000479276	*Peperomia suizana*		move to Peperomia crispa Sodiro
wfo-0000479278	*Peperomia taco-tacoi*	105	move to Peperomia petiolaris C.DC.
wfo-0000479266	*Peperomia tacticana*	328	move to Peperomia rhexiifolia C.DC.
wfo-0000479267	*Peperomia tacuariana*	146	move to Peperomia tetraphylla Hook. & Arn.
wfo-0000479268	*Peperomia tafelbergensis*	373	move to Peperomia ouabianae C.DC.
	Peperomia tafelbergensis	261	move to Peperomia ouabianae C.DC.
wfo-0000479269	*Peperomia tahitensis*		move to Peperomia fosbergii Yunck.
wfo-0000479273	*Peperomia tatamana*	21	move to Peperomia crispipetiola Trel.
wfo-0000479274	*Peperomia taylorii*		move to Peperomia glabella (Sw.) A.Dietr.
wfo-0000479250	*Peperomia tecticola*		move to Peperomia lanceolatopeltata C.DC.
wfo-0000479220	*Peperomia tenebraegaudens*		move to Peperomia maculosa (L.) Hook.
wfo-0001229301.	*Peperomia tenuiflora*	373	move to Peperomia pellucida (L.) Kunth
wfo-0000479212	*Peperomia tenuinervis*		move to Peperomia hirta C.DC.
wfo-0000479242	*Peperomia thionvilleana*		move to Peperomia nigropunctata Miq.
wfo-0000479247	*Peperomia thwaitesii*		move to Peperomia leptostachya Hook. & Arn.
wfo-0000479248	*Peperomia tilarana*		move to Peperomia naranjoana C. DC.
wfo-0000479245	*Peperomia tiniannensis*		move to Peperomia kraemeri C.DC.
wfo-0000479235	*Peperomia tortugana*		move to Peperomia blanda (Jacq.) Kunth
wfo-0000479289	*Peperomia translucens*		move to Peperomia pellucida (L.) Kunth
wfo-0000479325	*Peperomia treleasei*		move to Peperomia eekana C.DC.
wfo-0000479326	*Peperomia treleasei*		move to Peperomia resediflora Lind. & Andr
wfo-0000479327	*Peperomia tremuliformis*		move to Peperomia urocarpa Fisch. & Mey.
wfo-0000479328	*Peperomia tressis*		move to Peperomia sanjoseana C.DC.
wfo-0000479329	*Peperomia triadophylla*		move to Peperomia pellucida (L.) Kunth
wfo-0000479333	*Peperomia trichocaulis*		move to Peperomia portoricensis Urb.
wfo-0000479334	*Peperomia trichoclada*		move to Peperomia tetraphylla
wfo-0000479315	*Peperomia trinervia*		move to Peperomia trineura Miq.
wfo-0000479321	*Peperomia tristanensis*		move to Peperomia berteroana
wfo-0000479345	*Peperomia trumani*		move to Peperomia heterophylla Miq.
wfo-0000479344	*Peperomia tuberosa*		move to Peperomia lanceolatopeltata C.DC.
wfo-0000479350	*Peperomia tuberosa*		move to Peperomia ovatopeltata C.DC.
wfo-0000477186	*Peperomia turfosa*		move to Peperomia stellata (Sw.) A.Dietr. (
wfo-0000477187	*Peperomia turialvensis*	255	move to Peperomia rhexiifolia C.DC.
	Peperomia tyleri	51	move to Peperomia tenuipes Trel.
wfo-0000477189	*Peperomia tyleri*	373	move to Peperomia tenuipes Trel.
wfo-0000477193	*Peperomia ukingensis*		move to Peperomia retusa (L.f.) A.Dietr.
wfo-0000477175	*Peperomia umbellifera*	380	move to Peperomia talinifolia Kunth
	Peperomia umbellifera	29	move to Peperomia talinifolia Kunth
	Peperomia umbellifera	373	move to Peperomia talinifolia Kunth
wfo-0000477176	*Peperomia umbricola*		move to Peperomia reineckei
wfo-0000479349	*Peperomia unguiculata*		move to Peperomia crassicaulis Fawc. & Rendl.
wfo-0000479348	*Peperomia uphofii*	327	move to Peperomia bernoullii C.DC.
wfo-0000479340	*Peperomia usambarensis*		move to Peperomia retusa (L.f.) A.Dietr.
wfo-0000479337	*Peperomia vaccinifolia*		move to Peperomia thomeana C.DC.
wfo-0000479288	*Peperomia vanheurckii*		move to Peperomia myrtifolia (Vahl) A.Dietr.
wfo-0000479323	*Peperomia vanoverberghii*		move to Peperomia recurvata
wfo-0000490472	*Peperomia variabilis*		move to Peperomia polystachyoides Dahlst.
wfo-0000479293	*Peperomia variegata*		move to Peperomia maculosa (L.) Hook.
wfo-0000479284	*Peperomia verhuellia*		move to Peperomia brasiliensis (Miq.) Miq.
wfo-0000479297	*Peperomia victoriana*	373	move to Peperomia angustata Kunth
	Peperomia victoriana	276	move to Peperomia angustata Kunth
wfo-0000479313	*Peperomia violifolia*		move to Peperomia bernoullii C.DC.
wfo-0000479314	*Peperomia viridispica*	373	move to Peperomia angustata Kunth
wfo-0000479298	*Peperomia vogelii*		move to Peperomia pellucida (L.) Kunth
wfo-0000479299	*Peperomia volkensii*		move to Peperomia ponapensis C.DC.
wfo-0000479301	*Peperomia vulcanicola*	29	move to Peperomia heterophylla Miq.
	Peperomia vulcanicola	373	move to Peperomia heterophylla Miq.
wfo-0000479307	*Peperomia wagneri*	408	move to Peperomia angustata Kunth
wfo-0000479303	*Peperomia waihoiana*		move to Peperomia latifolia Miq.
wfo-0000479304	*Peperomia waipioana*		move to Peperomia membranacea Hook. & Arn.
wfo-0000479305	*Peperomia wallichii*		move to Peperomia gemella Miq.
wfo-0001093064	*Peperomia wedelii*	57	move to Peperomia brasiliensis (Miq.) Miq.
wfo-0000479520	*Peperomia wercklei*		move to Peperomia rhexiifolia Moritz ex C. DC.
wfo-0000479702	*Peperomia wightiana*	125	Wight is not the author of this species, so I think it can be joined with P. heyneana as well
wfo-0000479703	*Peperomia wightiana*		move to Peperomia heyneana Miq.

wfo-0000479711	*Peperomia wilmsii*		move to Peperomia retusa (L.) A.Dietr.
wfo-0000479705	*Peperomia wilsonii*	198	move to Peperomia nigropunctata Miq.
	Peperomia winged	273	not a species name, impossible to know the ID of the species
wfo-0000479700	*Peperomia winkleri*		move to Peperomia molleri C.DC.
wfo-0000479706	*Peperomia wurdackii*	414	move to Peperomia ouabianae C.DC.
	Peperomia wurdackii	182	move to Peperomia ouabianae C.DC.
wfo-0000479690	*Peperomia yapensis*		move to Peperomia pellucida (L.) Kunth
wfo-0000479691	*Peperomia yaquena*	106	move to Peperomia quadrifolia (L.) Kunth
wfo-0000479689	*Peperomia yojoana*		move to Peperomia glabella (Sw.) A.Dietr.
wfo-0000479694	*Peperomia yousei*		move to Peperomia spathulifolia Small
wfo-0000479695	*Peperomia yunckeri*		move to Peperomia sancarlosiana C.DC.
wfo-0000479716	*Peperomia zenkeri*		move to Peperomia molleri C.DC.
wfo-0000479693	*Peperomia zeylanica*		move to Peperomia heyneana Miq.
wfo-0000479724	*Peperomia zurquiana*		move to Peperomia hylophila C.DC.
