# Supplementary material for: “On a tree”, “terrestrial”, or “on the rocks”? Habit diversity in the megadiverse genus Peperomia
Source: Plant Biol (Stuttg). 2026 May 13;28(5):1373–84. doi: 10.1111/plb.70214 (PMC13358651; doi:10.1111/plb.70214)
Supplement: Supplementary file 5 — Fig. S5. Map of geographical regions as listed in Table 3 (Adapted from the world geographical scheme for recording plant distribution (Brummitt 2001)). Brummitt, R. K. 2001. World geographical scheme for recording plant distributions. International working group on taxonomic databases for plants sciences. Website: https://web.archive.org/web/20160125135239/http:/www.nhm.ac.uk/hosted_sites/tdwg/TDWG_geo2.pdf [accessed 27 March 2025]. Fig. S6. Percentage of species showing the tendency to be found in the respective habitats (EV/TV/LV >50%). Species geographical distribution was obtained from Plants of the World Online, based on the regional scheme. Species occurring in each regional area have an average ETL value extracted from Table S2, and the percentage of species (within each region) with EV/TV/LV >50% was further calculated (see Table S3 below). This map must be interpreted cautiously because the ETL value from Table S2 is an average value calculated for each species across the entire geographical range, and not collated for individual regions. Hence, it do not reflect intraspecific variation in habitat use due to differences in geographical locations – which can probably be visualized at a much finer scale. Furthermore, the percentage of species that are primarily found on each habitat must be interpreted together with the total number of species found in that region. For example, it seems that a majority of species in the Arabian Peninsula are lithophytic. However, there are only three species in that region, two of which have the tendency to be found in lithophytic habitats. Nonetheless, the map is useful in showing that a large percentage of species in the Andes are indeed found terrestrially. Table S7. Percentages of species that show a tendency towards a certain habitat (EV, LV and TV >50%) for each regional area. Fig. S8. Distribution of Peperomia species in five subgenera, in their epiphyte–lithophyte–terrestrial space. Symbol size varies with the number [file PLB-28-1373-s006.docx]

Figure S3: Workflow for data compilation and analysis. An annotated R script for creating the pivot table from the database, and to plot the triangle ordination is included as an upload into the supplementary materials (ELT analysis)

Reference: Lemon, J. 2006. Plotrix: a package in the red light district of R. R-News 6: 8-12.


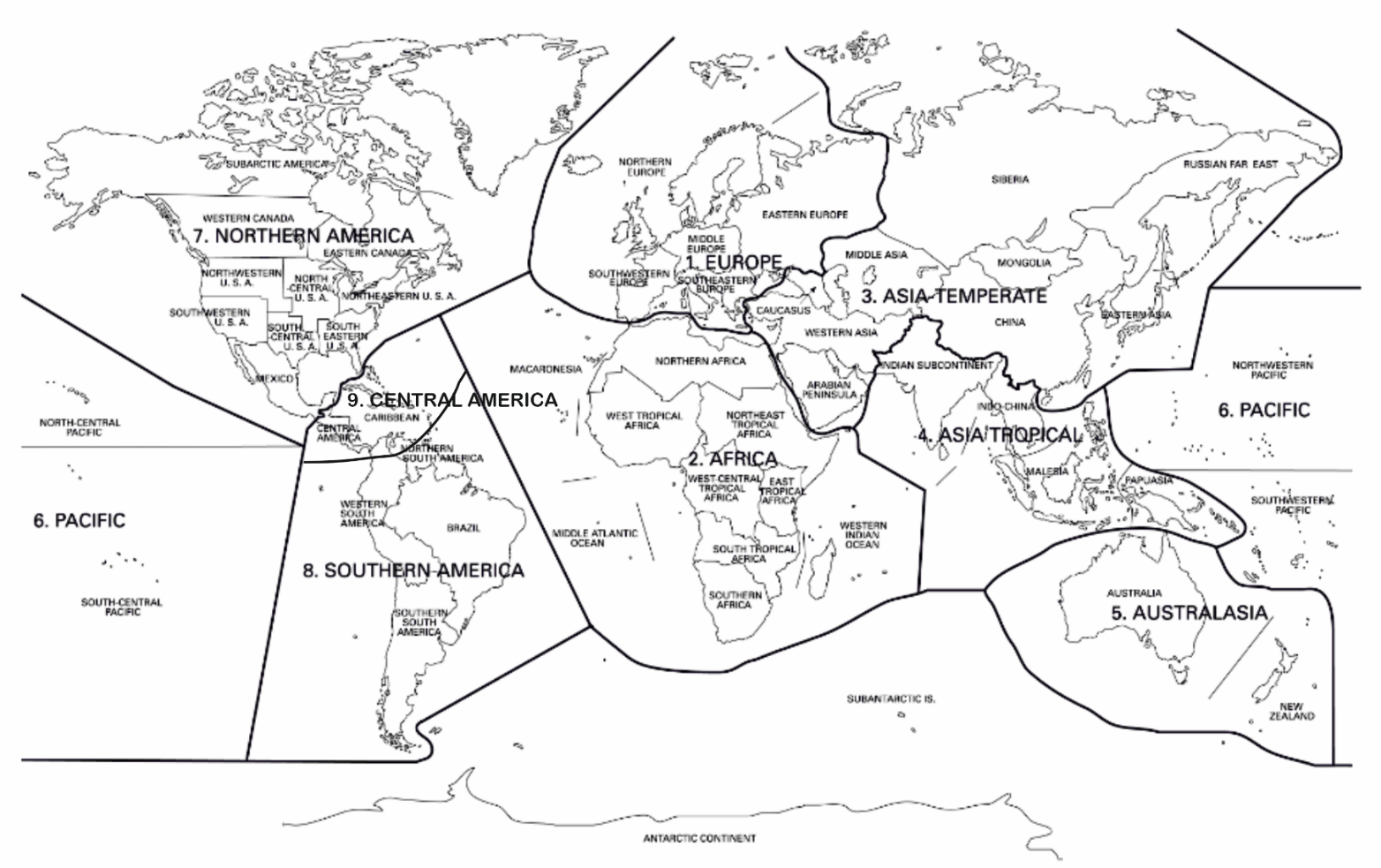


Figure S5: Map of geographical regions as listed in Table 3. (Adapted from the world geographical scheme for recording plant distribution (Brummitt, 2001)).
Brummitt, R. K. 2001. World geographical scheme for recording plant distributions. International working group on taxonomic databases for plants sciences. Website: https://web.archive.org/web/20160125135239/http:/www.nhm.ac.uk/hosted_sites/tdwg/TDWG_geo2.pdf. [accessed 27 March 2025].


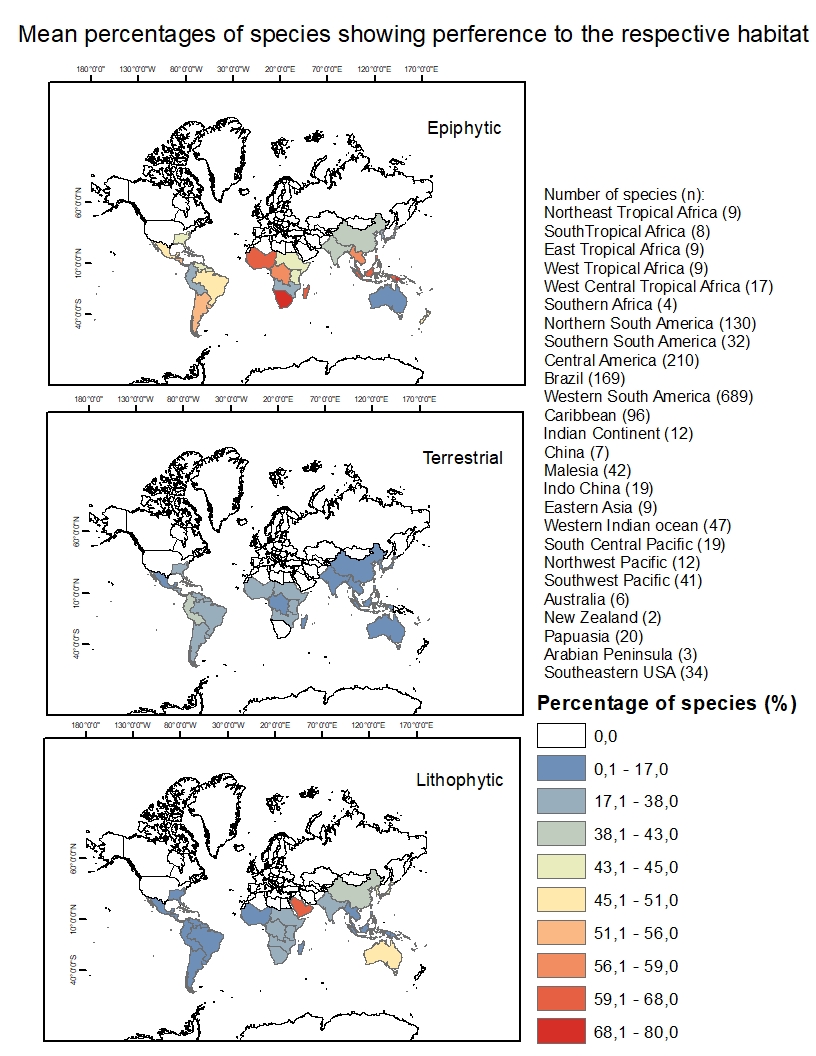


Figure S6: Percentage of species showing the tendency to be found in the respective habitats (EV/TV/LV > 50%). Species geographical distribution was obtained from Plants of the World Online, based on the regional scheme. Species occuring in each regional area have an average ETL value extracted from Table S2, and the percentage of species (within each region) with EV/TV/LV > 50% was further calculated (see Table S3 below). This map must be interpreted cautiously because the ETL value from Table S2 is an average value calculated for each species across the entire geograhical range, and not collated for individual regions. Hence, it do not reflect intraspecific variation in habitat use due to differences in geographical locations – which can probably be visualised at a much finer scale. Furthermore, the percentage of species that are primarily found on each habitat must be interpreted together with the total number of species found in that region. For example, it seems that a majority of species in the Arabian Peninsula are lithophytic. However, there are only three species in that region, two of which have the tendency to be found in lithophytic habitats. Nonetheless, the map is useful in showing that a large percentage of speces in the Andes are indeed found terrestrially.

Table S7: Percentages of species that show a tendency towards a certain habitat (EV, LV and TV > 50%) for each regional area.

| Region | Preference (value > 50) (%) | | | No. of species |
| --- | --- | --- | --- | --- |
|  | Epiphyte | Lithophyte | Terrestrial |  |
| South Tropical Africa | 38 | 25 | 25 | 8 |
| Southern South America | 51 | 2 | 30 | 53 |
| Australia | 17 | 50 | 17 | 6 |
| Caribbean | 51 | 19 | 8 | 96 |
| West Tropical Africa | 67 | 11 | 22 | 9 |
| Brazil | 50 | 11 | 18 | 169 |
| West Central Tropical Africa | 59 | 18 | 12 | 17 |
| Northeast Tropical Africa | 44 | 22 | 22 | 9 |
| China | 43 | 43 | 14 | 7 |
| Western South America | 35 | 3 | 40 | 689 |
| South Central Pacific | 42 | 16 | 11 | 19 |
| Southwest Pacific | 44 | 29 | 17 | 41 |
| Northern south america | 45 | 3 | 27 | 130 |
| Central America | 55 | 4 | 18 | 210 |
| indian Continent | 42 | 25 | 17 | 12 |
| Malesia | 67 | 17 | 5 | 42 |
| Eastern Asia | 33 | 44 | 11 | 9 |
| East Tropical Africa | 44 | 22 | 22 | 9 |
| Western Indian ocean | 68 | 9 | 15 | 47 |
| Mexico | 51 | 15 | 11 | 121 |
| Northwest Pacific | 42 | 42 | 17 | 12 |
| New Zealand | 50 | 0 | 0 | 2 |
| Papuasia | 80 | 15 | 0 | 20 |
| Arabian Peninsula | 0 | 67 | 0 | 3 |
| Southern Africa | 75 | 25 | 0 | 4 |
| Indo china | 58 | 16 | 5 | 19 |
| Southeastern USA | 50 | 0 | 38 | 8 |


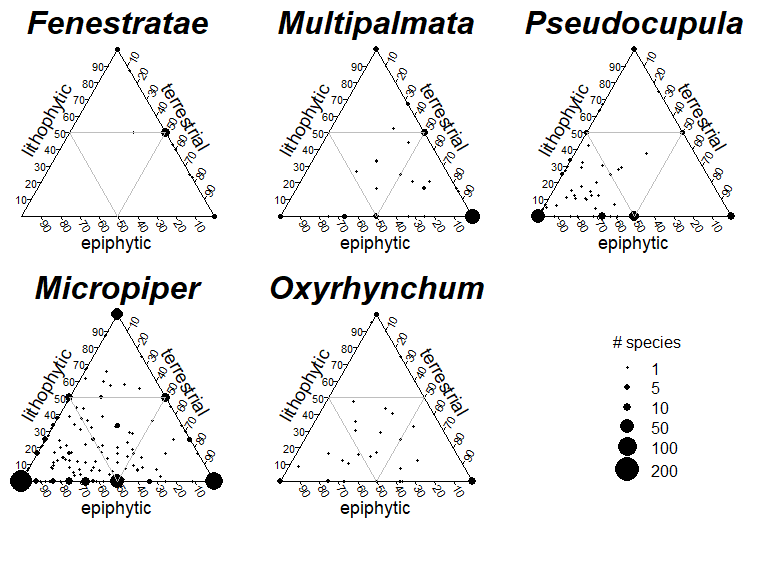


Figure S8: Distribution of *Peperomia* species in five subgenera, in their epiphyte-lithophyte-terrestrial space. Symbol size varies with the number of species that share the same values. The grey lines separate the triangle into four smaller ones. The central triangle is occupied by generalists, and the three other triangles indicate the tendency of a species to be found in an epiphytic, lithophytic or terrestrial habitat. The full data set is given in Tables S1 and S2.

Table S9: Percentages of species that are either specialists (EV, LV, and TV > 95%) or show a tendency towards a certain habitat (EV, LV and TV > 50%) for the 14 subgenera of *Peperomia*.

| Subgenus | Specialist (value > 95) (%) | | | Tendency (value > 50) (%) | | | Habitat use from Frenzke et al. 2015 |
| --- | --- | --- | --- | --- | --- | --- | --- |
|  | Epiphyte | Lithophyte | Terrestrial | Epiphyte | Lithophyte | Terrestrial |  |
| *Erasmia*  (n = 8) | 25 | 0 | 13 | 38 | 0 | 38 | Terrestrial |
| *Fenestratae*  (n = 28) | 0 | 11 | 11 | 0 | 14 | 25 | Terrestrial or lithophytic |
| *Hispidula*  (n = 6) | 0 | 0 | 33 | 17 | 0 | 67 | Terrestrial |
| *Leptorhynchum*  (n = 119) | 34 | 1 | 14 | 59 | 2 | 24 | Epiphytic, terrestrial or lithophytic |
| *Micropiper*  (n = 519) | 31 | 5 | 18 | 51 | 7 | 22 | Epiphytic or terrestrial |
| *Multipalmata*  (n = 95) | 3 | 5 | 58 | 6 | 8 | 66 | Terrestrial or lithophytic, rarely epiphytic |
| *Oxyrhynchum*  (n = 43) | 9 | 7 | 21 | 30 | 12 | 35 | Epiphytic or terrestrial |
| *Panicularia*  (n = 3) | 0 | 0 | 67 | 0 | 0 | 100 | Terrestrial |
| *Peperomia*  (n = 15) | 0 | 0 | 60 | 7 | 0 | 80 | Terrestrial, rarely epiphytic |
| *Perlucida*  (n = 7) | 0 | 0 | 86 | 0 | 0 | 100 | Terrestrial |
| *Phyllobryon*  (n = 8) | 0 | 0 | 63 | 0 | 0 | 88 | Terrestrial |
| *Pleurocarpidium*  (n = 11) | 20 | 0 | 0 | 80 | 0 | 10 | Epiphytic |
| *Pseudocupula*  (n = 132) | 35 | 3 | 7 | 67 | 5 | 7 | Mainly epiphytic, sometimes lithophytic or terrestrial |
| *Tildenia*  (n = 53) | 0 | 13 | 47 | 0 | 25 | 55 | Terrestrial or lithophytic |

Frenzke, L., E. Scheiris, G. Pino, L. Symmank, P. Goetghebeur, C. Neinhuis, S. Wanke, and M.-S. Samain. 2015. A revised infrageneric classification of the genus Peperomia (Piperaceae). Taxon 64: 424-444.
