# Supplementary material for: “On a tree”, “terrestrial”, or “on the rocks”? Habit diversity in the megadiverse genus Peperomia
Source: Plant Biol (Stuttg). 2026 May 13;28(5):1373–84. doi: 10.1111/plb.70214 (PMC13358651; doi:10.1111/plb.70214)
Supplement: Supplementary file 6 — Table S10. References list. [file PLB-28-1373-s004.pdf]

1. Agnew, A.D.Q. 1974. Upland Kenya wild flowers: A flora of the ferns and herbaceous flowering plants of upland Kenya. Oxford University Press, Oxford, UK.
2. Alemayehu, T. 2006. Diversity and ecology of vascular epiphytes in Harenna afromontane forest, Bale, EthiopiaMSc-thesis, Addis Ababa University, Addis Ababa, Ethiopia.
3. Allen. *Peperomia hispidorhachis* 31 (MO).
4. Allen. *Peperomia rivi-vetusti* (syn. of *P. san-joseana*) 1360 (MO).
5. Allen. *Peperomia sarcodes* (syn. of *P. adscendens*) 1452 (MO).
6. Allen. *Peperomia albescens* (syn. of *P. silvivaga*) 1259 (MO).
7. Álvarez, H.R.A. 2009. Flora y vegetación ribereña de la cuenca del río Tocuyo, estados Lara y Falcón (Venezuela). Doctoral Thesis, Universidad de Alicante, Spain.
8. Alvaro Vindas, E. 2003. Common plants of Chirripó National Park, Costa Rica, 2nd ed. Instituto Nacional de Biodiversidad, Santo Domingo, Heredia, Costa Rica.
9. Alves, R.J.V. and J. Kolbek. 2009. Summit vascular flora of Serra de São José, Minas Gerais, Brazil. *Check List* 5: 35-73.
10. Andersson, N.J. 1855. Enumeratio plantarum in Insulis Galapagensibus - Piperaceae. *Kungliga Svenska vetenskapsakademiens handlingar* 4: 158-159.
11. Arantes, F.M., L.F.A. de Paula, and R.C. Forzza. 2024. Checklist of vascular plant species on inselbergs in the Monumento Natural dos Pontões Capixabas, Espírito Santo State, Brazil. *Biodiversity Data Journal* 12: e105688.
12. Backer, C.A. and R.C.B. van den Brink. 1963. Flora of Java: (Spermatophytes only). N.V.P. Noordhoff, Groningen, Netherlands.
13. Baden, H.M., et al. 2016. A botanical inventory of forest on karstic limestone and metamorphic substrate in the Chiquibul Forest, Belize, with focus on woody taxa. *Edinburgh Journal of Botany* 73: 39-81.
14. Badia, C.d.C.V., M.C.T.B. Messias, and L. Echternacht. 2021. Zooming in on quartzitic outcrops: micro-habitat influences on flora and vegetation. *Rodriguésia* 72: e01792019.
15. Balick, M.J., M. Nee, and D.E. Atha. 2000. Checklist of the vascular plants of Belize: with common names and uses. The New York Botanical Garden Press, New York, USA.
16. Balle, S. 1942. Revision des Piperaceae du Congo belge. *Bulletin du Jardin botanique de l'Etat, Bruxelles* 16: 367-405.
17. Bang. *Peperomia pseudoumbilicata* 2448 (NY).
18. Barbosa, D.E.F., et al. 2015. Vascular epiphytes in a remnant of seasonal semideciduous forest in the zona da Mata, state of Minas Gerais, Brazil. *Bioscience Journal* 31: 623-633.
19. Barthlott, W., et al. 2001. Diversity and abundance of vascular epiphytes: a comparison of secondary vegetation and primary montane rain forest in the Venezuelan Andes. *Plant Ecology* 152: 145-156.
20. Bataghin, F.A., et al. 2017. Epífitas vasculares da Estação Ecológica Barreiro Rico, Anhembi, SP, Brasil: diversidade, abundância e estratificação vertical. *Hoehnea* 44: 172-183.
21. Bernal, R., S.R. Gradstein, and M. Celis. 2015. Catalogue of the plants and lichens of Colombia website. Website http://catalogoplantasdecolombia.unal.edu.co/ 2015].
22. Bernier. *Peperomia bernieriana* 260 (P).
23. Biolley. *Peperomia nervosa* (syn. of *P. pseudocasarettoi*) 7433 (US).
24. Blum, C.T., C.V. Roderjan, and F. Galvao. 2011. Composição florística e distribuição altitudinal de epífitas vasculares da floresta ombrófila densa na Serra da Prata, Morretes, Paraná, Brasil. *Biota Neotropica* 11: 141-159.
25. Bongers, F., et al. 1988. Structure and floristic composition of the lowland rainforest of Las Tuxtlas, Mexico. *Vegetatio* 74: 55-80.
26. Bostock, P.D. and P.I. Forster. 1995. Distribution, habitat and conservation status of *Peperomia bellendenkerensis* (Piperaceae), a rare endemic from the 'Wet Tropics' of north-eastern Queensland. *Austrobaileya* 4: 449-450.
27. Brach, A.R. and H. Song. 2008. Flora of China. Website http://www.efloras.org/florataxon.aspx?flora_id=2&taxon_id=124469 2024].
28. Brade. *Peperomia barbensis* 2016 (BR).

29. Brako, L. and J.L. Zarucchi. 1993. Catalogue of the flowering plants and gymnosperms of Peru. 1 - 1251. Monographs in Systematic Botany from the Missouri Botanical Garden, St. Louis, Missouri, USA.
30. Brenan, J.P.M. and Collaborators. 1954. Plants collected by the Vernay Nyasaland Expedition of 1946. *Memoirs of the New York Botanical Garden* 9: 1-132.
31. Bridgewater, S.G.M., et al. 2006. A preliminary checklist of the vascular plants of the Chiquibul Forest, Belize. *Edinburgh Journal of Botany* 63: 269-321.
32. Britton, N.L. 1918. The flora of the American Virgin Islands. 19 - 118. Contributions from the New York Botanical Garden, New York, USA.
33. Britton, N.L. 1920. Descriptions of Cuban plants new to science. *Memoirs of the Torrey Botanical Club* 16: 57-118.
34. Britton, N.L. and C.F. Millspaugh. 1962. The Bahama Flora. 101. Hafner Publishing Company New York, USA.
35. Brown. *Peperomia septentrionalis* 428 (PH).
36. Brown, F. 1935. Flora of Southeastern Polynesia - *Peperomia*. *Bulletin of the Bernice Pauahi Bishop Museum, Honolulu, Hawaii* 130: 20-27.
37. Burger, W.C. 1971. Flora Costaricensis. Field Museum Press, Chicago, USA.
38. Burger, W.C. 1981. A new *Peperomia* from Nicaragua. *Phytologia* 48: 186-187.
39. Bussmann, R.W. 2001. Epiphyte diversity in a tropical Andean forest - Reserva Biológica San Francisco, Zamora-Chinchipe, Ecuador. *Ecotropica* 7: 43-60.
40. Bussmann, R.W. 2003. The vegetation of Reserva Biológica San Francisco, Zamora-Chinchipe, Southern Ecuador – a phytosociological synthesis. *Lyonia* 3: 145-308.
41. Callejas-Posada, R. and J. Betancur. 1997. Especies nuevas de Piperaceae de los Andes al sur de Colombia. *Novon* 7: 17-24.
42. Callejas-Posada, R. 2008. Piperaceae. *In* D.C. Daly and M. Silveria [eds.], Primeiro Catálogo da Flora do Acre, Brasil, 296-305. EDUFAC, Rio Branco, Brazil.
43. Callejas-Posada, R. 2020. Flora Mesoamericana: Piperaceae. vol. part 2, 590. Universidad Nacional Autónoma de México, Instituto de Biología, Mexico City, Mexico.
44. Cámara-Leret, R., et al. 2020. New Guinea has the world's richest island flora. *Nature* 584: 579-583.
45. Cárdenas. *Peperomia multifolia* 3089 (US).
46. Carvalho-Silva, M. and E.F. Guimarães. 2008. *Peperomia ciliato-caespitosa* M. Carvalho-Silva & E.F. Guim. (Piperaceae): A new species from Brazil. *Acta Botanica Brasilica* 22: 559-561.
47. Carvalho-Silva, M. and E.F. Guimarães. 2009. Piperaceae do Parque Nacional da Serra da Canastra, Minas Gerais, Brasil. *Boletim de Botânica da Universidade de São Paulo* 27: 235-245.
48. Carvalho-Silva, M., E.F. Guimarães, and E.V.S. Medeiros. 2013. Flora da Serra do Cipó, Minas Gerais: Piperaceae *Boletim de Botânica da Universidade de São Paulo* 31: 27-40.
49. Carvalho-Silva, M., E.F. Guimarães, and P.E.A.S. Câmara. 2013. New species of *Peperomia* (Piperaceae) from Brazil. *Systematic Botany* 38: 571-575
50. Carvalho-Silva, M., E.F. Guimaraes, and V.B. Sarnaglia Junior. 2019. Two new species of *Peperomia* Ruiz & Pavon (Piperaceae) from southeastern Brazil and four new synonymies. *Phytotaxa* 422: 225-232.
51. Carvalho-Silva, M., D. Monteiro, and G.M. Marcusso. continuously updated. *Peperomia* in Flora e Funga do Brasil. Rio de Janeiro Botanical Garden. Website https://floradobrasil.jbrj.gov.br/consulta/#CondicaoTaxonCP [accessed 20 March 2024].
52. Ceja-Romero, J., et al. 2010. The vascular epiphytic flora of the State of Hidalgo, Mexico diversity and distribution. *Acta Botánica Mexicana* 93: 1-39.
53. Cerví, A.C. and M. Borgo. 2007. Epífitos vasculares no Parque Nacional do Iguaçu, Paraná (Brasil). Levantamento preliminar *Fontqueria* 55: 415-422.
54. Chapman, J.D. and H.M. Chapman. 2001. The forests of Taraba and Adamawa States, Nigeria. An ecological account and plant species checklist. University of Canterbury, Christchurch, New Zealand.

55. Christophersen. *Peperomia christophersenii* 2286 (NY).
56. Cornejo-Tenorio, G., G. Ibarra-Manríquez, and S. Sinaca-Colín. 2019. Flora de Los Tuxtlas. Universidad Nacional Autónoma de México Mexico City, Mexico.
57. Correa, A.M.D., C. Galdames, and M.S. de Stapf. 2004. Catálogo de las plantas vasculares de Panamá. Universidad de Panamá & Instituto Smithsonian de Investigaciones Tropicales, Panama City, Panama.
58. Coyne, P. 2011. Norfolk Island's fascinating flora. 192. Petaurus Press, Belconnen, Australia.
59. Croat. *Peperomia armadana* 71409 (MO).
60. Croat, T.B. 1978. Flora of Barro Colorado Island. Stanford University Press, Stanford, California, USA.
61. Daba, D., et al. 2023. Modelling the current and future distribution potential areas of *Peperomia abyssinica* Miq., and Helichrysum citrispinum Steud. ex A. Rich. in Ethiopia. *Bmc Ecology and Evolution* 23.
62. Dahlstedt, H. 1900. Studien über Süd-und Central-Americkanische: Peperomien mit besonderer Berücksichtigung der Brasilianischen Sippen. vol. 2. Kongliga Svenska Vetenskaps-Akademiens handlingar, Stockholm, Sweden.
63. Dantas, T.S., P. Camara, and M. Carvalho-Silva. 2017. *Peperomia* (Piperaceae) from Trindade Island: A new species based on morphological and molecular data. *Systematic Botany* 42: 747-753.
64. De Candolle, C. 1894. Piperaceae Africanae et Madagascariensis. Beitrage zur flora von Afrika VIII. *In* A. Engler [ed.], Botanische Jahrbücher fur Systematik, Pflanzengeschichte und Pflanzengeographie, 224-230. Schweizerbart, Stuttgart, Germany.
65. De Candolle, C. 1898. Piperaceae Sodiroane - *Peperomia*. *Bulletin de l'Herbier Boissier* 6: 505-521.
66. De Candolle, C. 1898. Piperaceae novae - III Peperomieae. *Annuaire du Conservatoire et Jardin Botaniques de Genève* 2: 276-288.
67. De Candolle, C. 1901. Piperaceae et Meliaceae Brasiliensis. *Bulletin de l'Herbier Boissier* 1: 357-360.
68. De Candolle, C. 1902. Piperaceae. *In* B.L. Robinson [ed.], Flora of the Galapagos Islands 131. American Academy of Arts & Sciences, Boston, USA.
69. De Candolle, C. 1907. Plantae damazianae Brasiliensis - Piperacées. *Bulletin de l'Herbier Boissier* 7: 140-143.
70. De Candolle, C. 1910. Philippine Piperaceae. *Leaflets of Philippine botany* 3: 759-789.
71. De Candolle, C. 1911. Pipéracées de Madagascar, espèces et localités nouvelles. *Notulae Systematicae, Herbier du Museum de Paris* 2: 46-50.
72. De Candolle, C. 1912. Piperaceae. *In* I. Urban [ed.], Symbolae Antillanae,seu, Fundamenta florae Indiae Occidentalis, 185-190. Fratres Borntraeger ;Paul Klincksieck., Berlin, Germany; Paris, France.
73. De Candolle, C. 1913. The Hawaiian Peperomias. *College of Hawaii Publications Bulletin No. 2*: 759-789.
74. De Candolle, C. 1914. Piperaceae Meeboldianae herbarii Vratislaviensis. *Repertorium Specierum Novarum Regni Vegetabilis* 13: 297-298.
75. De Candolle, C. 1914. Plantae novae Bolivianae VI. - Piperaceae. *Repertorium Specierum Novarum Regni Vegetabilis* 13: 304-308.
76. De Candolle, C. 1914. Piperaceae novae - species africanae, sinensis, americanae. *Notulae Systematicae, Herbier du Museum de Paris* 3: 38-43.
77. De Candolle, C. 1917. Piperaceae Antillanae. *Repertorium Specierum Novarum Regni Vegetabilis* 15: 3-5.
78. De Candolle, C. 1920. Piperaceae novae. *Annuaire du Conservatoire et Jardin Botaniques de Genève* 21: 223-322.
79. De Candolle, C. 1921. Piperaceae novae e Micronesia et Polynesia allatae. *In* A. Engler [ed.], Botanische Jahrbücher für Systematik, Pflanzengeschichte und Pflanzengeographie, 502-506. Schweizerbart Stuttgart, Germany.

80. de Figueiredo, R.A. and M. Sazima. 2007. Phenology and pollination biology of eight *Peperomia* species (Piperaceae) in semideciduous forests in southeastern Brazil. *Plant Biology* 9: 136-141.
81. de Lange, P.J. 2020. *Peperomia leptostachya* (Piperaceae) on Raoul Island, Kermadec Islands—a name reinstated. *Trilepidea* 197: 1-10.
82. de Moura, C.O., et al. 2022. *Peperomia* (Piperaceae) endemic to Brazil: distribution, richness, and conservation status. *Flora* 297: 152170.
83. de Oliveira, S.M. 2024. Flora of the Guianas: Piperaceae. Website https://portal.cybertaxonomy.org/flora-guianas/node/9 2024].
84. de Queiroz, G.A., E.F. Guimarães, and A.A.M. de Barros. 2014. O gênero *Peperomia* Ruiz & Pav. (Piperaceae) na Serra da Tiririca, Rio de Janeiro, Brasil. *Acta Biológica Catarinense* 1: 5-14.
85. de Queiroz, G.A. and E.F. Guimarães. 2020. *Peperomia* (Piperaceae) no Leste Metropolitano do Rio de Janeiro, Brasil. *Rodriguesia* 71: e02512018.
86. de Stefano, R.D., G. Aymard, and O. Huber. 2007. Catálogo anotado e ilustrado de la flora vascular de los llanos de Venezuela. 619-621. FUDENA : Fundación Empresas Polar : FIBV, Caracas, Venezuela.
87. Dettke, G.A., A.C. Orfrini, and M.A. Milaneze-Gutierre. 2008. Composição florística e distribuição de epífitas vasculares em um remanescente alterado de floresta estacional semidecidual no Paraná, Brasil. *Rodriguésia* 59: 859-872.
88. Diniz, M.A. 1996. Piperaceae of the flora Zambesiaca area. *Kirkia* 16: 69-83.
89. Dodson, C.H., A.H. Gentry, and F.M. Valverde. 1986. Flora of Jauneche Los Rios Ecuador. *Selbyana* 8: 1-512.
90. Dorr, L.J. and S.M. Niño. 2024. Flora of guaramacal (Venezuela) II: Gymnosperms, angiosperms (chloranthales, magnoliids). Smithsonian Institute Scholarly Press, Washington, USA.
91. Düll, R. 1973. Die Peperomia-arten Afrikas. *Botanische Jahrbücher für Systematik, Pflanzengeschichte und Pflanzengeographie* 93: 56-129.
92. Duss. *Peperomia subbracteiflora* (syn. of *P. hirtella*) 4107 (NY).
93. Duss. *Peperomia broadwayi* (syn. of *P. myrtifolia*) 1262 (US).
94. Eggers. *Peperomia monteverdensis* (syn. of *P. guadaloupensis*) 5127 (NY).
95. Eggers. *Peperomia papillosa* 5289 (B).
96. Ekman. *Peperomia gloriosifolia* H526 (B).
97. Ekman. *Peperomia moncionis* (syn. of *P. nizaitoensis*) H12886 (S).
98. Ekman. *Peperomia ocoana* H11861 (S).
99. Ekman. *Peperomia perherbaceae* (syn. of *P. petiolata*) H5052 (S).
100. Ekman. *Peperomia productamenta* H5773 (S).
101. Ekman. *Peperomia pullispica* H9832b (ILL).
102. Ekman. *Peperomia ramosa* (syn. of *P. humilis*) H1575 (S).
103. Ekman. *Peperomia semidecurrens* (syn. of *P. erytrhopremna*) 16740 (S).
104. Ekman. *Peperomia siguaneana* (syn. of *P. hirta*) 16890 (S).
105. Ekman. *Peperomia taco-tacoi* (syn. of *P. petiolaris*) 17620 (S).
106. Ekman. *Peperomia yaquena* (syn. of *P. quadrifolia*) H13689 (S).
107. Ekman. *Peperomia amphioxys* (syn. of *P. nizaitoensis*) 11133 (B).
108. Ekman. *Peperomia cabaiana* (syn. of *P. quadrifolia*) H1590 (B).
109. Engler, A. 1899. Piperaceae africanae III. *Botanische Jahrbücher fur Systematik* 26: 361.
110. Espejo-Serna, A., et al. 2021. Mexican vascular epiphytes: Richness and distribution. *Phytotaxa* 503: 1-124.
111. Espinosa. *Peperomia persucculenta* 2283 (NY).
112. Fawcett, W. and A.B. Rendle. 1914. Flora of Jamaica, containing descriptions of the flowering plants known from the island: Dicotyledons. 1 -280. Trustees of the British Museum, London, UK.

113. Ferreira, V.L. and J.R. Stehmann. 2023. Saxicolous vascular flora of karst outcrops: An overlooked component of Brazilian biodiversity. *Flora* 305: 152314.
114. Fischer, E., et al. 2010. Annotated checklist of the vascular plants of Kakamega Forest, Western Province, Kenya. *Journal of East African Natural History* 99: 129-226.
115. Florence, J. 1997. Flore de la Polynésie française. 393. Institut de recherche pour le développement, Paris, France.
116. Fondom, N.Y., S. Castro-Nava, and A.J. Huerta. 2009. Seasonal variation in photosynthesis and diel carbon balance under natural conditions in two *Peperomia* species that differ with respect to leaf anatomy. *Journal of the Torrey Botanical Society* 136: 57-69.
117. Forster. *Peperomia hunteriana* 21777 (MEL).
118. Fosberg, F.R. and M.H. Sachet. 1975. Flora of Micronesia: Casuarinaceae, Piperaceae, and Myricaceae - Volume 2 vol. 2, 28. Smithsonian Institute Scholarly Press, Washington, USA.
119. Foster, P.I. 1993. A taxonomic revision of the genus *Peperomia* Ruiz & Pav. (Piperaceae) in mainland Australia. *Austrobaileya* 4: 93-104.
120. Francisco, T.M., et al. 2023. Inselbergs from Brazilian Atlantic Forest: high biodiversity refuges of vascular epiphytes from Espirito Santo. *Biodiversity and Conservation* 32: 2561–2584.
121. Franquemont, C., et al. 1990. The ethnobotany of Chinchero, an Andean community in southern Peru. *Fieldiana* 24: 1-126.
122. Fries, R.E. and T.C. Fries. 1929. Beiträge zur Kenntnis der Flora des Kenia, Mt. Aberdare und Mt. Elgon XII. *Notizblatt des Botanischen Gartens und Museums zu Berlin-Dahlem*: 594-621.
123. Friis, I. and K. Vollesen. 1998. Flora of the Sudan-Uganda border area east of the Nile, I: Catalogue of vascular plants, 1st part. Biologiske Skrifter. The Royal Danish Academy of Sciences and Letters, Copenhagen, Denmark.
124. Galeotti. *Peperomia monticola* 6023 (U).
125. Gamble, J.S. 1967. Flora of the Presidency of Madras. vol. 2. Botanical Survey of India, Kolkata, India.
126. Garber. *Peperomia tutuilana* 863 (BISH).
127. Garcia-Martinez, R., et al. 2018. Two new lithophytic species of the genus *Peperomia* (Piperaceae) from the state of Chiapas, Mexico. *Phytotaxa* 338: 109-116.
128. Gatti, A.L.S. 2000. O componente epifítico vascular na reserva natural Salto Morato, Guaraqueçaba - PR. Msc. thesis, Universidade Federal do Paraná.
129. González, F. and N. Pabón-Mora. 2022. Floristic novelties for the Sanctuary of Fauna and Flora of Iguaque and its areas of influence as the sole protected area of the Ricaurte Province (Boyaca, Colombia). *Caldasia* 44: 260 - 273.
130. Goodwin, Z.A., et al. 2013. A checklist of the vascular plants of the lowland savannas of Belize, Central America. *Phytotaxa* 101: 1-119.
131. Görts-van Rijn, A.R.A. 1998. *Peperomia gracieana* (Piperaceae), a new species from French Guiana. *Brittonia* 50: 56-58.
132. Grayum, M.H. 1995. Notes on Costa Rican *Peperomia* (Piperaceae), including four new species. *Phytologia* 79: 108-113.
133. Green, P.S. 1994. Piperaceae. *In* A.E. Orchard and A.J.G. Wilson [eds.], Flora of Australia, Oceanic islands 1, 46-50. Australian Government Publishing Service, Canberra, Australia.
134. Grisebach, A.H.R. 1864. Flora of the British West Indian Islands. 789. Lovell Reeve & Company, London, UK.
135. Guimarães, E.F. and C.G. Costa. 1980. Notas sobre Piperaceae. Novos sinônimos. *Rodriguésia* 32: 7-14.
136. Guimarães, E.F. and L.C.d.S. Giordano. 2004. Piperaceae do Nordeste brasileiro I: estado do Ceará. *Rodriguésia* 55: 21-46.
137. Guimarães, E.F., et al. 2023. *Piperaceae Raddianae*: A taxonomic and nomenclatural study of Giuseppe Raddi's Brazilian Piperaceae. *Taxon* 72: 880-893.
138. Hargreaves, B.J. 1979. Succulents of Malaŵi. *The Society of Malawi Journal* 32: 31-44.
139. Harling. *Peperomia harlingii* 3609 (S).

140. Harris. *Peperomia penicillata* 8315 (NY).
141. Harrison. *Peperomia hanaensis* 575A (BISH).
142. Hasler. *Peperomia saxigaudens* (syn. of *P. increscens*) 3080 (K).
143. Heads, M. 2006. Seed plants of Fiji: An ecological analysis. *Biological Journal of the Linnean Society* 89: 407-431.
144. Hedberg, I. and S. Edwards. 1989. Flora of Ethiopia & Eritrea Part 1. Magnoliaceae to Flacourtiaceae. 532. Addis Ababa University Uppsala, Sweden, Uppsala, Sweden.
145. Herrera. *Peperomia ppucu-ppucu* 802 (US).
146. Herter. *Peperomia tacuariana* (syn of. *P. tetraphylla*) 2004 (G).
147. Hill, A.W. 1907. A revision of the geophilous species of *Peperomia*, with some additional notes on their morphology and seedling structure. *Annals of Botany* 21: 141-160.
148. Hillebrand, W. 1888. Flora of the Hawaiian Islands. Williams & Norgate, London, UK.
149. History, N.M.o.N. 2024. Department of Botany Collections. Website https://collections.nmnh.si.edu/search/botany/ 2024].
150. Hokche, O., P.E. Berry, and O. Huber. 2008. Nuevo catálogo de la flora vascular de Venezuela. Fundación Instituto Botánico de Venezuela, Caracas, Venezuela.
151. Hosokawa. *Peperomia pacificola* 6654 (TAI).
152. Howard, R.A. 1962. Botanical and other observations on Redonda, the West Indies. *Journal of the Arnold Arboretum* 43: 51-66.
153. Howard, R.A. 1988. Flora of the Lesser Antilles: Leeward and windward islands: Dicotyledoneae - Part 1. vol. 4, 14-24. Arnold Arboretum of Harvard University, Boston, USA.
154. Hoyos, J. 1985. Flora de la Isla Margarita, Venezuela. Sociedad y Fundacion La Salle de Ciencias Naturales, Caracas, Venezuela.
155. Huber, H.F.J. 1987. A revised handbook to the flora of Ceylon: Piperaceae. vol. 6, 272 - 300. Amerind Publishing Company, New Delhi, India
156. Ichaso, C. and E. Guimarães. 1984. Piperaceae do Município do Rio de Janeiro - II. *Peperomia* Ruiz et Pavon. *Rodriguésia* 36: 47-60.
157. Jimeno-Sevilla, H.D., et al. 2018. Five endemic *Peperomia* (Piperaceae) novelties from Veracruz, Mexico. *Phytotaxa* 369: 93-106.
158. Jørgensen, P.M. and S. León-Yánez. 1999. Catalogue of the vascular plants of Ecuador. Monographs in Systematic Botany from the Missouri Botanical Garden, St. Louis, Missouri, USA.
159. Julia, S. and R. Kiew. 2023. The biodiversity of Bukit Anak Takun, Selangor, Malaysia, with emphasis on the flora. *Malayan Nature Journal* 75: 425-440.
160. Junior, V.B.S., et al. 2015. New records of *Peperomia armondii* Yunck, *Peperomia hispidula* (Sw.) A. Dietr., and *Peperomia mandioccana* Miq. for the state of Espírito Santo, southeastern Brazil. *Check List* 11: 1-5.
161. Keller, H. and S. Tressens. 2005. Novedades en *Peperomia* (Piperaceae) para la Argentina: con una clave para las especies de Misiones. *Boletín de la Sociedad Argentina de Botánica* 40: 297-306.
162. Killip. *Peperomia immolata* 17100 (NY).
163. Killip. *Peperomia machaerodonta* (syn. of *P. alpina*) 3538 (US).
164. Killip. *Peperomia pennellii* 20049 (US).
165. Killip. *Peperomia pubinervosa* 25269 (US).
166. Killip. *Peperomia variculata* 26792 (US).
167. Kirby, C. 2014. Field guide to New Zealand's epiphytes, vines & mistletoes Environmental Research Institute, University of Waikato, Hamilton, New Zealand.
168. Koorders, S.H. 1908. Die Piperaceae von Java. *Verhandelingen der Koninklijke Akademie van Wetenschappen te Amsterdam* 14: 59-71.
169. Lewis. *Peperomia yungasana* 88977 (LPB).
170. Liebmann. *Peperomia oajacensis* (syn. of *P. leptophylla*) 127 (C).
171. Liogier, H.A. 1985. Descriptive flora of Puerto Rico and adjacent Islands: Spermatophyta, 1 ed., vol. 1. University of Puerto Rico Press, San Juan, Puerto Rico.

172. Liogier, A.H. and L.F. Martorell. 2000. Flora of Puerto Rico and adjacent islands: a systematic synopsis. Editorial de la Universidad de Puerto Rico, San Juan, Puerto Rico.
173. Little, E.L., R.O. Woodbury, and F.H. Wadsworth. 1976. Flora of Virgin Gorda (British Virgin Islands). Forest Service, US Department of Agriculture Rio Piedras, Puerto Rico.
174. Liu, T.-S. and F.-L. Wang. 1976. Piperaceae, Flora of Taiwan. Vol. 2, 556-559. Tah Jinn Printing Company, Taipei, Taiwan.
175. Lourenço, J. and M. Carvalho-Silva. 2014. Piperaceae do Parque Nacional da Chapada dos Veadeiros, Goiás, Brasil. *Heringeriana* 5: 11-18.
176. Lundell, C.L. 1937. The vegetation of Petén. With an appendix: Studies of Mexican and Central American plants - I. Carnegie Institution of Washington, Washington, USA.
177. Macbride. *Peperomia longispica* (syn. of *P. galioides*) 3928 (BM).
178. Macbride, J.F. 1936. Flora of Peru: Part 2. Field Museum Press, Chicago, USA.
179. Machado-Silva, T., M. Carvalho-Silva, and L.G. Temponi. 2020. *Peperomia* (Piperaceae) no Parque Estadual de Vila Velha, Paraná. *Rodriguésia* 71: e00982018.
180. Madsen. *Peperomia graveolens* 87044 (AAU).
181. Maguire, B. and J.J. Wurdack. 1957. The botany of the Guyana Highland - Part II. *Memoirs of the New York Botanical Garden*: 235-392.
182. Maguire, B., J.A. Steyermark, and J.J. Wurdack. 1957. Botany of the Chimanta Massif - I Gran Sabana, Venezuela. *Memoirs of the New York Botanical Garden* 9: 393-440.
183. Mai, P., et al. 2016. Taxonomic revision of *Peperomia* (Piperaceae) from Uruguay. *Phytotaxa* 244: 125–144.
184. Mai, P., et al. 2019. Catalogue of the vascular epiphytic flora of Uruguay. *Acta Botanica Brasílica* 33: 683-708.
185. Mandon. *Peperomia saxicola* 1118 (P).
186. Mandon. *Peperomia boliviensis* 1121 (K).
187. Marcusso, G., P.H. Melo, and J. Lombardi. 2020. *Peperomia calcicola* (Piperaceae), a new species from limestone outcrops of the southeastern Brazilian Atlantic Forest. *Phytotaxa* 446: 245–252.
188. Marcusso, G.M., H.L. Ribeiro, and J.A. Lombardi. 2020. *Peperomia crypticola* C. DC. (Piperaceae): rediscovery and emended description of a rare species of southeastern Brazil. *Feddes Repertorium* 131: 46-50.
189. Marcusso, G.M., et al. 2024. Taxonomic notes and a new country record for *Peperomia* subgenus *Pseudocupula* (Piperaceae) in Brazil. *Phytotaxa* 635: 227-237.
190. Marcusso, G.M., P.H.A. De Melo, and R.C. Forzza. 2024. Peperomia dryadica (Piperaceae), a new karstic species from the Brazilian Atlantic Forest. *Kew Bulletin*.
191. Vasquez Martinez, R., R. Rojas Gonzáles, and H. van der Werff. 2010. Flora del Río Cenepa, Amazonas, Perú: Volume 1. Monographs in Systematic Botany from the Missouri Botanical Garden, St. Louis, Missouri, USA.
192. Mathew, J., et al. 2018. Two new species of *Peperomia* Ruiz & Pav. (Piperaceae) from South Western Ghats, India. *Species* 19: 162-166.
193. Mathieu et al. *Peperomia congesta* 2009-047 (BR, GENT, USM).
194. Mathieu et al. *Peperomia espinosae* 2009-051 (BR, GENT, MO, USM).
195. Mathieu et al. *Peperomia ferreyrae* 2009-091 (BR, GENT, USM).
196. Mathieu et al. *Peperomia columella* 2009-105 (BR, GENT, USM).
197. Mathieu et al. *Peperomia conjugata* 2009-147 (BR, G, GENT, K, MO, P, UC, US, USM).
198. Mathieu, G. 2001-2023. Peperomia bibliography online. Website www.peperomia.net [accessed 20 December 2024].
199. Mathieu, G. 2003. New endemic *Peperomia* species (Piperaceae) from Madagascar. *Systematics and Geography of Plants* 73: 71-81.
200. Mathieu, G. 2003. *Peperomia nicolliae*, a new endemic species (Piperaceae) from Madagascar. *Systematics and Geography of Plants* 73: 288-290.
201. Mathieu, G. 2006. More new *Peperomia* species (Piperaceae) from Madagascar. *Systematics and Geography of Plants* 76: 221-228.

202. Mathieu, G. 2008. Three new creeping *Peperomia* species (Piperaceae) from Ecuador. *Novon* 18: 80-85.
203. Mathieu, G., et al. 2008. Taxonomy of the *Peperomia* species (Piperaceae) with pseudo-epiphyllous inflorescences, including four new species. *Botanical Journal of the Linnean Society* 157: 177-196.
204. Mathieu, G., et al. 2011. New geophytic *Peperomia* (Piperaceae) species from Mexico, Belize and Costa Rica. *Revista Mexicana de Biodiversidad* 82: 357-382.
205. Mathieu, G., et al. 2015. *Peperomia* (Piperaceae) novelties from Veracruz State, Mexico. *Phytotaxa* 205: 268-276.
206. Mathieu, G. 2017. *Peperomia tubericordata* (Piperaceae), a new geophytic species from Oaxaca, Mexico. *Phytotaxa* 313: 293-295.
207. Mathieu, G. 2018. Notes on 'rabbit tail' Peperomias (Piperaceae) with description of two new species. *Candollea* 73: 209-215.
208. Mathieu, G. 2020. Endemic *Peperomia* (Piperaceae) novelties from eastern Madagascar. *Candollea* 75: 75-82.
209. Mathieu, G. and J. Viccon-Esquivel. 2020. Two new *Peperomia* species (Piperaceae) from southwest Mexico. *Phytotaxa* 458: 69-76.
210. Mathieu, G. 2024. New Mexican *Peperomia* species in subgenus *Panicularia* (Piperaceae). *Phytotaxa* 636: 229-237.
211. Maxon. *Peperomia glabrior* 5401 (US).
212. Maxon. *Peperomia maxonii* 4043 (NY).
213. Meave, J.A., et al. 2017. Checklist of the vascular flora of a portion of the hyper-humid region of La Chinantla, Northern Oaxaca Range, Mexico. *Botanical Sciences* 95: 722-759.
214. Melo, A., E.F. Guimarães, and M. Alves. 2016. Synopsis of the genus *Peperomia* Ruiz & Pav. (Piperaceae) in Roraima State, Brazil. *Hoehnea* 43: 119-134.
215. Millspaugh, C.F. 1895. Contribution II - to the coastal and plain flora of Yucatan. 293. Field Columbian Museum Chicago, USA.
216. Millspaugh, C.F. 1902. Flora of the Island of St. Croix. *Publications of the Field Columbian Museum, Botanical Series* 1.
217. Miquel, F.A.W. 1843. Systema Piperacearum. 64-199. Apud H. A. Kramers, Rotterdam, Netherlands.
218. Miquel, F.A.W. 1847. Mantissa Piperacearum *Linnaea* 20: 118-129.
219. Monro, A.K., et al. 2017. A first checklist to the vascular plants of La Amistad International Park (PILA), Costa Rica-Panama. *Phytotaxa* 322: 1-283.
220. Monteiro, D. and E.F. Guimarães. 2008. Flora do Parque Nacional do Itatiaia-Brasil: *Peperomia* (Piperaceae). *Rodriguésia* 59: 161-195.
221. Monteiro, D. 2013. Piperaceae em um fragmento de floresta atlântica da Serra da Mantiqueira, Minas Gerais, Brasil. *Rodriguésia* 64: 379-398.
222. Monteiro, D., P. Leitman, and M.M. Coelho. 2016. Two new *Peperomia* (Piperaceae) species from Southern Bahia, Brazil. *Phytotaxa* 258: 287-294.
223. Monteiro, D. 2018. Flora of the canga of the Serra dos Carajás, Pará, Brazil: Piperaceae. *Rodriguésia* 69: 1285-1309.
224. Monteiro, D. and E.F. Guimarães. 2020. Piperaceae do nordeste brasileiro II: estado de Alagoas. *Rodriguesia* 71: e03122018.
225. Moore. *Peperomia raiateensis* (syn. of *P. societatis*) 352 (P).
226. Moran. *Peperomia guamana* 4540 (MO).
227. Mori, S.A., et al. 2002. Guide to the vascular plants of Central French Guiana - Dicotyledons. New York Botanical Garden Press, New York, USA.
228. Moura. *Peperomia mourae* (syn. of *P. megapotamica*) 989 (B).
229. Nash. *Peperomia infravillosa* 769 (NY).
230. Nash. *Peperomia maleuvreana* (syn. of *P. glabella*) 1119 (US).
231. Nash. *Peperomia perinduta* (syn. of *P. foraminum*) 1487 (B).

232. Nicolson, D.H. 1991. Flora of Dominica, Part 2: Dicotyledoneae. vol. 77. Smithsonian Institute Scholarly Press, Washington, USA.
233. Nieder, J. and W. Barthlott. 2001. The flora of the Río Guajalito mountain rain forest (Ecuador). Results of the Bonn - Quito epiphyte project, funded by the Volkswagen Foundation (Vol. 1 of 2). Books on Demand, Bonn, Germany.
234. Oliver, W.R.B. 1930. New Zealand epiphytes. *Journal of Ecology* 18: 1-50.
235. Osten. *Peperomia misionense* (syn of. *P. arifolia*) 8096 (LIL).
236. Paniagua-Zambrana, N.Y., R.W. Bussmann, and C. Romero. 2020. *Peperomia* alata Ruiz & Pav. *Peperomia blanda* (Jacq.) Kunth *Peperomia fraseri* C. DC. *Peperomia galioides* Kunth *Peperomia hartwegiana* Miq. *Peperomia inaequalifolia* Ruiz & Pav. *Peperomia quadrifolia* (L.) Kunth Piperaceae. *In* N. Paniagua-Zambrana, Bussmann, R. [ed.], Ethnobotany of the Andes, 1391–1403. Springer, Cham, Germany.
237. Parodi, D. 1878. Contribuciones à la Flora del Paraguay. *Anales de la Sociedad Científica Argentina* 6: 93-95.
238. Pelser, P.B. 2023. Piperaceae. Website https://www.philippineplants.org/Families/Piperaceae.html [accessed 12 March 2024].
239. Pennell. *Peperomia praeruptorum* 1433 (PH).
240. Pennell. *Peperomia quindioensis* 10082 (US).
241. Pino. *Peperomia lanuginosa* 1215 (USM).
242. Pino, G., O. Klopfenstein, and N. Cieza. 2003. Three new taxa of *Peperomia* from northern Peru. *Cactus and Succulent Journal* 75: 27-35.
243. Pino, G., O. Klopfenstein, and N. Cieza. 2004. Four new taxa of *Peperomia* (Piperaceae) from San Marcos, northern Peru. *Haseltonia* 10: 87-95.
244. Pino, G., O. Klopfenstein, and N. Cieza. 2005. Four new Peperomias from northern Peru. *Haseltonia* 11: 103-112.
245. Pino, G. 2008. Three new succulent peperomias from Perú. *Cactus and Succulent Journal* 80: 232-239.
246. Pino, G. 2010. Alte und neue Namen der sukkulenten Arznei-Peperomias aus Peru. *Avonia* 28: 39-55.
247. Pino, G., et al. 2012. New succulent window-leaved Peperomias from Peru. *Haseltonia* 18: 3-26.
248. Pino, G., R. Fernández, and M.-S. Samain. 2021. Succulent Peruvian species of *Peperomia* subgenus *Panicularia*. *Haseltonia* 27: 116-129.
249. Pino, G., et al. 2022. Two new geophytic species of *Peperomia* subgenus *Tildenia* (Piperaceae) from Central Peru. *Haseltonia* 29: 27-35.
250. Pino, G. and M.-S. Samain. 2022. *Peperomia ostolazae*, a new species from the department of Apurimac, Peru. *Revista Quero Saber* 35: 19-39.
251. Pino, G., et al. 2023. Species of *Peperomia* (Piperaceae) from the Saña River Valley, Peru. *Phytokeys* 225: 1-40.
252. Piovesani, W.S. and P. Brack. 2024. Lista atualizada e aspectos sobre a conservação da flora vascular do Parque Estadual do Turvo, Rio Grande do Sul, Brasil. *Iheringia* 79.
253. Pittier. *Peperomia gatunensis* 2280 (US).
254. Pittier. *Peperomia oblongibacca* (syn. of *P. macrostachyos*) 3891 (US).
255. POWO. 2024. Plants of the World Online. Royal Botanic Gardens. Website http://www.plantsoftheworldonline.org/ [accessed 20 December 2024].
256. Preuss. *Peperomia preussii* (syn. of *P. fernandopoiana*) 863 (B).
257. Pringle. *Peperomia macrandra* 4654 (GH).
258. Proctor. *Peperomia proctorii* 11038 (NY).
259. Proctor, G.R. 2012. Flora of the Cayman Islands. Royal botanic Garden, Kew, London, UK.
260. Pujals, J.L.R.R. and R.P. Velez. 2010. Sinopsis anotada y comentada de la flora del bosque estatal de Maricao. Forest Service, U.S. Department of Agriculture, United States.
261. Pulle, A. and J. Lanjouw. 1968. Flora of Suriname vol. I, part 2. 293-441. The Royal Tropical Institute, Amsterdam, Netherlands.

262. Radh, S.S. and S. Nampy. 2018. *Peperomia ekakesara*: A new species of Piperaceae from Mathikettan Shola National Park, Kerala, India. *Phytotaxa* 364: 283-288.
263. Rai, P. and G. Mathieu. 2023. *Peperomia mangalbaria* (Piperaceae), a new species from Sikkim Himalaya, India. *Phytotaxa* 609: 138-144.
264. Rao, S.K., et al. 2019. Flora of Peninsular India. Website https://indiaflora-ces.iisc.ac.in/FloraPeninsular/herbsheet.php?id=7060&cat=7 2024].
265. Rauh, W. and W. Barthlott. 1975. Two new species of succulent Peperomias from South America: *Peperomia graveolens* (Ecuador) & *Peperomia asperula* (Peru). *Cactus and Succulent Journal* 47: 198-208.
266. Rechinger. *Peperomia biformis* 1873 (W).
267. Rechinger. *Peperomia lonchophylloides* 138 (W).
268. Rechinger. *Peperomia novemnervia* 1576 (W).
269. Rechinger. *Peperomia rechingerae* 380 (no herbarium data).
270. Rendle, A.B. 1908. Plants collected on Mt. Ruwenzori. *Journal of the Linnean Society* 38: 278.
271. Ribeiro, J.E.L. 1999. Flora da Reserva Ducke: Guia de identificacão das plantas vasculares de uma floresta de terra-firme na Amazônia Central. Instituto Nacional de Pesquisas da Amazônia - INPA, Manaus, Brazil.
272. Ridley, H.N. 1924. The Flora of the Malay Peninsula. L. Reeve & Company, London, UK.
273. Rodrigues, L.C.G., G.A. de Queiroz, and E.F. Guimarães. 2021. *Peperomia* Ruiz & Pav. (Piperaceae) da Reserva Biológica do Tinguá, RJ, Brasil. *Hoehnea* 48: e382020.
274. Rose. *Peperomia painteri* 8966 (US).
275. Rossetto, E.F.S. and A.O.S. Vieira. 2013. Vascular flora of the Mata dos Godoy State Park, Londrina, Paraná, Brazil. *Check List* 9: 1020-1034.
276. Rudas Lleras, A.n. and A. Prieto Cruz. 2005. Flórula del Parque Nacional Natural Amacayacu, Amazonas, Colombia. Monographs in Systematic Botany from the Missouri Botanical Garden, St. Louis, Missouri, USA.
277. Samain et al. *Peperomia verschaffeltii* 2009-139 (BR, GENT, USM).
278. Samain et al. *Peperomia berlandieri* 2007-004 (BR, GENT, MEXU).
279. Samain et al. *Peperomia hintonii* 2007-008 (BR, GENT, MEXU).
280. Samain et al. *Peperomia urocarpoides* 2007-066 (BR, GENT, MEXU).
281. Samain et al. *Peperomia consoquitlana* 2007-113 (BR, GENT, MEXU).
282. Samain et al. *Peperomia pruinosifolia* 2009-111 (BR, GENT, USM).
283. Samain et al. *Peperomia foliiflora* 2009-168 (GENT, USM).
284. Samain et al. *Peperomia juniniana* 2009-171 (GENT, USM).
285. Samain et al. *Peperomia choroniana* 2009-174 (BR, GENT, USM).
286. Samain et al. *Peperomia naviculifolia* 2009-211 (GENT, USM).
287. Samain et al. *Peperomia procumbens* 2009-232 (BR, GENT, USM).
288. Samain et al. *Peperomia areolata* 2009-233 (GENT, USM).
289. Samain. *Peperomia cavispicata* 2008-153 (GENT).
290. Samain and Bates. *Peperomia aceramarcana* 2008-003 (BR, GENT, LPB).
291. Samain and Bates. *Peperomia pachydermis* 2008-148 (BR, LPB).
292. Samain, M.S., et al. 2011. The geophytic *Peperomia* subgenus *Tildenia* (Piperaceae) in the Andes with the description of new species in a phylogenetic framework. *Plant Ecology and Evolution* 144: 148-176.
293. Samain, M.-S. and M. Tebbs. 2020. Flora del Bajío y de regiones adyacentes: familia Piperaceae. *Fascículo* 215: 1-62.
294. Saralegui, B. 2004. Flora de la República de Cuba: Piperaceae. *Fascículo* 9: 94.
295. Sarasin. *Peperomia canalensis* 265 (Z).
296. Sarasin. *Peperomia kanalensis* 265 (Z).
297. Sarasin. *Peperomia lifuana* 824 (Z).
298. Sarasin. *Peperomia sarasinii* 501 (Z).
299. Scheiris et al. *Peperomia phyllantha* 015 (GENT, HOXA, MO).
300. Schiede. *Peperomia schiedeana* (syn. of *P. tenerrima*) s.n. (MO).

301. Schlechter. *Peperomia microstachya* 16248 (G).
302. Schroeder, L.J. 1926. Data of certain American Piperaceae. *Candollea* 3: 121-140.
303. Setchell. *Peperomia setchelii* (syn. of *P. hombronii*) 502 (UC).
304. Shäfer. *Peperomia guanensis* 10541 (NY).
305. Silverstone-Sopkin, P.A. 2011. A new Species of *Peperomia* (Piperaceae) from the Cauca Valley, Colombia. *Novon: A Journal for Botanical Nomenclature* 21: 266-269.
306. Skottsberg, C. 1921. The phanerogams of the Juan Fernandez Islands. *In* C. Skottsberg [ed.], The Natural History of Juan Fernandez and Easter Island, 95 - 240. Almqvist & Wiksells Boktryckeri, Uppsala, Sweden.
307. Smith, A.C. 1979. Flora Vitiensis nova : a new Flora of Fiji (Spermatophytes only). Pacific Tropical Botanical Garden, Lawaii, Hawaii.
308. Sodiro, R.P.L. 1900. Piperaceas Ecuatorianas. *Contributiones de la Flora Ecuatoriana - Monografia* 1: 64-145.
309. Solomon et al. *Peperomia yungasana* 19022 (LPB).
310. Solomon. *Peperomia adenocarpa* 18082 (MO).
311. Solomon. *Peperomia procumbens* 3362 (MO).
312. Souza, L.A., I.S. Moscheta, and J.H.G. Oliveira. 2004. Comparative morphology and anatomy of the leaf and stem of *Peperomia dahlstedtii* C.DC., *Ottonia martiana* Miq. and *Piper diospyrifolium* Kunth (Piperaceae). *Gayana Botanica* 61: 6-17.
313. St. John. *Peperomia nahikuensis* (syn. of *P. eekana*) 17963 (BISH).
314. St. John. *Peperomia raivavaeana* (syn. of *P. australana*) 16160 (NY).
315. St. John. *Peperomia rotumaensis* 19096 (US).
316. St. John. *Peperomia rurutana* (syn. of *P. australana*) 16729 (NY).
317. Standley. *Peperomia multifida* (syn. of *P. hylophila*) 34349 (US).
318. Standley. *Peperomia orientalis* (syn. of *P. naranjoana*) 36943 (US).
319. Standley. *Peperomia pilulifera* 37439 (US).
320. Standley. *Peperomia pothifolia* (syn. of *P. striata*) 37427 (US).
321. Standley. *Peperomia punctatifolia* (syn. of *P. matlalucaensis*) 37968 (US).
322. Standley. *Peperomia stenophyllopsis* (syn. of *P. angularis*) 47590 (US).
323. Standley. *Peperomia breviscapa* (syn. of *P. pernambucensis*) 36888 (US).
324. Standley, P.C. 1930. Studies of American plants - III. *Publications of the Field Museum of Natural History* 8: 5.
325. Standley, P.C. 1931. Flora of the Lancetilla Valley, Honduras. Field Museum of Natural History Botanical Series, Chicago, USA.
326. Standley, P.C. 1937. Flora of Costa Rica Part 1. Field Museum of Natural History Botanical Series, Chicago, USA.
327. Standley, P.C. 1940. Studies of American plants - IX. *Publications of the Field Museum of Natural History* 22: 8-10.
328. Standley, P.C. and J.A. Steyermark. 1952. Flora of Guatemala - Part III, Piperaceae. *Fieldiana Botany, Chicago* 24: 228-337.
329. Stehlé. *Peperomia stehleana* (syn. of *P. nigropunctata*) 25 (NY).
330. Stehlé, H. 1939. Notes sur la répartition et l’écologie de dicotylédones nouvelles ou rares de la Martinique (5e contribution). *Bulletin de la Société Botanique de France* 85: 576-579.
331. Stehlé, H. 1940. Flore descriptive des Antilles françaises Tome 2 : Les Pipérales des Antilles françaises. Etude descriptive et écologique des Pipéracées et Chlorantacées. 119-144. Imprimerie Officielle de la Martinique, Fort-De-France, Martinique.
332. Stehlé, H. 1941. Les Pipérales des Antilles Françaises - *Peperomia*. *Bulletin Agricole de la Martinique, nouvelle. série* 9: 196-221.
333. Stehlé, H. 1946. Piperaceae ex Insulis Caribaeis. *Candollea* 10: 288-292.
334. Stehlé, H. 1948. Flore de la Guadeloupe et dépendances et de la Martinique. 39-63. Imprimerie Clément Brunel, Montpellier, France.
335. Stevens, W.D., et al. 2001. Flora de Nicaragua. Monographs in Systematic Botany from the Missouri Botanical Garden, St. Louis, Missouri, USA.

336. Steyermark. *Peperomia nilssonii* (syn. of *P. alata*) 523 (NY).
337. Steyermark, J.A. 1966. Botanical novelties in the region of Sierra de Lema, Estado Bolivar, Venezuela III. *Boletin de la Sociedad Venezolana de Ciencias Naturales* 26: 412-413.
338. Steyermark, J.A. and O. Huber. 1978. Flora del Avila. Sociedad Venezolana de Ciencias Naturales, Caracas, Venezuela.
339. Steyermark, J.A. 1986. A new species of *Peperomia* (Piperaceae) from Venezuela. *Brittonia* 38: 220-221.
340. Suwanphakdee, C., T.R. Hodkinson, and P. Chantaranothai. 2014. New records of *Peperomia* (Piperaceae) in Thailand. *Thai Forest Bulletin (Botany)* 42: 61-67.
341. Suwanphakdee, C., T.R. Hodkinson, and P. Chantaranothai. 2017. New species and a reinstatement in *Peperomia* (Piperaceae) from Thailand. *Kew Bulletin* 72: 1-15.
342. Suwanphakdee, C., T.R. Hodkinson, and P. Chantaranothai. 2022. New species and a revision of the genus *Peperomia* (Piperaceae) in Thailand. *Thai Forest Bulletin (Botany)* 50: 104-119.
343. Sykes, W.R. and C.J. West. 1996. New records and other information on the vascular flora of the Kermadec Islands. *New Zealand Journal of Botany* 34: 447-462.
344. Symmank et al. *Peperomia olens* 2008-042 (BR, GENT, LPB, MO).
345. Symmank et al. *Peperomia peltifolia* 2008-008 (BR, GENT, LPB).
346. Symmank et al. *Peperomia effusa* 2008-003 (BR, LPB).
347. Symmank et al. *Peperomia buchtienii* 2008-007 (BR, GENT, LPB).
348. Symmank et al. *Peperomia rusbyi* 2008-010 (BR, G, GENT, K, LPB, MO).
349. Symmank et al. *Peperomia pseudoumbilicata* 2008-011 (BR, GENT, LPB).
350. Symmank et al. *Peperomia pseudocobana* 2008-012 (BR, GENT, LPB).
351. Symmank et al. *Peperomia perlongipes* 2008-017 (BR, GENT, LPB).
352. Symmank et al. *Peperomia yanacachiana* 2008-022 (BR, GENT, LPB).
353. Symmank et al. *Peperomia ticunhuayana* 2008-028 (BR, GENT, LPB).
354. Symmank et al. *Peperomia crystallina* 2008-039 (BR, GENT, LPB, MO).
355. Symmank et al. *Peperomia silvarum* 2008-040 (BR, GENT, LPB).
356. Symmank et al. *Peperomia stuebelii* 2008-053 (BR, GENT, LPB).
357. Symmank et al. *Peperomia kuntzei* 2008-122 (BR, GENT, K, LPB, MO).
358. Symmank and G. Mathieu. *Peperomia marcapatana* 2008-060 (BR, GENT, LPB).
359. Symmank and G. Mathieu. *Peperomia steinbachii* 2008-061(BR, GENT, LPB, MO).
360. Symmank and G. Mathieu. *Peperomia tenuipeduncula* 2008-063 (BR, LPB).
361. Symmank and G. Mathieu. *Peperomia asperula* 2009-023 (BR, GENT, MO, USM).
362. Takamatsu. *Peperomia trukensis* 21 (NY).
363. Thomas, A. and J. Jameson. 2022. *Peperomia albertiae* (Piperaceae), a new species from India. *Kew Bulletin* 77: 341-346.
364. Tonduz. *Peperomia longibacca* 13958 (B).
365. Tonduz. *Peperomia pendula* (syn. of *P. macrostachyos*) 8433 (BR).
366. Tonduz. *Peperomia queserana* (syn. of *P. adscendens*) 12184 (BR).
367. Tonduz. *Peperomia sepicola* 17790 (G-DC).
368. Torres-Hormaza, T., et al. 2020. Flora de Bogotá: Piperaceae. *Pérez-Arbelaezia* 21: 50-99.
369. Trelease, W. 1927. The Piperaceae of Panama *Contributions from the United States National Herbarium, Smithsonian Institute, Washington* 26: 35 + VIII.
370. Trelease, W. 1928. Piperaceae hispaniolenses - III. *Repertorium specierum novarum regni vegetabilis* 25: 54-55.
371. Trelease, W. 1929. The Piperaceae of Costa Rica - *Peperomia*. *Contributions from the United States National Herbarium, Smithsonian Institute, Washington* 26: 185-226.
372. Trelease, W. 1934. Risultati della Spedizione Biologica Austriaca in Costarica (Cufodontis) - Piperaceae. *Archivo Botanico per la Sistematica, Fitogeografia e Genetica* 10: 26-27.
373. Trelease, W. and T.G. Yuncker. 1950. The Piperaceae of northern South America. University of Illinois Press, Illinois, USA.
374. Tropicos. 2024. Missouri Botanical Garden: Catalogue of the vascular plants of Ecuador.
375. Tropicos. 2024. Missouri Botanical Garden: Paraguay checklist.

376. Turner, I.M. 1995. A catalogue of the vascular plants of Malaya. National Parks Board Singapore Botanic Gardens, Singapore.
377. Ule. *Peperomia papillispica* 4858 (B).
378. Ule. *Peperomia roraimana* (syn. of *P. trinervula*) 8593 (B).
379. Urban, I. 1908. Plantae novae andinae imprimis Weberbauerianae III. *Botanische Jahrbücher fur Systematik* 40: 254-267.
380. Vareschi, V. 1970. Flora de los Paramos de Venezuela. Universidad de Los Andes, Mérida, Venezuela.
381. Vasquez Martinez, R. 1997. Flórula de las reservas biológicas de Iquitos, Perú: Allpahuayo-Mishana, Explornapo Camp, Explorama Lodge. 575- 576. Monographs in Systematic Botany from the Missouri Botanical Garden, St. Louis, Missouri, USA.
382. Véliz Pérez, M.E., F. Archila, and L.E. Vélasquez Méndez. 2017. New species of *Peperomia* (Piperaceae) from Guatemala. *Cactus-Aventures International* 2: 42-55.
383. Véliz. *Peperomia nigropunctata* 95.5107 (MO).
384. Verdcourt, B. 1996. Flora of tropical East Africa - Piperaceae Balkema, Rotterdam, Netherlands.
385. Vergara-Rodríguez, D., et al. 2017. Diversity, distribution, and conservation status of *Peperomia* (Piperaceae) in the state of Veracruz, Mexico. *Tropical Conservation Science* 10: 1940082917702383.
386. von Humboldt, A. and A. Bonpland. 1815. Nova genera et species plantarum 1. 60-74, illust. tab 2-17. Ex officina Christophori Plantini, Antwerp, Belgium.
387. Wagner, W.L., D.R. Herbst, and S.H. Sohmer. 1999. Manual of the flowering plants of Hawai'i. Revised edition. University of Hawai'i Press, Honolulu, Hawaii.
388. Wagner, W.L. and D.H. Lorence. 2002. Flora of the Marquesas Islands website. Website https://botany.si.edu/pacificislandbiodiversity/marquesasflora/index.htm [accessed March 2024].
389. Wendt, T. 2003. *Peperomia hobbitoides* (Piperaceae), a new species of karstophile from the rain forests of the Isthmus of Tehuantepec, Mexico. *Lundellia* 6: 37-43.
390. Wiggins, I.L., D.M. Porter, and E.F. Anderson. 1971. Flora of the Galapagos Islands. Stanford University Press, Stanford, USA.
391. Wight, R. 1853. Icones plantarum Indiae Orientalis :or figures of Indian plants. J.B. Pharoah, Madras, India.
392. Williams. *Peperomia sarcocarpa* (syn. of *P. angustata*) 382 (NY).
393. Woodson. *Peperomia casitana* (syn. of *P. adscendens*) 952 (NY).
394. Yamazaki, T. 1992. A new species of *Peperomia* from Ryukyus. *Journal of Japanese Botany* 67: 15-18.
395. Yuncker. *Peperomia caducipilosa* 5444 (NY).
396. Yuncker. *Peperomia circulifolia* 6361 (MO).
397. Yuncker. *Peperomia eripipunctulata* 6079 (NY).
398. Yuncker. *Peperomia mala* 5737 (NY).
399. Yuncker. *Peperomia montis-verticis* (syn. of *P. dendrophila*) 6244 (MO).
400. Yuncker. *Peperomia nebuligaudens* 6186 (NY).
401. Yuncker. *Peperomia novae-hispaniae* (syn. of *P. urocarpa*) 4848 (ILL).
402. Yuncker. *Peperomia novella* (syn. of *P. olivacea*) 6139 (MO).
403. Yuncker. *Peperomia perplexa* (syn. of *P. granulosa*) 4865 (MO).
404. Yuncker. *Peperomia praeteruentifolia* 5614 (ILL).
405. Yuncker. *Peperomia rhodophlebia* (syn. of *P. angustata*) 4896 (NY).
406. Yuncker. *Peperomia rinconensis* (syn. of *P. heterophylla*) 6080 (ILL).
407. Yuncker, T.G. 1937. Observations on the teratology of the genus *Peperomia*. *Occasional Papers of the Bernice Pauahi Bishop Museum, Honolulu, Hawaii* 13: 5-9.
408. Yuncker, T.G. 1940. Flora of the Aguan Valley and the coastal regions near La Ceiba, Honduras. *Publications of the Field Columbian Museum, Botanical Series* 9: 245 - 346.

409. Yuncker, T.G. 1949. Additional notes on the Fijian species of *Peperomia*. *Journal of the Arnold Arboretum* 30: 443-449.
410. Yuncker, T.G. 1950. Flora of Panama. Part IV. Fascicle I. *Annals of the Missouri Botanical Garden* 37: 1-120.
411. Yuncker, T.G. 1952. New species of Peruvian and Colombian Piperaceae. *American Journal of Botany* 39: 633-636.
412. Yuncker, T.G. 1953. The Piperaceae of Argentina, Bolivia, and Chile. *Lilloa* 27: 97-461.
413. Yuncker, T.G. 1954. A new ornamental *Peperomia* from Venezuela. *Brittonia* 8: 61-63.
414. Yuncker, T.G. 1955. Plants collected in Ecuador by W.H. Camp. Piperaceae. 9: 147–170.
415. Yuncker, T.G. 1956. Additions to the Fijian species of *Peperomia*. *Bulletin of the Torrey Botanical Club* 83: 300-304.
416. Yuncker, T.G. 1966. *Piper* and *Peperomia* (Piperaceae) new to Panama. *Annals of the Missouri Botanical Garden* 53: 261-264.
417. Zanotti, C. and F. Biganzoli. 2010. *Peperomia nitida* y *Peperomia armondii* (Piperaceae), nuevos registros para la Argentina. *Darwiniana* 48: 210-213.
418. Zanotti, C.A., M.A. Suescún, and G. Mathieu. 2012. Sinopsis y novedades taxonomicas de *Peperomia* (Piperaceae) en la Argentina. *Darwiniana* 50: 124-147.
419. Zoghbi, M.G.B., et al. 2005. *Peperomia circinnata* link and *Peperomia rotundifolia* (L.) Kunth growing on different host-trees in Amazon: volatiles and relationship with bryophytes. *Biochemical Systematics and Ecology* 33: 269-274.
